# An ancient mitochondrial program tunes translation to haem availability

**DOI:** 10.1038/s41586-026-10885-x

**Published:** 2026-08-05

**Authors:** Xiang Zhang, Max-Hinderk Schuler, Gonca Çetin, Eva-Maria Eckl, Lara Rheinemann, Julia Mergner, Barbara Steigenberger, Andreas Pichlmair, Lucas T. Jae

**Affiliations:** 1 Gene Center and Department of Biochemistry, Ludwig-Maximilians-Universität München, Munich, Germany; 2 Institute of Virology, School of Medicine and Health, Technical University of Munich, Munich, Germany; 3 Bavarian Center for Biomolecular Mass Spectrometry at University Hospital Rechts der Isar (BayBioMS@MRI), School of Medicine and Health, Technical University of Munich, Munich, Germany; 4 Mass Spectrometry Core Facility, Max Planck Institute of Biochemistry, Martinsried, Germany; 5 Institute of Virology, Helmholtz Center Munich, Neuherberg, Germany; 6 German Center for Infection Research (DZIF), Munich partner site, Munich, Germany

**Keywords:** Stress signalling, Mitochondria, Proteins, Metabolism, Mutagenesis

## Abstract

Anaemia is a major global health burden that affects one-quarter of the human population and annually accounts for over 50 million years of healthy life lost^1^. It arises from nutritional iron deficiency, hereditary disorders (including thalassaemia and sickle cell disease) and malaria, and is characterized by haemoglobin imbalances^2^. Haem—the active component of haemoglobin—is both essential and potentially toxic, which necessitates tight control of levels. However, the molecular circuitry that monitors haem levels remains obscure. The cytosolic eIF2α kinase HRI counteracts anaemia amid iron deficiency or thalassaemia^3,4^ by acting as a gatekeeper of translation during erythroid differentiation, which has been attributed to its haem-binding ability^5^. Here we uncover that haem scarcity is sensed inside mitochondria through an OMA1–DELE1 axis. Mechanistically, haem deficiency triggers OMA1-dependent mitochondrial release of DELE1. In the cytosol, DELE1 releases inhibitory haem from HRI, which enables modifications in a crucial disordered segment of the kinase. We demonstrate that this sensor–actuator operates across human tissues, including erythroid progenitors, and is evolutionarily conserved down to bloodless invertebrates, thus predating the emergence of haemoglobin-based oxygen transport. Notably, pharmacological manipulation of this system enhances fetal globin expression—a central therapeutic objective in haemoglobinopathies. Together, these results reveal a primordial sentinel system that safeguards against haem-related toxicity from the single-cell to the organismic scale.

## Abstract (web summary)

An evolutionarily conserved mitochondrial haem-sensing mechanism that predates the emergence of haemoglobin-based oxygen transport could have therapeutic value in iron deficiency disorders.

## Main

Iron is the most biologically used transition metal and near-universally essential across all domains of life^6–8^. In humans, most iron is bound as haem in haemoglobin in erythrocytes^8^. Haem is synthesized in a multistep process that begins and concludes in mitochondria^9^, but mechanisms for extracellular haem uptake also exist^10^. Together with iron–sulfur clusters^11^, haem represents the principal functional pool of cellular iron that supports redox reactions, electron and oxygen transport and signalling^7^. However, like free iron, excess haem is cytotoxic and must be tightly regulated^12^. A factor decoding the cellular haem status (hereafter referred to as haemeostasis) and accordingly tuning translation was proposed over half a century ago based on experiments with reticulocytes (precursors of red blood cells)^13^. This pioneering work demonstrated that the addition of haemin, a cell-permeable haem source, to reticulocyte lysates promotes ribosome activity, which indicated the existence of a haem-inhibited translational suppressor. This factor was later identified as haem-regulated inhibitor (HRI), a serine/threonine kinase that phosphorylates eukaryotic initiation factor 2α (eIF2α)^14,15^. eIF2α phosphorylation is a hallmark of the integrated stress response (ISR), which blocks translation for most cellular mRNAs while favouring biogenesis of distinct proteins, including activating transcription factor 4 (ATF4) and C/EBP homologous protein (CHOP)^16^. The key function of HRI in tuning globin synthesis to haem availability is currently understood to be enabled by direct haem sensing in the cytosol^5,17^. Initially considered erythroid-specific^18^, HRI was subsequently found to be broadly expressed and to function beyond the development of red blood cells^19,20^. Recently, we and others discovered that HRI acts as a critical kinase in a relay system that communicates mitochondrial defects to the cytosol^21,22^. The stress-activated protease OMA1 cleaves the mitochondrial protein DELE1 during import, which triggers its redistribution to the cytosol. Cytosolic short DELE1 (S-DELE1) binds to and activates HRI through an unknown mechanism to initiate the mitochondrial ISR. Neither this interaction^21^ nor DELE1-mediated ISR signalling is quenched by haemin^22^, which suggests that DELE1 and haem may represent unrelated HRI inputs. Notably, proteins with predicted structural similarity^23^ to HRI can be found in lower organisms that lack haemoglobin biology, which hints at more ancient functions of the kinase.

## HRI haem sensing relies on mitochondria

**Fig. 1:**
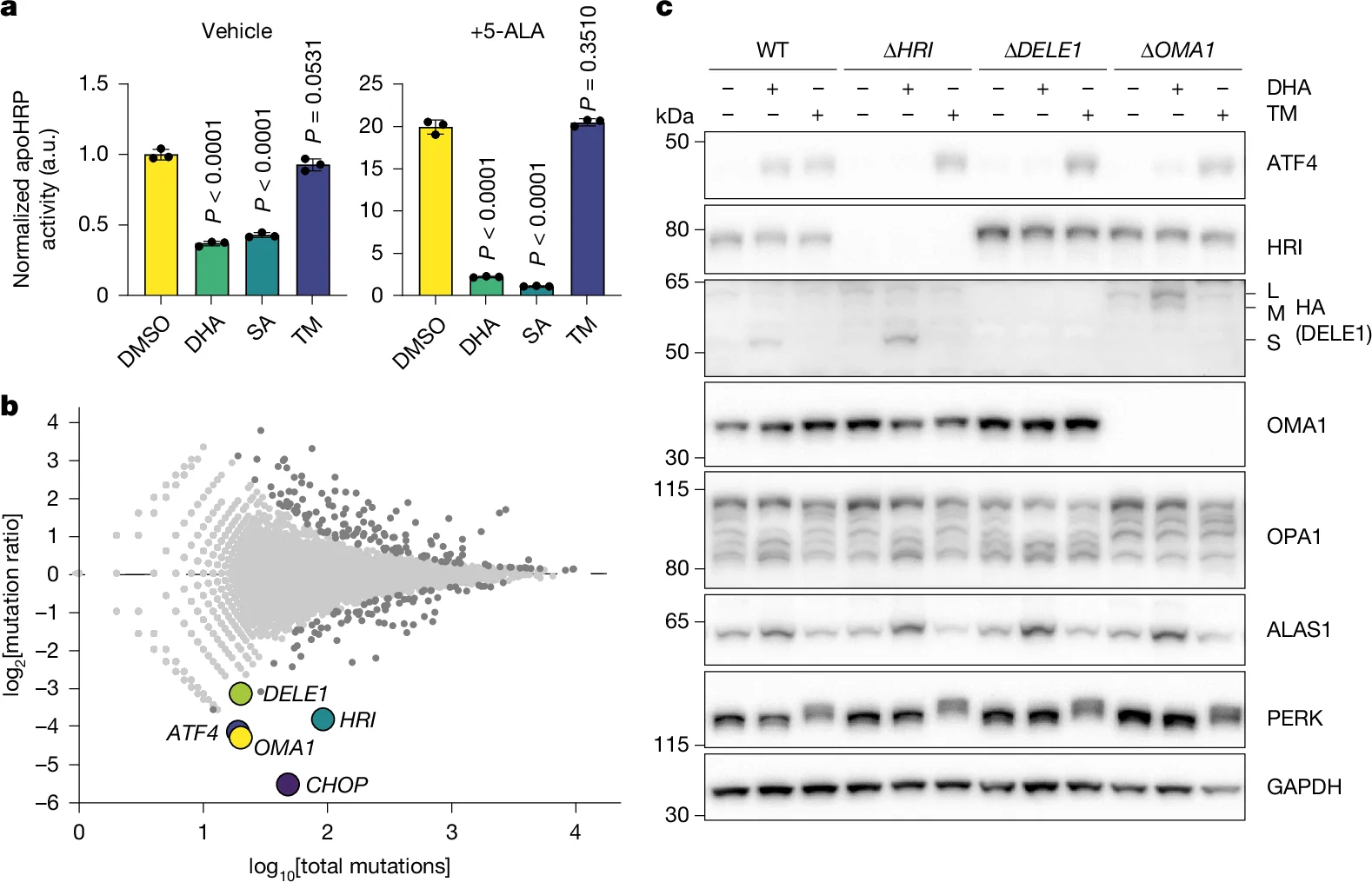
Haem sensing by HRI is a mitochondrial phenotype routed through OMA1 and DELE1. **a**, Haem levels determined by apoHRP activity in 293T cells treated for 6 h with DHA, SA or tunicamycin (TM). Where indicated, 5-ALA was used to stimulate haem synthesis (mean ± s.d. of *n* = 3 independent biological replicates; one-way analysis of variance (ANOVA) with Dunnett’s multiple comparisons test). a.u., arbitrary units. **b**, Haploid genetic screen for regulators of ISR signalling induced by haem starvation based on *CHOP*^*Neon*^ (*n* = 2.19 × 10^7^ interrogated single cells). Genes (dots) significantly enriched for mutations are shown in dark grey or coloured (two-sided Fisher’s exact test, false discovery rate (FDR)-corrected *P* = < 0.05). **c**, Immunoblot of ISR signalling in 293T (kidney) *DELE1*^*HA*^ WT and knockout cells treated for 6 h with DHA or TM. L-DELE1, long DELE1; M-DELE1, mature DELE1.

**Extended Data Fig. 1:**
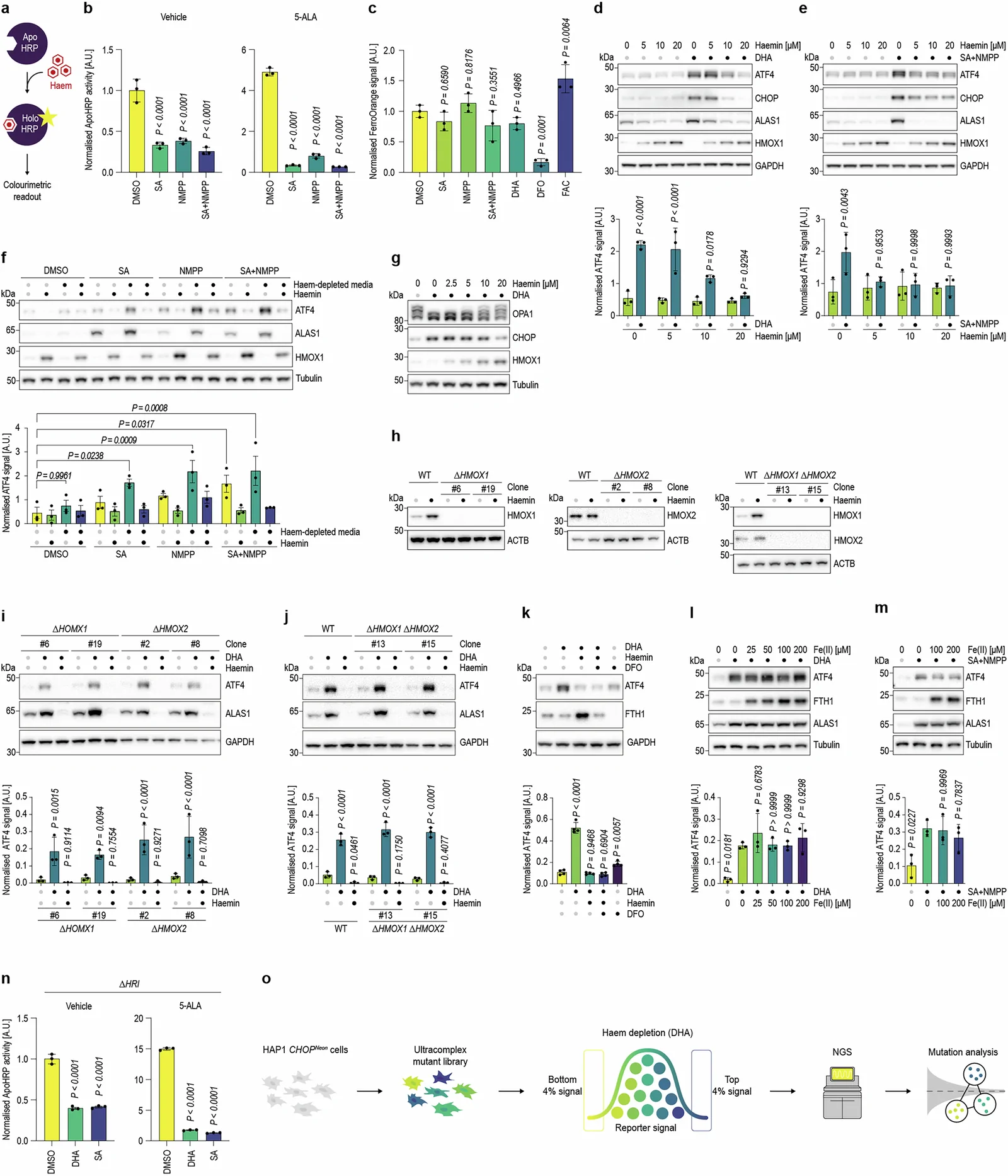
Different modes of haem depletion activate HRI, reversed by haem but not iron. **a**, Schematic of apoHRP-based haem detection. **b**, Haem levels determined by apoHRP activity in 293T cells treated for 6 h with SA, NMPP, or the combination. Where indicated, 5-ALA was used to stimulate haem synthesis. **c**, Iron levels determined by FerroOrange fluorescence in 293T cells treated for 6 h with DHA or ferric ammonium citrate (FAC), and for 16 h with Deferoxamine (DFO), SA, NMPP, or the combination. **d**, **e**, ISR signalling in 293T cells treated for 6 h with DHA (**d**) or for 24 h with SA and NMPP in haem-depleted media (**e**) in presence or absence of haemin. **f**, ISR signalling in 293T cells treated for 24 h with SA, NMPP, or the combination in haem-depleted or normal media. **g**, OPA1 processing in 293T cells treated with DHA as in (d). **h**, Characterization of 293T *HMOX1* and *HMOX2* single and double knockout cells. **i-k**, ISR signalling in 293T WT vs. *HMOX1/2* knockout cells (**i, j**) or 293T WT cells (**k**) treated with DHA as in (d). Where indicated, samples were co-treated with haemin or DFO. **l**, **m**, ISR signalling in 293T cells treated with DHA (**l**) as in (d) or SA + NMPP (**m**) as in (b) in presence of ferrous iron. **n**, Haem levels as in (b) but in 293T *∆HRI* cells. **o**, Schematic of the haploid genetic screen. Mean ± s.d. of *n* = 3 (**b**-**f**, **i**, **j**, **l**-**n**) or *n* = 4 (**k**) independent biological replicates; one-way ANOVA with Dunnett’s (**b**, **c**, **k**, **n**) or Tukey’s (**l**, **m**) multiple comparisons tests; two-way ANOVA with Šídák’s (**d**, **e**), Dunnett’s (**f**), or Tukey’s (**i**, **j**) multiple comparisons tests.

To elucidate how HRI responds to haem deficiency, we first established methods for haem depletion and detection. One haem depletion strategy is to inhibit its synthesis using succinylacetone (SA) or *N*-methyl protoporphyrin IX (NMPP). SA competes with the precursor δ-aminolevulinic acid (5-ALA), whereas NMPP inhibits ferrochelatase (FECH) at the final step of haem biogenesis^24,25^. To measure cellular haem, we established a colorimetric assay based on horseradish peroxidase (HRP), which requires haem as a cofactor^26^ (Extended Data Fig. 1a). This method detected haem depletion induced by SA or NMPP, with differences further enhanced by 5-ALA supplementation (Fig. 1a and Extended Data Fig. 1b). To deplete cellular haem beyond de novo synthesis inhibition, we considered the antimalarial drug dihydroartemisinin (DHA), which chemically reacts with haem^27^ and stimulates HRI activity^28^. Brief treatment with DHA resulted in haem depletion, whereas free iron levels remained largely unaffected by DHA, SA or NMPP (Fig. 1a and Extended Data Fig. 1c). As expected, perturbation of haemeostasis triggered ISR signalling, which was quenched by haemin supplementation (Extended Data Fig. 1d–g). Quenching occurred independently of haem oxygenases, which liberate iron from haem^29^, and was not recapitulated by iron supplementation (Extended Data Fig. 1h–m). Finally, acute treatment of cells with the endoplasmic reticulum stressor tunicamycin did not cause haem deficiency, and SA-induced or DHA-induced haem loss persisted in HRI-deficient cells (Fig. 1a and Extended Data Fig. 1n).

**Extended Data Fig. 2:**
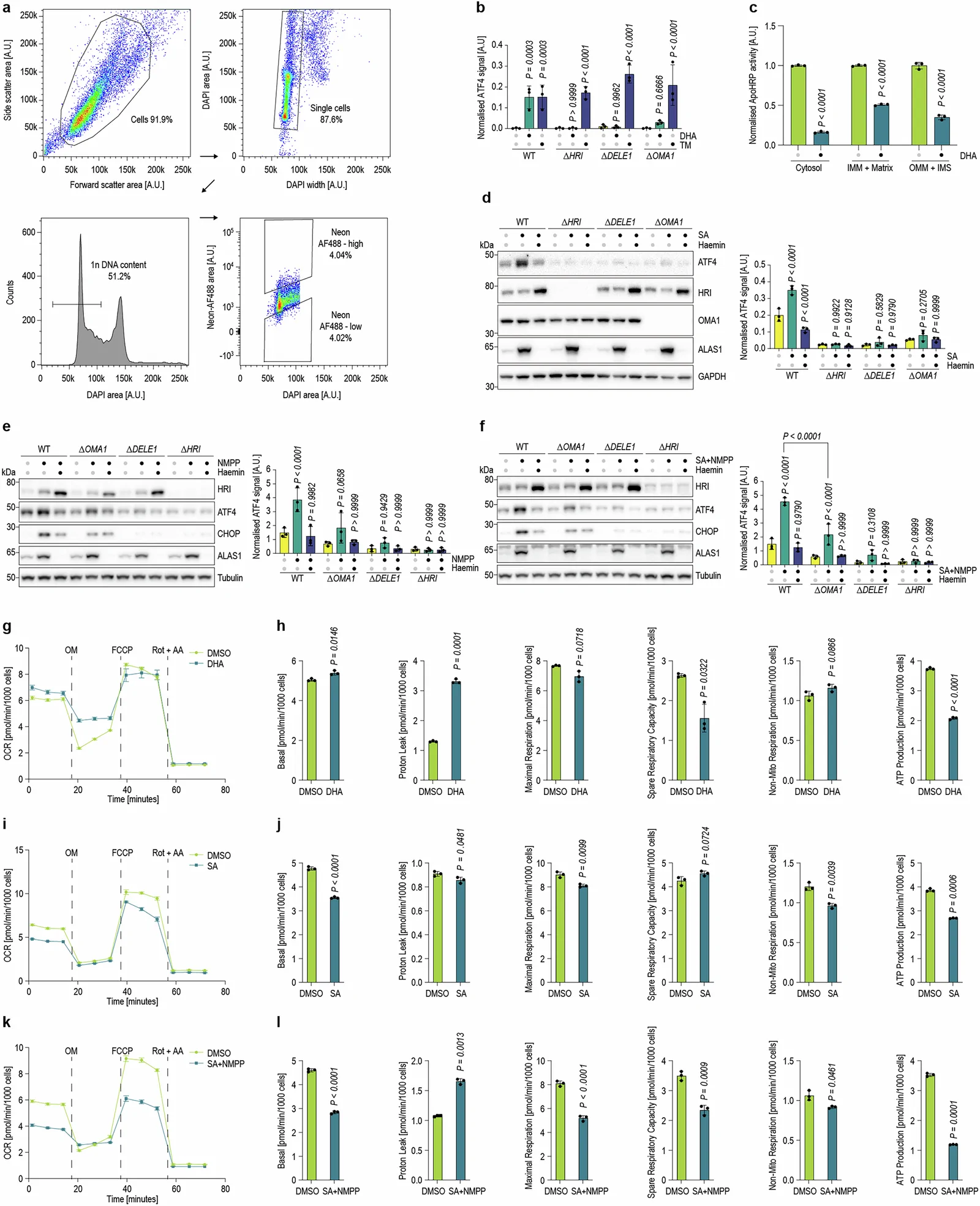
Haem sensing by HRI depends on OMA1 and DELE1. **a**, Gating strategy for the haploid genetic screen. **b**, Quantification of experiments relating to Fig. 1c. **c**, Haem levels determined by apoHRP activity in the indicated cellular compartments of 293T cells treated with DHA for 6 h. Outer mitochondrial membrane (OMM); intermembrane space (IMS); inner mitochondrial membrane (IMM). **d**-**f**, ISR signalling in 293T WT vs. knockout cells treated with SA (**d**), NMPP (**e**), or the combination (**f**) for 24 h. **g**-**l**, Mitochondrial respiratory chain function assessed by Seahorse assay in 293T cells treated for 4 h with DHA (**g**, **h**), or 24 h with SA (**i**, **j**), or SA + NMPP (**k**, **l**). Mean ± s.d. of *n* = 3 independent biological replicates (**b**-**l**); two-way ANOVA with Tukey’s (**b**, **d**) or Šídák’s (**c**, **e**, **f**) multiple comparisons tests; Welch’s t-test (**h**, **j**, **l**).

**Extended Data Fig. 3:**
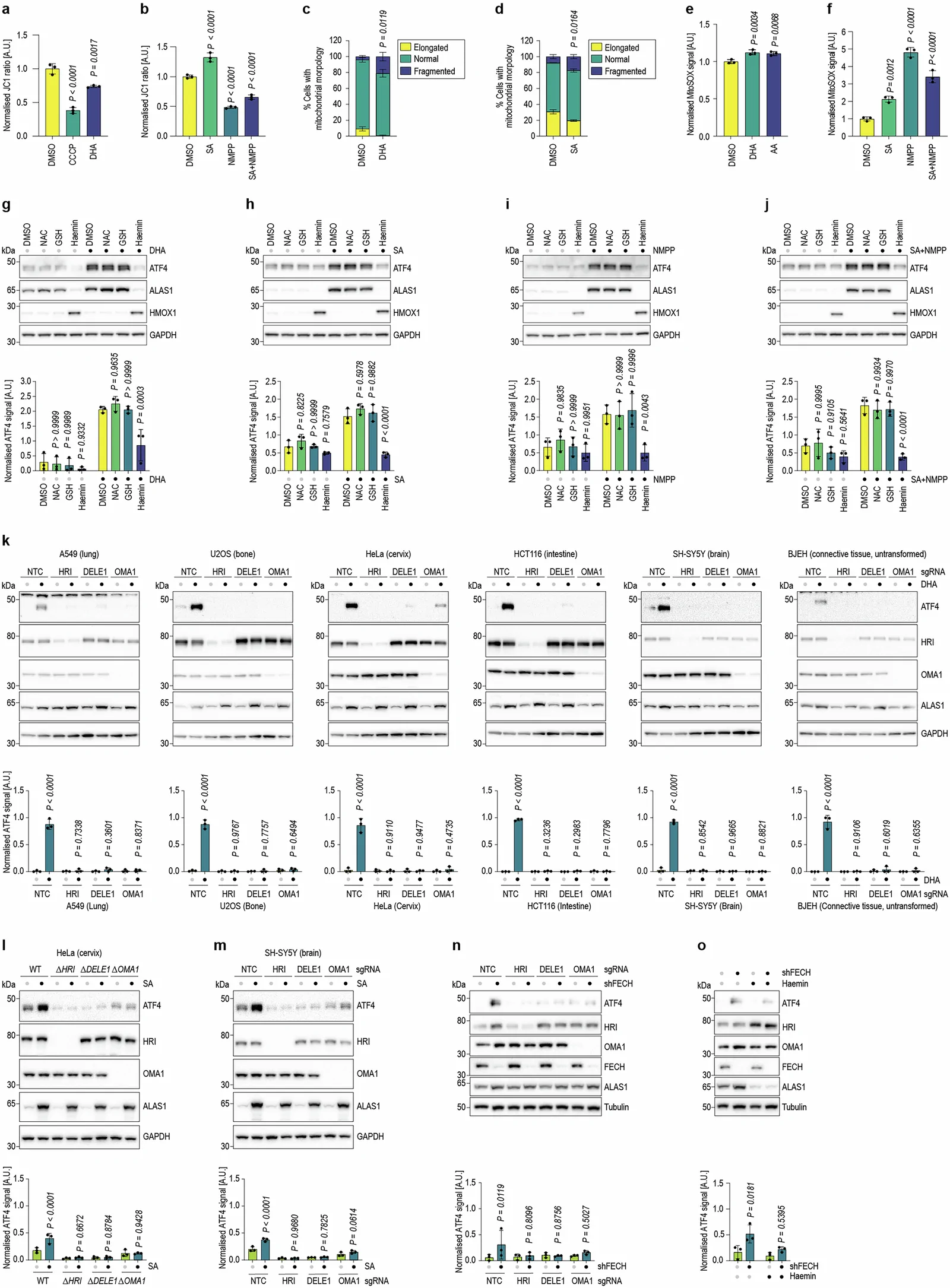
Haem depletion impairs mitochondria and activates HRI via OMA1-DELE1, independent of ROS. **a**, **b**, Mitochondrial polarization assessed by JC-1 fluorescence in 293T cells treated for 4 h with DHA or CCCP (**a**) or for 20 h with SA, NMPP, or the combination (**b**). **c**, **d**, Mitochondrial morphology in BJEH cells treated as in (a, b). *P*-values compare percentage of fragmented mitochondria. **e**, **f**, Mitochondrial reactive oxygen species (ROS) production assessed by MitoSOX fluorescence in 293T cells treated for 4 h with DHA or antimycin A (AA) (**e**) or for 20 h with SA, NMPP, or the combination (**f**). **g-j**, ISR signalling in 293T cells treated for 4 h with DHA (**g**) or for 24 h with SA (**h**), NMPP (**i**), or the combination (**j**). Where indicated, cells were pre-treated for 2 h with N-acetylcysteine (NAC), reduced glutathione (GSH), or haemin. **k-m**, ISR signalling in the indicated cell systems with the specified genotypes (**l**) or upon exposure to the indicated single-guide (sg)RNAs (**k, m**) and treated with DHA for 6 h (**k**), or with SA for 24 h (**l, m**); non-targeting control (NTC). **n**, **o**, ISR signalling upon genetic knockdown of FECH by short-hairpin (sh)RNA in 293T cells exposed to the indicated sgRNAs (**n**) or in presence or absence of haemin (**o**). Mean ± s.d. of *n* = 3 independent biological replicates (**a-o**); one-way ANOVA with Dunnett’s multiple comparisons test (**a**, **b**, **e**, **f**); two-way ANOVA with Šídák’s (**c**, **d**, **g**-**j**, **o**) or Tukey’s (**k**-**n**) multiple comparisons tests.

We therefore sought to dissect how HRI processes haem deprivation. We performed a genome-wide screen based on the HRI downstream factor CHOP^21^ (Extended Data Figs. 1o and 2a). As expected, this screen identified a strong requirement for HRI and ATF4 in DHA-induced CHOP expression. However, it also revealed the mitochondrial proteins OMA1 and DELE1 as critical factors (Fig. 1b and Supplementary Data 1). This result suggests that HRI activation by DHA represents a mitochondrial phenotype that depends on the OMA1–DELE1 axis. Indeed, DHA-induced haem deficiency extended to the mitochondrial compartment, and clonal deletion of *OMA1* or *DELE1* confirmed predictions from the screen (Fig. 1c and Extended Data Fig. 2b,c). The dependence on OMA1 and DELE1 was similarly observed when haem was depleted with SA or NMPP (Extended Data Fig. 2d–f). This was in spite of the partially distinct effects of these compounds on mitochondrial function, which is consistent with their differing mechanisms of haem depletion (Extended Data Figs. 2g–l and 3a–d). Although mitochondrial impairment was accompanied by increased reactive oxygen species (ROS), ROS were not the cause of HRI activation (Extended Data Fig. 3e–j). Together, these results identify lack of haem itself as the common driving force behind an OMA1–DELE1-dependent mitochondrial signal. Notably, the requirement for OMA1 and DELE1 was conserved across all human cell systems tested, spanning multiple tissue origins and untransformed cells (Extended Data Fig. 3k–m). Finally, genetic inhibition of haem synthesis via FECH also activated HRI in a manner that depended on OMA1 and DELE1 (Extended Data Fig. 3n,o). In summary, these results suggest that—contrary to the current paradigm—the ability of HRI to respond to haem deficiency involves mitochondrial signals provided by OMA1 and DELE1 across different human tissues.

## HRI dimers bind up to two DELE1 molecules

**Fig. 2:**
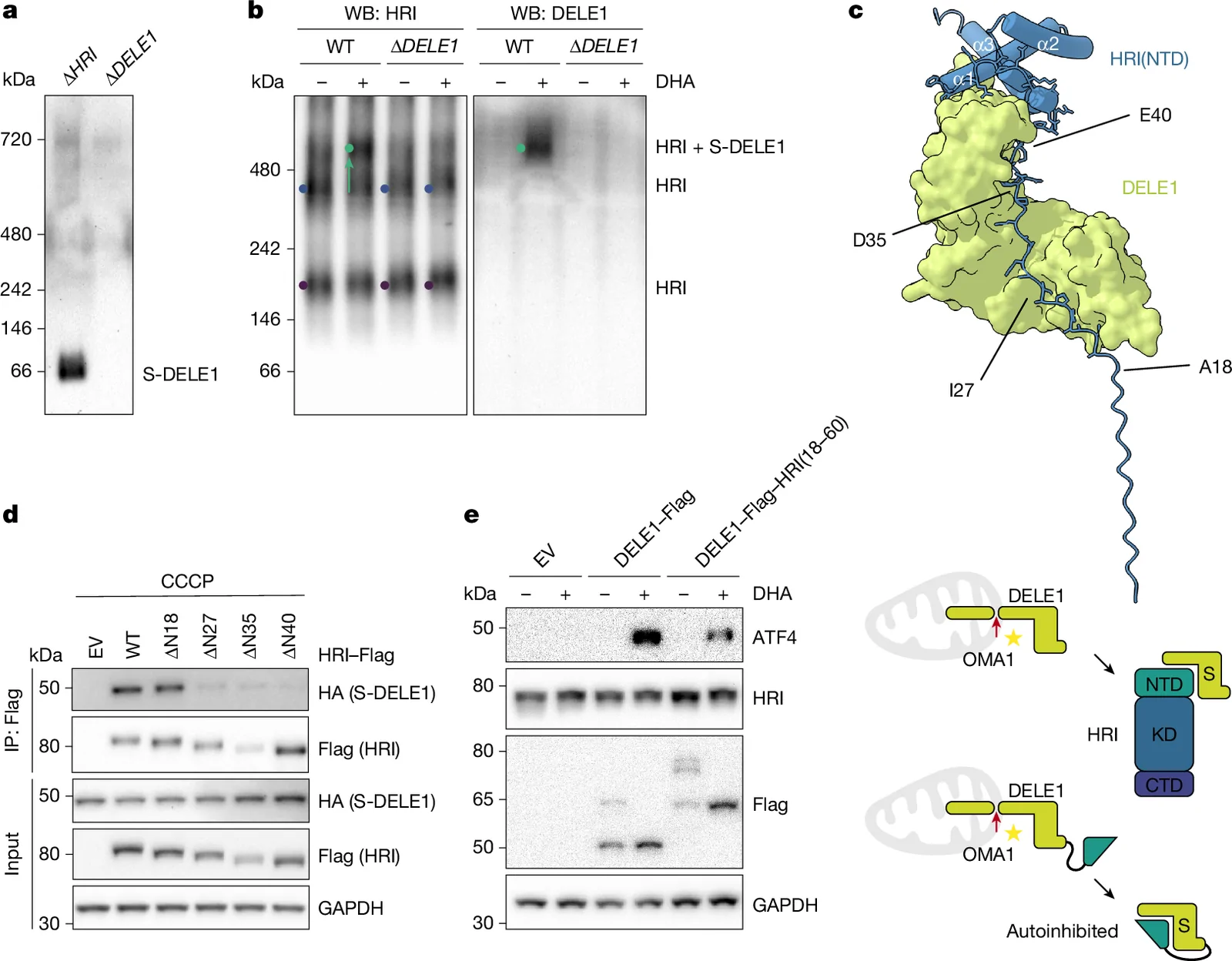
S-DELE1 promotes maturation of inactive HRI dimers. **a**, Blue native (BN)-PAGE analysis of endogenous S-DELE1 in cytosolic extracts of 293T ∆*HRI* cells treated for 2 h with CCCP. **b**, BN-PAGE analysis of HRI assemblies in cytosolic extracts of 293T WT and ∆*DELE1* cells treated for 8 h with DHA. Purple dots indicate ~200 kDa HRI species, blue dots indicate ~400 kDa HRI species, green dots indicate ~500 kDa DELE1–HRI complexes, and the arrow indicates the size shift of the HRI complex upon S-DELE1 binding. **c**, AlphaFold prediction of DELE1 bound to the HRI NTD. **d**, Binding of endogenous S-DELE1 to N-terminal HRI truncation mutants expressed in *DELE1*^*HA*^ ∆*HRI* cells treated as in **a**. EV, empty vector. **e**, Blot (left) and schematic (right) of ISR signalling in 293T ∆*DELE1* cells expressing WT DELE1 or a DELE1 variant carrying the HRI-derived DELE1-binding peptide, treated as in **b**.

**Extended Data Fig. 4:**
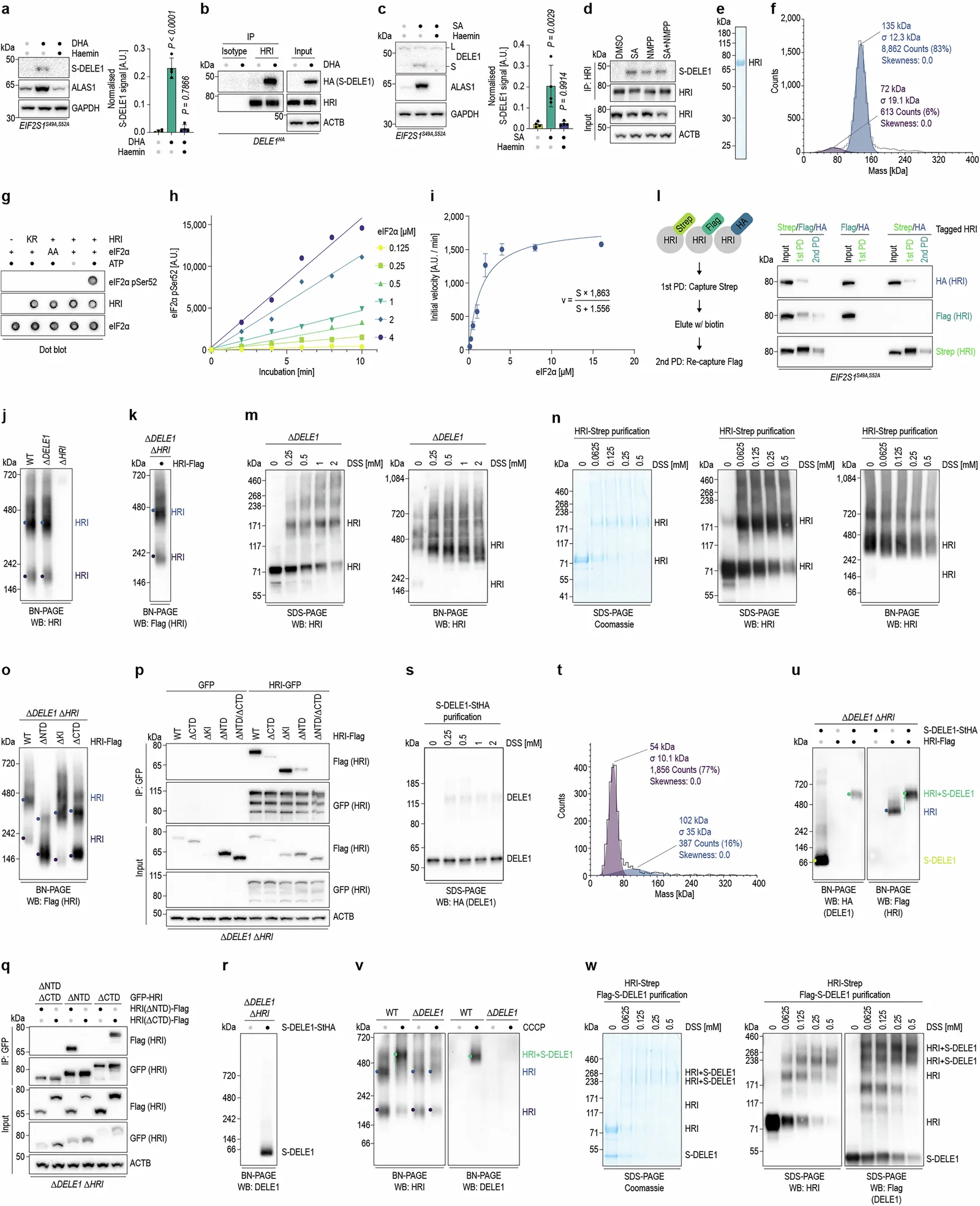
Analysis of HRI complexes in presence or absence of S-DELE1. **a**, Analysis of DELE1 cleavage in 293T cells treated for 6 h with DHA in presence or absence of haemin. **b**, DELE1 binding to HRI from 293T cells treated as in (a), assessed by co-IP. **c**, Analysis of DELE1 cleavage in 293T cells treated for 24 h with SA in presence or absence of haemin. **d**, DELE1 binding to HRI from 293T cells treated for 24 h with SA, NMPP, or the combination, assessed by co-IP. **e**, **f**, Coomassie staining (**e**) and mass photometry analysis (**f**) of bacterially purified HRI. **g**-**i**, Dot blotting analysis (**g**) and densitometric quantification (**h**, **i**) of eIF2α or eIF2α(S49A,S52A) (‘AA’) phosphorylation by WT HRI or HRI(K196R) (‘KR’) in vitro. Kinetic constants were determined by fitting to the Michaelis-Menten equation. **j**, **k**, Blue native (BN)-PAGE analysis of endogenous HRI in the cytosol of 293T cells of the indicated genotypes (**j**) or ectopically expressed in 293T *ΔDELE1/ΔHRI* (DKO) cells (**k**). **l**, Analysis of HRI complex stoichiometry by sequential affinity purification. First pulldown (PD): Streptavidin; second PD: anti-Flag; 0.25% input; 5% 1^st^ PD; 25% 2^nd^ PD. **m**, **n**, Denaturing and BN-PAGE analysis of HRI assemblies in the cytosol of 293T *∆DELE1* cells (**m**) or of HRI purified from 293T *EIF2S1*^*S49A,S52A*^ cells (**n**). Crosslinking was performed with the indicated DSS concentrations. **o**, BN-PAGE analysis of the indicated HRI mutants expressed in 293T DKO cells. **p**, **q**, Mapping of HRI dimerization interfaces by co-IP. **r**, BN-PAGE analysis of ectopically expressed S-DELE1 in the cytosol of DKO cells; Strep-HA tag (StHA). **s, t**, Denaturing-PAGE after crosslinking with DSS (**s**) and mass photometry analysis (**t**) of S-DELE1 purified from 293T *EIF2S1*^*S49A,S52A*^/*ΔHRI* cells. **u**, BN-PAGE analysis of HRI expressed in 293T DKO cells in presence or absence of S-DELE1. **v**, BN-PAGE analysis of HRI and DELE1 in 293T WT and *∆DELE1* cells treated with CCCP for 2 h. **w**, Denaturing-PAGE analysis of DELE1:HRI assemblies purified from 293T *EIF2S1*^*S49A,S52A*^ cells and crosslinked with DSS. Data in (**h**) show a representative of three independent biological replicates. Mean ± s.d. of *n* = 4 (**a**, **c**) or *n* = 3 (**i**) independent biological replicates; one-way ANOVA with Dunnett’s multiple comparisons test (**a, c**).

**Extended Data Fig. 5:**
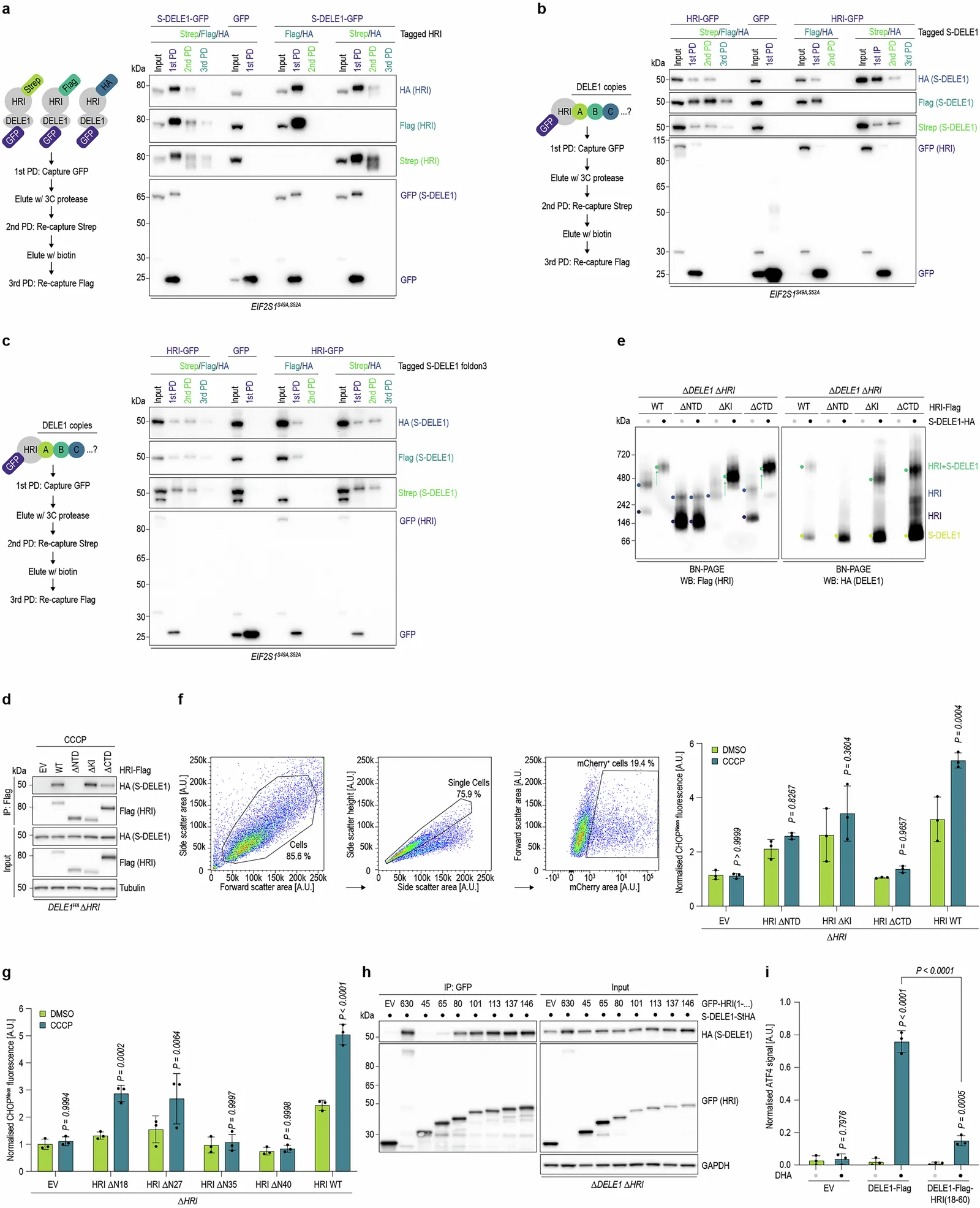
Functional characterization of DELE1–HRI interactions. **a**-**c**, Analysis of HRI–DELE1 complex stoichiometry by sequential affinity purification/elution. First pulldown (PD): GFP; second PD: Streptavidin; third PD: anti-Flag; 0.25% input; 5% 1^st^ PD; 25% 2^nd^ PD; and 100% 3^rd^ PD. Where indicated, S-DELE1 was tagged with the trimer-inducing foldon3 tag (**c**). **d**, Binding of endogenous S-DELE1 to the indicated HRI mutants in response to CCCP. EV, empty vector. **e**, BN-PAGE analysis of the indicated HRI mutants in presence or absence of S-DELE1. **f**, **g**, FACS gating strategy and analysis of CHOP^Neon^ expression in HAP1 *∆HRI* cells expressing the indicated HRI mutants and treated for 8 h with DMSO or CCCP. **h**, Binding of ectopically expressed S-DELE1 to the indicated GFP-fused HRI fragments. **i**, Quantification of experiments relating to Fig. 2e. Mean ± s.d. of *n* = 3 independent biological replicates (**f**, **g**, **i**); two-way ANOVA with Šídák’s (**f, g**) or Tukey’s (**i**) multiple comparisons tests.

Reticulocyte lysate data led to the development of a model in which haem deficiency causes HRI to mature from a ‘pro-inhibitor’ into its active form^30^. We wondered whether mitochondria-released DELE1 might mediate this conversion. Consistent with the genetic requirement for *OMA1* and *DELE1* in haem-related HRI activation, DHA, SA or NMPP caused haem-dependent mitochondrial release of cleaved S-DELE1 into the cytosol, where it engaged HRI (Extended Data Fig. 4a–d). Next, we characterized inactive HRI relative to the active DELE1-bound form. In agreement with previous reports^31^, HRI is dimeric in vitro, with a dimer mass of around 135 kDa (Extended Data Fig. 4e–i). When assessed by native gel electrophoresis, HRI migrated predominantly at about 400 kDa, with a minor species around 200 kDa, independent of the DELE1 status in unstressed cells (Extended Data Fig. 4j,k). This result is in line with the idea that interaction occurs only after cytosolic DELE1 accumulation^21,22^. The slow migration behaviour of HRI is consistent with previous reports and reminiscent of other eIF2α kinases^5,32^. It was not explained by additional HRI copies, as sequential co-immunoprecipitation (co-IP) and crosslinking experiments revealed that the majority of HRI is also dimeric in cells (Extended Data Fig. 4l–n). To assess which regions of HRI mediate its dimerization, we generated deletions in the three domains of HRI not directly involved in its kinase activity: the N-terminal domain (NTD), the kinase insert (KI) domain and the C-terminal domain (CTD). Deletion of either the NTD or the CTD impaired complex formation, but neither fully abrogated it (Extended Data Fig. 4o). Targeted co-IP experiments confirmed that HRI dimerization involves both homotypic NTD–NTD and CTD–CTD interactions (Extended Data Fig. 4p,q). We next explored how HRI complexes are remodelled by S-DELE1. The structured portion of DELE1 can form octameric assemblies in vitro, which have been proposed as platforms for HRI activation^33^. However, working with the endogenous protein, virtually all of S-DELE1 migrated close to its theoretical monomeric mass in native gel electrophoresis in the absence of HRI (Fig. 2a). Even after exogenous overexpression, only a small fraction of S-DELE1 migrated more slowly, which indicates that S-DELE1 is predominantly monomeric in the cytosol (Extended Data Fig. 4r–t). By contrast, in presence of HRI, S-DELE1 was recruited into an approximately 500 kDa complex and HRI shifted into a complex of similar size (Extended Data Fig. 4u). This shift also occurred after mitochondrial perturbation and required DELE1, which indicated the formation of a hetero-oligomeric DELE1–HRI complex (Fig. 2b and Extended Data Fig. 4v). To elucidate its composition, we first performed molecular crosslinking in situ followed by denaturing gel electrophoresis. In the presence of S-DELE1, otherwise dimeric HRI migrated at a higher molecular weight, in line with S-DELE1 recruitment (Extended Data Fig. 4w). This setup revealed two principal species, which probably correspond to HRI dimers bound to either one or two copies of S-DELE1. Second, we determined the maximum number of HRI and S-DELE1 molecules present in the complex through sequential capture experiments. In agreement with the crosslinking data, we detected two copies of HRI when using S-DELE1 as bait (Extended Data Fig. 5a). When HRI was used as bait, a maximum of two copies of S-DELE1 co-precipitated (Extended Data Fig. 5b). This result was not due to a detection limit, as the assay readily detected three copies of S-DELE1 when it was artificially rendered trimeric by a foldon3 tag^34^ (Extended Data Fig. 5c).

Previously, we and others mapped the HRI-interacting region of S-DELE1 to its tetratricopeptide repeat (TPR) segment^21,22^. Notably, TPR1 was dispensable for HRI binding but required for ISR signalling^21^. This result suggests that this region has a crucial role in HRI activation, whereas HRI binding involves additional downstream sequences. A computational prediction^23^ of the DELE1–HRI interface supports these data. In this model, an HRI N-terminal coil inserts into the DELE1 TPR groove as part of a larger HRI NTD interface (Fig. 2c). We first confirmed the S-DELE1–HRI NTD interaction by co-IP and native gel electrophoresis (Extended Data Fig. 5d,e). Notably, deletion of any HRI domain (NTD, KI or CTD) blunted kinase activity, which indicated that each is required for function (Extended Data Fig. 5f). Second, we confirmed that the first 18 HRI residues are dispensable for S-DELE1 binding and kinase activity (Fig. 2d and Extended Data Fig. 5g), whereas the downstream coil plus helices 1–3 are sufficient for strong interaction with S-DELE1 (Extended Data Fig. 5h). Finally, we reasoned that fusing the DELE1-binding HRI coil region directly to the DELE1 C terminus should result in DELE1 autoinhibition through competition for the TPR groove in *cis*, which we indeed observed (Fig. 2e and Extended Data Fig. 5i). Together, these findings indicate that activation of the inactive HRI dimer is mediated by the recruitment of up to two S-DELE1 copies through interactions between portions of the TPR segment and the HRI NTD.

## DELE1 antagonizes HRI inhibition by haem

**Fig. 3:**
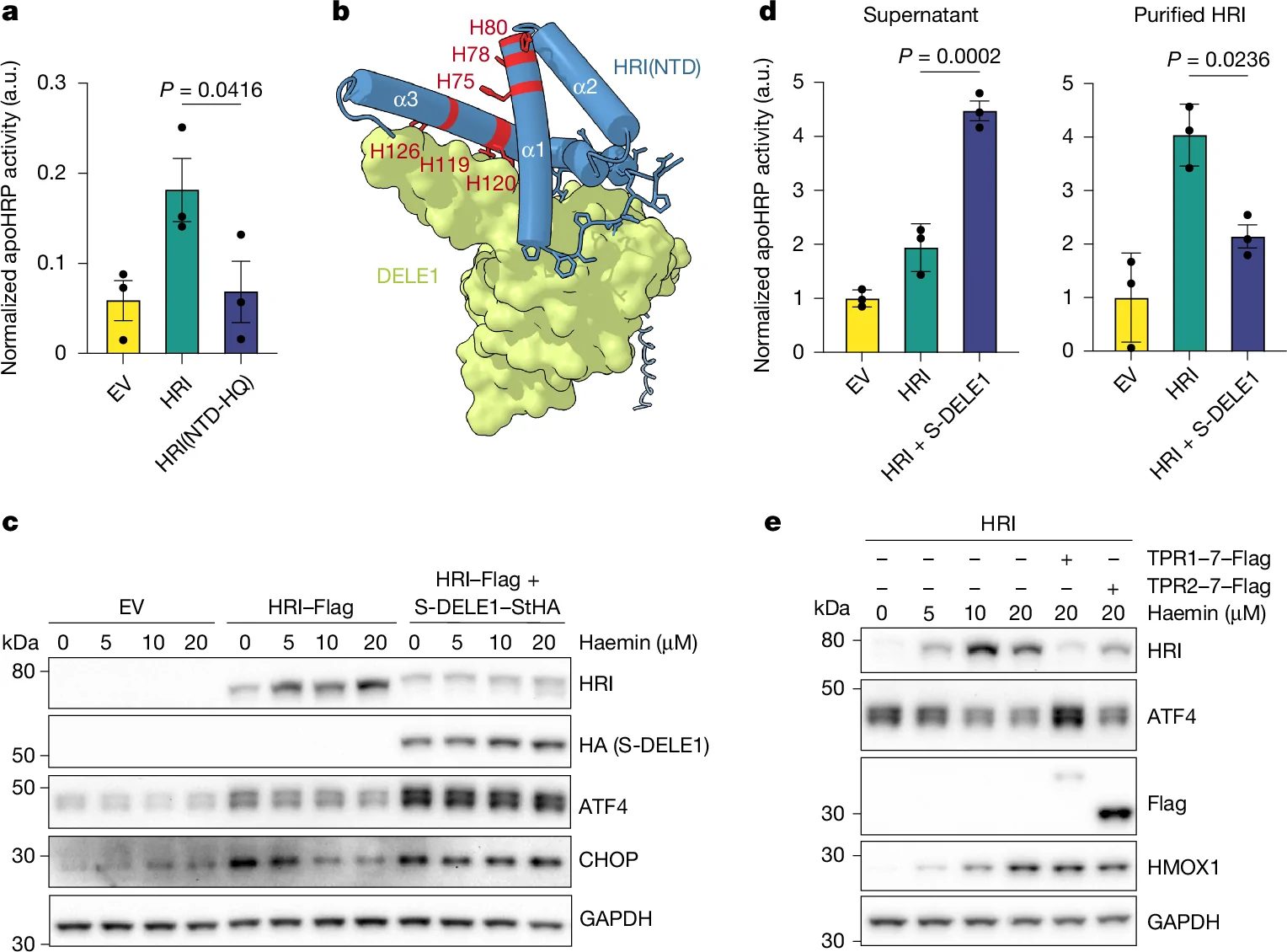
DELE1 releases inhibitory haem molecules from HRI. **a**, Haem binding to an HRI variant containing histidine-to-glutamine substitutions in the NTD (NTD-HQ), purified from 293T *EIF2S1*^S49A,S52A^ Δ*HRI* cells with endogenously edited non-phosphorylatable eIF2α(S49A,S52A). **b**, AlphaFold prediction of the DELE1–HRI(NTD) interaction, with HRI(NTD) histidine residues highlighted. **c**, Activity and levels of ectopically expressed HRI in haemin-treated 293T ∆*DELE1* and ∆*HRI* (DKO) cells in presence or absence of S-DELE1–Strep-HA (S–DELE1–StHA). **d**, Haem release from purified HRI after incubation with purified S-DELE1 in vitro, assessed by apoHRP activity. **e**, Activity and levels of HRI expressed in haemin-treated DKO cells in the presence or absence of DELE1 truncations TPR1–TPR7 (TPR1–7) or TPR2–TPR7 (TPR2–7). Mean ± s.d. of *n* = 3 independent biological replicates (**a**,**d**); one-way ANOVA with Šídák’s (**a**) or Tukey’s (**d**) multiple comparisons tests.

**Extended Data Fig. 6:**
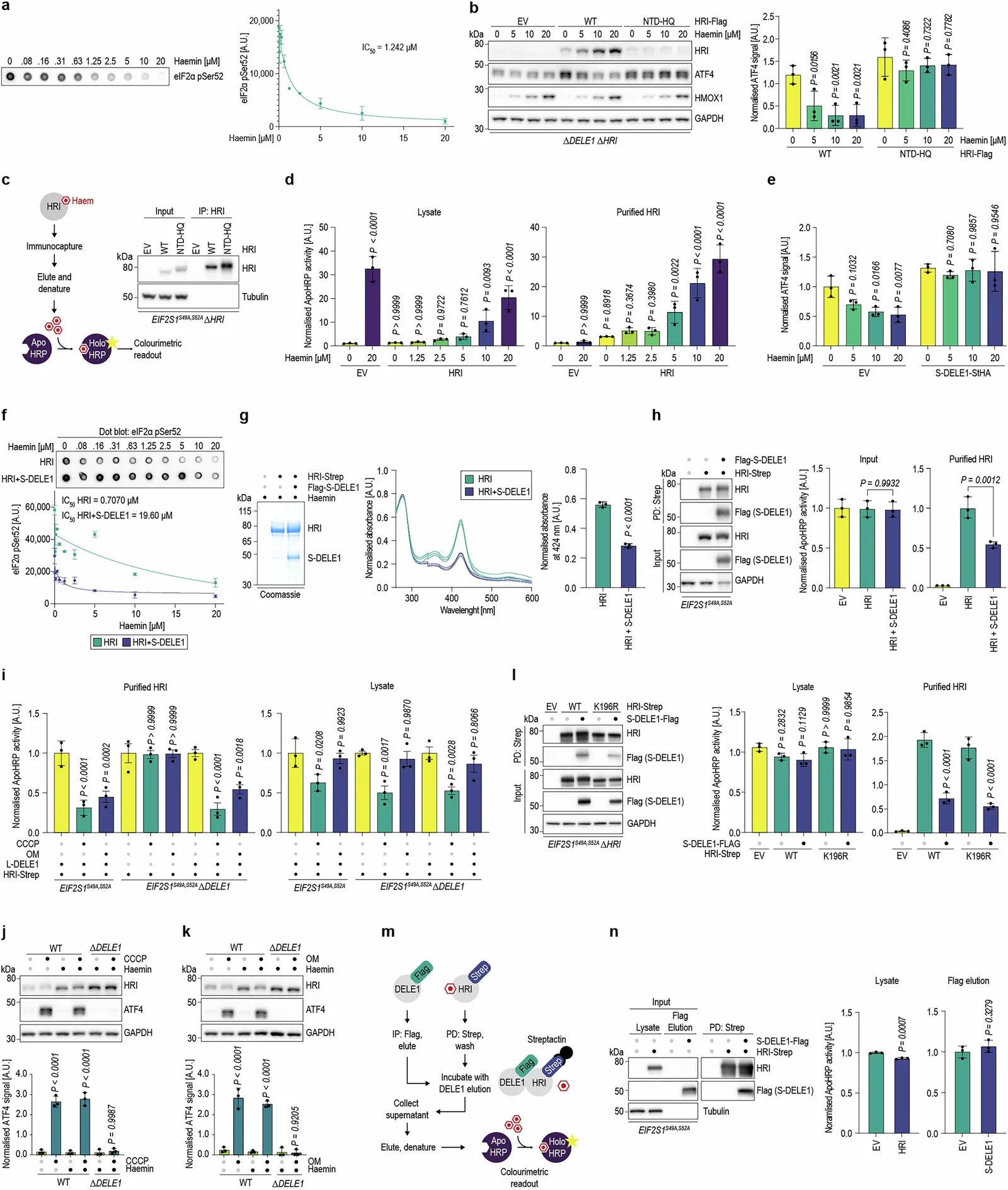
DELE1 promotes haem release from HRI. **a**, Dot blotting analysis of eIF2α phosphorylation by HRI in vitro in the presence of haemin. A non-linear regression model was used to fit the dose-response curve. **b**, Activity and levels of an HRI variant containing histidine-to-glutamine substitutions in the NTD (NTD-HQ) ectopically expressed in 293T DKO cells in presence or absence of haemin. **c**, Schematic and control immunoblot relating to Fig. 3a. **d**, Haem binding to ectopically expressed HRI purified from 293T *EIF2S1*^*S49A,S52A*^ cells in presence or absence of haemin analysed by apoHRP activity. **e**, Quantification of experiments relating to Fig. 3c. **f**, Dot blotting analysis as in (b) but in presence or absence of S-DELE1. **g**, **h**, Haem binding to HRI purified as in (d) in presence or absence of ectopic S-DELE1 analysed by UV-Vis spectroscopy (**g**) or apoHRP activity (**h**). **i**, Haem binding to HRI purified from 293T *EIF2S1*^*S49A,S52A*^ WT or *ΔDELE1* cells treated for 2 h with 10 μM CCCP or for 5 h with oligomycin A (OM) analysed by apoHRP activity. **j**, **k**, ISR signalling in 293T WT or *ΔDELE1* cells treated for 6 h with CCCP (**j**) or OM (**k**). Where indicated cells were pre-treated for 12 h with haemin. **l**, Haem binding to ectopically expressed HRI WT or K196R purified from haemin-treated 293T *EIF2S1*^*S49A,S52A*^/*ΔHRI* cells in presence or absence of S-DELE1 analysed by apoHRP activity as in (d). **m**, Workflow to assess haem release from HRI by S-DELE1 in vitro using apoHRP-based haem detection. **n**, Control immunoblot and apoHRP data relating to Fig. 3d. Mean ± s.d. of *n* = 3 independent biological replicates (**a, b, d-l, n**); two-way ANOVA with Dunnett’s multiple comparisons test (**b, e**); one-way ANOVA with Dunnett’s (**d**, **j**, **k**, **l** – lysate), Tukey’s (**h**), or Šídák’s (**i**, **l** – purified HRI) multiple comparisons tests; unpaired t-test (**n**); Welch’s t-test (**g**).

**Extended Data Fig. 7:**
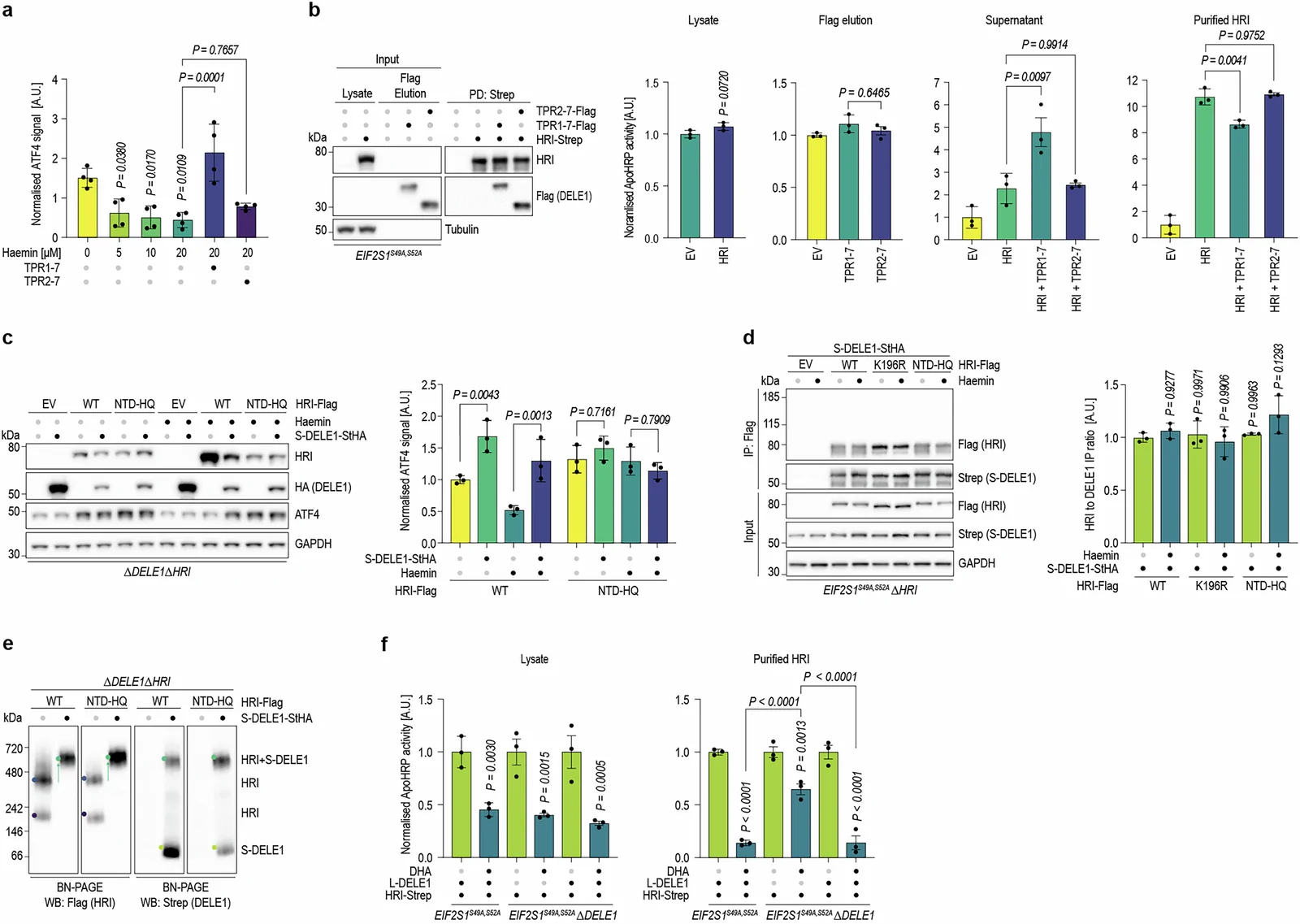
DELE1-mediated haem release promotes HRI activation. **a**, Quantification of experiments relating to Fig. 3e. **b**, Control immunoblot and apoHRP-based analysis of haem released into supernatants and retained on purified HRI following incubation with purified DELE1 truncations TPR1-7 or TPR2-7 in vitro. **c**, Activity and levels of HRI(NTD-HQ) ectopically expressed in 293T DKO cells in presence or absence of S-DELE1. Where indicated, cells were treated with haemin. **d**, Binding of S-DELE1 to the indicated HRI variants ectopically expressed in *EIF2S1*^*S49A,S52A*^/*ΔHRI* cells. Where indicated, cells were treated with haemin. **e**, BN-PAGE analysis of the indicated HRI variants in presence or absence of S-DELE1. **f**, Haem binding to HRI purified from 293T *EIF2S1*^*S49A,S52A*^ WT or *ΔDELE1* cells treated for 4 h with DHA analysed by apoHRP activity. Mean ± s.d. of *n* = 3 (**b**-**d**, **f**) or *n* = 4 (**a**) independent biological replicates; two-way ANOVA with Šídák’s (**a**) or Tukey’s (**c**) multiple comparisons test; unpaired t-test (**b** – lysate); one-way ANOVA with Tukey’s (**b** – supernatant, purified HRI), Dunnett’s (**b** – Flag elution, **d**), or Šídák’s (**f**) multiple comparisons tests.

The four human eIF2α kinases have similar kinase domains but distinct sensor domains^35^. HRI can respond to changes in cellular haemeostasis and directly bind haem^5^. The distinct nature of the HRI NTD makes it a good candidate for the sensor domain, and although different HRI haem-binding sites have been proposed, a role for NTD histidine residues marks a consensus^36–38^. Indeed, wild-type (WT) HRI accumulated to increased levels and showed reduced activity under haemin supplementation, whereas an HRI variant with mutated NTD histidines (NTD-HQ) no longer responded to haemin (Extended Data Fig. 6a,b). To more directly measure haem binding to HRI in cellulo, we combined apoHRP-based haem detection with HRI immunocapture (Extended Data Fig. 6c). This experiment confirmed that histidine substitution in the NTD severely impaired the ability of HRI to bind haem (Fig. 3a). We next explored how DELE1 promotes HRI activity in cellular haemeostasis. Our data demonstrated that the central region of the HRI NTD mediates both haem and S-DELE1 binding (Fig. 3b), which raises the possibility that S-DELE1 recruitment relieves HRI of inhibitory haem. To test this idea, we first made use of the observation that without exogenous haemin, ectopically expressed HRI is constitutively active in cells^21^. This is because physiological haem levels are insufficient to saturate the overexpressed kinase (Extended Data Fig. 6d). Although addition of haemin blocked HRI activity, thereby causing HRI accumulation, this effect was overridden by the co-expression of S-DELE1. This result suggests that S-DELE1 binding counteracts haem-mediated HRI inhibition (Fig. 3c and Extended Data Fig. 6e,f). Next, we assessed whether the presence of S-DELE1 interferes with HRI haem binding. HRI purified from cells co-expressing S-DELE1 exhibited a reduced haem content compared with HRI from control cells (Extended Data Fig. 6g,h). Likewise, treatment of cells with the oxidative phosphorylation inhibitors CCCP or oligomycin caused a DELE1-dependent reduction in HRI haem occupancy. This finding is consistent with the observation that DELE1 overrides haem-mediated HRI inhibition in response to mitochondrial dysfunction (Extended Data Fig. 6i–k). Notably, DELE1-mediated reduction of HRI haem binding did not require HRI catalytic activity (Extended Data Fig. 6l), which suggested that it may be a direct consequence of DELE1 binding. We therefore tested whether DELE1 can release haem from HRI in vitro. To this end, we incubated immobilized HRI with purified S-DELE1 and measured haem released into the supernatant and retained on HRI (Extended Data Fig. 6m). Most haem remained bound to HRI in vitro even after prolonged incubation, whereas S-DELE1 addition triggered haem dissociation from HRI (Fig. 3d and Extended Data Fig. 6n). Consistent with DELE1 TPR1 being dispensable for HRI binding but critical for activation^21^, TPR1–TPR7 but not TPR2–TPR7 released haem from HRI in vitro and overrode the inhibitory effect of haem on HRI, despite comparable binding to the kinase (Fig. 3e and Extended Data Fig. 7a,b). Finally, HRI(NTD-HQ), which is defective in binding haem, remained hyperactive in the presence of haemin and was not stimulated by S-DELE1 despite recruiting it. This result demonstrates that DELE1 becomes dispensable for HRI activity when haem binding by HRI is impaired (Extended Data Fig. 7c–e). In line with these observations, despite a strong reduction in cellular haem after DHA treatment, DELE1-deficient cells did not effectively reduce HRI haem occupancy, which was corrected by DELE1 re-expression (Extended Data Fig. 7f). Altogether, these data indicate that association of S-DELE1 with the HRI NTD during haem deficiency promotes the release of inhibitory haem that would otherwise remain largely bound to the kinase, which enables HRI activation.

## DELE1 post-translationally remodels HRI

**Fig. 4:**
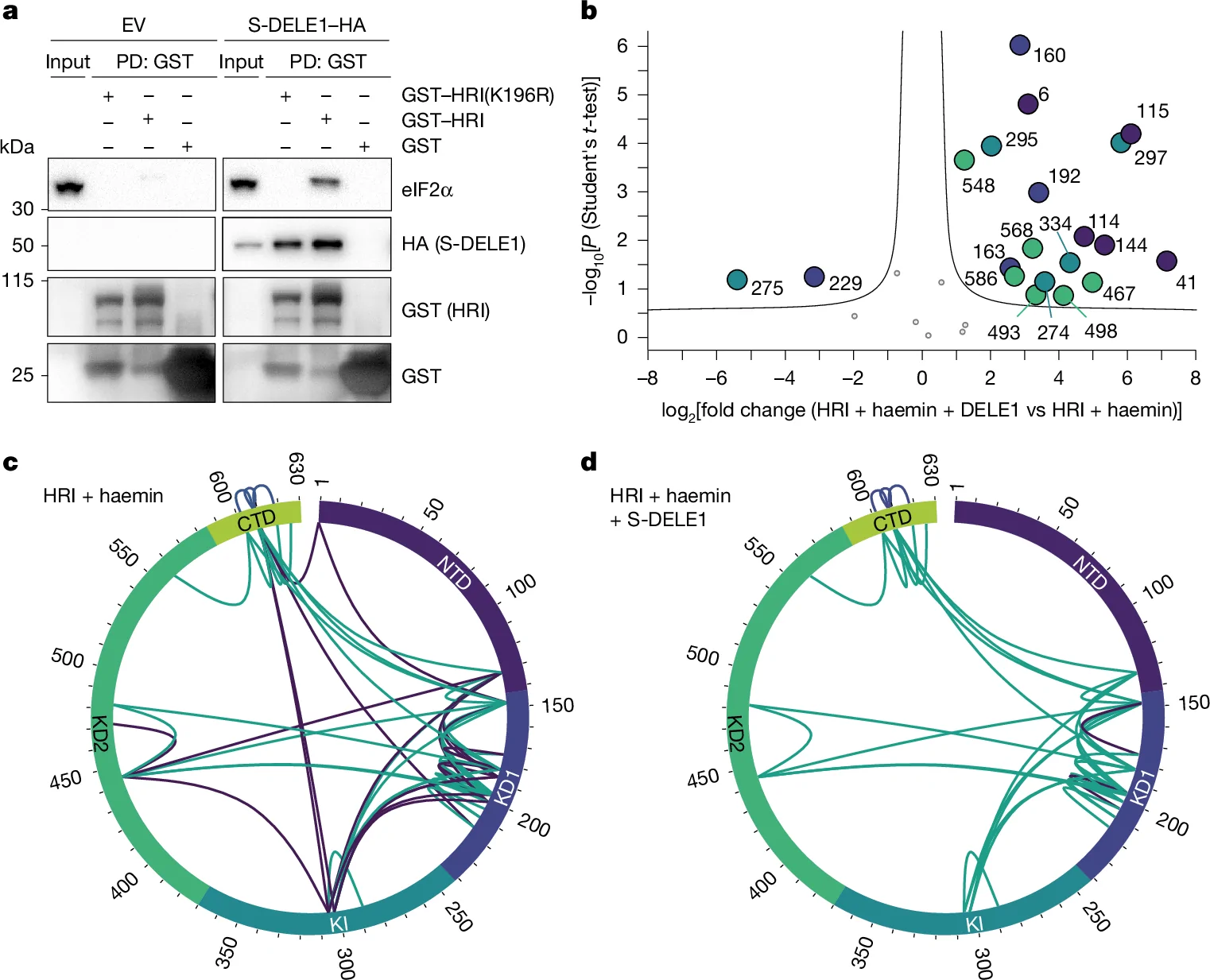
DELE1-mediated haem release promotes HRI autophosphorylation and structural maturation. **a**, In vitro recruitment of eIF2α to glutathione-*S*-transferase (GST)-tagged HRI or the catalytically inactive mutant HRI(K196R) in the presence or absence of S-DELE1 expressed in ∆*HRI* cells. PD, pull-down. **b**, HRI phosphorylation sites identified by mass spectrometry. Significant phosphorylation changes in the presence of haemin or S-DELE1 are coloured. **c**,**d**, Circos plots of HRI crosslinks identified by mass spectrometry in the presence of haemin with or without S-DELE1, observed in at least 3 out of *n* = 4 independent biological replicates. Crosslinks in purple are lost (**c**) or gained (**d**) in the presence of S-DELE1.

**Extended Data Fig. 8:**
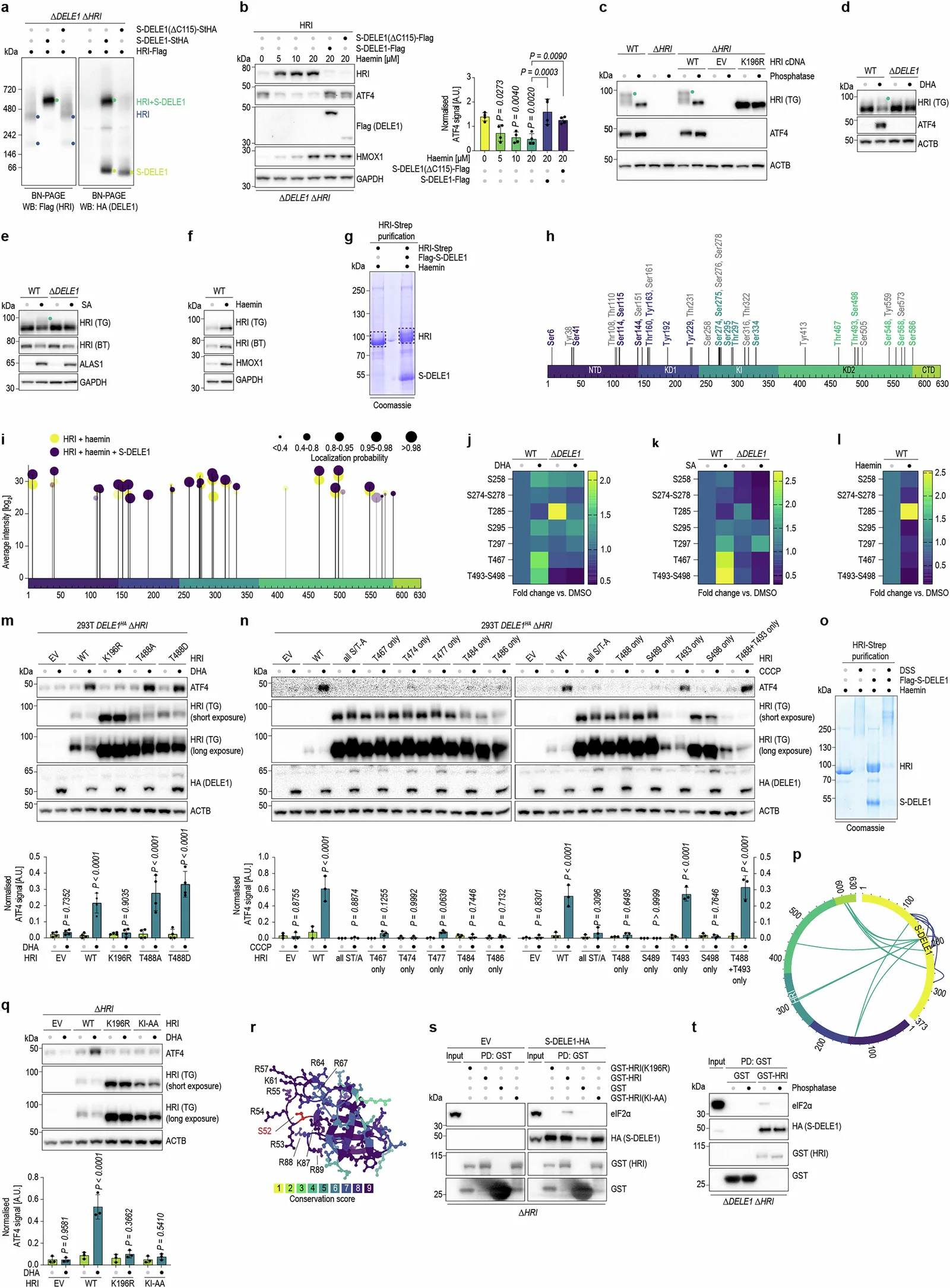
Biochemical analysis of HRI conformation and phosphorylation. **a**, BN-PAGE analysis of HRI in 293T DKO cells expressing HRI and the indicated S-DELE1 variants. **b**, Activity and levels of ectopically expressed HRI in 293T DKO cells expressing the indicated S-DELE1 variants in presence or absence of haemin. **c-f**, Analysis of HRI phosphorylation in 293T *ΔHRI* cells stably expressing the indicated HRI variants (**c**) or in 293T WT vs. *ΔDELE1* cells (**d-f**) treated with CCCP for 4 h (**c**), 10 µM DHA for 4 h (**d**), SA for 36 h (**e**), or haemin for 16 h (**f**). Dot indicates hyperphosphorylated HRI; 7.5% Tris-Glycine PAGE (TG); 4-12% Bis-Tris PAGE (BT). **g**, Denaturing-PAGE analysis of inactive and active HRI molecules (dashed boxes) purified from 293T *EIF2S1*^*S49A,S52A*^ cells for mass spectrometry (MS) analysis to assess HRI phosphorylation. **h**, **i**, HRI phosphorylation sites identified by MS. Coloured sites indicate significantly changed phosphorylation in presence or absence of S-DELE1 (**h**). Lollipop plot shows the average phosphorylation intensity and localization probability (**i**). **j-l**, MS analysis of phosphorylation of endogenous HRI purified from 293T *EIF2S1*^*S49A,S52A*^ WT or *ΔDELE1* cells treated for 6 h with DHA (**j**), 36 h with SA (**k**), or 16 h with haemin (**l**). **m**, **n**, ISR signalling in 293T *DELE1*^*HA*^ *∆HRI* cells stably expressing the indicated HRI variants and treated for 6 h with DHA (**m**) or 10 µM CCCP (**n**). **o**, Denaturing-PAGE analysis of HRI purified from 293T *EIF2S1*^*S49A,S52A*^ cells in presence or absence of S-DELE1 and cross-linked with DSS. **p**, Circos plot showing DELE1-DELE1 (blue) and DELE1-HRI (green) cross-links identified by MS. Indicated cross-links were observed in at least three of *n* = 4 independent biological replicates. **q**, Analysis of ISR signalling and HRI phosphorylation in 293T *∆HRI* cells stably expressing the indicated HRI variants and treated with DHA for 6 h; non-phosphorylatable kinase insert mutant (KI-AA). **r**, Conservation of positively charged amino acids (R, K) surrounding serine52 (red) of human eIF2α. Generated via ConSurf^64^. **s**, In vitro recruitment of eIF2α to the indicated GST-HRI variants in presence or absence of S-DELE1. **t**, In vitro recruitment of eIF2α to S-DELE1-bound HRI in presence or absence of lambda phosphatase. Mean ± s.d. of *n* = 3 (**n**, **q**) or *n* = 4 (**b**, **m**) independent biological replicates; one-way ANOVA with Šídák’s multiple comparisons test (**b**); two-way ANOVA with Tukey’s multiple comparisons test (**m**, **n**, **q**).

We next investigated how DELE1-mediated haem loss might enable HRI to attain eIF2α kinase activity. It could induce changes in HRI that promote eIF2α engagement or DELE1 itself could act as an adaptor between HRI and its substrate. S-DELE1 similarly reduced haem occupancy of both WT HRI and its catalytically inactive variant (K196R) (Extended Data Figs. 6l and 7d). However, it stimulated eIF2α recruitment only to the active kinase, which suggested that S-DELE1 does not scaffold eIF2α onto HRI (Fig. 4a). Consistently, a truncated S-DELE1 version that does not stably associate with HRI was still able to activate HRI in the presence of haemin (Extended Data Fig. 8a,b). We therefore examined DELE1-induced post-translational modifications of HRI, the active form of which is substantially autophosphorylated (Extended Data Fig. 8c). Unstressed cells displayed two main HRI species, which transitioned into a third, slowly migrating form after haem-depletion-induced DELE1 signalling (Extended Data Fig. 8d–f). Phosphoproteomics revealed multiple phosphorylation sites across the principal domains of ectopically expressed and endogenous HRI in cellulo, several of which were oppositely regulated by S-DELE1 and haem (Fig. 4b, Extended Data Fig. 8g–l and Supplementary Data 2 and 3). A recent study reported that in vitro, only phosphorylated HRI is active towards eIF2α in a manner that requires neither the NTD nor the CTD^31^. It is plausible that critical autophosphorylation would therefore target the KD activation segment^39^. For mouse HRI expressed in bacteria, autophosphorylation at T485 (human T488) is essential for activity towards eIF2α^40,41^. Our phosphoproteomic experiments identified S-DELE1-stimulated phosphorylation at T467, T493 and S498 of human HRI in cellulo. By contrast, T488 was dispensable for haem-deficiency-induced ISR signalling (Extended Data Fig. 8m). Mutagenesis of the activation segment revealed that T493 was sufficient to enable HRI activity (Extended Data Fig. 8n), and this site underwent DELE1-dependent phosphorylation after haem starvation (Extended Data Fig. 8j,k). Nevertheless, an HRI mutant lacking all serine and threonine residues in the activation segment still exhibited autophosphorylation, which suggested that additional phosphorylation events outside this region contribute to HRI activity. We noted multiple phosphorylation sites responsive to S-DELE1 and haem in the disordered KI domain (Fig. 4b and Extended Data Fig. 8g–l). HRI crosslinking mass spectrometry in the presence of haemin revealed substantial crosslinks between the KI and the CTD–KD, which suggested a compact configuration that possibly obstructs the active site (Fig. 4c, Extended Data Fig. 8o and Supplementary Data 4). By contrast, in presence of S-DELE1, crosslinks between the KI and the CTD–KD were reduced (Fig. 4d), and new crosslinks emerged between S-DELE1 and any of the principal HRI domains (Extended Data Fig. 8p). These data indicate that there are substantial, DELE1-mediated rearrangements of the KI in the HRI dimer, which could be coupled to KI phosphorylation. In support of this idea, serine and threonine substitutions in the KI eliminated slowly migrating HRI phospho-species and abrogated HRI downstream activity (Extended Data Fig. 8q). We wondered how KI phosphorylation might promote HRI activity. The HRI target region in eIF2α contains numerous conserved positively charged residues that may need to be neutralized (Extended Data Fig. 8r). Notably, unlike WT HRI, the KI serine/threonine mutant did not recruit eIF2α (Extended Data Fig. 8s), and in vitro dephosphorylation of purified HRI molecules strongly attenuated eIF2α binding (Extended Data Fig. 8t). Together, these data indicate that HRI dimers undergo substantial rearrangements after transition from a haem-bound state into an S-DELE1-bound state and attain eIF2α kinase activity in a manner that involves T493 and phosphorylatable serine and threonine residues in the disordered KI.

## An ancient haem checkpoint gates globins

**Fig. 5:**
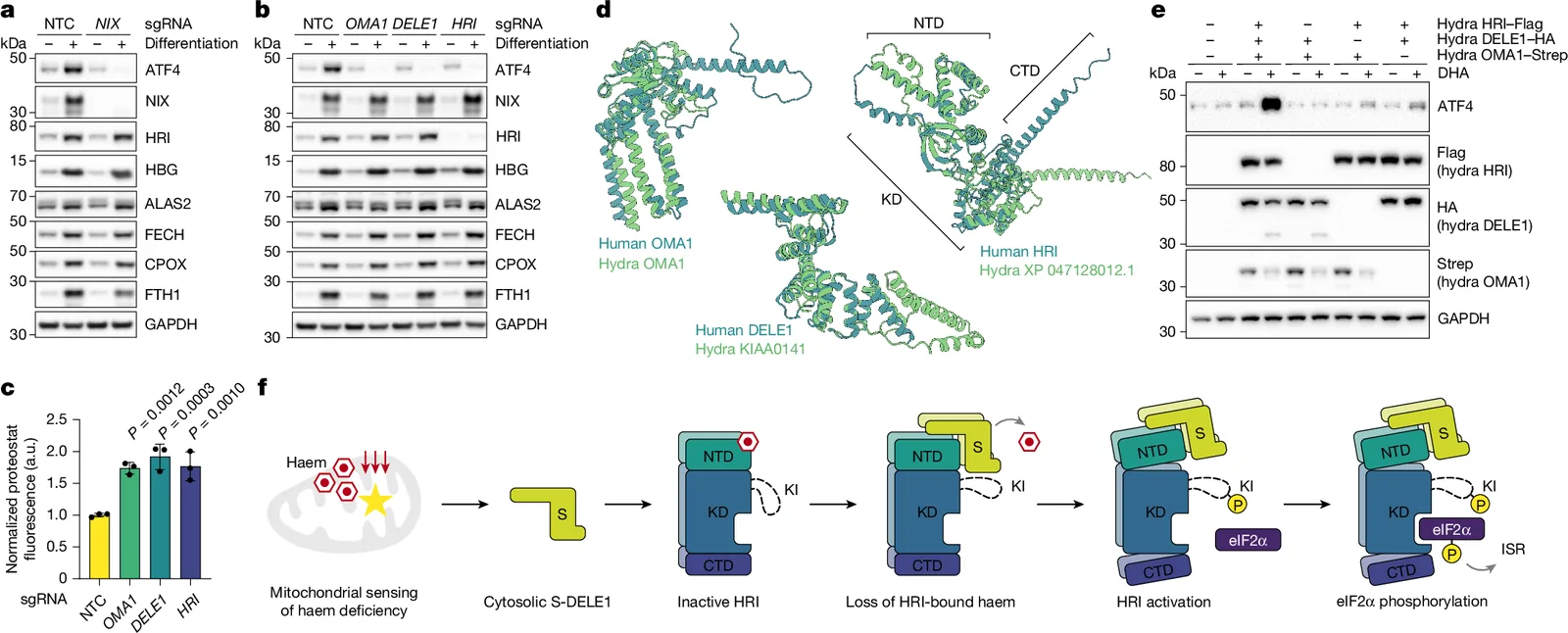
A conserved mitochondrial response to haem deficiency tunes globin biology. **a**,**b**, ISR signalling in K562 cells exposed to the indicated single-guide RNAs (sgRNAs) under differentiation for 3 days. NTC, non-targeting control. **c**, Protein aggregation in K562 cells treated as in **b** (mean ± s.d. of *n* = 3 independent biological replicates; one-way ANOVA with Dunnett’s multiple comparisons test). **d**, Superposition of AlphaFold-predicted ordered domains from human (dark green) and hydra (light green) homologues of OMA1, DELE1 and HRI. **e**, Reconstitution of the mitochondrial haem-sensing pathway using *H. vulgaris* orthologues in 293T cells lacking OMA1, DELE1 and HRI treated with DHA for 6 h. **f**, Model of human HRI activation in response to haem deficiency.

**Extended Data Fig. 9:**
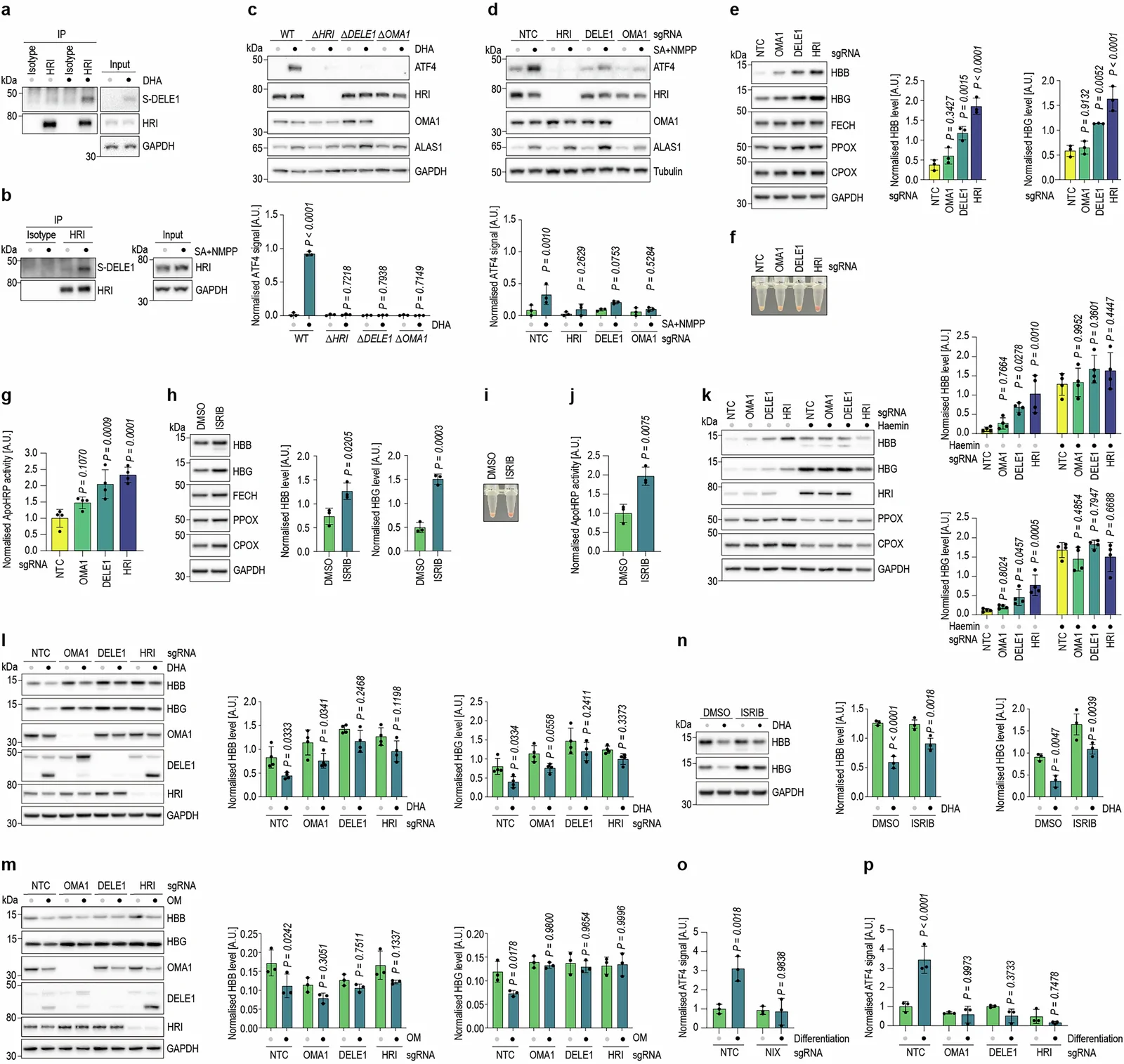
The DELE1 pathway regulates globin biology in the erythroid lineage. **a**, **b**, Analysis of DELE1 binding to HRI by co-IP using cytosolic extracts of K562 cells treated with DHA for 4 h (**a**) or SA and NMPP for 24 h (**b**). **c**, **d**, ISR signalling in K562 cells of the specified genotypes (**c**) or exposed to the indicated sgRNAs (**d**) treated as in (a) or (b). **e-g**, β-globin (HBB) and γ-globin (HBG) levels (**e**), cell colouration (**f**) and haem levels assessed by apoHRP (**g**) in K562 cells exposed to the indicated sgRNAs. **h**-**j**, Globin levels (**h**), cell colouration (**i**), and haem levels assessed by apoHRP (**j**) in K562 cells exposed to ISRIB for 5 days. **k**-**m**, Globin levels in K562 cells exposed to the indicated sgRNAs and treated for 24 h with haemin (**k**), 12 h with DHA (**l**), or 16 h with 0.1 μM OM (**m**). **n**, Globin expression in K562 cells exposed to ISRIB and treated with DHA for 16 h. **o**, **p**, Quantification of experiments relating to Fig. 5a,b. Mean ± s.d. of *n* = 3 (**c**-**e**, **h**, **j**, **m**-**p**) or *n* = 4 (**g**, **k**, **l**) independent biological replicates; two-way ANOVA with Tukey’s (**c**, **d**) or Šídák’s (**l**-**p**) multiple comparisons tests; one-way ANOVA with Dunnett’s multiple comparisons test (**e**, **g**, **k**); unpaired t-test (**h**, **j**).

Our data indicated that the cellular response to haem deficiency is a mitochondrial process that requires OMA1 and DELE1 upstream of HRI across human cells from diverse tissues. This finding made us wonder whether this mechanism extends to the erythroid lineage, in which HRI was originally proposed as an autonomous haem sensor^3^. Mammals eliminate mitochondria during erythrocyte differentiation^42^. We therefore analysed erythroid progenitors, which must balance cellular haem levels and globin synthesis rates during erythropoiesis^43^. Haem depletion in K562 cells triggered cytosolic interactions between HRI and S-DELE1 and resulted in ISR activation, which was abrogated in cells lacking OMA1, DELE1 or HRI (Extended Data Fig. 9a–d). In line with HRI-mediated ISR signalling tuning globin synthesis in the erythroid lineage, β-globin (HBB) and γ-globin (HBG) were increased in HRI-deficient K562 cells. This effect was also observed after DELE1 depletion or the use of an ISR inhibitor (ISRIB)^44^ and was largely lost under conditions of haemin supplementation that resulted in high globin levels (Extended Data Fig. 9e–k). Moreover, haem deprivation and mitochondrial impairment resulted in reductions in HBB and HBG, which was blunted in HRI-deficient cells but similarly observed after DELE1 depletion or ISRIB treatment (Extended Data Fig. 9l–n). Together, these findings indicate that in erythroid precursors, globin translation rates are tuned to haem availability through mitochondrial signals. We therefore explored the reason for this wiring logic. A characteristic step in erythroid maturation is the elimination of mitochondria mediated by NIP3-like protein X (NIX)^42^, which ends cellular haem biosynthetic capacity. In this context, the DELE1 pathway may help align haem and globin biogenesis to limit the aggregation-associated toxicity of haem-free globin. Differentiation of K562 cells was accompanied by increased ISR activation, which was abrogated by NIX deficiency (Fig. 5a and Extended Data Fig. 9o). Notably, this effect was phenocopied by genetic disruption of the DELE1 pathway, which resulted in increased protein aggregation. This finding indicates that DELE1-dependent ISR signalling counteracts proteotoxicity in this setting (Fig. 5b,c and Extended Data Fig. 9p).

**Extended Data Fig. 10:**
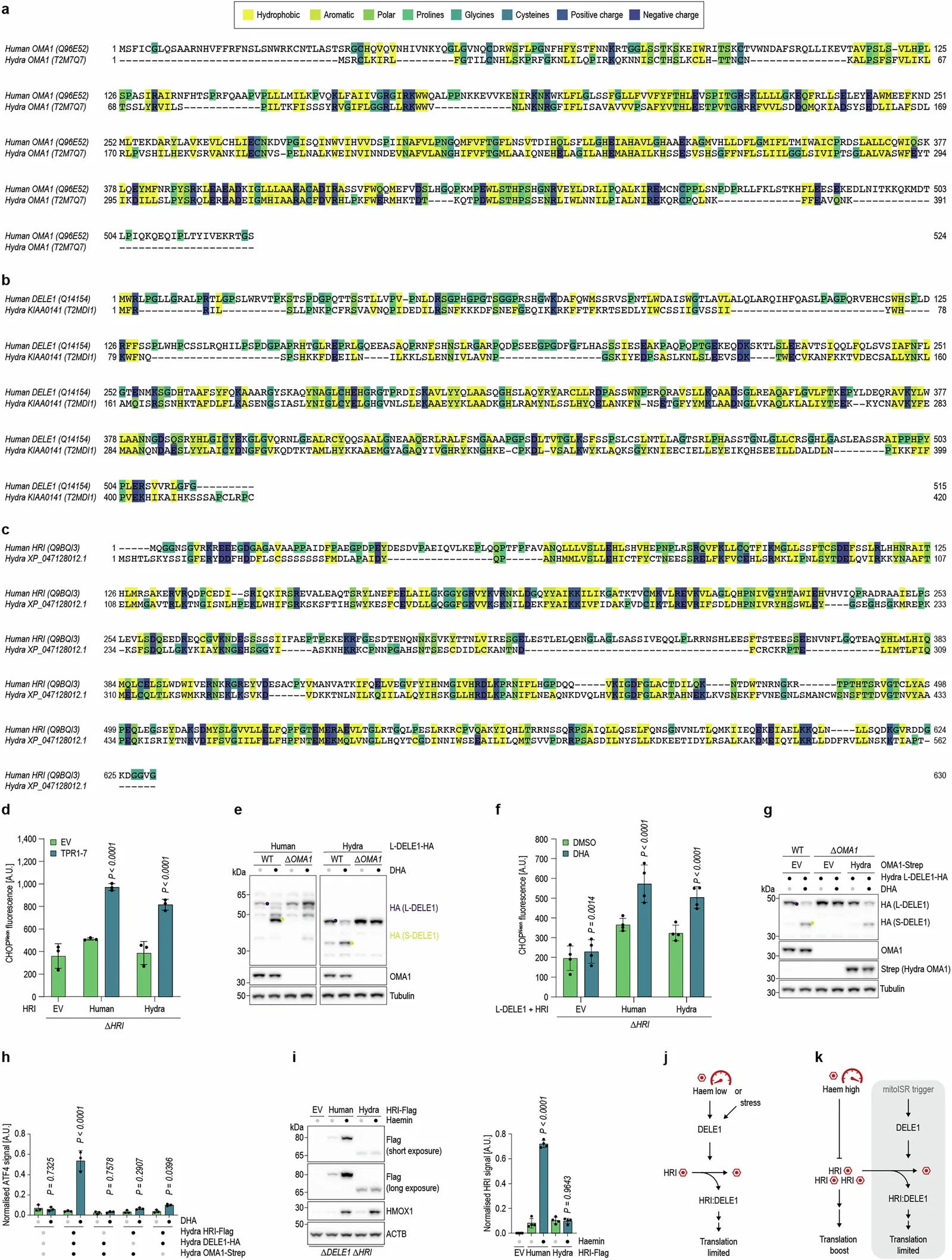
OMA1- and DELE1-dependent haem sensing is an evolutionarily ancient mitochondrial pathway. **a-c**, Amino acid sequence alignment of human and hydra OMA1 (**a**), DELE1 (**b**), and HRI (**c**) using MUSCLE^65^ (version 3.8.31). **d**, ISR signalling assessed by CHOP^Neon^ fluorescence in HAP1 *∆HRI* cells expressing human or hydra HRI and the corresponding DELE1 TPR1-7 proteins. **e**, Processing of human and hydra DELE1 stably expressed in 293T WT or *∆OMA1* cells treated with DHA for 6 h. **f**, ISR signalling as in (d) in HAP1 *∆HRI* cells expressing human or hydra HRI and L-DELE1 treated with DHA for 8 h. **g**, Processing of hydra DELE1 stably expressed in 293T WT or *∆OMA1* cells in presence or absence of hydra OMA1 and treated as in (e). **h**, Quantification of experiments relating to Fig. 5e. **i**, Analysis of HRI levels in 293T DKO cells ectopically expressing human or hydra HRI in presence or absence of haemin. **j, k**, Model for antagonistic regulation of HRI activity by DELE1 and haem in different cellular scenarios. Mean ± s.d. of *n* = 3 (**d**, **h**) or *n* = 4 (**f**, **i**) independent biological replicates; two-way ANOVA with Šídák’s (**d, f**) or Tukey’s (**h**) multiple comparisons tests; one-way ANOVA with Šídák’s multiple comparisons test (**i**).

Our results reveal a fundamental character of cellular haem sensing via the DELE1 pathway, which raises the possibility that this mechanism predates the evolutionary appearance of haemoglobin-based oxygen transport. OMA1 is conserved down to fungi, whereas DELE1 and HRI seem to be absent from the classical model organisms *Drosophila melanogaster*, *Caenorhabditis elegans* and *Saccharomyces cerevisiae*. However, structurally related^23^ proteins exist in bloodless invertebrates, including in insects and cnidaria like *Hydra vulgaris* (Fig. 5d and Extended Data Fig. 10a–c). We therefore examined the function of HRI orthologues present in species lacking haemoglobin. Like humans, these organisms use haem across various cellular processes, which will require mechanisms to monitor cellular haemeostasis. To evaluate this idea, we first tested whether the folded portion of hydra DELE1 (structurally corresponding to TPR1–TPR7 of human DELE1) could stimulate hydra HRI. We observed strong ISR activation for the hydra DELE1–HRI pair in a trans-species reconstitution experiment (Extended Data Fig. 10d). Next, we asked whether the hydra orthologues could functionally reconstitute mitochondrial haem sensing in this setting. Human OMA1 efficiently cleaved hydra DELE1 into its short form after haem deficiency to drive hydra HRI activation (Extended Data Fig. 10e,f). Finally, complete reconstitution of the hydra OMA1–DELE1–HRI axis in human cells demonstrated that the hydra pathway responds to haem deficiency and induces ISR signalling in the same manner as its human counterpart (Fig. 5e and Extended Data Fig. 10g–i). Together, our data demonstrate that the ability of mitochondria to sense and signal changes in haemeostasis through the DELE1 pathway is an evolutionarily ancient mechanism conserved beyond organisms that use haemoglobin-containing blood for oxygen transport.

## Conclusion

Here we investigated how HRI monitors cellular haem levels to synchronize translational output and haem availability. Our data revealed that although mammalian HRI can bind haem in the cytosol, haem deficiency is sensed at the level of mitochondria. Specifically, haem depletion prompts the mitochondrial protease OMA1 to cleave DELE1, which is then released from mitochondria, binds to HRI and relieves it of inhibitory haem. This process enables kinase activation that involves reconfiguration of the KI to facilitate eIF2α recruitment (Fig. 5f), which is conceptually reminiscent of PERK^32^. This haem-sensing mechanism evolutionarily predates the emergence of haemoglobins, as OMA1, DELE1 and HRI orthologues exist in bloodless invertebrates and can reconstitute the human circuit. Mitochondrial regulation of HRI-mediated haemeostasis monitoring extends to the erythroid lineage, in which the primary role of HRI is to harmonize haem availability with haemoglobin biogenesis. This process is compromised in prevalent human pathologies such as thalassaemia and sickle cell disease^45–47^.

It is interesting to speculate why haem deficiency is sensed in mitochondria. Direct cytosolic haem sensing by HRI is probably insufficient to capture the broad range of haem concentrations that exist across tissues or developmental stages. By contrast, mitochondria, which house crucial haem-dependent proteins^7^, offer a functional readout. That is, if haem levels meet the requirements for normal mitochondrial function, HRI remains inactive, whereas alterations in haemeostasis that compromise mitochondrial activity trigger a translational shutdown (Extended Data Fig. 10j). This wiring logic not only couples the haem generator, sensor and initial actuator but may also confer benefits beyond translational control by co-engaging programs that remodel mitochondrial metabolism. It also allows translation tuning through additional inputs, including NIX-mediated mitochondrial depolarization^42^. In this context, mitochondrial ISR signalling may limit globin accumulation in the proteotoxic haem-free state, which mirrors observations reported for HRI in mice subjected to iron-deficient diets^3^. This raises the question of the purpose of direct haem binding by human HRI. Although *H. vulgaris* HRI did not directly respond to haemin in human cells (Extended Data Fig. 10i), orthologues of OMA1, DELE1 and HRI from this invertebrate were able to reconstitute the mitochondrial haem-sensing circuit. This result suggests that HRI haem sensing may have emerged in organisms that use haemoglobin for oxygen transport. Given the toxicity of haem–globin imbalances^3,48^, this feature may enable boosted translation when mitochondria are energized and have high haem output, with DELE1 available as a circuit breaker after a qualifying trigger (Extended Data Fig. 10k). At late stages of erythroid differentiation, direct haem responsiveness of HRI may assume primary regulatory importance. Together, compartmentalized HRI regulation probably provides an evolutionary advantage by enabling the same molecular machinery to faithfully monitor cellular haem status and mitochondrial health across tissues with different haem content.

Artemisinins are front-line treatments for malaria, a disease that affects about 1 in 30 people^49^. Despite extensive research, their mechanism of action remains incompletely understood but may be key to combating the rise of artemisinin-resistant malaria parasites^50^. Our finding that DHA activates the ISR via mitochondrial signals offers new avenues to explore how artemisinins stunt parasite growth. In contrast to malaria, which induces anaemia through parasite-driven haemolysis, in highly prevalent congenital globinopathies like thalassaemia and sickle cell disease, it results from perturbed globin states. For these conditions, upregulation of fetal globin ameliorates pathology and is pursued as a treatment strategy^51^. The ancient haemeostasis circuit decoded here may help chart a course for selective HRI activity modulation by targeting its interactions with DELE1 and haem.

## Methods

### Cell lines and culture

HAP1 cells were cultured in Iscove’s modified Dulbecco’s medium (IMDM) (Thermo Fisher Scientific) supplemented with 10% heat-inactivated fetal calf serum (FCS) (BioSell) and 1% penicillin–streptomycin–glutamine (PSG) solution (Thermo Fisher Scientific). 293T (HEK293T), A549, HeLa, U2OS, BJEH and HCT116 cells were maintained in Dulbecco’s modified Eagle’s medium (DMEM) (Thermo Fisher Scientific) supplemented with 10% heat-inactivated FCS and 1% PSG. SH-SY5Y cells were maintained in DMEM supplemented with 15% FCS, 1% PSG and 1 mM sodium pyruvate. K562 cells were maintained in Roswell Park Memorial Institute 1640 (RPMI) medium (Thermo Fisher) supplemented with 10% heat-inactivated FCS and 1% PSG. K562 differentiation was induced as previously described^52^. In brief, cells were pretreated with 200 nM imatinib (MedChemExpress, HY-15463) in IMDM at a density of 200,000 cells per ml for 24 h followed by treatment with 1 µM decatibine (MedChemExpress, HY-A0004R) in HEMA medium (IMDM, 20% FCS, 1% PSG and 2% BSA (Sigma Aldrich, A7030-50G)), 0.5 mg ml^–1^ holo-transferrin (Merck-Sigma, T0665), 2 U ml^–1^ erythropoietin (MedChemExpress, HY-P7164) and 20 ng ml^–1^ insulin (MedChemExpress, HY-P0035) for 3 days. HAP1 *CHOP*^*Neon*^ WT and ∆*HRI* cells and clonal 293T ∆*OMA1*, ∆*DELE1*, ∆*HRI* and *DELE1*^*HA*^ cells have been previously described^21^. All cell lines were tested initially for mycoplasma contamination.

### Gene editing

To mutate *EIF2S1* (which encodes eIF2α) endogenously into a non-phosphorylatable variant (*EIF2S1*^S49A,S52A^), 293T cells were transfected with a pX330 CRISPR plasmid (Addgene, 42230) containing a sgRNA targeting *EIF2S1* exon 2, a donor vector encoding the serine 49 and 52 to alanine mutation and around 700 bp homology arms upstream and downstream of the sgRNA target site, and a puromycin-resistance vector. After 24 h of transfection, cells were selected with puromycin (1 μg ml^–1^), and single cell clones were derived and analysed for gene editing by PCR and Sanger sequencing.

Clonal and polyclonal knockout cell lines were also generated using the CRISPR–Cas9 system. Specifically, transient transfection of a pX330 containing the sgRNA of interest together with a puromycin-resistance or blasticidin-resistance vector was used to generate clonal 293T cells lacking *OMA1*, *DELE1*, *HRI*, *HMOX1* and/or *HMOX2*. Lentiviral transduction of a pLentiCRISPR v.2 variant (derived from Addgene, 52961) containing the sgRNA of interest was used to delete *OMA1*, *DELE1*, *HRI* or *NIX* in A549, HeLa, U2OS, BJEH, HCT116, SH-SY5Y or K562 cells. After 24 h of transduction or transfection, cells were selected using puromycin (1 μg ml^–1^) or blasticidin (10 μg ml^–1^), and knockout efficiency was assessed by immunoblotting. Where indicated, clonal progeny of the polyclonal knockout populations were generated by single-cell cloning and verified by PCR, Sanger sequencing and immunoblotting. sgRNA sequences and primers for genotyping PCR used in this study are listed in Supplementary Table 1.

### Haploid genetic screen for identification of CHOP regulators

Genome-wide mutagenesis of haploid HAP1 cells was carried out as previously described^21^. In brief, gene-trap virus particles were produced in 293T cells, concentrated by ultracentrifugation at 22,800 rpm for 2 h at 4 °C and stored at 4 °C overnight. To generate random genomic mutations via insertional mutagenesis by gene trapping, 1.5 × 10^7^ haploid HAP1 *CHOP*^*Neon*^ cells were transduced with concentrated retroviral particles 24 h after plating, followed by two additional transductions. The resulting library of mutants was expanded, plated at 20% confluence in a total of 20 T175 flasks (Sarstedt) and treated for 9 h with 5 μM DHA 48 h after plating. Cells were collected using trypsin–EDTA (0.25%, Gibco), passed through a 40 μm cell strainer (Greiner, 542040) and fixed with one volume of BD fix buffer I (BD Biosciences) for 10 min at 37 °C. The fixation was stopped with PBS (Gibco) containing 1% FCS, cells were passed through a 40 μm cell strainer, and approximately 1.5 × 10^9^ cells were permeabilized with 1 pellet volume of cold BD Perm Buffer III (BD Biosciences) for 30 min on ice. Permeabilization was stopped with PBS containing 1% FCS, and cells were blocked in PBS with 1% FCS and 3% bovine serum albumin (BSA) for 30 min at room temperature. To increase fluorescence intensity of the CHOP(Neon) protein, cells were stained with anti-mNeonGreen antibodies (ProteinTech, 32F6) diluted 1:2,500 in PBS with 1% FCS and 1% BSA for 2.5 h on a rotor wheel at room temperature, followed by three 15-min washing steps with PBS and 1% FCS at room temperature. Primary antibodies were detected using AlexaFluor 488-conjugated secondary antibodies (anti-mouse-AF488, Life Technologies) diluted 1:500 in PBS supplemented with 1% FCS and 1% BSA for 1 h at room temperature on a rotor wheel protected from light. DAPI (Sigma-Aldrich, D9542) was added to the secondary antibody dilution at a final concentration of 2.5 μg ml^–1^ for a DNA counterstain. After three 15-min washing steps, cells were resuspended in PBS and 1% FCS, stored at 4 °C until sorting on a BD Fusion cell sorter (BD Biosciences, via FACSDiva v.8.0.2) using a 70 μm nozzle. Staining specificity was determined using a secondary antibody-only control. Haploid cells were identified on the basis of DNA content in the DAPI channel and of those, approximately 10^7^ cells of the bottom 4% CHOP(Neon)-low and top 4% CHOP(Neon)-high cells were sorted into PBS and 10% FCS for isolation of gDNA.

### Insertion site mapping and analysis

To extract gDNA from the sorted cell populations de-crosslinking was performed at 56 °C overnight followed by DNA isolation using a QIAamp DNA Mini kit (Qiagen, 51306) according to the manufacturer’s instructions. Gene-trap insertion sites of CHOP(Neon)-high and CHOP(Neon)-low populations were recovered as previously described^21^. The amplified libraries were sequenced on a NextSeq1000 (Illumina) with a read length of 60 nucleotides. Demultiplexing of indexed sequencing reactions was performed, allowing one mismatch. Reads were aligned to the human reference genome (hg19) and analysed as previously described^21^. Bowtie^53^ (v.1.0.1) was used to align reads to the human genome, allowing one mismatch, followed by mapping to the coordinates of RefSeq protein-coding genes with intersectBED^54^ (v.2.26.0). Only integrations in the sense orientation were considered disruptive and used for downstream analyses. To identify CHOP(Neon) regulators in DHA-treated cells per gene, the number of unique gene-trap insertion sites in the query gene versus the whole sample was compared between the CHOP(Neon)-high and CHOP(Neon)-low cell populations using a two-sided Fisher’s exact test and Benjamini–Hochberg FDR correction. Data were plotted as the combined number of unique mutations identified in the CHOP(Neon)-high and CHOP(Neon)-low population (*x* axis) versus their mutation ratio (high versus low) normalized to the respective sizes of the datasets (*y* axis). Fishtail plots were created using GraphPad Prism 10.

### Treatments, transfections and transductions

Unless otherwise stated, cells were treated with 20 μM CCCP (Sigma-Aldrich, C2759), 1 μM oligomycin A (Sigma-Aldrich, 75351), 10 μM antimycin A (Sigma-Aldrich, A8674), 10 μM tunicamycin (MedChemExpress, HY-A0098), 5 μM DHA (MedChemExpress, HY-N0176), 100 μM SA (Sigma-Aldrich D1415), 20 μM NMPP (CaymanChemical, Cay20846-5), 250 nM ISRIB (Sigma-Aldrich SML0843), 20 μM haemin (Sigma-Aldrich, 51280), 5 mM GSH (Merck-Sigma, G4251), 10 mM NAC (Sigma-Aldrich, A9165), 100 μM FeCl_2_ (Sigma-Aldrich 372870) or 100 μM DFO (Sigma-Aldrich, D9533) for the indicated times. To induce robust haem starvation with SA, NMPP or the combination, media were supplemented with 5% haem-depleted FCS instead of normal FCS. To this end, heat-inactivated FCS was incubated with 10 mM ascorbic acid for 8 h at 37 °C and dialysed 3 times against PBS. Haemin stock solutions were prepared according to a previously described method^55^, with the exception that ethylene glycol was used as a solvent. To prepare a 5 mM haemin stock solution, 32.6 mg haemin chloride was dissolved in 500 μl 1 M NaOH, 500 μl 0.5 M Tris and 8.4 ml ethylene glycol. Finally, the pH was adjusted to pH 7.4 by the addition of 600 µl 1 M HCl and the solution was stored at −20 °C. A solution lacking haemin chloride was used as the control. For all haem rescue experiments, haemin was added 2 h (for SA or NMPP) or 12 h (for DHA) before haem-starvation induction. For induction of shRNAs from the pLKO vector, cells were treated with 500 ng ml^–1^ doxycycline hyclate (Biomol, Cay14422-1) for 3 days. shRNA sequences used in this study are listed in Supplementary Table 1.

Where indicated, cells were transfected using polyethylenimine (PEI 25000, Polysciences) or Turbofectin (OriGene Technologies). PEI was used to transfect 293T cells at a PEI-to-DNA ratio of 3:1. Turbofectin was used to transfect HAP1 cells at a Turbofectin-to-DNA ratio of 2.5:1. Transfection reagents and DNA dilutions were prepared separately in OptiMEM (Gibco), incubated for 10 min at room temperature, mixed by pipetting and added to 50% confluent cells after an additional 20 min of incubation. For activity analysis of ectopically expressed HRI in presence or absence of haemin, 293T *DELE1* and *HRI* double-knockout cells were grown in 24-well plates and transfected with 100 ng HRI and 400 ng DELE1 or empty-vector plasmid DNA. After 4 h of transfection, cells were treated with the indicated haemin concentration and cells were collected 24 h after transfection. For HRI and/or DELE1 purifications, 293T *EIF2S1*^S49A,S52A^ WT or *HRI* knockout cells were grown on 15 cm cell culture dishes, transfected with 30 μg plasmid DNA, treated with haemin 24 h after transfection and collected after an additional 24 h of culture.

The generation of lentiviral and retroviral particles was performed as previously described^21^. In brief, 293T cells were transfected with lentiviral (pCMVd8.2dVPR, pCMV-VSV-G, pAdVAntage) or retroviral (pCMV-Gag-Pol, pCMV-VSV-G, pAdVAntage) packaging plasmids and the indicated transfer vector encoding the cDNA or Cas9 and sgRNA of interest. After 48 h of transfection, virus particles were collected, filtered through a 0.45 μm syringe filter (Sarstedt), and the indicated cell line was transduced with 1:2 diluted virus supernatant containing protamine sulfate. After 24 h of transduction, cells were selected for at least 48 h with puromycin (1 μg ml^–1^), blasticidin (10 μg ml^–1^) or hygromycin (300 μg ml^–1^), followed by a 24-h recovery period in antibiotic-free medium.

### DNA cloning

The coding sequences of genes expressed in this study were amplified from human cDNA or synthesized as codon-optimized gene blocks (IDT) listed in Supplementary Table 2. Point mutations were introduced by overlap-extension PCR or by gene synthesis. All oligonucleotide sequences are listed in Supplementary Table 1. Amplified DNA was subjected to restriction digest and ligation using standard cloning procedures. All cloned constructs were sequence-verified by Sanger sequencing.

### Gel electrophoresis and immunoblotting

For analysis of protein expression by denaturing gel-electrophoresis, cells were treated as described in the figure legends, washed with PBS and lysed with SDS-sample buffer (60 mM Tris pH 6.8, 2% SDS, 10% glycerol, 0.01% bromophenol blue and 4% β-mercaptoethanol). For phosphatase treatment of protein lysates, cells were lysed in DISC buffer (30 mM Tris-HCl, pH 7.5, 150 mM NaCl, 10% glycerol) supplemented with protease inhibitor (complete protease inhibitor cocktail, Roche, 11697498001) and 1% NP-40 for 15 min on ice, and lysates were cleared twice by centrifugation at 20,000*g* for 10 min at 4 °C. Supernatants were transferred to a microfuge tube, and FastAP buffer and FastAP thermosensitive alkaline phosphatase (Thermo Fisher Scientific, EF0654) were added. Samples were incubated at 37 °C for 1 h, and the reaction was stopped by the addition of SDS sample buffer. All samples were denatured for 10 min at 95 °C. Subsequently, equal amounts of protein were subjected to denaturing SDS–PAGE and transferred to PVDF membrane (Millipore) by semi-dry transfer using a Bolt gel electrophoresis and transfer system (Thermo Fisher Scientific) according to the manufacturer’s instructions and homemade transfer buffer (190 mM glycine, 25 mM Tris and 20% ethanol). Bolt gradient gels (4–12%; Thermo Fisher) and a MOPS or MES buffer system (Thermo Fisher) were used for routine immunoblot analyses. For analysis of HRI phosphorylation, proteins were separated using 7.5% Tris–glycine gels and a Tris buffer system (25 mM Tris, 192 mM glycine and 0.1% SDS). After transfer, membranes were blocked by incubation with Tris-buffered saline (25 mM Tris-HCl, pH 7.6, and 150 mM NaCl), containing 0.02% Tween 20 (TBST) and 5% dry milk (Sigma Aldrich) for 1 h at room temperature. After incubation with primary antibodies for 2 h at room temperature or at 4 °C overnight, membranes were washed three times with TBST and incubated with secondary antibodies (goat-anti-rabbit/mouse HRP-conjugated, diluted in TBST + 5% dry milk, Bio-Rad) for 1 h at room temperature. Membranes were washed three times with TBST, enhanced chemiluminescence solution^56^ was applied and the signal was detected using a Bio-Rad Chemidoc MP system. The antibodies used for immunoblotting are listed in Supplementary Table 3.

To analyse DELE1-containing and HRI-containing protein assemblies in cytosolic extracts by native gel electrophoresis, cells were seeded and treated as described in the figure legends, washed with PBS and lysed for 20 min on ice with NativePAGE sample buffer (Thermo Fisher) supplemented with protease inhibitors and 0.02% digitonin (Sigma-Aldrich, D141). Subsequently, cytosolic extracts were cleared twice by centrifugation at 20,000*g* for 10 min at 4 °C, NativePAGE G-250 sample additive was added to a final concentration of 0.025% (v/v) and samples were separated on 3–12% Bis-Tris Mini protein gels (Invitrogen) according to the manufacturer’s protocol using NativePAGE cathode and anode buffers (Thermo Fisher). Native mark (Fisher scientific) was used to estimate the molecular weight of protein assemblies. Subsequently, proteins were transferred to PVDF membrane by semi-dry transfer using NuPAGE transfer buffer (Invitrogen) and a Bolt gel electrophoresis and transfer system (Thermo Fischer) according to the manufacturer’s protocol. Protein assemblies were detected by immunoblotting as described above.

### Isolation and fractionation of mitochondrial membranes

To determine haem levels in the cytosol and different mitochondrial subfractions, 293T WT cells were treated with 5 μM DHA for 6 h. Afterwards, cells were collected by scraping, washed with PBS and cell pellets were collected by centrifugation at 450*g* for 5 min. Cells were resuspended with homogenization buffer (20 mM HEPES-KOH pH 7, 220 mM mannitol, 70 mM sucrose and 20 μM EGTA) and lysed on ice by passing the cell suspension through a 25 G needle (0.50 × 16 mm, Sterican, B. Braun) attached to a 1 ml syringe (Omnifix-F Luer, B. Braun) in 3 cycles of 10 passes each, with cooling intervals between cycles. Cell debris was removed by centrifugation twice at 700*g* for 5 min at 4 °C, and supernatant was transferred into new tubes and centrifuged at 8,000*g* for 15 min at 4 °C to pellet the crude mitochondrial fraction. Cytosolic fractions were transferred into new tubes and centrifuged again at 20,000*g* for 5 min at 4 °C to remove residual mitochondria. Mitochondrial pellets were resuspended in EM buffer (10 mM HEPES-KOH, pH 7.4, and 1 mM EDTA–KOH pH 8.0) followed by lysis with SEM buffer (250 mM sucrose, 10 mM HEPES-KOH, pH 7.4, and 1 mM EDTA–KOH pH 8.0) containing 0.1% digitonin for 20 min on ice to release outer mitochondrial membrane and intermembrane space fractions into the solution. Subsequently, the inner mitochondrial membrane and matrix were re-isolated by centrifugation at 10,000*g* for 10 min at 4 °C. The pellets were resuspended with SEM buffer containing 1% Triton X-100, incubated on ice for 20 min and labelled as inner mitochondrial membrane together with mitochondrial matrix fractions.

### Haem measurements

Commercial apoHRP, APO/HRP4C-peroxidase (BBI Solutions, APO/HRP4C), was used in all enzyme-based haem detection assays. To deplete residual haem from apoHRP present in the preparation, apoHRP was dissolved to 4 mg ml^–1^ in PBS and haem was extracted by the addition of 5 ml acetone and 125 μl concentrated hydrochloric acid per mg apoHRP. Acetone-extracted apoHRP was recovered by centrifugation at 2,000*g* for 2 min at room temperature, dissolved to a final concentration of 50 μM in PBS and stored at −20 °C.

To measure cellular haem levels, cells were treated as indicated and lysed with PBS or DISC buffer containing protease inhibitor and 1% Triton-X100 as described above. Protein concentrations were determined by Bradford assay and adjusted to equalize protein concentrations across all samples. Subsequently, apoHRP solution was added to a final concentration of 12.5 μM followed by incubation for 10 min on ice. To detect HRP-bound haem, TMB ELISA substrate (Serva, 37068.01) was added to the reaction, and absorbance at 562 nm was measured on a microplate reader (Tecan Spark, via SparkControl, v.3.1) after incubation at room temperature in the dark for 10 min. Haem binding to purified, endogenous, stably or transiently expressed HRI was assessed analogously, with the exception that biotin-eluted or pH-shift-eluted HRI was denatured for 15 min at 98 °C before incubation with 12.5 μM apoHRP. For analysis of haem release from HRI after treatment of cells with CCCP, oligomycin A or DHA, Strep-tagged HRI, stably expressed in 293T *EIF2S1*^S49A,S52A^ WT or *DELE1* knockout cells in presence or absence of stable L-DELE1 co-expression, was used as a handle for affinity capture.

### Soret-band measurements

For analysis of haem binding to HRI by UV–vis spectroscopy, the absorbance of HRI purified in the presence or absence of S-DELE1 was recorded from 250 nm to 600 nm using a V630 Bio spectrophotometer (Jasco) equipped with a quartz cuvette (Hellma Analytics, 10 mm path length, 105-202-15-40) and Jasco Spectra Manager II software. The UV–vis spectrum was blanked against elution buffer and curves were normalized to the protein concentration calculated based on the absorbance at 280 nm using extinction coefficients of 116,180 M^−1 ^cm^−1^ for the HRI dimer and 190,340 M^−1 ^cm^−1^ for the HRI–DELE1 tetramer. Equal protein loading was subsequently confirmed by Coomassie gel analysis.

### Immunoprecipitation and affinity purifications

For purification of HRI from cytosolic extracts, cells were treated as indicated, collected by scraping in ice-cold PBS, washed and lysed in DISC buffer supplemented with protease inhibitor, phosphatase inhibitor (Thermo Fisher, A32957) and 0.02% digitonin for 20 min on ice. Cytosolic extracts were cleared by centrifugation twice at 20,000*g* for 10 min at 4 °C. Subsequently, cleared cytosolic extracts were incubated with either Strep-Tactin Sepharose resin (IBA, 2-1201-010), anti-Flag M2 magnetic beads (Sigma, M8823) or GFP-Trap magnetic agarose (Chromotek, gtma-20) for precipitation of StrepTagII-containing, Flag-containing or GFP-containing protein complexes, respectively. Immunoprecipitations of endogenous HRI were performed with anti-eIF2AK1 antibodies (Proteintech, 20499-1-AP) coupled to Pierce protein A beads (Thermo Fisher, 20333) at an antibody-to-cell lysate ratio of 1:1,000. All beads were washed five times with detergent free DISC buffer before addition of supernatants. After incubation at 4 °C on a rotor wheel for 1.5 to 3 h, beads were washed six times with DISC buffer containing 0.1% Triton-X100. After the last wash step, all washing buffer was removed, and bead bound proteins were eluted with SDS sample buffer for 10 min at 95 °C before analysis by gel electrophoresis. For sequential immunoprecipitations or haem measurements, Strep-tagged proteins were eluted with 50 mM biotin in elution buffer (50 mM NaCl, 20 mM HEPES pH 8.0, and 10% glycerol), GFP-containing protein complexes were eluted with 3C protease (Sigma-Aldrich, GE27-0843-01) in the experiments relating to sequential affinity purification and elution, and anti-Flag or anti-EIF2AK1 antibody-bound proteins were eluted with 200 mM glycine pH 2.5 followed by neutralization with Tris-HCl pH 10.5. Unless otherwise indicated in the figure, 1–1.5% of input material was loaded for immunopurification control immunoblotting.

### Medium-scale HRI and DELE1 affinity purification from mammalian cells

To purify large quantities of Strep-tagged HRI, Flag-tagged S-DELE1 or StHA-tagged S-DELE1 for in vitro analyses, 293T *EIF2S1*^S49A,S52A^ cells were transfected with the respective construct and grown in the presence or absence of 20 μM haemin as described above. After 48 h of transfection, cells were collected, washed with PBS and lysed for 30 min on ice with modified DISC buffer (20 mM HEPES, pH 7.5, 150 mM NaCl and 10% glycerol) supplemented with protease inhibitor, phosphatase inhibitor and 0.02% digitonin. Cell debris was removed by centrifugation twice at 21,000*g* for 10 min at 4 °C, and cytosolic extracts were incubated with Strep-Tactin Sepharose resin (IBA, 2-1201-010) or anti-Flag M2 magnetic beads (Sigma, M8823) for 1.5 h at 4 °C. Beads were washed six times with lysis buffer and Strep-tagged or StHA-tagged proteins were eluted with 50 mM biotin in elution buffer for haem binding, mass photometry or crosslinking experiments. For in vitro haem release assays, Strep-tagged HRI was not eluted after washing but instead incubated with pH-eluted Flag-tagged S-DELE1 or elution buffer for an additional 4 h. Subsequently, supernatant was collected, beads were washed three times with lysis buffer and HRI was eluted with biotin. For phosphorylation analysis of exogenously expressed HRI in presence or absence of S-DELE1 by mass spectrometry (MS), Strep-tagged HRI was eluted with SDS sample buffer for 10 min at 95 °C. Flag-tagged DELE1 was eluted with 200 mM glycine pH 2.5 followed by neutralization with Tris-HCl, pH 10.5.

### GST pull-down assay

GST-fused HRI or HRI mutants were expressed in *Escherichia coli* strain BL21-CodonPlus (DE3)-RIPL (Agilent Technologies) in LB medium. When the optical density (OD_600_) of bacterial cultures reached 0.2, protein expression was induced using 1 mM IPTG (Thermo Scientific, R0393) at 8 °C, 120 rpm for 48 h. Bacteria were pelleted by centrifugation (4 °C, 6,000*g*, 10 min). Bacteria were lysed by sonication on ice in lysis buffer (30 mM Tris-HCl pH 7.5, 500 mM NaCl, 10% glycerol and 0.5 mM DTT) supplemented with protease inhibitor and phosphatase inhibitor. Lysates was cleared by centrifugation (4 °C, 18,000*g*, 30 min) and the cleared lysate was incubated with glutathione sepharose 4B beads (Cytiva, GE17-0756-01) at 4 °C for 1 h with rolling. The beads were pelleted by (4 °C, 700*g*, 20 s) and washed 3 times with washing buffer (30 mM Tris-HCl pH 7.5, 150 mM NaCl, 10% glycerol) supplemented with 0.25 mM DTT. Beads immobilized with GST-fusion were ready to incubate with cell lysates. 293T cells were transfected with a plasmid encoding triple HA-tagged S-DELE1. Cells were pelleted by centrifugation (4 °C, 500*g*, 5 min) 2 days after transfection. Cells were lysed in DISC buffer supplemented with protease inhibitor, phosphatase inhibitor and 0.02% digitonin. Cell lysate was cleared by centrifugation (4 °C, 20,000*g*, 12 min) and the cleared lysate was incubated with beads immobilized with GST-fusion at 4 °C for 1 h. Beads were pelleted by centrifugation (4 °C, 700*g*, 20 s) and washed 5 times with washing buffer. Proteins immobilized on the beads were eluted in SDS sample buffer, heated at 95 °C for 10 min and then subjected to SDS–PAGE and immunoblotting analyses. For the dephosphorylation reaction coupled with GST pull-down assays, GST-fusion-coupled beads were incubated with lambda protein phosphatase (New England Biolabs) according to the manufacturer’s instructions. In these experiments, phosphatase inhibitor was omitted.

### Protein purification from bacteria

6×His-TEV-tagged HRI, HRI(K196R) or HRI along with S-DELE1 were expressed in the same manner for GST-fusion proteins but at 8 °C and 120 rpm for 48 h. Bacteria were pelleted by centrifugation (4 °C, 6,000*g*, 10 min). Bacteria were lysed by sonication on ice in lysis buffer (30 mM Tris-HCl pH 8.0, 500 mM NaCl, 25 mM imidazole, 10% glycerol and 0.5 mM DTT) supplemented with protease inhibitor and phosphatase inhibitor. Lysate was cleared by centrifugation (4 °C, 18,000*g*, 30 min) and then incubated with Ni-NTA resin (HisPur, 88221) at 4 °C for 1 h with rolling. The solution was loaded into gravity-flow columns. Resin was washed 3 times with washing buffer (30 mM Tris-HCl pH 8.0, 150 mM NaCl, 50 mM imidazole, 5% glycerol and 0.25 mM DTT). Immobilized proteins were eluted in elution buffer (30 mM Tris-HCl pH 8.0, 150 mM NaCl, 500 mM imidazole, 0.25 mM DTT) and concentrated using ultracentrifuge filters (Amicon). Concentrated proteins were loaded onto a superose 6 increase 10/300 GL column (Cytiva) connected to a chromatography system (ÄKTA pure) and eluted with SEC buffer (30 mM HEPES pH 7.5, 100 mM NaCl and 0.25 mM DTT). Fractions eluted in the main peak were collected and checked by SDS–PAGE for purity. Fractions of high purity (>95% judged by Coomassie brilliant blue staining) were further concentrated using ultracentrifuge filters (Amicon), then directly used for subsequent experiments or snap-frozen in liquid N_2_ and then stored at −80 °C.

6×His-MBP-GST-TEV-tagged eIF2α or eIF2α(S49A,S52A) were similarly purified. Protein was expressed at 16 °C, 120 rpm overnight. After elution from Ni-NTA resin, proteins were incubated with TEV protease (a gift from K.-P. Hopfner) to remove the tag. For size exclusion chromatography, a superdex 75 10/300 GL column (Cytiva) was used.

### In vitro kinase assay

To determine the linearity range of HRI and eIF2α kinase reactions, 0–200 nM HRI, 20 μM eIF2α and 40 μM ATP were reacted in PK buffer (50 mM Tris-HCl pH 7.5, 10 mM MgCl_2_, 0.1 mM EDTA, 2 mM DTT and 0.01% Brij 35) at 37 °C for 0–12 min. To determine the initial velocity and maximal velocity of the HRI and eIF2α kinase reaction, 50 nM HRI, 1.25–40 μM eIF2α and 40 μM ATP were reacted in PK buffer at 37 °C for 0–10 min. To determine the IC_50_ of haemin for HRI alone or HRI with S-DELE1, 50 nM HRI or HRI–S-DELE1 was incubated with 0–20 μM haemin at 25 °C for 10 min and then reacted with 5 μM eIF2α and 40 μM ATP in PK buffer at 37 °C for 10 min. Reactions were terminated by the addition of SDS sample buffer. Next, 3 μl of the reaction was subjected to dot blot analysis using nitrocellulose membranes and a phospho-eIF2α(Ser52) antibody.

### Mass photometry

The molecular mass of HRI or DELE1 was determined by mass photometry. Measurements were performed using a TwoMP mass photometer (Refeyn). Before each measurement, the focus was adjusted by applying 10 μl mass photometry buffer (30 mM HEPES pH 7.5, and 100 mM NaCl) to a new flow chamber. Next, 10 μl HRI or DELE1 was added to the mass photometry buffer to a final concentration of 50 nM immediately before mass photometry measurements. Videos were recorded for 60 s and data were collected and analysed using Refeyn AcquireMP 2.3 and Refeyn DiscoverMP 2.3, respectively.

### Sample preparation for determination of HRI phosphorylation sites by MS

To assess phosphorylation levels of overexpressed HRI after haem release and kinase activation by S-DELE1, Strep-tagged HRI purified from human cells was subjected to denaturing gel electrophoresis as described above. Gels were stained with 0.1% Coomassie brilliant blue R in 10% acetic acid and 20% ethanol, destained with 10% acetic acid and 30% ethanol, and HRI gel bands were cut into small pieces and incubated in 100% acetonitrile for 15 min at room temperature. To reduce proteins, samples were incubated in 10 mM DTT at 60 °C for 1 h, followed by a wash in acetonitrile for 15 min at room temperature. To alkylate proteins, samples were incubated in 55 mM chloroacetamide for 30 min at room temperature in the dark. Samples were destained by alternating washes in acetonitrile and 50 mM ammonium bicarbonate for 15 min at room temperature. Protein material was digested overnight at 37 °C using trypsin (10 ng µl^–1^, Promega). After overnight incubation, supernatants were acidified with formic acid (1% final concentration) and dried using a SpeedVac centrifuge (Eppendorf, Concentrator Plus). The samples were resuspended in 0.1% formic acid before liquid chromatography–tandem MS (LC–MS/MS) analysis.

To assess DELE1-dependent phosphorylation of endogenous HRI in response to haem deficiency or repression of HRI phosphorylation by haemin, 293T *EIF2S1*^S49A,S52A^ WT or *DELE1* KO cells were treated as indicated and endogenous HRI was purified using protein-A-coupled EIF2AK1 antibodies as described above, with the exception that the last three washes were performed using detergent-free DISC buffer. After the last wash step, all washing buffer was removed, beads were snap-frozen in liquid nitrogen and stored at −80 °C until further processing. Bead pellets were resuspended in sodium deoxycholate buffer (4% SDC in 100 mM Tris-HCl pH 8.5) and denatured for 5 min at 95 °C with shaking (1,000 rpm). Samples were reduced and alkylated with 10 mM TCEP and 40 mM 2-chloroacetamide for 5 min at 45 °C with shaking (1,000 rpm) and digested with 0.5 µg LysC (Wako Chemicals) and 0.5 µg trypsin (Promega, sequencing grade) for 16 h at 30 °C. After digestion, peptides were acidified with trifluoroacetic acid and desalted using SDB-RPS solid-phase extraction discs (3M). The samples were resuspended in 0.1% formic acid before LC–MS/MS analyses.

### LC–MS/MS data acquisition for determination of phosphorylation sites

Samples were measured on an Eclipse mass spectrometer (Thermo Fisher Scientific) coupled online to a Dionex Ultimate 3000 RSLCnano system (Thermo Fisher Scientific). The liquid chromatography setup consisted of a 75 μm × 2 cm trap column and a 75 μm × 40 cm analytical column, packed in-house with Reprosil Pur ODS-3 1.9 μm particles (Dr Maisch). Peptides were loaded onto the trap column using 0.1% formic acid in water at a flow rate of 5 μl min^–1^ and separated using a 50 min linear gradient from 4% to 32% of solvent B (0.1% (v/v) formic acid and 5% (v/v) DMSO in acetonitrile) at a 300 nl min^–1^ flow rate. nanoLC solvent A was 0.1% (v/v) formic acid and 5% (v/v) DMSO in HPLC-grade water. The Eclipse mass spectrometer was operated in data-dependent-acquisition (DDA) mode and positive ionization mode. Full-scan MS1 spectra were recorded in the Orbitrap from 360 to 1,300 *m/z* at 60,000 resolution using an automatic gain control target value of 100% and a maximum injection time of 50 ms. The cycle time was set to 2 s. Orbitrap readout MS2 scans were performed using higher energy collision-induced dissociation and a normalized collision energy of 30%. For full proteome analysis, the precursor isolation window was set to 1.3 *m/z* with 15,000 MS2 resolution, an automatic gain control target value of 200% and maximum injection time of 22 ms. Only precursors with a charge state of 2–6 were selected and dynamic exclusion was set to 30 s. Endogenous HRI (phospho)peptide levels were monitored using a parallel reaction monitoring assay with the same gradient settings used in the DDA experiment. PROCAL^57^ retention time peptides were added to each sample before LC–MS measurements.

### Processing of raw MS data for determination of phosphorylation sites

DDA raw MS data files were processed using MaxQuant^58^ (v.2.4.0.0) with default settings and intensity based absolute quantification (iBAQ) enabled (protein FDR = 0.01, PSM FDR = 0.01, site FDR = 0.01, maximum missed trypsin site = 2) enabled. Phosphorylation of Ser, Thr and Tyr residues (phospho (STY)) was included as a variable modification, and ‘match between runs’ was activated. Spectra were searched against forward and reverse sequences of the human reference proteome, including isoforms (UniProt UP000005640, taxon ID 9606).

The MaxQuant output was further analysed using Perseus^59^ (v.1.6.15.0). For analysis of differentially phosphorylated sites, phosphosite intensities from the phospho(STY)Sites.txt file were log_2_-transformed and filtered for a minimum number of three valid values in at least one group. Missing values were imputed from the normal distribution, and differentially phosphorylated sites on HRI between HRI + haemin and HRI + haemin + DELE1 samples were identified by two-sided Student’s *t*-test (*S*_0_ = 0.5; permutation-based FDR < 0.05, 250 randomizations). To ensure equal HRI protein expression between samples, iBAQ values from the proteinGroups.txt file for HRI were compared across samples. For the lollipop plot depicting the phosphorylation sites, summed intensities of phosphosites were normalized to the relative expression levels of HRI (derived from log_2_-transformed iBAQ values) in the respective sample.

The recorded parallel reaction monitoring raw MS data files were imported into Skyline-daily^60^ (v.25.1) for data filtering and analysis. A spectral library was constructed with the HRI (phospho)peptide MS/MS spectra identified in the DDA experiment. Peaks were integrated using automatic settings followed by manual curation. The summed area under the fragment ion traces was exported for quantitative comparison. Phosphopeptide intensities were normalized to the summed intensity of unmodified HRI peptides in the respective sample.

### Sample preparation for determination of protein–protein crosslinks by MS

To assess confirmational changes occurring in HRI after haem release and kinase activation by S-DELE1, Strep-tagged HRI purified from human cells in the presence or absence of S-DELE1 was crosslinked for 30 min at room temperature with 125 μM DSS. The crosslinking reaction was terminated by the addition of 25 mM Tris-HCl pH 7.5 for 10 min on ice. Crosslinked proteins were denatured by the addition of 4 M urea in 50 mM Tris buffer. To reduce and alkylate disulfides, 10 mM tris(2-carboxyethyl)phosphine (TCEP, Thermo Fisher Scientific) and 40 mM 2-chloroacetamide (Sigma-Aldrich) were added. Samples were incubated at 37 °C for 20 min. Following incubation, samples were diluted 1:3 with MS-grade water (VWR). Proteins were enzymatically digested overnight at 37 °C using 1 µg LysC and 2 µg trypsin (Promega). After digestion, the reaction was acidified to a final concentration of 1% trifluoroacetic acid (Merck), and peptides were desalted using Sep-Pak C18 1cc vacuum cartridges (Waters). A total of 200 ng peptides was loaded onto Evotips Pure (Evosep).

### LC–MS/MS data acquisition for determination of protein–protein crosslinks

Peptides were eluted from Evotips onto a 15-cm PepSep C18 column (15 cm × 150 µm, 1.5 µm particle size, Bruker Daltonics) using an Evosep One HPLC system, using the 30 samples per day method. MS analysis was carried out on an Orbitrap Exploris 480 (Thermo Fisher), operated in DDA mode. Full MS scans were collected from *m/z* 300 to 1,650 Th at a resolution of 60,000 (at *m/z* 200 Th). The top 15 most intense precursor ions were selected for fragmentation using stepped higher-energy C-trap dissociation at normalized collision energies of 19, 27 and 35. MS2 spectra were acquired with a resolution of 30,000 (at *m/z* 200 Th) across a dynamic *m/z* range. Normalized automatic gain control targets were set to 300% for MS1 and 100% for MS2, with a maximum injection time of 25 ms for MS1 and auto for MS2. Ions with a charge state of +2 were excluded to prioritize crosslinked precursors.

### Processing of raw MS data for determination of protein–protein crosslinks

Raw data were analysed using Proteome Discoverer (v.2.5.0.400), incorporating XlinkX/PD nodes^61^. Crosslinked peptides were identified via database search against a FASTA file containing the relevant protein sequences. DSS/BS3 was selected as the crosslinking reagent. Carbamidomethylation of cysteines was set as a static modification, whereas oxidation of methionines and N-terminal acetylation were defined as variable modifications. Trypsin/P was used as the specified protease, allowing for up to two missed cleavages. Peptide identifications were accepted with a minimum score of 40 and a delta score of at least 4. A 1% FDR at the peptide level was applied for filtering.

### Analytical flow cytometry

For analysis of CHOP(Neon) fluorescence in HAP1 ∆*HRI* cells transiently expressing cDNAs of interest, cells were treated as indicated 24 h after transfection, detached using trypsin–EDTA (0.25%, Gibco) and measured on a BD LSRFortessa flow cytometer (BD Biosciences). Co-transfection of mCherry was used to identify transfected cells.

The mitochondrial membrane potential was measured using the ratiometric probe JC-1 (MedChemExpress, HY-15534), mitochondrial ROS production was determined using the fluorescent ROS probe MitoSOX (Thermo Fisher, M36007) and free cellular iron was assessed using FerroOrange (CST, 36104). Cells were treated as indicated, collected by trypsinization, washed with PBS and incubated with 1 µM MitoSOX, 1 µM JC-1 or 1 µM FerroOrange in serum-free medium for 30 min at 37 °C. Subsequently, cells were washed with serum-free medium and fluorescence was recorded on a BD LSRFortessa flow cytometer. JC-1 was excited at 488 nM and fluorescence was recorded using 585/42 nm (JC-1 monomers) and 530/30 nm (JC-1 aggregates) emission filters. MitoSOX was excited at 405 nM, and emission was recorded using a 610/20 nm filter. FerroOrange was excited at 561 nm and emission was recorded using a 582/15 nm filter.

To assess protein aggregation by flow cytometry, 1 million K562 cells were fixed with 4% formaldehyde (Sigma Aldrich) in PBS for 30 min at room temperature. Subsequently, cells were washed with PBS, permeabilized with PBS supplemented with 0.5% Triton-X100 (Sigma Aldrich) for 30 min at room temperature and stained for 30 min at room temperature with Proteostat (Enzo, ENZ-51023, 1:1,250 in PBS). Before detection, cells were washed once with PBS, and proteostat fluorescence was recorded on a BD LSRFortessa flow cytometer using 488 nm laser excitation and 585/42 emission filters.

All data were acquired using FACSDiva (BD, v.8.0.1) and analysed using FlowJo software (BD, v.10.4). Cells were identified by forward scatter area versus sideward scatter area gating, and doubles were excluded on the basis of sideward scatter height versus area gating. For analysis of HRI domain deletion and truncation mutant activity, the top 10% of mCherry-positive cells were gated and their mean mNeon intensity was divided by the mean mNeon intensity of untransfected (mCherry-negative) cells. For analysis of CHOP(Neon) fluorescence induction by invertebrate DELE1 and HRI constructs in response to DHA, background fluorescence in the green channel (induced by DHA treatment) of untransfected, DMSO-treated or DHA-treated cells was subtracted from the mean mNeon fluorescence of all mCherry-positive cells. MitoSOX, FerroOrange and proteostat data represent unprocessed mean fluorescence intensity values from single cells normalized to the respective control. JC-1 data represent the mean fluorescence intensity of red, JC-1 monomers divided by the mean fluorescence intensity of green JC-1 aggregates and normalized to the DMSO control.

### Microscopy

For analysis of mitochondrial morphology following haem starvation, BJEH cells were plated onto live-cell imaging slides (Ibidi, 80807), treated as indicated, and stained for 30 min with 100 nM TMRM (Thermo Fisher, T668) at 37 °C. Subsequently, cells were washed with normal medium, and images of mitochondria were acquired with an DMi8 (Leica) scanning confocal microscope equipped with a ×40 water-immersion objective (Leica, Plan Apochromat NA 1.1), a white light laser excitation and HyD detector unit (Leica, TCS SP8 X), and a humidified environmental chamber (Okolab, 5% CO_2_, 37 °C). All images were acquired using Leica Application Suite X (Leica, v.3.5.7) and mitochondrial morphology was scored in Fiji (v.2.16.0/1.54p) and ImageJ (v.1.54f). Cells with short, rounded mitochondria were scored as ‘fragmented’, whereas cells containing unusually long and highly connected mitochondria were scored as ‘elongated’.

### Structure predictions

Computational protein structure predictions were obtained using AlphaFold^23^ (v.3) and visualized using UCSF ChimeraX^62^ (v.1.8). The DELE1–HRI interaction was modelled using two copies of HRI(1–143) and two copies of DELE1(238–436) as input. Overlays between human and *H. vulgaris* proteins are based on the following AlphaFold models from UniProt: Q96E52 (192–524), T2M7Q7 (110–391), Q14154 (229–436), T2MDI1 (135–420), Q9BQI3 (63–238; 376–630); and XP047128012.1 (45–220; 303–562).

### Seahorse assays

Mitochondrial function was assessed using an Agilent Seahorse XF Cell Mito Stress Test kit and XFe96/XF Pro FluxPak Mini (Agilent Technologies, 103015-100 and 103793-100, respectively), and the oxygen consumption rate was monitored using a Seahorse XFe96 Analyzer (Agilent Technologies) according to the manufacturer’s instructions. 293T cells (2 × 10^4^ per well for DHA, 1 × 10^4^ per well for SA and SA + NMPP) were seeded in a XFe96/XF 96-well plate coated with poly-l-lysine (Sigma-Aldrich) 1 day before treatment. Cells were then treated for 4 h with 5 μM DHA and for 24 h with 100 μM SA or 100 μM SA + 20 μM NMPP. One hour before the assay, cells were washed twice with Seahorse XF DMEM medium (Agilent Technologies, 103575-100) supplemented with 1 mM pyruvate, 2 mM glutamine and 10 mM glucose (Agilent Technologies, 103578-100, 103579-100 and 103577-100, respectively). Subsequently, cells were placed in a CO_2_-free BioTek Cytation 1 imaging reader, in which bright-field images of each well were taken. The mito-stress test was performed following a standard protocol. For oligomycin, FCCP and rotenone–antimycin A, concentrations of 1.5 μM, 1 μM and 0.5 μM, respectively, were used. To determine cell numbers for normalization, Hoechst 33342 (Thermo Fisher Scientific, 62249) was added to the rotenone–antimycin A solution to reach a final concentration of 10 μM. After completion of the mito-stress test, cells were returned to the BioTek Cytation 1 imaging reader, in which fluorescence images of the Hoechst 33342 signal were taken. The resulting cell numbers were imported into Wave software (v.2.6.1) and normalization was applied. Seahorse Analytics (v.1.0.0.796) was used for analyses.

### Statistics and reproducibility

For the genome-wide genetic screen, 2.19 × 10^7^ single cells were interrogated phenotypically according to their CHOP(Neon) signal and genetically by deep sequencing of gene-trap integration sites. This process produced a total of 2,743,720 unique mutations in the sense orientation of the affected genes. A two-sided Fisher’s exact test was used to calculate enrichment of mutations in the high or low channel, and *P* values were FDR-corrected using the Benjamini–Hochberg method, as previously described^21^.

For haem measurements, data show the mean ± s.d. of at least three independent biological replicates, with each experiment containing technical duplicates or triplicates. The exact number of independent biological replicates (*n*) is stated in the figure legends. HRI haem-binding data were normalized to the average signal intensity of the respective experiment to account for differences in the background signal between biologically independent experiments.

For the phosphorylation analysis by MS of overexpressed HRI (Fig. 4b and Extended Data Fig. 8i), *n* = 4 independent biological replicates were measured in the same run. For the MS analysis of endogenous HRI phosphorylation after DHA or SA treatment (Extended Data Fig. 8j,k), *n* = 3 (WT – DMSO) or *n* = 4 (all other samples) independent biological replicates were measured in the same run. For MS analysis of endogenous HRI phosphorylation after haemin treatment (Extended Data Fig. 8l), *n* = 4 independent biological replicates were measured in the same run. For cross-linking MS experiments (Fig. 4c,d and Extended Data Fig. 8p), *n* = 4 independent biological replicates were analysed in the same run, and crosslinks identified in at least three independent biological replicates are depicted.

Immunoblots and protein gels are representative of at least three independent biological replicates with similar results obtained. Quantifications of immunoblotting data and the corresponding statistical analyses show the mean ± s.d., with the sample size (*n*) stated in the figure legends. Immunoblotting data were acquired using Image Lab (Bio-Rad, v.5.2) or FusionCapt Advance FX7 (Vilber, v.17.04a). Chemiluminescence was quantified using Image Lab (Bio-Rad, v.5.2) and normalized to the average signal intensity of the respective experiment and to a loading control to account for differences in the background signal between biologically independent experiments and protein loading, respectively^63^. Uncropped blots and protein gels are presented in Supplementary Fig. 1.

Mitochondrial morphology data show the mean ± s.d. of *n* = 3 independent biological replicates, with each experiment containing at least 30 cells per condition.

Analytical flow cytometry and Seahorse data show the mean ± s.d. of *n* = 3 independent biological replicates, with each experiment containing a minimum of technical triplicates.

No statistical methods were used to predetermine sample sizes. The experiments were not randomized, and investigators were not blinded to allocation during experiments and outcome assessments. Statistical significance was determined by *t*-tests (comparison of two groups) or one-way or two-way ANOVA (comparison of multiple groups) tests followed by the appropriate multiple comparison test using GraphPad Prism 10. All statistical tests are two-sided. The specific test used in each experiment is indicated in the figure legends. All numerical values are presented in Supplementary Data 5.

### Inclusion and ethics statement

This study was conducted using commercially available human cell lines. No human participants or animals were involved, and no personally identifiable information was used. All experimental procedures followed institutional biosafety and ethical guidelines. The research team collaborated across different career stages and institutions, with all contributors meeting authorship criteria and being appropriately credited.

### Reporting summary

Further information on research design is available in the Nature Portfolio Reporting Summary linked to this article.

## Online content

Any methods, additional references, Nature Portfolio reporting summaries, source data, extended data, supplementary information, acknowledgements, peer review information; details of author contributions and competing interests; and statements of data and code availability are available at https://doi.org/10.1038/s41586-026-10885-x.

## Supplementary information

Supplementary Information Supplementary Fig. 1, which includes the uncropped immunoblots and gels used in this study.

Reporting Summary

Supplementary Table 1 Oligonucleotides used in this study.

Supplementary Table 2 Gene blocks used in this study.

Supplementary Table 3 Antibodies used in this study.

Supplementary Data 1 Results of the haploid genetic screen for *CHOP*^*Neon*^ regulators using DHA.

Supplementary Data 2 Results of phospho-MS data analyses.

Supplementary Data 3 Results of analysis of endogenous HRI phospho-MS data.

Supplementary Data 4 Results of XL-MS data analyses.

Supplementary Data 5 Source data for graphs in this study.

Peer Review File

## Data Availability

The raw data for HRI crosslinks in the presence of absence of S-DELE1 is available via ProteomeXchange with the identifier PXD063587. The raw data for overexpressed HRI phosphorylation in the presence of absence of S-DELE1 is available via ProteomeXchange with the identifier PXD062916. The raw data for endogenous HR1 phosphorylation can be accessed on the Panorama web repository server under the PRIDE identifier PXD074030. Deep-sequencing datasets have been deposited into the NCBI Sequence Read Archive under accession code PRJNA1263803. The human reference genome assembly hg19 is available from NCBI (PRJNA31257).
