# Supplementary material for: An ancient mitochondrial program tunes translation to haem availability
Source: Nature. 2026 Aug 5;657(8133):1027–34. doi: 10.1038/s41586-026-10885-x (PMC13600944; doi:10.1038/s41586-026-10885-x)
Supplement: Supplementary file 1 [file 41586_2026_10885_MOESM11_ESM.pdf]

nature portfolio

Peer Review File

An ancient mitochondrial program tunes translation to heme availability

Corresponding Author: Professor Lucas Jae

Parts of this Peer Review File have been redacted as indicated to maintain patient confidentiality.

Any redactions in this file are there to maintain patient confidentiality, the confidentiality of unpublished data, or to remove third-party material.

Attachments originally included by the reviewers as part of their assessment can be found at the end of this file.

Version 0:

Reviewer comments:

Referee #1

(Remarks to the Author)
In this manuscript, the authors probe the relationship between heme binding and DELE1 binding in the activation of the ISR kinase HRI. The show that heme deficiency activates ISR through a mitochondrial mechanism involving OMA1-DELE1-HRI signaling, challenging the traditional dogma in the field that HRI directly senses heme levels. Using genetic and biochemical approaches, they go on to further define the interactions between HRI and S-DELE1 and show that S-DELE1 binding to HRI disrupts heme binding to HRI to promote kinase activation. They go on to show that kinase activation recruits the substrate eIF2a through a auto-phosphorylation cascade that promotes structure remodeling of the active kinase. Finally, they demonstrate that this mechanisms of heme sensing is evolutionarily conserved across organisms.

Overall this is an interesting and compelling manuscript that challenges the traditional dogma of HRI activation. While I do feel the field has been moving in this direction for a while now (starting with seminal work from this group and others from 2020), this manuscript clearly demonstrates the relationship between heme binding and DELE1 binding in regulating HRI activation. The work is well done and generally convincing, although there are a few areas for improvements (see Comments below). However, upon addressing these comments, this manuscript would be suitable for publication in Nature and will be high interest to communities interested in mitochondrial regulation, stress-responsive signaling, and global cellular biology.

Comment.

1. How does heme depletion impact mitochondrial biology and function? The authors should probe how different heme depleting conditions impact mitochondrial biology (e.g., membrane potential, ETC activity, morphology). This will provide additional insights into how reductions in heme induce OMA1-dependent DELE1 processing and further link mitochondrial dysfunction to HRI activation induced by heme deficiency. For example, does heme depletion also induce OMA1-dependent OPA1 processing to drive mitochondrial fragmentation? These are important questions to further relate the 'heme sensing' activity of HRI to mitochondrial dysfunction and OMA1-DELE1 signaling.
2. Small thing, but I'd recommend changing Figure 1g to show the cell type then tissue the cell type is derived in parentheses above the figure (E.g., U2OS (bone)). As it is, it can be a bit confusing and it is important to be clear in this figure to indicate that this is all done in cell lines.
3. In Figure 2g,h, the authors indicate that S-DELE1 is shown in monomeric form, but the size of the observed species is unclear based on the MW markers (lowest is 146 which wouldn't exclude the possibility the observe band is a dimer). Can the authors better define the size in cellulo and in vitro (e.g., is it a monomer or a dimer)? Not a huge point, but would be good to better clarify the specific size of this species.
4. It would be useful to quantify some of the immunoblots from key experiments. For example, the authors claim that hemin addition does not influence ATF4 expression in cells co-expressing HRI and S-DELE1, but I do not a reduction at higher doses of hemin. This doesn't disprove their hypothesis (as one would expect that S-DELE1 would shift the competition not eliminate it) but some quantifications that show these effects more clearly would be useful.

5. Along similar lines #4, how does HRI overexpression alone robustly activate this kinase? Does DELE1 accumulate upon HRI overexpression? If not, does the overexpression overwhelm available heme rendering some protein heme unbound? If that is the case, why does addition of hemin not fully suppress this activity. Some more clarification on this point would be helpful.
6. The authors do a nice job of showing that S-DELE1 binding reduces heme binding to HRI allowing activation both in vitro and in vivo. It would be nice to include other studies that show that the activation of OMA1-DELE1-HRI signaling induced by mitochondrial insults (e.g., CCCP, oligomycin) similarly induce heme dissociation from HRI.
7. Along the same lines a #6, the authors use an indirect HRP assay to monitor heme binding to HRI. Could they also use an alternative assay that directly monitor heme binding to HRI (e.g., looking for Soret peaks in immunopurified HRI)? This would be a more direct measure of HRI heme binding that would further support their findings in cells.
8. Does the ISR regulate heme biosynthesis? I get that ISR activation in the context of maturing RBCs could regulate globin synthesis through ISR-dependent translation attenuation, but with these findings that HRI is sensing heme deficiency through mitochondria signaling could suggest that the ISR, maybe even specifically activated by HRI, regulates heme biosynthesis within mitochondria. Could the authors potentially speculate as to why sensing heme deficiency through this mechanism could be beneficial, particularly to cells that are not maturing RBCs.

Referee #2

(Remarks to the Author)
This manuscript proposes a substantial revision to the current model of heme sensing via HRI, highlighting the mitochondrial OMA1-DELE1 axis as central to this process. Specifically, the authors examined the interaction between S-DELE1 and HRI and its role in regulating HRI sensing of cytosolic heme fluctuation. The biochemical and genetic experiments are comprehensive. However, the conclusions are often overstated, and some critical experiments are missing or insufficiently quantified. Prior literature from the author's own work is not reconciled with the current findings, and several technical inconsistencies (e.g., HRI migration patterns, heme assays) undermine confidence in the proposed model. The manuscript would benefit from a more balanced interpretation, clearer methodological descriptions, and additional mechanistic validation—particularly regarding the interaction between S-DELE1 and HRI, heme displacement, and the functional necessity of this pathway under physiologically relevant conditions.

Major:
1. My first and foremost concern is the scientific rigor and reproducibility of the author's results. Many results contradict previously published findings or are inconsistent within the manuscript, not to mention a lack of quantification and statistical analyses (see minor points for details).
For example, the authors previously reported (PMID: 37327776, Fig. 4E) that succinylacetone (SA) treatment does not activate the integrated stress response (ISR), despite inducing ALAS1 expression. In contrast, the current manuscript shows robust ISR activation under SA treatment (Fig. 1). This discrepancy must be addressed. Is this due to differences in treatment duration (6 h vs. 16 h), concentrations, or detection methods? I call this into question because the previous study treated cells with SA for longer times so the cytosolic heme will be further depleted, yet activation of HRI was not observed. Additionally, Figure 1 shows that DELE1 KO cells fail to activate HRI under heme depletion, which is unexpected given prior claims that heme deprivation alone is sufficient to activate HRI through direct cytosolic sensing. If DELE1 functions to activate HRI by displacing inhibitory heme, why is HRI inactive in DELE1 KO cells under heme-deficient conditions? The statement, "we first made use of the observation that in the absence of additional heme, exogenously expressed HRI is constitutively active in cells (Reference 23)," along with the results shown in Fig. 3, also appears inconsistent with the observation that heme deficiency alone is insufficient to activate HRI in DELE1 KO cells.
Reproducibility is another major concern. What was the concentration of heme used for Fig. 3C and 3F? Why is there a 10-fold increase in apo-HRP activity (for purified WT HRI alone)? Does it mean there is 10-fold difference in heme measurement, even though the experimental conditions are the same? Why is HRI in Fig. 3F not destabilized in the presence of S-DELE1, as in Fig. 3E?
In several figures (e.g., 2K, 2M, 2O), MW markers are missing. This is relevant as to which HRI species is being analyzed (activated or inactivated) since the molecular weight of HRI varies across figures (e.g., 100 kDa vs. 80 kDa). Clarify whether these represent different isoforms, phosphorylation states, or migration artifacts. For example, in Fig. S1H vs. S1I, the HRI MW is clearly different (<80 kDa in S1H vs. ~100 kDa in S1I). Were different HRI species examined in these two experiments? I would like to see the full blots for HRI. In the authors' previous publication (PMID: 32132706), HRI is shown at ~100 kDa, but in this manuscript, HRI migrates at <80 kDa. Across most figures, regardless of cell line or expression, there is only one band for HRI—except for Fig. 4C and S4B. Why is that? It does not seem to be a difference between endogenous vs. exogenous expression of HRI, as Fig. 1F still shows a single band even under activation conditions.
It would also be interesting to determine whether different states of HRI (active vs. inactive) exhibit differing affinities for S-DELE1 and heme, as this would provide additional support for the authors' proposed model. This could be important for understanding how HRI activation might be reversed, potentially through dephosphorylation by a yet-to-be-characterized mechanism (PMID: 11689689).

2. The manuscript claims that S-DELE1 displaces inhibitory heme from HRI to activate it. However, this conclusion is speculative. Evidence for direct displacement is indirect and primarily based on apoHRP assays and co-IP. The data presented in this manuscript can also be explained by a model in which S-DELE1 binds to an allosteric site on HRI, causing a conformational transformation that prevents heme binding. The heme-bound and unbound forms of HRI and their interactions with S-DELE1 need to be determined biochemically (e.g. spectroscopic analysis). A competition assay between DELE1 and heme for purified HRI would significantly strengthen this point.

3. The use of DHA is problematic. DHA is known to interact with heme, not chelate it. Its mechanism in heme depletion is distinct from that of biosynthetic inhibition (e.g., SA or NMPP). A discussion on the specificity of DHA's action is required. DHA also causes oxidative stress and has off-target effects on mitochondrial proteins. It is unclear whether the observed ISR is truly due to heme depletion or secondary to mitochondrial damage.
According to the authors' previous work (PMID: 37327776, Fig. 2A), SA treatment did not induce production of S-DELE1 as observed in DHA-treated cells in this manuscript. Similarly, the same work showed that iron deficiency did not induce S-DELE1 production; instead, full-length DELE1 was arrested on the mitochondrial surface to activate HRI. Together, this suggests that DHA may not be an appropriate proxy for heme depletion in studying the function of DELE1 in HRI activation, particularly since production of S-DELE1 is not necessarily induced under heme-depleted conditions.
This also makes the title of Fig. 2 inappropriate, since the figure only studies the effect of S-DELE1 on HRI activation, with no direct heme depletion experiments—even though the title claims "Heme depletion promotes maturation of inactive HRI dimers."

4. The authors finally conclude: "Altogether, these data suggest that association of cytosolic S-DELE1 molecules with the NTD of HRI during heme deficiency promotes release of inhibitory heme molecules from the kinase to enable HRI activation." Given that HRI is a moderate-affinity heme-binding protein (its kinase activity is inhibited by submicromolar concentrations of heme, PMID: 9874252), cytosolic heme concentrations during heme deficiency are likely to be insufficient to inhibit HRI. So, why is DELE1 still needed to activate HRI?
Under physiological conditions during erythrocyte differentiation, the cytosolic heme concentrations may not fall to the levels mimicked by experimental setups using DHA, SA, or NMPP. Thus, HRI activation may simply result from reduced heme binding, without requiring S-DELE1 (as S-DELE1 may not be produced under these conditions, e.g. Fig. 5A).
It is also curious that although the authors propose S-DELE1 functions under heme-depletion conditions, they study its interaction with and impact on HRI by adding extra heme in Fig. 4. Why not perform the experiment under heme-deficient conditions (with and without S-DELE1) to observe how HRI phosphorylation is affected?

Minor:
1. The figures in this manuscript are difficult to follow. For example, not all SDS-PAGE vs. native PAGE blots is labeled. At minimum, the authors should indicate which blots are not from SDS-PAGE. In many cases, the use of DKO cells or CCCP treatment is not visible in the figure itself—I had to check the legend constantly. There is plenty of empty space in the figures that could be used to clarify labeling.
2. None of the quantifications include statistical analysis. While it may be excessive to quantify every blot, for some key figures (e.g., Fig. 2D, 2P, 3E, 4B), quantification is needed. In particular, changes in ATF4 expression—as an HRI activity reporter—are difficult to assess without quantification.
3. For all co-IP experiments, clarify whether inputs and immunoprecipitates were loaded at equal ratios. For example, in Fig. 2D and Fig. S2M, the pulldown signals exceed the inputs—how is this possible? In Fig. 2I, the first pulldown uses anti-GFP to pull down HRI-GFP. FLAG-DELE1 was co-IPed at levels similar to the input, but less HRI-GFP compared to input was recovered—how can this be?
4. The legend/cartoon of Fig. 2I is confusing. The first pulldown should be removed.
5. Fig. 2O should include a full-length HRI control to compare pulldown efficiency.
6. Fig. S2L should be labeled as SDS-PAGE, not native PAGE, according to the text and the observed MW.
7. Fig. 3K shows vertical lines in the ATF4 blot—are these cropped lanes?
8. I could not find any information about the truncated S-DELE1 (Δ400, Fig. 4B). Which part of S-DELE1 was deleted?
9. Some terminology is undefined. What is StHA (a variant of HA tag?) and NTC (non-treated control)? BN-PAGE presumably refers to blue-native PAGE, but this should be explicitly defined.

Referee #3

(Remarks to the Author)
This manuscript by Zhang et al characterizes the mechanism of HRI activation by the cleavage product of DELE1. Data is presented to demonstrate that HRI activation by DELE1 is through direct binding and multimer formation, displacement of heme from HRI and phosphorylation. The authors then show that this mechanism is evolutionarily conserved from bloodless invertebrates through mammals. This is novel work with potentially broad relevance.

My concerns and suggestions for strengthening the manuscript are as follows:

First, statistical analyses of biological replicates are needed for the data that are presented, such as heme quantifications in Figs 1&3. And although it is appropriate to show representative western blots such as in Figs 1-5 , a quantitative assessment of the bands and relevant statistics (which include data from the biological replicates) also needs to be provided. While the findings appear to be significant, it is not possible to critically evaluate the robustness of the conclusions without this information.

Second, recent work (PMID: 37327776) demonstrates that iron chelation is sufficient to activate HRI even in the presence of added hemin – which blunts HRI activation in this study. This rases the concern that the initiation of DELE1 cleavage by OMA1 does not reflect the loss of mitochondrial heme but rather results from the loss of mitochondrial iron. As an extension, it is unclear whether the activation of HRI induced by DHA, or other treatments (shown by ATF4 & CHOP) is directly blocked by the added hemin or is blocked by the increased iron released from hemin after metabolism by HMOX1. Repeating these studies with iron chelators (or after genetically depleting HMOX1) would determine if the activating event is loss of mitochondrial heme, loss of mitochondrial iron, or both.

In addition, more clarity about the HRP-based heme detection is needed. The authors show very tight technical replicates in this assay; however, comparison of independent experiments performed identically without and with 5-ALA (Fig 1a, 1b, Ext Fig 1b) shows a 5-fold variability (4 AU vs 20 AU) in detection of heme across experiments. This could reflect variability in the assay that may be corrected by calibrating the assay to a heme standard curve, or it could reflect experimental variability raising concerns about the robustness of the data from this assay. Since apoHRP can only detect available heme, including heme bound to proteins with lower affinity for heme than apoHRP, small changes in the prevalence of low vs high affinity hemoproteins in the cells would greatly impact the sensitivity of this assay. Also, is the use of total protein the most appropriate approach to normalization when HRI activation is known to inhibit protein translation which could lead to differential protein levels across samples?

Lastly, writing about an inhibitor of an inhibitor is tough, especially for a general audience such as the Nature readership. Thus, including a figure showing the hypothesis and clearer writing would help. Broadly the writing is dense and difficult to follow.

Referee #4

(Remarks to the Author)
I co-reviewed this manuscript with one of the reviewers who provided the listed reports.

Referee #5

(Remarks to the Author)
I co-reviewed this manuscript with one of the reviewers who provided the listed reports.

Version 2:

Reviewer comments:

Referee #1

(Remarks to the Author)
The authors have addressed all of my comments from the original submission through the inclusion of extensive new data and revisions ot the text. The manuscript is suitable for publication.

Referee #2

(Remarks to the Author)
I thank the authors for making a substantial effort to address the concerns raised in my initial review, and the manuscript is clearly improved. In particular, I appreciate the additional experiments clarifying the relationship between DELE1 and heme binding to HRI. The inclusion of an orthogonal assay (UV–Vis/Soret) alongside the HRP-based approach strengthens the conclusion that DELE1 binding reduces heme occupancy on HRI. This was an important point, and the new data make the mechanism more convincing. The authors also provide a more careful treatment of the HRI overexpression phenotype. The demonstration that ectopic HRI is not fully heme-saturated, and that excess hemin can suppress its activity, addresses my earlier concern. The revised interpretation is reasonable, although this system still likely reflects a non-physiological regime and should be interpreted with caution.
I also find additional data examining HRI heme binding under mitochondrial perturbations useful. The DELE1-dependent reduction in heme occupancy across different stress conditions helps generalize the model beyond a single perturbation. In addition, the expanded analysis of mitochondrial function better links heme depletion to OMA1 activation, which improves the overall framework. Finally, the inclusion of downstream functional readouts (including globin-related outputs) is helpful and adds biological context that was missing before.
However, I still think the central conceptual claim is a little overstated. The data support a model in which DELE1 facilitates heme release from HRI and can override heme-mediated inhibition under stress, but do not fully exclude a direct role of heme in regulating HRI under physiological conditions. Similarly, the claim that heme does not dissociate from HRI in the absence of DELE1 remains somewhat indirect.
Overall, the authors have addressed my main concerns, and the manuscript is now significantly stronger. I would be supportive of publication if the authors moderate their conclusions.

Referee #3

(Remarks to the Author)
This manuscript is greatly improved. Many prior concerns are answered, statistics are now included, and much new data has been added. Some remaining concerns and suggestions follow:

1. It would be useful to include the statistical analysis of the western blots in the main text’s figures, so that the reader does

not have to flip to the Extended data to determine which results are significant and which are not.

2. The interpretation of the K562 experiments remains problematic. During differentiation, erythroid precursors synthesize heme at exceedingly high rates (mature red cells contain ~270x106 hemoglobin tetramers, PMID 24478716) while shutting off expression of most all other genes. Heme is a key regulator during erythropoiesis (PMID 32610133) and most relevantly, induces globin transcription (via releasing Bach1 inhibition, PMID 14660636) in addition to releasing HRI-mediated translation inhibition (PMID 24714526).
The K562 studies indeed show that there is increased beta (HBB) & gamma (HBG) globin expression in OMA, DELE1 or HRI-deficient cells. The contention that this is “blocked by heme”, however, is not sufficiently supported because of the way that data are currently normalized. The issue is that globin expression when heme is present should be higher than that induced by ablation of the HRI axis alone. Thus, it seems likely that there would not be further increase resulting from OMA, DELE1, or HRI ablation, which is also consistent with the data as shown. The authors need to directly compare results with heme to results without heme to justify their conclusion. Otherwise, Lines 282-5 need amendment.

3. To conclude that stress regulates globin expression (line 289 and Ext Data Fig 9k), the authors need to include studies with stressors other than heme, given heme’s physiological role in globin production and erythroid differentiation. Also, if this were indeed the mechanism, how would red cells complete differentiation in the setting of non-heme stress? If cleaved DELE1 displaces heme from HRI throughout erythropoiesis, then HRI would be re-activated, reduce globin translation, and impair hemoglobin production while heme is still being rapidly synthesized by the existing heme biosynthetic enzymes. The excess heme would lead to oxidative damage and cell death in the absence of sufficient globin production.Thus,shouldn’t heme override stress induced DELE1 activation of HRI during erythropoiesis?

4. Additionally, ALAS1 expression is regulated by a heme feedback loop and thus is shut off during erythroid differentiation as is shown in Figs 5f&g. However, in erythroid cells, heme synthesis initiates with the upregulation of ALAS2, an erythroid-specific enzyme which is induced by iron and not inhibited by heme. If the authors intend to monitor heme biosynthesis during differentiation (Figs 5f&g), then ALAS2 is what should be monitored to show high levels of endogenous heme synthesis.

5. The discussion of the OMA-DELE1-HRI axis regulation of fetal globin as potential line of therapy for sickle cell disease and thalassemia (lines 383-9) is an overextension of the data and needs to be toned down or removed. Primary cells and cell lines (i.e., K562) routinely express fetal globin in in vitro culture. Showing that the OMA-DELE1-HRI axis specifically and significantly upregulates fetal globin in primary cells is necessary to make this conclusion.

Referee #5

(Remarks to the Author)
I co-reviewed this manuscript with one of the reviewers who provided the listed reports.

Version 3:

Reviewer comments:

Referee #3

(Remarks to the Author)

Referee #5

(Remarks to the Author)
I co-reviewed this manuscript with one of the reviewers who provided the listed reports.

Open Access This Peer Review File is licensed under a Creative Commons Attribution 4.0 International License, which permits use, sharing, adaptation, distribution and reproduction in any medium or format, as long as you give appropriate credit to the original author(s) and the source, provide a link to the Creative Commons license, and indicate if changes were made.
In cases where reviewers are anonymous, credit should be given to 'Anonymous Referee' and the source.
The images or other third party material in this Peer Review File are included in the article's Creative Commons license, unless indicated otherwise in a credit line to the material. If material is not included in the article's Creative Commons license and your intended use is not permitted by statutory regulation or exceeds the permitted use, you will need to obtain permission directly from the copyright holder.
To view a copy of this license, visit https://creativecommons.org/licenses/by/4.0/

We thank all five reviewers for their thoughtful and constructive comments. All points raised by the reviewers have been addressed in our revised manuscript. Detailed point-by-point responses are provided below. The revisions prompted by this feedback have substantially strengthened the work.

Referee #1 (Remarks to the Author):

In this manuscript, the authors probe the relationship between heme binding and DELE1 binding in the activation of the ISR kinase HRI. They show that heme deficiency activates ISR through a mitochondrial mechanism involving OMA1-DELE1-HRI signaling, challenging the traditional dogma in the field that HRI directly senses heme levels. Using genetic and biochemical approaches, they go on to further define the interactions between HRI and S-DELE1 and show that S-DELE1 binding to HRI disrupts heme binding to HRI to promote kinase activation. They go on to show that kinase activation recruits the substrate eIF2a through an auto-phosphorylation cascade that promotes structure remodeling of the active kinase. Finally, they demonstrate that this mechanisms of heme sensing is evolutionarily conserved across organisms.

Overall this is an interesting and compelling manuscript that challenges the traditional dogma of HRI activation. While I do feel the field has been moving in this direction for a while now (starting with seminal work from this group and others from 2020), this manuscript clearly demonstrates the relationship between heme binding and DELE1 binding in regulating HRI activation. The work is well done and generally convincing, although there are a few areas for improvements (see Comments below). However, upon addressing these comments, this manuscript would be suitable for publication in Nature and will be high interest to communities interested in mitochondrial regulation, stress-responsive signaling, and global cellular biology.

We thank this reviewer for their constructive feedback and for emphasizing the work's departure from current textbook models and its relevance for a broad audience. We address all points raised in detail below.

1. How does heme depletion impact mitochondrial biology and function? The authors should probe how different heme depleting conditions impact mitochondrial biology (e.g., membrane potential, ETC activity, morphology). This will provide additional insights into how reductions in heme induce OMA1-dependent DELE1 processing and further link mitochondrial dysfunction to HRI activation induced by heme deficiency. For example, does heme depletion also induce OMA1-dependent OPA1 processing to drive mitochondrial fragmentation? These are important questions to further relate the 'heme sensing' activity of HRI to mitochondrial dysfunction and OMA1-DELE1 signaling.

We welcome this important point. As suggested by the referee, we comprehensively assessed the effect heme depletion has on mitochondrial biology and function.

We find that in DHA-treated cells, basal ETC flux is increased but inefficient, as evidenced by a decreased spare respiratory capacity, impaired ATP-linked respiration, and increased basal respiration and proton leak, suggesting moderate ETC uncoupling (new Extended Data Fig. 2g, h). Consistently, we observe moderate mitochondrial depolarization (new Extended Data Fig. 3a), heme-reversible OPA1 cleavage (new Extended Data Fig. 1f), and mild mitochondrial fragmentation in DHA-treated cells (new Extended Data Fig. 3c).

Treatment of cells with SA alone or the combination of SA and NMPP impaired electron flow through the respiratory chain, with a decline in basal, maximal, and ATP-linked respiration. The combinatorial treatment also caused an increased proton leak, indicating OXPHOS uncoupling (new Extended Data Fig. 2i-l). Consistently, SA, NMPP, or the combination affected mitochondrial polarization, with SA causing moderate mitochondrial hyperpolarization and NMPP mitochondrial depolarization, respectively (new Extended Data Fig. 3b), and SA-treated cells displayed increased mitochondrial fragmentation (new Extended Data Fig. 3d).

The subtle differences in mitochondrial impact between the various modes of heme depletion are likely reflective of the distinct molecular mechanisms—DHA reacting with existing heme, SA inhibiting early steps of heme biosynthesis, and NMPP blocking the final step. Importantly, despite these differences, heme depletion by DHA or SA leads to mitochondrial release of S-DELE1 (new Fig. 2a and new Extended Data Fig. 2a, b) and its subsequent binding to HRI (Fig. 2b and new Extended Data Fig. 2c), fully consistent with the genetic requirement for OMA1 in this pathway (Fig. 1f-i, and new Extended Data Figs. 2b, 2d-f, and 3k-n).

2. Small thing, but I'd recommend changing Figure 1g to show the cell type then tissue the cell type is derived in parentheses above the figure (E.g., U2OS (bone)). As it is, it can be a bit confusing and it is important to be clear in this figure to indicate that this is all done in cell lines.

We agree that this is a preferable representation. We have changed the figure accordingly (updated Fig. 1h).

3. In Figure 2g,h, the authors indicate that S-DELE1 is shown in monomeric form, but the size of the observed species is unclear based on the MW markers (lowest is 146 which wouldn't exclude the possibility the observe band is a dimer). Can the authors better define the size in cellulo and in vitro (e.g., is it a monomer or a dimer)? Not a huge point, but would be good to better clarify the specific size of this species.

We appreciate the reviewer catching this detail. To provide a more informative presentation, we have updated the Blue Native panels to include the 66 kDa marker. Moreover, we have rigorously tested the oligomerization state of S-DELE1 by two orthogonal approaches: mass photometry and *in situ* crosslinking. Both methods reveal that the vast majority of purified S-DELE1 is monomeric, with only a small fraction present as dimers or higher-order assemblies (new Extended Data Fig. 4n, o). We thus conclude that the DELE1 species migrating between the 66 kDa and 143 kDa markers in BN-PAGE corresponds to monomeric DELE1 (updated Fig. 2g and Extended Data Figs. 4m, 5b). Any minor higher-order species likely reflect overexpression artifacts, as they are not detected for the endogenous protein (updated Fig. 2g).

4. It would be useful to quantify some of the immunoblots from key experiments. For example, the authors claim that hemin addition does not influence ATF4 expression in cells co-expressing HRI and S-DELE1, but I do not a reduction at higher doses of hemin. This doesn't disprove their hypothesis (as one would expect that S-DELE1 would shift the competition not eliminate it) but some quantifications that show these effects more clearly would be useful.

A similar point was raised by the other reviewers. In the revised manuscript, we now include quantification for all key immunoblot results (see list below).

WB Quantifications

**Main**	**Quantification in Ext. Data**	**Ext. Data Fig. + Quantification**
Fig. 1c	New Extended Data Fig. 1c	

Fig. 1d	New Extended Data Fig. 1d	
Fig. 1g	New Extended Data Fig. 2b	
Fig. 1h	New Extended Data Fig. 3k	
Fig. 1i	New Extended Data Fig. 3n	
		New Extended Data Fig. 1e, h-l
		New Extended Data Fig. 2d-f
		New Extended Data Fig. 3g-j, l, m, o
New Fig. 2a	New Extended Data Fig. 4a	
Fig. 2n	New Extended Data Fig. 5i	
		New Extended Data Fig. 4b
Fig. 3a	New Extended Data Fig. 6a	
Fig. 3e	New Extended Data Fig. 6d	
Fig. 3k	New Extended Data Fig. 6k	
Updated Fig. 3l	New Extended Data Fig. 6l	
		New Extended Data Fig. 6h, i, m
Fig. 4b	New Extended Data Fig. 7b	
Fig. 4f	New Extended Data Fig. 7k	
		New Extended Data Fig. 7l, o
Fig. 5c	New Extended Data Fig. 8a	
Fig. 5d	New Extended Data Fig. 8c	
Fig. 5e	New Extended Data Fig. 8j	
New Fig. 5f	New Extended Data Fig. 8l	
New Fig. 5g	New Extended Data Fig. 8m	
New Fig. 5j	New Extended Data Fig. 9h	
		New Extended Data Fig. 8b, f, i, k
		New Extended Data Fig. 9i

We quantified the blots represented by the new Fig. 3e and found that hemin addition up to 20 μM does not significantly affect ATF4 expression in cells co-expressing HRI and S-DELE1 (new Extended Data Fig. 6d). While an individual blot could give the visual impression of a modest effect at higher hemin concentrations (in this case due to slight lane-to-lane loading differences), this was not supported by careful quantitative analysis across independent biological replicates. We therefore replaced the Western blot panels in Fig. 3e, as well as Figs. 3a, k, and l, with representative examples that more faithfully reflect the quantified results from $n \geq 3$ biological repeats (new Extended Data Fig. 6a, d, k, l). We also conducted new experiments *in vitro*, showing that the heme IC50 of DELE1-bound HRI is increased more than 20-fold compared to HRI alone, and that suppression of HRI activity in the presence of DELE1 occurs only at very high heme concentrations (new Extended Data Fig. 6e). Notably, such high concentrations of free heme are only found *in vivo* in mature erythrocytes, which have already eliminated their mitochondria (PMID: 26460266).

5. Along similar lines #4, how does HRI overexpression alone robustly activate this kinase? Does DELE1 accumulate upon HRI overexpression? If not, does the overexpression overwhelm available heme rendering some protein heme unbound? If that is the case, why does addition of hemin not fully suppress this activity. Some more clarification on this point would be helpful.

We apologize for the missing explanation. Spurious kinase activity upon overexpression has been observed for various kinases (PMIDs: 26282175, 30688659), including recombinant HRI purified from baculovirus-infected Sf9 cells (PMID: 9874252). Similarly, we find that in cells, HRI is constitutively active when artificially overexpressed.

Following the reviewer's suggestion, we directly tested whether this behavior results from incomplete heme saturation. To this end, we transiently expressed HRI in the presence or absence of exogenous heme and quantified heme bound to the purified kinase. Using this approach, we find that endogenous cellular heme levels are indeed insufficient to saturate ectopically expressed HRI (new Extended Data Fig. 6c). This explains why, in this artificial setting, HRI acquires activity even in the absence of DELE1 (which is required for release of inhibitory heme from HRI under endogenous conditions).
We next revisited the suppressibility of this spurious HRI activity by hemin supplementation. Careful quantitative analysis across multiple independent experiments shows that addition of excess hemin indeed quenches this activity, consistent with incomplete heme saturation being its underlying cause (updated Fig. 3a and new Extended Data Fig. 6a). Importantly, this inhibitory effect of heme is abolished in the presence of S-DELE1 (updated Figs. 3e, 3k, 3i and new Extended Data Figs. 6d, 6k, 6i), demonstrating that DELE1 overrides heme-mediated inhibition of HRI in cells.
A related point was also raised by Reviewer 2, and we address it with additional data and discussion on pages 9-10 of this rebuttal (the key notion being that heme does not meaningfully dissociate from human HRI unless prompted by DELE1). We have incorporated these data and an explicit explanation of the consequences of ectopic HRI overexpression into the revised manuscript (lines 192–195).

6. The authors do a nice job of showing that S-DELE1 binding reduces heme binding to HRI allowing activation both in vitro and in vivo. It would be nice to include other studies that show that the activation of OMA1-DELE1-HRI signaling induced by mitochondrial insults (e.g., CCCP, oligomycin) similarly induce heme dissociation from HRI.

We thank the reviewer for this excellent idea. Investigating this point has further broadened the scope of our discovery, as we found that these other mitochondrial insults indeed also reduce HRI heme binding in a DELE1-dependent manner (new Extended Data Fig. 6g). Notably, in the absence of DELE1, the amount of heme bound to HRI remains unchanged, even though cytosolic heme levels decline in the case of CCCP (presumably due to disruption of the biosynthetic machinery). This provides additional evidence against spontaneous dissociation of heme from HRI under heme-limiting conditions, and thus against the current dogma of HRI acting as an autonomous cytosolic heme sensor (a point raised by Reviewer 2 and further addressed in our response below).

Together, these new results support a model in which DELE1-dependent heme release from HRI is the mechanistic basis for HRI activity in a general manner, extending to mitochondrial insults not explicitly linked to heme depletion. In further support of this idea, we find that DELE1 can suppress the inhibitory effect heme has on HRI also in cells treated with CCCP or oligomycin (new Extended Data Fig. 6h, i).

7. Along the same lines a #6, the authors use an indirect HRP assay to monitor heme binding to HRI. Could they also use an alternative assay that directly monitor heme binding to HRI (e.g., looking for Soret peaks in immunopurified HRI)? This would be a more direct measure of HRI heme binding that would further support their findings in cells.

We appreciate this point, which was also raised by other reviewers. We developed the apoHRP-based assay because it enables high-throughput and sensitive detection of heme bound to HRI when isolated from its physiological cellular environment—features required to assess HRI heme binding across the diverse experimental settings based on cultured human cells used in this study.

In response to the reviewers' suggestion, we have now benchmarked this approach against the gold standard of measuring the Soret peak by UV-Vis spectroscopy. Consistent with previous reports using recombinant rabbit HRI (PMIDs: 9874252, 10671563), human HRI purified from human cells displayed a characteristic Soret peak at 424 nm. Importantly, binding of S-DELE1 led to a marked reduction in absorbance at 424 nm, indicating heme release from the kinase and orthogonally validating our apoHRP-based measurements. These data are shown in new Fig. 3f, Fig. 3g, and Extended Data Fig. 6f.

8. Does the ISR regulate heme biosynthesis? I get that ISR activation in the context of maturing RBCs could regulate globin synthesis through ISR-dependent translation attenuation, but with these findings that HRI is sensing heme deficiency through mitochondria signaling could suggest that the ISR, maybe even specifically activated by HRI, regulates heme biosynthesis within mitochondria. Could the authors potentially speculate as to why sensing heme deficiency through this mechanism could be beneficial, particularly to cells that are not maturing RBCs.

We thank the reviewer for this thought-provoking question, which indeed invites speculation beyond the core experimental scope of the study. While we have not systematically dissected feedback regulation of heme biosynthesis by the ISR in this work, our data nevertheless provide initial experimental insight into this possibility. Specifically, we find that DELE1 pathway deficiency results in elevated levels of heme biosynthetic enzymes and, correspondingly, increased cellular heme levels (Fig. 5d and new Extended Data Fig. 8e). Similar effects are observed upon prolonged treatment with ISRIB (Extended Data Fig. 8f), consistent with the mitochondrial ISR constituting a major baseline source of ISR signaling under steady-state cell culture conditions (Eckl and Jae, unpublished observations).
Why might mitochondrial heme sensing, as revealed by our study, be advantageous over the previously proposed model of autonomous cytosolic heme sensing by HRI? While both models ultimately converge on HRI activation, the mitochondrial pathway uncovered here additionally engages OMA1 and DELE1, potentially conferring functional benefits beyond translational attenuation alone.
In particular, our data show that heme depletion is accompanied by mitochondrial respiratory dysfunction and increased oxidative stress (new Extended Data Figs. 2g-l and 3e-j). Activation of OMA1 under these conditions may therefore contribute to mitochondrial adaptation or damage mitigation, for example through modulation of mitochondrial dynamics via substrates such as OPA1, which influences respiration and mitochondrial DNA maintenance (PMID: 27189080). Notably, we find that OPA1 is processed in DHA-treated cells (Fig. 1g and new Extended Data Fig. 1f).
In addition, emerging evidence suggests that mitochondrial protease activity and membrane potential can directly impinge on heme metabolism. A recent preprint reports that the mitochondrial glycine/PLP transporter SLC25A38—required for heme biosynthesis (PMIDs: 27476175, 39856062)—is negatively regulated by YME1L1 and stabilized upon mitochondrial depolarization (bioRxiv 2024.05.12.593764; doi: https://doi.org/10.1101/2024.05.12.593764). As heme depletion activates OMA1, which antagonizes YME1L1 (PMID: 26923599), and is associated with mild mitochondrial depolarization, mitochondrial heme sensing may enable metabolic adaptation in parallel with ISR activation. Such coupling could promote stabilization of factors like SLC25A38, thereby helping to counteract heme scarcity and/or mitigate mitochondrial dysfunction arising from heme deficiency.
Notably, mitochondria harbor important hemoproteins yet possess comparatively limited proteostasis and DNA repair capacity, rendering them particularly vulnerable to disturbances in heme homeostasis. This vulnerability may necessitate additional protective and adaptive layers that could be functionally co-engaged by the mitochondrial heme-sensing mechanism uncovered here. Coupling mitochondrial stress signaling to regulation of heme biosynthesis may thus be

broadly beneficial for cellular fitness beyond the erythroid lineage. We now include a brief discussion of these concepts in the revised manuscript (see discussion lines 340-346).

Referee #2 (Remarks to the Author):

This manuscript proposes a substantial revision to the current model of heme sensing via HRI, highlighting the mitochondrial OMA1-DELE1 axis as central to this process. Specifically, the authors examined the interaction between S-DELE1 and HRI and its role in regulating HRI sensing of cytosolic heme fluctuation. The biochemical and genetic experiments are comprehensive. However, the conclusions are often overstated, and some critical experiments are missing or insufficiently quantified. Prior literature from the author's own work is not reconciled with the current findings, and several technical inconsistencies (e.g., HRI migration patterns, heme assays) undermine confidence in the proposed model. The manuscript would benefit from a more balanced interpretation, clearer methodological descriptions, and additional mechanistic validation—particularly regarding the interaction between S-DELE1 and HRI, heme displacement, and the functional necessity of this pathway under physiologically relevant conditions.

We thank Reviewer 2 for their careful and detailed evaluation of the manuscript. We appreciate the reviewer's acknowledgment of the comprehensive genetic and biochemical analyses underlying this work and agree that revising the prevailing model of HRI-mediated heme sensing warrants careful interpretation and strong experimental support. We have taken these concerns very seriously and have revised the manuscript extensively to address each point.

At the outset, we wish to clarify that the new model of HRI signaling emerging from our study does not invalidate prior work showing that heme directly *inhibits* HRI. In fact, this feature of HRI is an important aspect of our model, as it provides a means to boost protein translation when mitochondria are energized and heme levels are high (see discussion lines 357-362 and new Extended Data Fig. 9k). Instead, our work conceptually defines how HRI is *activated* in a cell, revealing a clear mechanistic departure from prevailing ideas. We rigorously demonstrate that HRI does not autonomously acquire activity in response to declining heme levels. Instead, across all conditions examined here (except artificial ectopic overexpression), HRI activation is under strict mitochondrial control. We reveal this mitochondrial grip to be the DELE1 pathway (which had not been discovered at the time most of the earlier HRI literature was generated).

Specifically:

1. Our genetic analyses provide direct and decisive evidence that HRI activation in response to heme depletion requires DELE1. Across multiple distinct heme depletion regimens (SA, NMPP, DHA, genetic knockdown of FECH) and a broad panel of human cell lines derived from diverse tissues, loss of DELE1 abolishes HRI activation and downstream ISR signaling (Fig. 1f-i and new Extended Data Figs. 2b, 2d-f, and 3k-n). Importantly, heme—but not iron—supplementation suppresses ISR signaling at the level of DELE1 (Fig. 1c, d, new Fig. 2a and new Extended Data Figs. 1c-l, 2d-f, 3o, and 4a, b), establishing DELE1 as the essential genetic gatekeeper linking heme availability to HRI activation.

2. Consistent with the mitochondrial control over HRI's response to heme deficiency, we find that heme starvation causes mitochondrial respiratory chain dysfunction (new Extended Data Figs. 2g-l, and 3a, b), leading to DELE1 release into the cytosol where it binds and activates HRI (Fig. 2b and new Extended Data Fig. 4c). HRI activation under heme-deficient conditions is accompanied by DELE1-dependent molecular rearrangements and hyperphosphorylation,

which do not occur in the absence of DELE1 (Fig. 4c-e, g, h, Extended Data Fig. 7f, g, and new Extended Data Fig. 7c-e, h-j). Consistently, HRI activation occurs with a delay following heme depletion (Fig. 1a-d and new Extended Data Fig. 1c, d). Together, these data support the idea that mitochondrial signals are needed for heme sensing through HRI.

3. Using multiple orthogonal experimental strategies, we demonstrate that in the human system, DELE1 binding leads to heme release from HRI *ex vivo* (updated Fig. 3i, j) and reduces HRI heme binding in cells (new Fig. 3f, Fig. 3g, new Extended Data Fig. 6f, g, n). This heme dissociation is directly coupled to HRI activation (updated Fig. 3j, k and new Extended Data Fig. 6k), and a heme-binding-impaired HRI mutant is hyperactive and not further stimulated by S-DELE1 (updated Fig. 3l and new Extended Data Fig. 6l). In response to a question raised by Reviewer 1, we further find that DELE1 also antagonizes HRI heme binding in other settings not explicitly linked to heme starvation (new Extended Data Fig. 6g). Our work thus identifies a general mechanism underlying HRI activation.

4. In full agreement with these observations, we find that HRI requires DELE1 for heme release even when ambient heme concentrations are low because heme does not meaningfully dissociate from HRI by itself (updated Fig. 3i, j and new Extended Data Fig. 6g, n). These results provide a direct mechanistic explanation for the requirement of DELE1 in HRI activation under heme-deficient conditions and, to our knowledge, represent the first experimental assessment of heme binding in HRI activation in a human cellular context.

5. We further broaden the relevance of DELE1-dependent HRI regulation in the erythroid lineage. We demonstrate the impact of the DELE1 pathway on globin biology in erythroid precursors and outline the biomedical potential of our findings. In the revised manuscript, we further show that induction of erythroid differentiation itself elicits ISR signaling—a previously recognized characteristic feature of this process (PMIDs: 31554636; 18454133)—which requires the mitophagy factor NIX (new Fig. 5f and new Extended Data Fig. 8l). Strikingly, we demonstrate that disruption of the OMA1-DELE1-HRI axis phenocopies NIX deficiency with respect to this differentiation-associated ISR activation (new Fig. 5g and new Extended Data Fig. 8m). Finally, our new data demonstrate that the DELE1 pathway counteracts protein aggregation during erythroid differentiation (new Fig. 5h), mirroring observations with Hri in mice subjected to iron-deficient diets. Together, these findings support a role for mitochondrial DELE1 signaling in coordinating erythroid globin regulation and proteostasis.

6. We establish that this biology is broadly relevant beyond the erythroid lineage and is highly conserved. Our evolutionary analyses show that orthologs of OMA1, DELE1, and HRI are already present in very low organisms, including *Hydra vulgaris*, a life form composed of only two epithelial cell layers (Fig. 5i, Extended Data Fig. 9a-c). Notably, these orthologs cooperate seamlessly with their human counterparts despite hundreds of millions of years of evolutionary separation (new Fig. 5j, Extended Data Fig. 9d-i). This indicates that the cellular response to heme deficiency is not restricted to the erythroid lineage of higher organisms but represents an ancient, general principle that appears biologically sensible, given the broad requirement for heme throughout the eukaryotic cell.

In summary, we have expanded and clarified methodological descriptions, incorporated appropriate quantification throughout the manuscript, resolved apparent technical inconsistencies, broadened and contextualized biological relevance, and added extensive new mechanistic data. Together with key experiments from the original submission, our revised manuscript now provides strong genetic, cellular, and biochemical support for a revised model of HRI regulation that distinguishes heme-mediated inhibition from DELE1-dependent activation

under heme-limiting or mitochondrial stress conditions. This framework reconciles prior observations and provides a mechanistic basis for how mitochondrial status governs HRI activity across evolutionary and tissue contexts (Fig. 5k and new Extended Data Fig. 9j, k).

A detailed response to all points raised by this reviewer follows below.

Major:
1. My first and foremost concern is the scientific rigor and reproducibility of the author's results. Many results contradict previously published findings or are inconsistent within the manuscript, not to mention a lack of quantification and statistical analyses (see minor points for details).

We acknowledge the reviewer's concerns regarding scientific rigor and reproducibility. All experiments in this study were performed with at least three independent biological replicates; however, in the initial submission we primarily displayed representative experiments and did not include quantification for immunoblot-based analyses. In the revised manuscript, we have now addressed this explicitly by adding quantifications and/or statistics where appropriate. These are based on a minimum of n = 3 independent biological replicates and are provided throughout the revised figures. For a detailed list of all quantifications, please also refer to Reviewer 1 point #4.

For example, the authors previously reported (PMID: 37327776, Fig. 4E) that succinylacetone (SA) treatment does not activate the integrated stress response (ISR), despite inducing ALAS1 expression. In contrast, the current manuscript shows robust ISR activation under SA treatment (Fig. 1). This discrepancy must be addressed. Is this due to differences in treatment duration (6 h vs. 16 h), concentrations, or detection methods? I call this into question because the previous study treated cells with SA for longer times so the cytosolic heme will be further depleted, yet activation of HRI was not observed.

We thank the reviewer for highlighting this apparent discrepancy. In Sekine et al. 2023 (PMID: 37327776), to which our group contributed a limited subset of experiments—none of which involved SA—SA treatment did not result in clear ISR activation at the analyzed time point (16 h), although a mild increase in ATF4 levels can be discerned upon close inspection of Fig. 4E of that study. In the present study, we find that SA treatment leads to a decrease in heme levels and subsequent robust activation of ISR signaling through the DELE1 pathway.

The differing outcomes can be explained by two experimental factors:

- First, we treated cells for 24h rather than 16h with a more modest dose of SA (SA is often used at a 10-fold higher concentration, including in the mentioned study). At 16h of SA treatment, we likewise do not observe significant HRI activation, despite a decline in cellular free heme levels. This is consistent with our finding that HRI activation requires a mitochondrial signal rather than cytosolic heme depletion alone. Moreover, as discussed in more detail in our response to point #3 raised by this reviewer, unlike DHA, SA does not react with existing heme but inhibits an early step of heme synthesis. Given the long half-life of respiratory chain components (PMID: 34715012), a temporal delay between SA treatment and respiratory chain impairment—and thus mitochondrial DELE1 signaling—is therefore expected.
- Second, fetal bovine serum contains heme, and cells can therefore take up exogenous heme from standard culture media. In the present study, SA treatments were performed in heme-depleted medium (updated Methods section, lines 961-964), which was critical to elicit robust ISR activation when SA was used to interfere with heme synthesis (new Extended Data Fig. 1e).

Additionally, Figure 1 shows that DELE1 KO cells fail to activate HRI under heme depletion, which is unexpected given prior claims that heme deprivation alone is sufficient to activate HRI through direct cytosolic sensing. If DELE1 functions to activate HRI by displacing inhibitory heme, why is HRI inactive in DELE1 KO cells under heme-deficient conditions? The statement, "we first made use of the observation that in the absence of additional heme, exogenously expressed HRI is constitutively active in cells (Reference 23)," along with the results shown in Fig. 3, also appears inconsistent with the observation that heme deficiency alone is insufficient to activate HRI in DELE1 KO cells.

We thank the reviewer for raising this important conceptual point, which goes to the core of the revised model proposed in this study. Below, we clarify how our findings reconcile prior work with the data presented here.

First, a key observation from our analyses in a human cellular context is that heme does not spontaneously dissociate from HRI in a meaningful manner. Instead, DELE1 is required to enable HRI activation. This discovery departs from prior models and necessitates their revision. Importantly, this discrepancy reflects inherent limitations of earlier studies rather than an error in their conclusions. Most prior work—much of it conducted over 20 years ago—relied on *in vitro* assays with purified HRI (often heterologously expressed). Moreover, DELE1 signaling to HRI had not yet been identified. Consequently, any earlier cell-based experiments were necessarily performed in DELE1-proficient cells, precluding recognition of a requirement for DELE1 in enabling heme release from HRI. Consistent with this, Fig. 1 shows that the decline in cytosolic heme and activation of HRI are temporally uncoupled (e.g. 6h vs. 24h for SA, respectively), and that in the absence of DELE1, HRI remains inactive even when heme levels are low for extended periods. Mechanistically, we find that in DELE1-deficient cells, HRI remains heme-bound despite declining cytosolic heme levels (new Extended Data Fig. 6g, n), and that even *in vitro* (i.e. in heme-free buffer) heme release from human HRI requires DELE1 (updated Fig. 3i, j).

Second, heme depletion does not manifest in a cell as a spatially isolated event confined to cytosolic HRI. On the contrary, because mitochondrial heme levels are an order of magnitude lower than cytosolic levels (PMID: 27247412), extreme mitochondrial heme scarcity in the context of a relatively heme-replete cytosol is the more plausible scenario. Moreover, as mitochondria are also the site of *de novo* heme synthesis, they would experience heme deficiency before any other cellular compartment. Consistently, our data indicate that, in human cells, all modes of cellular heme depletion are intrinsically coupled to some degree of mitochondrial impairment. HRI activation follows this heme starvation-induced mitochondrial dysfunction (new Extended Data Figs. 2g-l and 3a-f) or other mitochondrial insults (new Extended Data Fig 6h-i) and strictly requires the mitochondrial DELE1 pathway, because heme deficiency is sensed at the level of this organelle. Mechanistically, DELE1 is released from mitochondria (new Fig. 2a and Extended Data Fig 4a, b), binds to HRI (Fig. 2b, and new Extended Data Fig. 4c), and relieves the kinase of heme inhibition (new Fig. 3f, Fig. 3g, i, j, Extended Data Fig. 6f, and new Extended Data Fig. 6g, j, and n), thereby permitting HRI activation.

Third, this framework also resolves the apparent discrepancy between endogenous loss-of-function experiments and the observation that exogenously overexpressed HRI can be constitutively active in the absence of DELE1. Our data show that in this overexpression setting, endogenous heme is insufficient to saturate the artificially elevated HRI pool, creating a non-physiological state (updated Fig. 3a, Extended Data Fig. 6b, and new Extended Data Fig. 6a, c). Consistently, this can be suppressed by heme addition and mimicked with a heme-binding deficient HRI mutant (updated Fig. 3a-c, l, and new Extended Data Fig. 6a, l). By contrast, in the

range of endogenous expression levels, HRI remains heme-bound and inactive under heme deprivation unless DELE1 is mobilized, explaining the strict genetic requirement for the DELE1 pathway in physiological settings. This distinction is now demonstrated (new Extended Data Fig. 9j, k) and clarified in the revised manuscript (lines 192-195).

Fourth, we now extend our investigation of this point to erythroid differentiation. During this process, HRI levels rise (new Fig. 5f, g) and could, in principle, lead to incomplete heme saturation. Importantly, however, heme production increases concomitantly during erythropoiesis. Regardless, we find that DELE1-dependent signaling remains functionally relevant for HRI activity even in erythroid progenitors, as loss of DELE1 mimics HRI deficiency with respect to ISR activation, globin regulation, and prevention of protein aggregation (Fig. 5c-e, new Fig. 5f-h, and new Extended Data Fig. 8a-c, j, l, m). We envisage that this may serve to coordinate attenuation of globin biogenesis through HRI activation as mitochondrial heme synthesis ceases during the course of erythroid differentiation. Conceivably, at late differentiation stages, especially once mitochondria have been eliminated and DELE1 is no longer present, extensive HRI biogenesis could permit HRI activity due to incomplete heme saturation, analogous to artificial HRI overexpression. This could allow for DELE1-independent control of globin synthesis in late-stage reticulocytes, and we now explicitly acknowledge this in the text (lines 365-367).

Together, these findings establish that, in human cells, heme depletion alone is insufficient to activate HRI, and that DELE1-dependent heme dissociation provides the required signal, originating from mitochondria—the organelle also responsible for heme synthesis.

Reproducibility is another major concern. What was the concentration of heme used for Fig. 3C and 3F? Why is there a 10-fold increase in apo-HRP activity (for purified WT HRI alone)? Does it mean there is 10-fold difference in heme measurement, even though the experimental conditions are the same?

The apo-HRP-based measurements shown in these two assays are not directly comparable due to differences in the experimental design. We have updated the figure legends accordingly. Specifically, in the first panel referred to, HRI was immunopurified from cells stably expressing (via viral transduction) the respective HRI variant at low levels using HRI antibodies coupled to Protein A beads. By contrast, in the other panel, Strep-tagged HRI was transiently expressed by transfection and purified from hemin-treated cells using Strep-Tactin beads, yielding substantially higher amounts of HRI protein. The ~10-fold difference in apo-HRP activity therefore does not indicate a 10-fold difference in heme binding per HRI molecule but rather scales with the increased amount of HRI recovered.

Why is HRI in Fig. 3F not destabilized in the presence of S-DELE1, as in Fig. 3E?

We thank this reviewer for flagging this apparent inconsistency, which is readily resolved by considering the genetic background in which HRI is expressed in the respective experiments (wild-type eIF2α in Fig. 3e vs. non-phosphorylatable eIF2α (S49/52A) in Extended Data Fig. 6f (formerly main Fig. 3F)). We now clarify this through updates to the Methods section, figure legends, and manuscript text, and also include new supporting data explaining these effects:
As clarified above, when HRI is transiently expressed, it is constitutively active because endogenous heme levels are insufficient to saturate the kinase (Fig. 3a and new Extended Data Fig. 6c). Consequently, active HRI causes ISR signaling, thereby limiting its own expression, resulting in relatively low HRI protein levels. Addition of extra heme inhibits HRI activity, allowing for increased HRI expression, as observed in Fig. 3a. Conversely, when S-DELE1 is co-expressed, heme is effectively released from HRI (Fig. 3f, g, and new Extended Data Fig. 6j),

resulting in increased HRI activity and, consequently, reduced HRI expression (Fig. 3e and 3l). For heme measurements, we thus expressed HRI in the ISR-defective eIF2α (S49/52A) genetic background to ensure that HRI levels are identical across conditions with active vs. inactive DELE1 signaling (Reviewer Fig. 1), thereby enabling us to accurately quantify heme on HRI.

[Figure Redacted]

Reviewer Fig. 1: HRI accumulates upon heme addition in an ISR-dependent manner.

In several figures (e.g., 2K, 2M, 2O), MW markers are missing.

We thank the reviewer for pointing this out. Molecular weight markers have been added to the revised figures.

This is relevant as to which HRI species is being analyzed (activated or inactivated) since the molecular weight of HRI varies across figures (e.g., 100 kDa vs. 80 kDa). Clarify whether these represent different isoforms, phosphorylation states, or migration artifacts. For example, in Fig. S1H vs. S1I, the HRI MW is clearly different (<80 kDa in S1H vs. ~100 kDa in S1I). Were different HRI species examined in these two experiments? I would like to see the full blots for HRI. In the authors' previous publication (PMID: 32132706), HRI is shown at ~100 kDa, but in this manuscript, HRI migrates at <80 kDa. Across most figures, regardless of cell line or expression, there is only one band for HRI—except for Fig. 4C and S4B. Why is that? It does not seem to be a difference between endogenous vs. exogenous expression of HRI, as Fig. 1F still shows a single band even under activation conditions.

We apologize that the migration behavior of HRI was not explained more clearly in the initial submission and have improved the immunoblot labeling in the revised figures. We also provide the uncropped immunoblots in new Supplementary Data 1-5, as requested.

Importantly, the apparent differences in HRI molecular weight across figures are fully consistent and reflect the respective gel types used rather than differences in HRI biology. In most figures, samples were resolved on 4-12% Bis-Tris gradient gels run with MOPS buffer. Under these conditions, HRI migrates close to the ~"80kDa" marker, and phosphorylated HRI is not, or only poorly, resolved from the non-phosphorylated species, resulting in a single apparent band even under activating conditions.

By contrast, for Fig. 4c, f, Extended Data Fig. 7l, o, and new Extended Data Fig. 7c-f, some of the samples were resolved on 7.5% Tris-Glycine gels specifically to improve separation of phosphorylated HRI species. We have now labeled these gels "TG", for Tris-Glycine. In this gel system, HRI runs close to the ~"100kDa" marker and phosphorylated HRI is well separated from unphosphorylated HRI, explaining the appearance of multiple bands. Importantly, we experimentally confirmed that these reflect different phosphorylation states rather than alternative isoforms or migration artifacts and that the observed bands do indeed represent HRI. We

demonstrate this by (i) λ-phosphatase treatment, which collapses the higher-molecular-weight species (Fig. 4c), (ii) comparison with the kinase-dead HRI (K196R) mutant, which lacks the shifted species, and (iii) an absence of immune staining in HRI knockout cells (Fig. 4c and new Extended Data Fig. 7o).

Consistently, in our 2020 *Nature* study (PMID: 32132706), HRI also migrates below ~"80kDa" in Fig. 3a and Extended Data Fig. 9e, m on 4-12% Bis-Tris gradient gels using MOPS running buffer, as shown below (Reviewer Fig. 2). Please note that in panels e and m, HRI is expressed as a tagged protein from a cDNA, which introduces additional mass and thus results in slightly slower migration.

[Figure Redacted]

Reviewer Fig. 2: Examples of HRI migration in PMID: 32132706.

Finally, Reviewer Fig. 3 illustrates how protein/marker migration depends on gel chemistry and buffer system. This explains why HRI migrates at an apparent molecular weight of ~"80kDa" in Bis-Tris gels, whereas in Tris-glycine gels it migrates at ~"100kDa".

[Figure Redacted]

Reviewer Fig. 3: Thermo Fisher molecular weight marker reference sheet.

It would also be interesting to determine whether different states of HRI (active vs. inactive) exhibit differing affinities for S-DELE1 and heme, as this would provide additional support for the authors' proposed model. This could be important for understanding how HRI activation might be reversed, potentially through dephosphorylation by a yet-to-be-characterized mechanism (PMID: 11689689).

We agree that understanding how HRI activation might be reversed by dephosphorylation is an important question for future research in this field. Conceptually, however, our model differs from the premise underlying this suggestion: rather than the HRI phosphorylation state dictating affinity for its ligands, DELE1 and heme, our data support the opposite direction of control: binding of heme vs. DELE1 differentially governs HRI phosphorylation and thus activity.

To experimentally test the reviewer's idea of possible differing affinities, we first assessed binding of DELE1 to wild-type HRI, kinase-dead HRI, and the heme-binding-impaired HRI variant in the presence or absence of added heme. These assays demonstrate that DELE1 binds comparably to all of these HRI variants (new Extended Data Fig. 6m). Moreover, heme addition does not meaningfully affect these interactions in cells (new Extended Data Fig. 6m), consistent with the ability of DELE1 to release heme from HRI (Fig. 3f-j).
We next tested the inverse—whether HRI's affinity for heme is influenced by DELE1. To this end, wild-type HRI and kinase-dead HRI were expressed in the presence or absence of S-DELE1 under conditions of excess heme, isolated, and subjected to heme quantification. We find that heme binds comparably to wild-type and inactive HRI (new Extended Data Fig. 6j). Interestingly, DELE1 reduced HRI heme binding to a similar extent for either HRI variant (new Extended Data Fig. 6j), demonstrating that HRI kinase activity is dispensable for DELE1-mediated heme release.

While reversal of HRI activation by dephosphorylation is not the focus of the current study, we experimentally tested whether dephosphorylation alters the interaction between HRI and DELE1. To this end, HRI and S-DELE1 were co-expressed, and DELE1-containing complexes were isolated from cell extracts. Complexes were then incubated with λ-phosphatase prior to analysis by immunoblotting. These experiments revealed that the HRI:DELE1 interaction was not altered by dephosphorylation (Reviewer Fig. 4), indicating that the HRI phosphorylation state does not control its ability to bind DELE1, in line with our model. Consistently, analysis of the phenotypic contribution of any known protein phosphatase in our systematic genome-wide genetic screen for HRI activity under heme deficiency revealed no significant impact for any of them (Reviewer Fig. 5). Recent work by other groups has also identified proteolytic mechanisms for the attenuation of HRI signaling (PMIDs: 36576405, 38297121, 40328314). While these findings do not rule out a layer of HRI regulation by dephosphorylation, they suggest that in the biological contexts investigated here, HRI activity is not primarily governed by phosphatase-mediated reversal. Instead, consistent with our model, association of HRI with its ligands—heme vs. DELE1—determines HRI phosphorylation state and activity, whereas the phosphorylation state itself does not appear to dictate ligand binding.

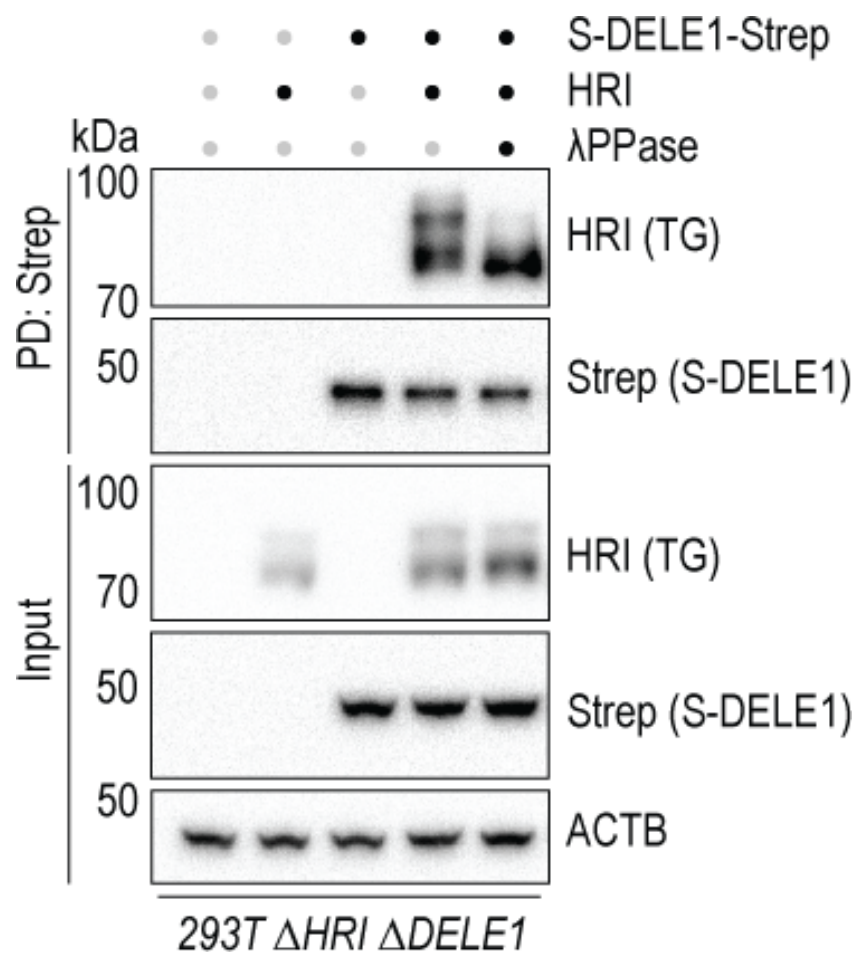

Reviewer Fig. 4: Dephosphorylation doesn't affect HRI binding to DELE1.

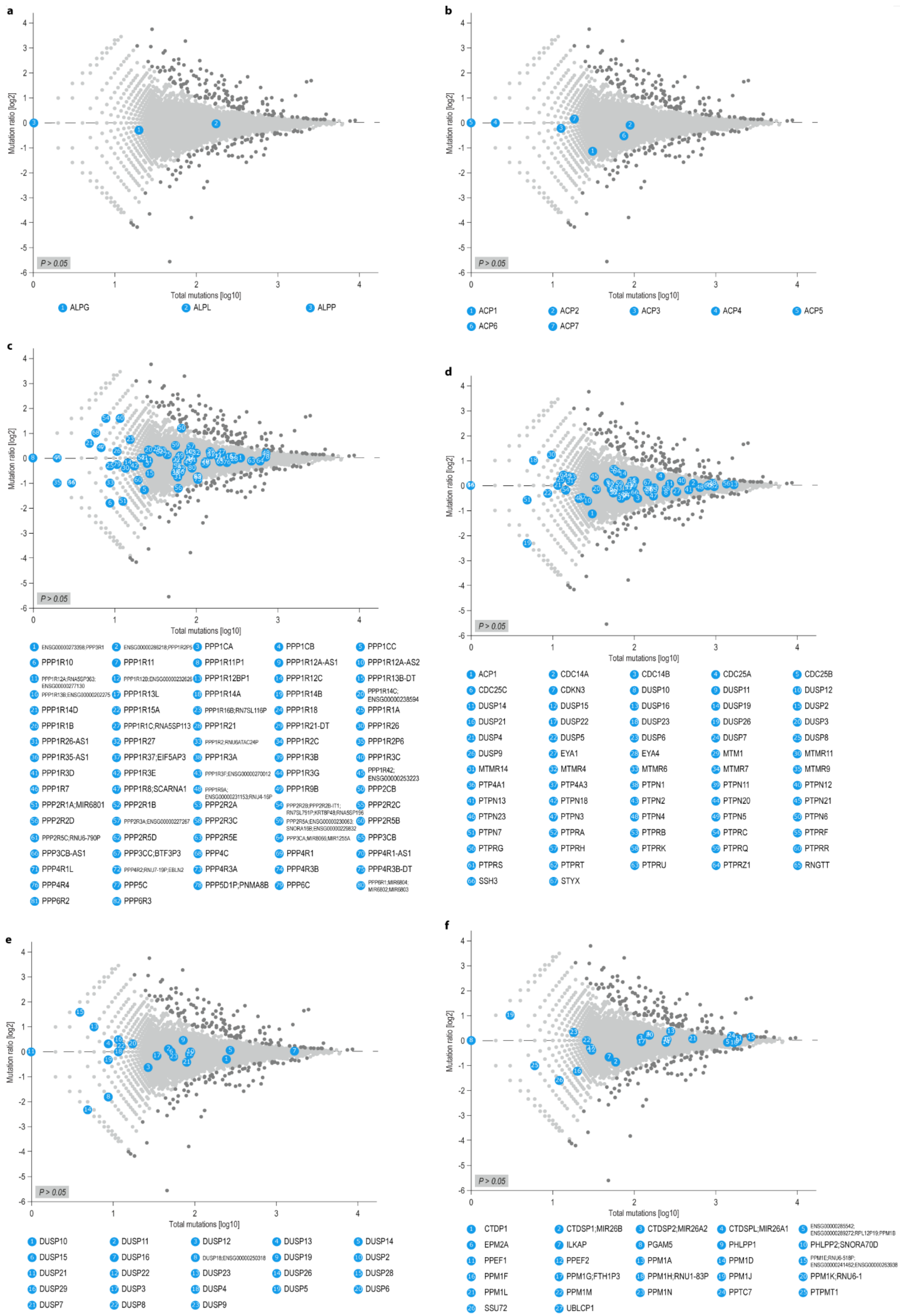
a
b
c
d
e
f
Mutation ratio [log2]
Total mutations [log10]
P > 0.05
1 ALPG 2 ALPL 3 ALPP
1 ACP1 2 ACP2 3 ACP3 4 ACP4 5 ACP5 6 ACP6 7 ACP7
1 ENSG00000273398;PPP3R1 2 ENSG00000285218;PPP1R2P5 3 PPP1CA 4 PPP1CB 5 PPP1CC
6 PPP1R10 7 PPP1R11 8 PPP1R11P1 9 PPP1R12A-AS1 10 PPP1R12A-AS2
13 PPP1R12BP1 14 PPP1R12C 15 PPP1R13B-DT
17 PPP1R13L 18 PPP1R14A 19 PPP1R14B
21 PPP1R14D 22 PPP1R15A 24 PPP1R18 25 PPP1R1A
26 PPP1R1B 27 PPP1R1C;RNA5SP113 28 PPP1R21 29 PPP1R21-DT 30 PPP1R26
31 PPP1R26-AS1 32 PPP1R27 34 PPP1R2C 35 PPP1R2P6
36 PPP1R35-AS1 37 PPP1R37;EIF5AP3 38 PPP1R3A 39 PPP1R3B 40 PPP1R3C
41 PPP1R3D 42 PPP1R3E 44 PPP1R3G
46 PPP1R7 47 PPP1R8;SCARNA1 49 PPP1R9B 50 PPP2CB
51 PPP2R1A;MIR6801 52 PPP2R1B 53 PPP2R2A 55 PPP2R2C
56 PPP2R2D 58 PPP2R3C 60 PPP2R5B
62 PPP2R5D 63 PPP2R5E 65 PPP3CB
66 PPP3CB-AS1 67 PPP3CC;BTF3P3 68 PPP4C 69 PPP4R1 70 PPP4R1-AS1
71 PPP4R1L 73 PPP4R3A 74 PPP4R3B 75 PPP4R3B-DT
76 PPP4R4 77 PPP5C 78 PPP5D1P;PNMA8B 79 PPP6C
81 PPP6R2 82 PPP6R3
1 ACP1 2 CDC14A 3 CDC14B 4 CDC25A 5 CDC25B
6 CDC25C 7 CDKN3 8 DUSP10 9 DUSP11 10 DUSP12
11 DUSP14 12 DUSP15 13 DUSP16 14 DUSP19 15 DUSP2
16 DUSP21 17 DUSP22 18 DUSP23 19 DUSP26 20 DUSP3
21 DUSP4 22 DUSP5 23 DUSP6 24 DUSP7 25 DUSP8
26 DUSP9 27 EYA1 28 EYA4 29 MTM1 30 MTMR11
31 MTMR14 32 MTMR4 33 MTMR6 34 MTMR7 35 MTMR9
36 PTP4A1 37 PTP4A3 38 PTPN1 39 PTPN11 40 PTPN12
41 PTPN13 42 PTPN18 43 PTPN2 44 PTPN20 45 PTPN21
46 PTPN23 47 PTPN3 48 PTPN4 49 PTPN5 50 PTPN6
51 PTPN7 52 PTPRA 53 PTPRB 54 PTPRC 55 PTPRF
56 PTPRG 57 PTPRH 58 PTPRK 59 PTPRQ 60 PTPRR
61 PTPRS 62 PTPRT 63 PTPRU 64 PTPRZ1 65 RNGTT
66 SSH3 67 STYX
1 DUSP10 2 DUSP11 3 DUSP12 4 DUSP13 5 DUSP14
6 DUSP15 7 DUSP16 9 DUSP19 10 DUSP2
11 DUSP21 12 DUSP22 13 DUSP23 14 DUSP26 15 DUSP28
16 DUSP29 17 DUSP3 18 DUSP4 19 DUSP5 20 DUSP6
21 DUSP7 22 DUSP8 23 DUSP9
1 CTDP1 2 CTDSP1;MIR26B 3 CTDSP2;MIR26A2 4 CTDSPL;MIR26A1
6 EPM2A 7 ILKAP 8 PGAM5 9 PHLPP1 10 PHLPP2;SNORA70D
11 PPEF1 12 PPEF2 13 PPM1A 14 PPM1D
16 PPM1F 17 PPM1G;FTH1P3 18 PPM1H;RNU1-83P 19 PPM1J 20 PPM1K;RNU6-1
21 PPM1L 22 PPM1M 23 PPM1N 24 PPTC7 25 PTPMT1
26 SSU72 27 UBLCP1

Reviewer Fig. 5: Genome-wide genetic screen for HRI activity under heme depletion (corresponding to Fig. 1f) does not detect a significant role for phosphatases. ***a-f,*** *Plots and lists of alkaline phosphatases (a), acid phosphatases (b), protein S/T phosphatases (c), protein Y phosphatases (d), dual-specificity phosphatases (e), and ungrouped protein phosphatases (f). Significant genes ($P < 0.05$) are shown in dark grey; all phosphatases lie in the non-significant area.*

2. The manuscript claims that S-DELE1 displaces inhibitory heme from HRI to activate it. However, this conclusion is speculative. Evidence for direct displacement is indirect and primarily based on apoHRP assays and co-IP. The data presented in this manuscript can also be explained by a model in which S-DELE1 binds to an allosteric site on HRI, causing a conformational transformation that prevents heme binding. The heme-bound and unbound forms of HRI and their interactions with S-DELE1 need to be determined biochemically (e.g. spectroscopic analysis). A competition assay between DELE1 and heme for purified HRI would significantly strengthen this point.

Thank you! Reviewers 1 and 3 asked related questions, and we have substantially expanded the experimental support for this conclusion in the revised manuscript.

First, we validated the apoHRP-based heme measurements using UV–Vis spectroscopy, the established gold standard for assessing heme binding in *in vitro* settings. Purified HRI isolated from human cells displays a characteristic Soret peak, and S-DELE1 led to a marked reduction of this signal (new Fig. 3f), fully consistent with the apoHRP-based assays (Fig. 3g). Thus, independent biochemical readouts converge on the conclusion that DELE1 reduces heme occupancy on HRI.

Second, prompted by a question from Reviewer 1, we now show that S-DELE1 also antagonizes heme binding to HRI when it is released into the cytosol in response to mitochondrial insults not explicitly linked to heme biology (new Extended Data Fig. 6g). Notably, in these assays DELE1 was strictly required for reduced HRI heme occupancy, and HRI could not respond to changes in heme availability on its own.

Third, we would like to clarify that DELE1-mediated heme release is directly supported by our data. In updated Fig. 3i, j, purified heme-bound HRI is incubated with purified DELE1 (and its respective mutants) *in vitro*. Our data indicate that this results in heme being transferred from immobilized HRI into the supernatant in an acellular setting, where no new HRI molecules are produced. In other words, HRI molecules that contained heme prior to DELE1 exposure lose heme upon DELE1 binding. We thank the reviewer for pointing out that loss of heme could arise either through direct competition for an overlapping binding site or via an allosteric mechanism that induces heme release (see below).

Fourth, as requested, we now show that DELE1 binding to HRI increases its IC50 for heme by over 20-fold (new Extended Data Fig. 6e). Additionally, as mentioned in response to the previous point, we now include data demonstrating that DELE1 binding to HRI is unaffected when cells are cultured in the presence of 20 μM hemin, irrespective of the kinase activity status (new Extended Data Fig. 6m).

Together, these results fit a scenario of DELE1 and heme competing for an overlapping binding site in the HRI N-terminal domain (Figs. 2j-m and 3a-c), which is also compatible with the computational structure predictions (Figs. 2j and 3d). However, we agree that an allosteric mechanism cannot be excluded, in which DELE1 binding induces a conformational change in the N-terminal domain that causes heme dissociation. We have thus adjusted the text accordingly

throughout. Importantly, this molecular nuance does not affect any of the biological discoveries reported in this study, and our data clearly demonstrate that heme release from HRI is caused by DELE1 binding itself, rather than by any indirect cellular process (updated Fig. 3i, j).

3. The use of DHA is problematic. DHA is known to interact with heme, not chelate it. Its mechanism in heme depletion is distinct from that of biosynthetic inhibition (e.g., SA or NMPP).

We agree with the reviewer regarding the mechanistic distinction between these drugs and would like to respectfully point out that the original manuscript already stated that DHA reacts with heme (rather than chelating it) and that its mode of action is therefore distinct from that of biosynthetic inhibitors such as SA or NMPP. To maximize clarity, this distinction is now made even more explicit in the revised text (line 71-74, 78-80).

Importantly, irrespective of the specific mode of heme depletion, our data consistently demonstrate a strict requirement for the DELE1 pathway to enable the response by HRI. This convergence indicates that the DELE1 pathway responds to heme imbalance itself, rather than to a specific pharmacological trigger.

A discussion on the specificity of DHA's action is required. DHA also causes oxidative stress and has off-target effects on mitochondrial proteins. It is unclear whether the observed ISR is truly due to heme depletion or secondary to mitochondrial damage.

We thank the reviewer for this helpful comment. Rather than limiting our response to discussion, we directly tested the specificity of the effects observed with DHA experimentally.
First, we experimentally tested a possible involvement of mitochondrial ROS generated by DHA (and other heme-depletion strategies) in the elicited HRI signaling. To this end, we measured mitochondrial ROS production in cells treated with DHA, SA, and NMPP, and found that all treatments induced increased mitochondrial ROS at the relevant time point, i.e. just prior to detectable ISR induction (new Extended Data Fig. 3e, f). However, whereas ISR signaling was quenched by heme supplementation, this was not the case for antioxidant treatment with NAC or GSH (new Extended Data Fig. 3g-j), indicating that although ROS formation accompanies different types of heme depletion, ROS are not the trigger of ISR activation in heme-depleted cells, mirroring observations made for OXPHOS inhibition (PMID: 32132707).

Second, we tested if DHA-induced mitochondrial ISR signaling can be reversed by supplementing its target molecule, heme, which we observed (Fig. 1c, new Extended Data Figs. 1c, i, j), indicating that loss of heme—rather than any possible secondary effect—triggers the signaling.

Third, our data demonstrate that the DELE1 pathway is needed for HRI activity irrespective of the mode of heme depletion, whether induced by DHA, SA, NMPP, or genetic knockdown of FECH (Fig. 1g-i, Extended Data Figs. 2e, f, and new Extended Data Figs. 2b, d, and 3k-n).

According to the authors' previous work (PMID: 37327776, Fig. 2A), SA treatment did not induce production of S-DELE1 as observed in DHA-treated cells in this manuscript. Similarly, the same work showed that iron deficiency did not induce S-DELE1 production; instead, full-length DELE1 was arrested on the mitochondrial surface to activate HRI. Together, this suggests that DHA may not be an appropriate proxy for heme depletion in studying the function of DELE1 in HRI activation, particularly since production of S-DELE1 is not necessarily induced under heme-depleted conditions.

We acknowledge that in earlier work to which we contributed (PMID: 37327776)—although no experiments involving SA were performed in our laboratory—robust DELE1 cleavage was not readily detected by direct immunoblotting following SA or iron depletion. Importantly, however, DELE1 is a low-abundance and highly unstable protein, and we find that under relatively mild perturbations such as SA or NMPP treatment, cytosolic S-DELE1 can fall below the detection limit of standard whole-cell immunoblotting, despite functional engagement of the pathway.

Critically, across all heme-depleting conditions tested, HRI activation and downstream ISR signaling are completely abolished in DELE1 knockout cells. This essentially absolute genetic requirement for DELE1 in HRI activation represents the strongest possible evidence for its necessity, even in settings where the protein itself may be challenging to detect due to low abundance.

To directly address the question of DELE1 cleavage biochemically, we revisited DELE1 processing under heme biosynthesis inhibition using more sensitive approaches. First, we probed for DELE1 under heme depletion elicited by DHA or SA in the ISR-defective eIF2α (S49/52A) genetic background and found that cleaved S-DELE1 is readily detectable in this setting (new Fig. 2a; new Extended Data Fig. 4a, b). Second, we isolated cytosolic fractions from WT cells treated with SA and/or NMPP and used HRI as a functional capture handle for DELE1. As previously observed for DHA (Fig. 2b), this also revealed the presence of cytosolic S-DELE1 when heme synthesis was inhibited with SA or NMPP (new Extended Data Fig. 4c). Together, these approaches demonstrate that S-DELE1 is indeed released into the cytosol across different heme-depleted conditions. Importantly, we further show that heme suppresses HRI activation at the level of mitochondrial DELE1 cleavage (new Fig. 2a and new Extended Data Fig. 4a, b).

Thus, while DHA induces particularly robust and readily detectable DELE1 cleavage, likely due to its ability to potently deplete heme by direct interaction, SA- and NMPP-mediated heme depletion engage the same DELE1-HRI signaling axis, albeit at lower amplitude. This clarifies that the production and cytosolic action of S-DELE1 is not unique to DHA treatment, and that DHA serves as a strong but mechanistically consistent perturbation within a broader spectrum of heme-depleting conditions that converge on mitochondrial DELE1-dependent HRI activation.

This also makes the title of Fig. 2 inappropriate, since the figure only studies the effect of S-DELE1 on HRI activation, with no direct heme depletion experiments—even though the title claims “Heme depletion promotes maturation of inactive HRI dimers.”

We have revised the figure title to “Recruitment of S-DELE1 promotes maturation of inactive HRI dimers.”

4. The authors finally conclude: “Altogether, these data suggest that association of cytosolic S-DELE1 molecules with the NTD of HRI during heme deficiency promotes release of inhibitory heme molecules from the kinase to enable HRI activation.” Given that HRI is a moderate-affinity heme-binding protein (its kinase activity is inhibited by submicromolar concentrations of heme, PMID: 9874252), cytosolic heme concentrations during heme deficiency are likely to be insufficient to inhibit HRI. So, why is DELE1 still needed to activate HRI?

This question gets to the core conceptual advance of our study, and we also address it in our opening statement responding to this reviewer’s comments (page 6). While PMID: 9874252 shows that recombinant HRI heterologously expressed in insect cells is inhibited by submicromolar concentrations of heme *in vitro*, this does not imply that heme will spontaneously dissociate from human HRI in living cells once cytosolic heme levels decline. Importantly, the

prevailing model of direct heme sensing by HRI is largely based on inhibition assays using purified HRI *in vitro* and any prior cell-based experiments were necessarily performed in DELE1-proficient cells, since the identity of DELE1 and its role in HRI regulation were unknown at that time.

In the present study, we examined how the heme-inhibited state of HRI is relieved in human cells. We find that, in the absence of DELE1, HRI remains heme-bound even under conditions of pronounced cellular heme deficiency (new Extended Data Fig. 6n). Consistently, purified HRI isolated from human cells retains bound heme even after prolonged incubation in heme-free elution buffer, indicating that heme does not dissociate from HRI to a meaningful extent on its own (updated Fig. 3i, j). By contrast, in the presence of S-DELE1, heme was readily released from human HRI *in vitro* (updated Fig. 3i, j) and, correspondingly, reduced HRI heme occupancy was observed *in cellulo* (new Fig. 3f, g, and Extended Data Fig. 6g, n). Notably, these findings are fully consistent with earlier biochemical studies showing that HRI purifies as a hemoprotein and remains heme-bound despite extensive washing during purification, e.g. PMID: 9874252: HRI is washed in buffer containing 1M salt in this study. Our data extend these observations by demonstrating that, in cells, removal of heme from HRI requires DELE1.

Together, these results explain why DELE1 is essential for HRI activation during heme deficiency despite the moderate affinity of HRI for heme *in vitro*, and they redefine heme sensing by HRI as a mitochondria-controlled process rather than an autonomous cytosolic event.

Under physiological conditions during erythrocyte differentiation, the cytosolic heme concentrations may not fall to the levels mimicked by experimental setups using DHA, SA, or NMPP. Thus, HRI activation may simply result from reduced heme binding, without requiring S-DELE1 (as S-DELE1 may not be produced under these conditions, e.g. Fig. 5A).

We fully agree with the reviewer's point that, under physiological conditions during erythrocyte differentiation, cytosolic heme concentrations may not fall to the levels induced experimentally. However, a natural extension of this consideration is that, under such conditions, heme is unlikely to fall below a threshold at which HRI would become active. Instead, a distinct trigger is required to release heme from HRI and alert the cell to heme synthesis defects (or other forms of mitochondrial impairment). Here we provide rigorous genetic, biochemical, and cell biological evidence that this trigger is DELE1, and that this biology is also active under biological conditions that do not involve experimental inhibition of heme synthesis. Specifically, we find that in K562 cells, DELE1 is required for activation of HRI-mediated ISR signaling during differentiation (new Fig. 5g and new Extended Data Fig. 8m) and counteracts protein aggregation in this setting (new Fig. 5h), supporting a functional role for the pathway during erythroid maturation. That said, we now explicitly acknowledge in the revised manuscript that at late stages of erythroid differentiation direct heme responsiveness of HRI may assume primary regulatory importance (lines 365-367).

From a cell biological standpoint, localization of heme sensing to mitochondria is consistent with their central role in heme synthesis. Likewise, the conservation of mitochondrial heme surveillance upstream of HRI aligns with our evolutionary analyses showing that this pathway predates hemoglobin-based oxygen transport and likely reflects a primordial requirement to monitor cellular heme for its diverse essential functions throughout the cell.

It is also curious that although the authors propose S-DELE1 functions under heme-depletion conditions, they study its interaction with and impact on HRI by adding extra heme in Fig. 4. Why not perform the experiment under heme-deficient conditions (with and without S-DELE1) to observe how HRI phosphorylation is affected?

We appreciate the suggestion, which we have addressed in the following way:

First, we include new phospho-MS data for endogenous HRI isolated from SA- or DHA-treated wild-type or DELE1 knockout cells (new Extended Data Fig. 7h-j and new Supplementary Table 3). These data validate and extend our original phospho-MS analyses performed with overexpressed proteins and, to our knowledge, represent the first assessment of HRI phosphorylation at the endogenous level under heme-starved conditions.

Second, our data demonstrate that HRI phosphorylation—and thus activation—in response to heme depletion depends on DELE1, validating our proposition that S-DELE1 functions under heme depletion conditions (new Extended Data Fig. 7c-e).

Minor:
1. The figures in this manuscript are difficult to follow. For example, not all SDS-PAGE vs. native PAGE blots is labeled. At minimum, the authors should indicate which blots are not from SDS-PAGE. In many cases, the use of DKO cells or CCCP treatment is not visible in the figure itself—I had to check the legend constantly. There is plenty of empty space in the figures that could be used to clarify labeling.

We apologize for this lack of clarity and have updated the figures accordingly.

2. None of the quantifications include statistical analysis. While it may be excessive to quantify every blot, for some key figures (e.g., Fig. 2D, 2P, 3E, 4B), quantification is needed. In particular, changes in ATF4 expression—as an HRI activity reporter—are difficult to assess without quantification.

We now include quantifications and appropriate statistical analyses for all relevant immunoblots in the revised manuscript, including those highlighted by the reviewer.

3. For all co-IP experiments, clarify whether inputs and immunoprecipitates were loaded at equal ratios. For example, in Fig. 2D and Fig. S2M, the pulldown signals exceed the inputs—how is this possible? In Fig. 2I, the first pulldown uses anti-GFP to pull down HRI-GFP. FLAG-DELE1 was co-IPed at levels similar to the input, but less HRI-GFP compared to input was recovered—how can this be?

We apologize for the lack of clarity and have updated the relevant Methods section and figures accordingly. In the referenced co-IP experiments, inputs and pulldowns were intentionally loaded at different proportions to enable visualization of complexes captured at early versus late steps of the capture → elute → re-capture series on the same blot. In Fig. 2d, 0.25% input, 5% first pulldown, and 25% second pulldown were loaded; in Extended Data Fig. 2d (formerly Fig. S2M), 0.25% input, 5% first pulldown, 25% second pulldown, and 100% third pulldown were loaded. As a result, pulldown signals can exceed input signals without indicating increased protein abundance.

In Fig. 2i, the apparent discrepancy reflects both differential loading and the experimental design: HRI-GFP contains a 3C protease cleavage site and was eluted by 3C cleavage, resulting in recovery of both intact HRI-GFP and free GFP. Because the blot was probed with anti-GFP, the pulldown signal represents the sum of these species. To make this more explicit, we revised the panel.

4. The legend/cartoon of Fig. 2I is confusing. The first pulldown should be removed.

Done.

5. Fig. 2O should include a full-length HRI control to compare pulldown efficiency.

Done.

6. Fig. S2L should be labeled as SDS-PAGE, not native PAGE, according to the text and the observed MW.

Thank you. This was a labelling error and has been fixed.

7. Fig. 3K shows vertical lines in the ATF4 blot—are these cropped lanes?

The lanes on this blot were not cropped; the membrane had vertical streaks. We have replaced the panel with a higher-quality blot in the revised manuscript.

8. I could not find any information about the truncated S-DELE1 (Δ400, Fig. 4B). Which part of S-DELE1 was deleted?

Apologies for this oversight. The mutant is C-terminally truncated, ending at residue G400, i.e. missing the last 115 amino acids. We have clarified this by renaming it S-DELE1 (ΔC115).

9. Some terminology is undefined. What is StHA (a variant of HA tag?) and NTC (non-treated control)? BN-PAGE presumably refers to blue-native PAGE, but this should be explicitly defined.

Apologies for the missing information. StHA denotes a Strep-HA dual tag; NTC = non-targeting (sgRNA/shRNA) control. We have added explicit definitions of these abbreviations and "BN-PAGE" in the relevant figure legends and Methods sections.

Referee #3 (Remarks to the Author):

This manuscript by Zhang et al characterizes the mechanism of HRI activation by the cleavage product of DELE1. Data is presented to demonstrate that HRI activation by DELE1 is through direct binding and multimer formation, displacement of heme from HRI and phosphorylation. The authors then show that this mechanism is evolutionarily conserved from bloodless invertebrates through mammals. This is novel work with potentially broad relevance.

We thank this reviewer for their positive overall assessment of our work and their constructive suggestions, which have helped us to further strengthen the manuscript.

My concerns and suggestions for strengthening the manuscript are as follows:

First, statistical analyses of biological replicates are needed for the data that are presented, such as heme quantifications in Figs 1&3. And although it is appropriate to show representative western blots such as in Figs 1-5, a quantitative assessment of the bands and relevant statistics (which include data from the biological replicates) also needs to be provided. While the findings appear to be significant, it is not possible to critically evaluate the robustness of the conclusions without this information.

We agree and now include quantitative analyses and appropriate statistical evaluation for all key datasets in the revised manuscript, including heme measurements, immunoblot analyses, and flow cytometry experiments. All quantitative data are now presented as mean ± SD from at least 3 independent biological replicates, with n = 3 technical replicates per biological replicate for flow cytometry and heme measurements. For a complete list of all included quantifications, please also refer to Reviewer 1 point #4.

Second, recent work (PMID: 37327776) demonstrates that iron chelation is sufficient to activate HRI even in the presence of added hemin – which blunts HRI activation in this study. This rases the concern that the initiation of DELE1 cleavage by OMA1 does not reflect the loss of mitochondrial heme but rather results from the loss of mitochondrial iron. As an extension, it is unclear whether the activation of HRI induced by DHA, or other treatments (shown by ATF4 & CHOP) is directly blocked by the added hemin or is blocked by the increased iron released from hemin after metabolism by HMOX1. Repeating these studies with iron chelators (or after genetically depleting HMOX1) would determine if the activating event is loss of mitochondrial heme, loss of mitochondrial iron, or both.

We thank this reviewer for bringing up this interesting aspect. We have addressed it as follows:

First, using the labile iron stain FerroOrange, we show that the heme-depleting regimens tested do not induce cellular iron starvation (new Extended Data Fig. 1b). Conceivably, if heme depletion were to alter iron availability within the examined time scales, such changes could be effectively buffered by cellular iron stores.

Second—and most important—we demonstrate that ISR induction in response to heme depletion is efficiently quenched by supplementation with heme but not iron (Fig. 1c, d, new Extended Data Figs. 1c-e, f, k, l, and 2d-f).

Third, and as explicitly suggested by the reviewer, we find that heme also suppresses DHA-induced ISR signaling in HMOX1-deficient cells. We further extended this analysis to HMOX2 knockout and HMOX1/2 double-knockout cells, with identical results (new Extended Data Fig. 1g-i). Consistently, DFO addition does not affect the hemin quenching effect on DHA (new Extended Data Fig. 1j). These data demonstrate that the suppressive effect of heme is not mediated by secondary iron release via heme degradation.

Fourth—although not specifically asked here but prompted by a question from Reviewer 2—we also investigated whether ROS stress contributes to ISR activation under heme-depleting conditions. Although all heme-depleting treatments increased mitochondrial ROS production (new Extended Data Fig. 3e, f), consistent with mitochondrial respiration impairment (new Extended Data Fig. 2g-l and 3a, b), ISR signaling was not suppressed by antioxidant treatment (new Extended Data Fig. 3g-j). Thus, while ROS formation accompanies heme depletion, it is not the trigger for ISR activation in this setting.

Together, we conclude that the activating event for ISR signaling in response to SA, NMPP, or DHA is the loss of mitochondrial heme, rather than a secondary effect mediated by iron. However, as noted by the reviewer, iron starvation represents another—albeit weaker (new Extended Data Fig. 1j)—driver of mitochondrial ISR signaling, possibly acting through a distinct mechanism, as proposed in PMID: 37327776.

In addition, more clarity about the HRP-based heme detection is needed. The authors show very tight technical replicates in this assay; however, comparison of independent experiments performed identically without and with 5-ALA (Fig 1a, 1b, Ext Fig 1b) shows a 5-fold variability (4 AU vs 20 AU) in detection of heme across experiments. This could reflect variability in the assay that may be corrected by calibrating the assay to a heme standard curve, or it could reflect experimental variability raising concerns about the robustness of the data from this assay. Since apoHRP can only detect available heme, including heme bound to proteins with lower affinity for heme than apoHRP, small changes in the prevalence of low vs high affinity hemoproteins in the cells would greatly impact the sensitivity of this assay.

We thank the reviewer for pointing out this potential source of confusion and apologize for the lack of clarity in the description of the apoHRP-based heme detection assay. We have addressed the specific feedback as follows (but please also see our feedback to question #7 from Reviewer 1 and question #2 from Reviewer 2 on pages 4 and 16 of this rebuttal).

First, the reviewer is correct in noting assay-to-assay variability in the original apoHRP data. This variability is attributable to technical rather than biological factors, including inter-experimenter differences in lysate preparation and batch-to-batch variability of apoHRP preparations. In the revised manuscript all apoHRP-based measurements are now shown as mean ± SD from n = 3 independent biological replicates. We now explicitly describe the procedure and any normalization, if applicable, in the Methods section.

Second, we independently validated the apoHRP readout by benchmarking it against UV-Vis spectroscopy, the established gold standard *in vitro* method for assessing protein-bound heme. Using this orthogonal approach, we reach the same conclusion: DELE1 binding leads to a robust reduction in heme occupancy on HRI. Thus, independent biochemical readouts converge on the same result (new Fig. 3f and Fig. 3g). Notably, UV-Vis spectroscopy requires comparatively large amounts of purified protein. By contrast, the apoHRP-based assay enables high-throughput and sensitive detection of heme bound to HRI when isolated from its physiological cellular environment. These features were instrumental for assessing HRI heme binding across the diverse experimental settings in cultured human cells used in this study.

Also, is the use of total protein the most appropriate approach to normalization when HRI activation is known to inhibit protein translation which could lead to differential protein levels across samples?

Thank you. We also considered this point. The apoHRP assays in Fig. 1a/b were performed in wild-type cells, whereas the corresponding experiments in Extended Data Fig. 1m (formerly Extended Data Fig. 1b) were carried out in HRI-deficient cells, which do not mount an ISR (and therefore do not repress translation). The results obtained in these two genetic backgrounds are comparable, indicating that potential ISR-dependent effects on total protein levels are negligible under these experimental conditions relative to the magnitude of the observed changes in cellular heme.

Lastly, writing about an inhibitor of an inhibitor is tough, especially for a general audience such as the Nature readership. Thus, including a figure showing the hypothesis and clearer writing would help. Broadly the writing is dense and difficult to follow.

We agree and have revised the text for clarity throughout. In addition, we now include an additional cartoon that places the uncovered biology into a clear conceptual framework (new Extended Data Fig. 9j, k).

We have revised the manuscript in response to the editorial and reviewer suggestions and addressed all points raised. We hope that the manuscript is now suitable for publication. Detailed, point-by-point responses follow below.

Editorial points

STATISTICS: When revising your manuscript, you should ensure that any statistical analysis used is sound and that it conforms to Nature's guidelines). A collection of articles explaining the basics of statistical analysis and advice on how to best present it can be found here.

All statistical analyses meet Nature's guidelines, with reporting provided in the figure legends and Methods section.

REPRODUCIBILITY: All of the checklists provided with the current submission (Reporting summary, and Code and software checklist (if applicable)) should be updated to reflect the revisions made and submitted with the revised manuscript.

The applicable checklist (Reporting Summary) has been updated. As no code was developed in this study, the code checklist has been completed with identifying information only.

LENGTH: In print, biological sciences papers do not normally exceed 8 **OR 6** pages on average; the final print length, however, is at the editor's discretion. The typical length of an 8-page article with 5 modest (quarter-page) display items is 4300 words. **OR The typical length of a 6-page article with 4 modest display items (figures and tables) is 2500 words (summary paragraph plus body text).** If a composite figure (with multiple panels) must occupy at least half a page in order for all the elements to be visible, the text length may need to be reduced accordingly to accommodate such figures. Essential but technical details can be moved into the Methods or Supplementary Information (see below).

The panel distribution has been adjusted to comply with Nature's length requirements, with the main text figures reduced to those panels most essential to the conclusions. The main display items comprise four modest figures (single-column, each well below a quarter page) and one double-column figure (Fig. 5, between a quarter and half page). Accordingly, we have further shortened the text to 4,100 words.

TITLE: Titles cannot exceed 75 characters (including spaces); they must not contain punctuation. We suggest "...".

The title complies with Nature's guidelines (71 characters including spaces, no punctuation).

SUMMARY PARAGRAPH: All Nature papers begin with a fully referenced paragraph, typically no longer than 200 words, aimed at readers in other disciplines. This paragraph starts with a 2- to 3-sentence, basic introduction to the field; continues with a 1-sentence statement of the main findings starting 'Here we show' or an equivalent phrase; and finally, concludes with 2 to 3 sentences putting the main findings into general context so it is clear how the results described in the paper have moved the field forward. A downloadable, annotated example is available here. In some cases it may be necessary to exceed this limit modestly in order to explain complex material for readers in other fields. The extra length, however, is for introduction and context, and not for additional technical information.

The summary paragraph follows the required format and length.

MAIN TEXT: If further introductory material is necessary, the main text can begin with up to 500 words of introduction expanding on the background to the work (some overlap with the summary is acceptable), before proceeding to a concise, focused account of the findings,

and ending with 1 or 2 short paragraphs of discussion. Sections are separated with subheadings (up to 40 characters including spaces) to aid navigation.

The main text has been further streamlined for concision. Subheadings have been shortened to meet the 40-character limit.

REFERENCES: As a guideline, most papers should include no more than 50 main text references; additional references can be cited in (and listed after) the Methods section, as detailed below.

The reference list has been shortened to 50 main text references.

FIGURE LEGENDS: These should be listed sequentially after the main text references and not in the figure files; they should not exceed 300 words each. Each legend should begin with a brief title for the whole figure and continue with a short description of each panel and the symbols used. Each figure legend should contain, for each panel where relevant, the following information:
* the exact sample size (n) for each experimental group/condition, given as a number, not a range;
* a description of the sample collection allowing the reader to understand whether the samples represent technical or biological replicates (including how many animals, litters, cultures, etc);
* a statement of how many times the experiment shown was replicated;
* definitions of statistical methods and measures:
* very common tests (e.g., t-test, simple Chi-square tests, Wilcoxon and Mann-Whitney tests) can be identified by name only, but more complex techniques should be described in the Methods;
* whether tests are one-sided or two-sided;
* whether there are adjustments for multiple comparisons;
* the statistical test results (e.g., P values);
* the definition of 'center values' as median or average;
* the definition of error bars as s.d. or s.e.m.
Any descriptions too long for the figure legend should be included in the Methods section; see here for further explanation.

The figure legends include reporting of sample sizes (n), replicates, statistical tests, and measures of variability; additional details are provided in the Methods where necessary.

METHODS: After the main text figure legends there should be a section entitled "Methods", which provides the full, step-by-step instructions that would allow other researchers to replicate the results. The Methods section will not appear in print but will appear online in the full-text HTML and PDF versions. The Methods section should be written as concisely as possible but should contain all elements necessary to allow interpretation and reproduction of the results. If there are additional references in the Methods section, their numbering should continue from the last reference in the main text, and they should be listed following the Methods section. Specialized methods that require chemical structures, figures or tables, or methods requiring equations, cannot be accommodated in the Methods section of the main text file. If such information is part of the Methods, the entire Methods section must instead be included within a Supplementary Information text file.

The Methods section provides sufficient detail for reproducibility and clarity. References have been updated and continue the numbering from the main text.

ETHICS STATEMENT: For research involving human research participants, the Methods section must include an ethics statement. This statement should provide the name of the

committee that approved the study; confirm that the research was performed in accordance with all relevant guidelines and regulations; and confirm that informed consent was obtained from all participants. If the study was granted an exemption from requiring ethics approval, details of the committee granting the exemption must be included.
For research involving human embryos or gametes, or human stem cells in contexts requiring ethical oversight, the Methods section must include an ethics statement. This statement should provide the name of the committee that approved the study and confirm that the research was performed in accordance with all relevant guidelines and regulations. We encourage authors to follow the principles laid out in the 2021 ISSCR Guidelines for Stem Cell Research and Clinical Translation: https://www.isscr.org/guidelines. Where necessary, the ethics statement should also describe the conditions of donation of materials, such as human embryos or gametes, and confirm that informed consent was obtained from all donors of cells or tissues.

This study was conducted exclusively using commercially available human cell lines and did not involve human participants or primary human materials.

MAIN TEXT STATEMENTS: Several statements (which will not appear in print but will appear online in the full-text HTML and PDF) are required after the Methods, and before the Extended Data legends. First, there should be an Acknowledgements section, listing grant/financial support. Next, we require a detailed Author Contribution statement; the specific contributions of each author, particularly in terms of which authors performed which specific experiments, must be listed. This is followed by a Competing Interest statement. Financial or non-financial interests should be noted here, as well as any patents; patent information should include at a minimum what is covered by the patent and who submitted the patent application. Finally, an Additional Information statement should include information regarding reprints and permissions and name the author(s) to whom correspondence and requests for materials should be addressed. For details of "end note" style and an example see here.

All required statements are included.

DATA AND CODE AVAILABILITY STATEMENTS: All original research manuscripts published in Nature Portfolio journals must include a Data availability statement (DAS). This statement must make the conditions of access to the “minimum dataset” that is necessary to interpret, verify and extend the research in the article, transparent to readers. This minimum dataset may be provided through deposition in public community/discipline-specific repositories, custom proprietary repositories for certain types of datasets, or general repositories like Figshare, Zenodo and Dryad. Providing large datasets in supplementary information is strongly discouraged and the preferred approach is to make data available in repositories. More information on Nature Portfolio’s reporting standards and preparing your Data availability statement can be found here.

For all studies using custom code or mathematical algorithms that are deemed central to the conclusions, a Code availability statement (CAS) must be included, indicating whether and how the code or algorithm can be accessed, including any restrictions to access. the CAS should be provided as a separate section after the DAS but before the references. Code should be deposited in a DOI-minting repository such as Zenodo, Gigantum or Code Ocean and cited in the reference list. We encourage you to manage subsequent code versions and to use a license approved by the open source initiative. Additional details can be found here.

Wherever possible the data used in the paper should be placed into a public data repository or presented as Supplementary Information. If data can only be shared on request, please explain why in the DAS and in the cover letter. The DAS must list which data are included (e.g. by figure panels and data types) and mention any restrictions on availability. If a

dataset generated or analysed during the study is publicly available and has a unique DOI, please include it in the reference list and cite the dataset in the Methods. Accession numbers for any newly determined sequences, structures, microarray or zoobank data; project IDs for MG-RAST data; accession numbers for X-ray crystallographic coordinates and structure factor files; or comparable NMR or cryoEM data, should be included only in the DAS.

A Data Availability Statement is included, with all raw and processed datasets deposited in public repositories and corresponding accession numbers provided (PXD063587, PXD062916, PRJNA1263803, and PRIDE PXD074030); processed proteomics data are available via Panorama. No custom code was developed.

DISPLAY ITEMS: We ask that you take stock of all the data that have been generated throughout the review process and ensure that only the data most central to the conclusions are presented in the main text figures. Although figures can be presented within the text file during the review process, they must be removed from the final text file and uploaded as separate individual files. Figures should be comprehensible to readers in other or related disciplines and assist their understanding of the paper. Figures should be as small and simple as is compatible with clarity. Main text figures (but **not** Extended Data) must be provided in an editable format. Acceptable formats include .ai, .cmx, .cdr, .doc, .eps, .pdf, .ppt, .ps, .psd, .svg and .xls; unacceptable formats include .cvs, .gif, .jpg, .png and .tif. All panels of a figure should be logically connected; each panel of a multipart figure should be sized so that the whole figure can be reduced by the same amount and reproduced on the printed page at the smallest size at which essential details are visible. For guidance, Nature's standard figure sizes are 9, 12, or 18 cm wide; the maximum permitted height of a figure is 17 cm. All panels of figures must be on a single page and assembled into a rectangular shape for publication; any essential alignments (parts horizontal, vertical, spacings of stereo pairs, etc) should be indicated. Nature requests that authors of accepted manuscripts contribute towards the total cost of reproduction of colour figures in print. You will be charged a fee for the print reproduction of colour figures (see here for cost information). Tables should be prepared using the Table menu in Microsoft Word.

The main text figures include only data most central to the conclusions, with additional data presented in the Extended Data. All figures are clearly labeled, formatted as required, and provided as separate editable .ai files.

FIGURE FORMATTING: Lettering in all figures (e.g., labelling of axes) should be in uniform, sans-serif font, in lower-case type, and large enough to permit substantial reduction for publication (the minimum font size permitted is 5 pt). Separate parts of a figure are labelled a, b, etc. Units have a single space between the number and the unit, and follow SI nomenclature or the nomenclature common to a particular field. Thousands are separated by commas (1,000). Unusual units or abbreviations are defined in the legend.

The figures consistently use appropriately sized Arial font throughout, except for sequence alignments. Unit labeling follows SI nomenclature and abbreviations are defined in the legends.

IMAGE PRESENTATION: Data figures are integrated into the main paper online. An overview of the key features of this presentation may be found here. We strongly encourage you to consult the guidelines. A discussion of our standards regarding how images should be prepared and presented can be found here.

All images fully comply with Nature's guidelines.

EXTENDED DATA: Extended Data do not appear in print but are included online within the full-text HTML and integrated in the downloadable PDF. Extended Data are an integral part

of the paper, and only data that directly contribute to the main message should be included. All Extended Data must be referred to in the main text, and their legends should be listed sequentially at the end of the main text, not in the Extended Data files. The Extended Data should be assembled into a maximum of 10 A4 size, multi-panelled display items, submitted as individual files in .jpg, .tif or .eps format only. They should be of the same quality as print figures, but there are important differences in their formatting. More specific instructions can be found here. We encourage authors who are describing complex processes to include a schematic of the main finding as part of the Extended Data to aid readers unfamiliar with the immediate discipline.

The Extended Data (ten display items) have been re-organized to accommodate the reduction of main figure panels and comply with the requirements.

SUPPLEMENTARY INFORMATION: Supplementary Information (SI) is online-only, peer-reviewed material that is essential background to the study (e.g., large data sets, more complex methods, and calculations), but which is too large or impractical, or of interest only to a few specialists, to justify inclusion in the print version of the paper (see here for further details). While SI should not typically contain data figures (any figures additional to those appearing in the main text should be formatted as Extended Data), we require that the raw, uncropped data for gels be presented as an SI figure (see below). Tables may be included in SI, but only if they are unsuitable for formatting as Extended Data (e.g., tables containing large data sets or raw data tables that are best suited to Excel files). If a manuscript has SI, each discrete item of the SI (e.g., videos, tables) must be referred to at an appropriate point in the main manuscript. We ask that you provide a word file entitled “SI Guide”, containing a cover page with manuscript title and author information; a table of contents (preferably with page numbers); and then any SI text, notes, figures, and titles and legends for any separate SI files. Additional information can be found here.

Please pay careful attention to the formatting of the SI because it is not subedited. After the paper has been accepted, SI files can only be amended for critical changes to the scientific content, not for style.

The Supplementary Information is organized with an “SI Guide” Word file and all items appropriately referenced in the main text.

SOURCE DATA (GRAPHS): To increase transparency, we strongly encourage you to provide, in spreadsheet form, the data underlying the graphical representations used in the figures. In the case of all experiments presenting data from animal models, this is a requirement and is not optional. This is in addition to our well-established data-deposition policy for specific types of experiments and large datasets. Online readers of the manuscript will be able to access the graphical source data directly from the figure legend. Spreadsheets can only be submitted in .xls, .xlsx or .csv formats. One file per figure is permitted. If there is a multi-panelled figure, the source data for each panel should be clearly labeled in the file; alternatively the source data for a figure can be included in multiple, clearly labeled sheets within an Excel file. File sizes of up to 30 MB are permitted, but it is expected that the vast majority of graphical source data files will be considerably smaller than this. When submitting these files with your manuscript, you should select the "Source Data" file type and use the title field in the file description tab to indicate the figure(s) to which the source data pertain.

Source data underlying the figures are provided in spreadsheet format (Supplementary Data 1), with tabs corresponding to individual figures.

RAW DATA (GELS): You must provide the original source images for all data obtained by electrophoretic separation (e.g., EMSA, northern/Southern/western blots, etc). The raw

images must be assembled into a single .pdf or .tif file (multiple gels on a single page is encouraged). The file should be uploaded as Supplementary Figure 1. The full scanned images must be in uncropped form and contain size/molecular weight markers and loading controls. There should be an accurate indication of how the gels were cropped for the final figure. Please clarify in the figure legends and raw data files whether controls (such as beta-actin) were run on the same gel as loading controls, or on separate gels as sample processing controls (see here). While the data can be displayed in a relatively informal style, there must be a correspondence between each source data image and a specific main text or Extended Data figure. The main text or Extended Data figure legends should refer to the uncropped scans explicitly (e.g., "For gel source data, see Supplementary Figure 1."). For examples, see here or here.

Raw gel data are provided in a single annotated and referenced supplementary file (Supplementary Figure 1) as uncropped images with molecular weight markers and loading controls.

THIRD PARTY RIGHTS: Please identify any content used in your article, whether in the main text, extended data or supplementary information, that comes from a third party. This could include figures, tables, images, videos or text boxes that are reproductions or adaptations of items that have previously been published elsewhere and/or are owned by a third party. It also encompasses pictures taken by professional photographers, maps and images downloaded from the internet. You must obtain the right to use each of these items and provide evidence that you have these rights for your paper. You will also need to give proper attribution to the copyright holders in your paper. We ask that you fill out a Third party rights table and upload this with your manuscript. You can find more information about third party rights here.

We confirm that the manuscript does not contain any third-party content. The Third Party Rights table has been completed with identifying information only.

ORCID: As part of our efforts in improving transparency in authorship, we now request that all authors identified as 'corresponding author' create and link their Open Researcher and Contributor Identifier (ORCID) with their account on Nature's manuscript tracking system prior to acceptance. ORCID helps the scientific community achieve unambiguous attribution of all scholarly contributions. More information and instructions on how to associate your ORCID and MTS accounts can be found here. If you have any issues attaching an ORCID identifier to your MTS account, please contact the Platform Support Helpdesk.

The corresponding author's ORCID identifier is linked to the manuscript tracking system account.

Referee #1 (Remarks to the Author):
The authors have addressed all of my comments from the original submission through the inclusion of extensive new data and revisions ot the text. The manuscript is suitable for publication.

We thank the reviewer again for their constructive feedback.

Referee #2 (Remarks to the Author):
I thank the authors for making a substantial effort to address the concerns raised in my initial review, and the manuscript is clearly improved. In particular, I appreciate the additional experiments clarifying the relationship between DELE1 and heme binding to HRI. The inclusion of an orthogonal assay (UV–Vis/Soret) alongside the HRP-based approach strengthens the conclusion that DELE1 binding reduces heme occupancy on HRI. This was an important point, and the new data make the mechanism more convincing. The authors also provide a more careful treatment of the HRI overexpression phenotype. The demonstration that ectopic HRI is not fully heme-saturated, and that excess hemin can suppress its activity, addresses my earlier concern. The revised interpretation is reasonable, although this system still likely reflects a non-physiological regime and should be interpreted with caution. I also find additional data examining HRI heme binding under mitochondrial perturbations useful. The DELE1-dependent reduction in heme occupancy across different stress conditions helps generalize the model beyond a single perturbation. In addition, the expanded analysis of mitochondrial function better links heme depletion to OMA1 activation, which improves the overall framework. Finally, the inclusion of downstream functional readouts (including globin-related outputs) is helpful and adds biological context that was missing before. However, I still think the central conceptual claim is a little overstated. The data support a model in which DELE1 facilitates heme release from HRI and can override heme-mediated inhibition under stress, but do not fully exclude a direct role of heme in regulating HRI under physiological conditions. Similarly, the claim that heme does not dissociate from HRI in the absence of DELE1 remains somewhat indirect.
Overall, the authors have addressed my main concerns, and the manuscript is now significantly stronger. I would be supportive of publication if the authors moderate their conclusions.

We thank the reviewer for their positive assessment. The text has been moderated in the relevant sections (lines 181, 190 ("HRI remained heme-bound" → "most heme remained bound to HRI"), 199, 202 ("remain bound" → "remain largely bound"), and 325-326 ("At late stages of erythroid differentiation, direct heme responsiveness of HRI may assume primary regulatory importance.")).

Referee #3 (Remarks to the Author):
This manuscript is greatly improved. Many prior concerns are answered, statistics are now included, and much new data has been added. Some remaining concerns and suggestions follow:

We thank the reviewer for their favorable assessment and constructive comments. A point-by-point response is provided below.

1. It would be useful to include the statistical analysis of the western blots in the main text's figures, so that the reader does not have to flip to the Extended data to determine which results are significant and which are not.

We agree that quantification directly next to immunoblot data is ideal. While this was not possible in the main figures due to format constraints, most panels have been moved to the Extended Data, where immunoblots are consistently presented with their corresponding quantifications immediately adjacent to them.

2. The interpretation of the K562 experiments remains problematic. During differentiation, erythroid precursors synthesize heme at exceedingly high rates (mature red cells contain ~270x106 hemoglobin tetramers, PMID 24478716) while shutting off expression of most all other genes. Heme is a key regulator during erythropoiesis (PMID 32610133) and most relevantly, induces globin transcription (via releasing Bach1 inhibition, PMID 14660636) in addition to releasing HRI-mediated translation inhibition (PMID 24714526). The K562 studies indeed show that there is increased beta (HBB) & gamma (HBG) globin expression in OMA, DELE1 or HRI-deficient cells. The contention that this is "blocked by heme", however, is not sufficiently supported because of the way that data are currently normalized. The issue is that globin expression when heme is present should be higher than that induced by ablation of the HRI axis alone. Thus, it seems likely that there would not be further increase resulting from OMA, DELE1, or HRI ablation, which is also consistent with the data as shown. The authors need to directly compare results with heme to results without heme to justify their conclusion. Otherwise, Lines 282-5 need amendment.

We welcome this suggestion and now provide data with and without heme on the same membrane (**revised Extended Data Fig. 9k**). This shows that while HBB and HBG levels are indeed higher in the presence of exogenous heme, the increase in this setting is not massively higher than that observed without heme in the KO cells. Still, we agree with the reviewer that in terminal red blood cell differentiation, globin levels rise to levels where changes in globin expression (transcriptional or translational) would be expected to no longer strongly impact overall abundance. Consistent with prior work demonstrating an important role for HRI-mediated ISR signaling in erythropoiesis and globin biology (PMIDs 11726526, 29101239, 32299090), this suggests that the contribution of heme-dependent HRI inhibition to globin levels is most relevant under conditions where heme is not yet saturating, i.e. during earlier stages of erythroid differentiation. We have amended the corresponding text accordingly (lines 258-261 ("blocked by heme" → "largely lost under conditions of heme supplementation causing high globin levels")) and also simplified the label "(globin) translation" to "translation" (**Extended Data Fig. 10k**), to better reflect that HRI's importance in red cell formation may involve translational tuning of mRNAs beyond those encoding globins.

3. To conclude that stress regulates globin expression (line 289 and Ext Data Fig 9k), the authors need to include studies with stressors other than heme, given heme's physiological role in globin production and erythroid differentiation.

We agree that heme has an important role in globin production, with its abundance constraining globin transcription and the assembly of mature hemoglobin. Importantly, however, our data show that the globin reduction observed upon heme depletion is DELE1-dependent (**Extended Data Fig. 9l**), indicating a substantial ISR contribution at the level of globin translation. This is consistent with prior work on the relevance of HRI-mediated ISR signaling for globin biology (PMIDs 11726526, 29101239, 32299090).

As requested, we also tested the effect of the stressor oligomycin (complex V inhibitor) and found that it, too, reduces globin levels in K562 cells in a manner dependent on the OMA1-DELE1-HRI axis (**new Extended Data Fig. 9m**), mirroring our findings with direct heme depletion stress (**Extended Data Fig. 9l**).

Also, if this were indeed the mechanism, how would red cells complete differentiation in the setting of non-heme stress? If cleaved DELE1 displaces heme from HRI throughout erythropoiesis, then HRI would be re-activated, reduce globin translation, and impair hemoglobin production while heme is still being rapidly synthesized by the existing heme biosynthetic enzymes. The excess heme would lead to oxidative damage and cell death in the absence of sufficient globin production.Thus,shouldn't heme override stress induced DELE1 activation of HRI during erythropoiesis?

Continuous mitochondrial stress throughout erythropoiesis is unlikely to be compatible with effective red cell differentiation. Given the extensive dependence of heme synthesis on general mitochondrial homeostasis (reviewed in PMID: 35832791), these processes may not be readily separable. This is evident in multiple settings where mitochondrial dysfunction leads to impaired heme synthesis/anemia (e.g. PMID 18553104, as well as 35832791 and references therein), and consistent with our findings that DELE1-mediated heme dissociation from HRI is likewise observed upon mitochondrial perturbation (**Extended Data Figs. 6i and 7f**). Thus, while DELE1 can reduce globin levels under heme depletion (**Extended Data Fig. 9l**) as well as non-heme stress (**new Extended Data Fig. 9m**) and activate HRI even in heme-replete conditions (**Extended Data Fig. 6i-k** and PMID 32132707), *sustained* mitochondrial dysfunction and continued high heme output are unlikely to coexist physiologically.

Instead, our data favor a model whereby the DELE1 pathway acts as a sentinel that can short-circuit translation (including that of globins) in specific acute settings. Notably, a 2008 Nature study demonstrated that NIX, which is needed for erythropoiesis, causes mitochondrial depolarization, thus providing a trigger for the DELE1 pathway under physiological conditions of declining heme synthesis (PMID 18454133). Consistently, our data show that NIX deficiency eliminates the differentiation-associated ISR, and that this is phenocopied by deficiency in the DELE1 pathway (**Fig. 5a, b and Extended Data Fig. 9o, p**).

Finally, these HRI-related roles of the ISR in red cell differentiation are not adequately captured by the term "stress". We have therefore revised the corresponding text (line 264 and lines 323-325 ("with DELE1 available as a circuit breaker upon a qualifying trigger")) and replaced the label "stress override" with "mitoISR trigger" in **Extended Data Fig. 10k**.

4. Additionally, ALAS1 expression is regulated by a heme feedback loop and thus is shut off during erythroid differentiation as is shown in Figs 5f&g. However, in erythroid cells, heme synthesis initiates with the upregulation of ALAS2, an erythroid-specific enzyme which is induced by iron and not inhibited by heme. If the authors intend to monitor heme biosynthesis during differentiation (Figs 5f&g), then ALAS2 is what should be monitored to show high levels of endogenous heme synthesis.

We have updated **Fig. 5a, b** to show ALAS2 instead of ALAS1. As expected, levels of ALAS2, as well as those of FECH and CPOX, are increased when K562 cells enter differentiation. To probe for ALAS2, the original samples were re-run, and the corresponding loading controls are provided in Supplementary Figure 1 (uncropped immunoblots).

5. The discussion of the OMA-DELE1-HRI axis regulation of fetal globin as potential line of therapy for sickle cell disease and thalassemia (lines 383-9) is an overextension of the data and needs to be toned down or removed. Primary cells and cell lines (i.e., K562) routinely express fetal globin in in vitro culture. Showing that the OMA-DELE1-HRI axis specifically and significantly upregulates fetal globin in primary cells is necessary to make this conclusion.

The indicated text has been removed from the Discussion.

Referees' comments:

Referee #5 (Remarks to the Author):

I co-reviewed this manuscript with one of the reviewers who provided the listed reports.

We thank the editor and all reviewers for their evaluation and constructive feedback.
