# Supplementary material for: An ancient mitochondrial program tunes translation to haem availability
Source: Nature. 2026 Aug 5;657(8133):1027–34. doi: 10.1038/s41586-026-10885-x (PMC13600944; doi:10.1038/s41586-026-10885-x)
Supplement: Supplementary file 2 [file 41586_2026_10885_MOESM1_ESM.pdf]

nature portfolio
https://doi.org/10.1038/s41586-026-10885-x

Supplementary information

An ancient mitochondrial program tunes translation to haem availability

In the format provided by the
authors and unedited

Figure 1c

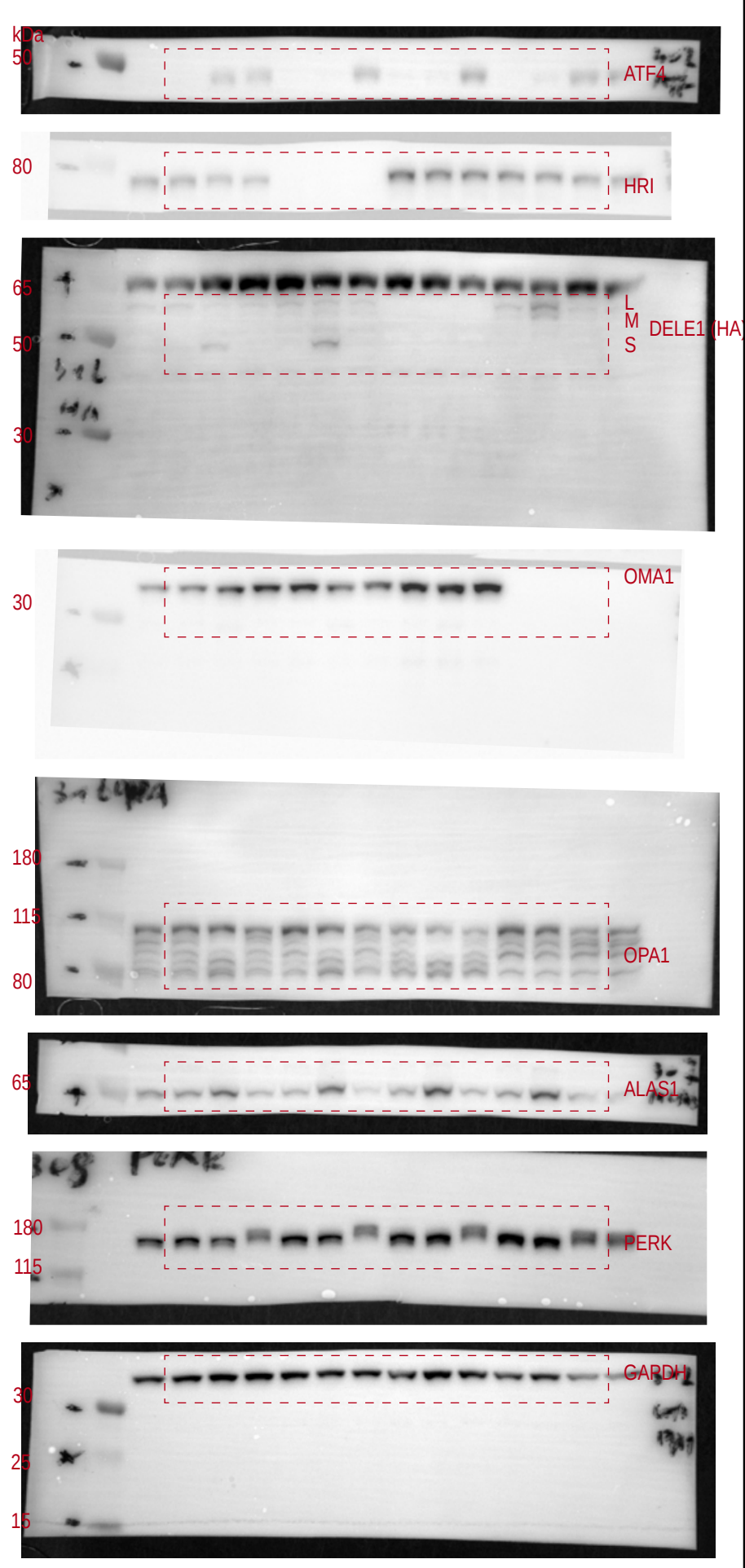

Figure 2a

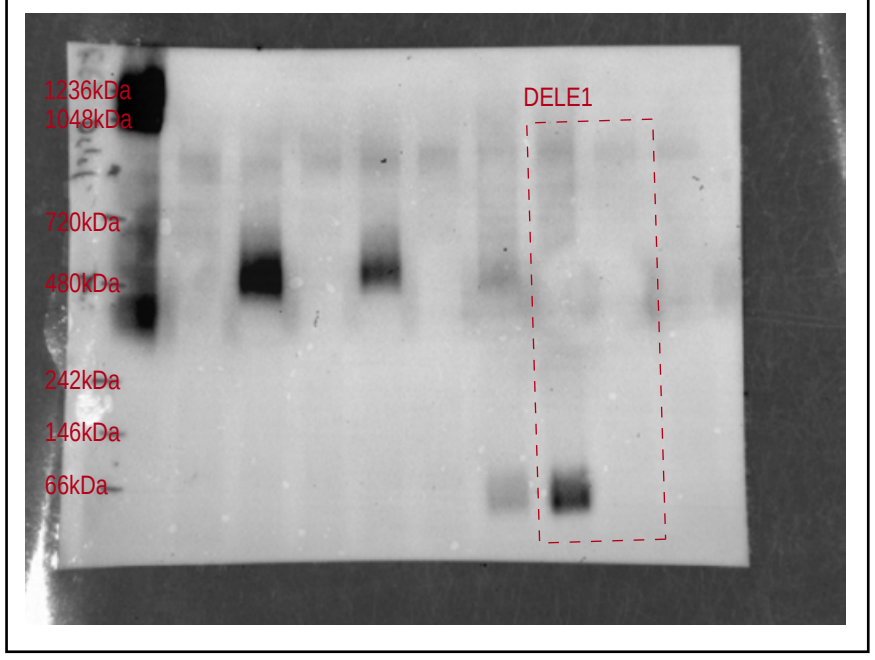

Figure 2b

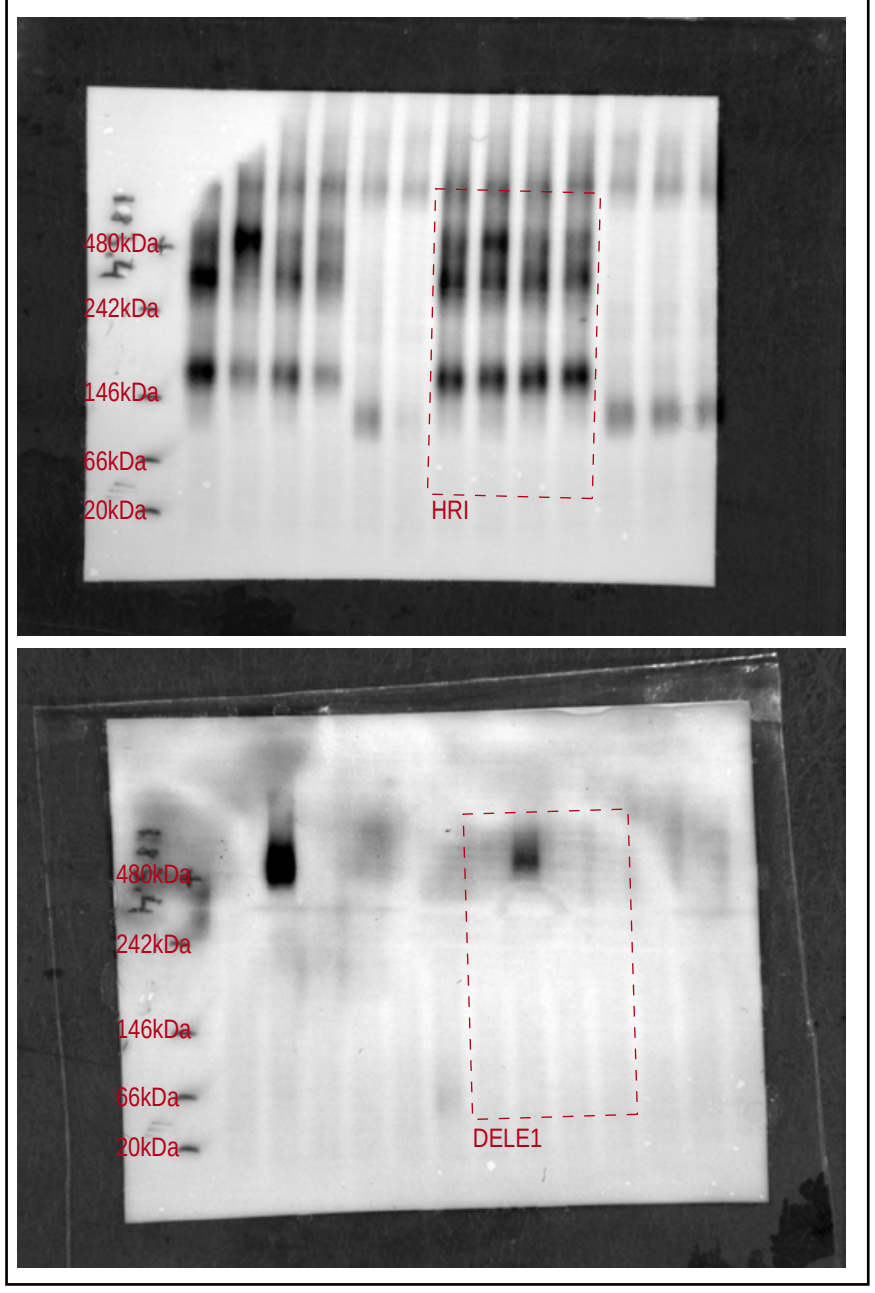

Figure 2e

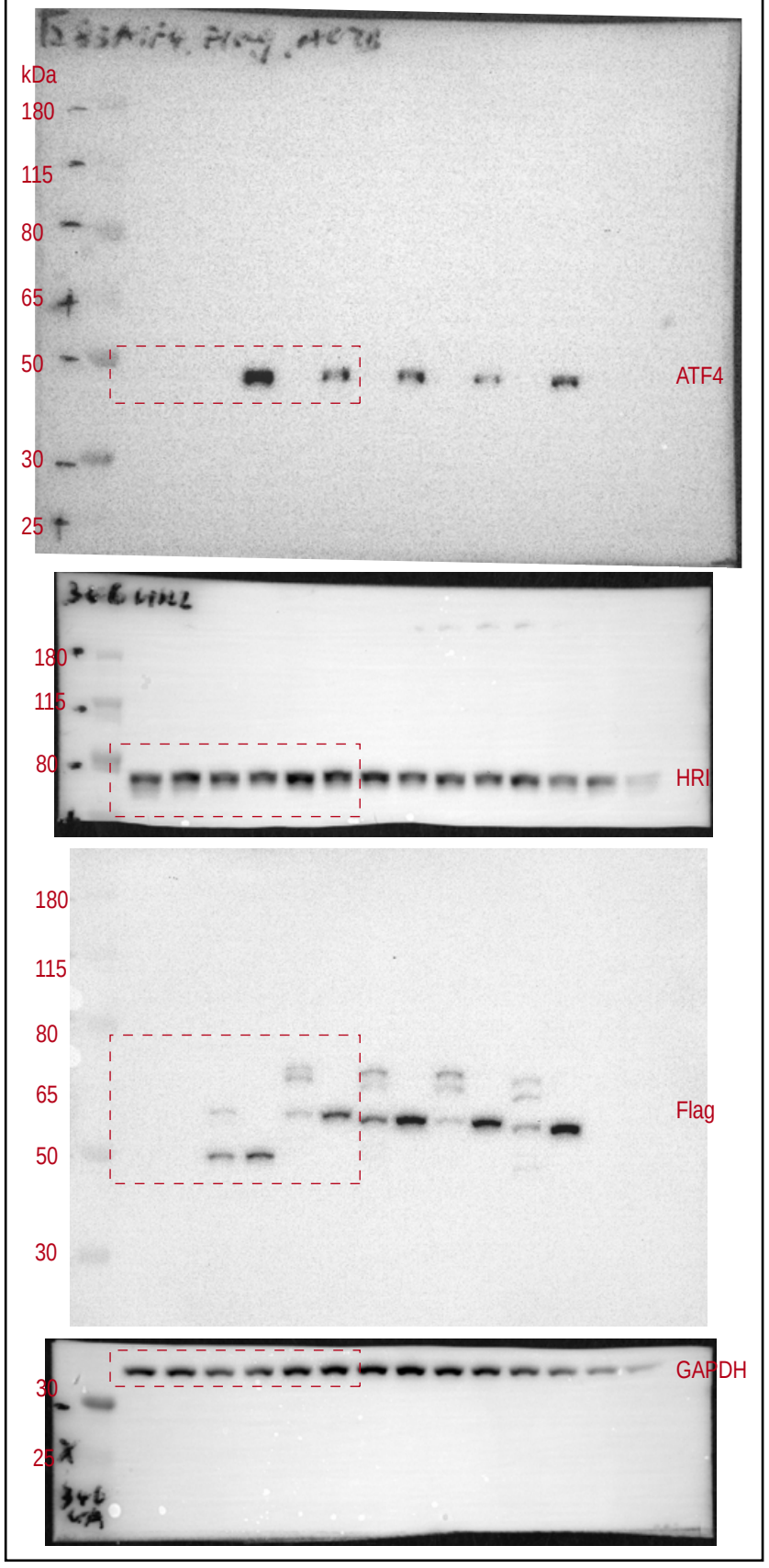

Figure 2d

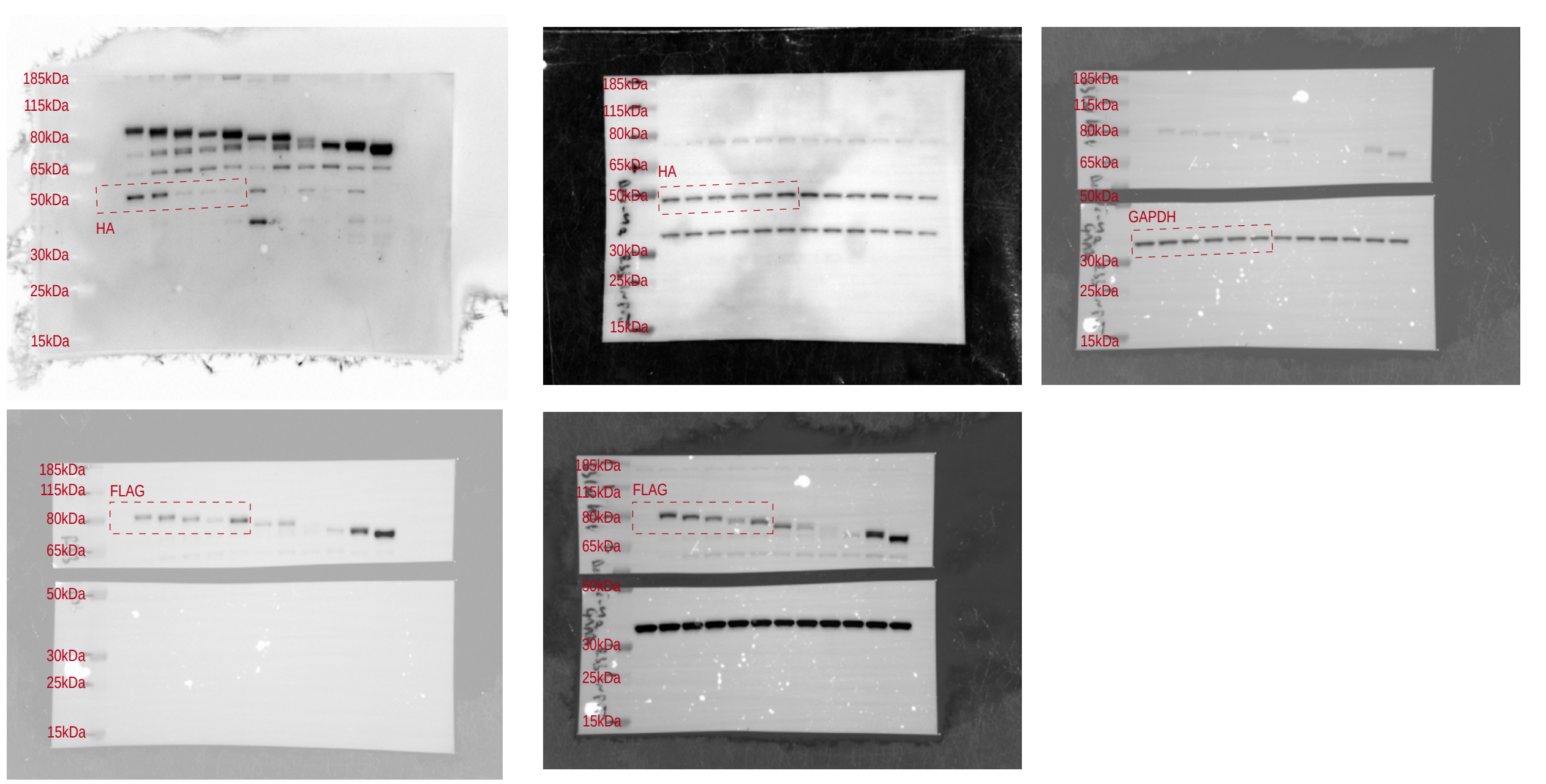

Figure 3c

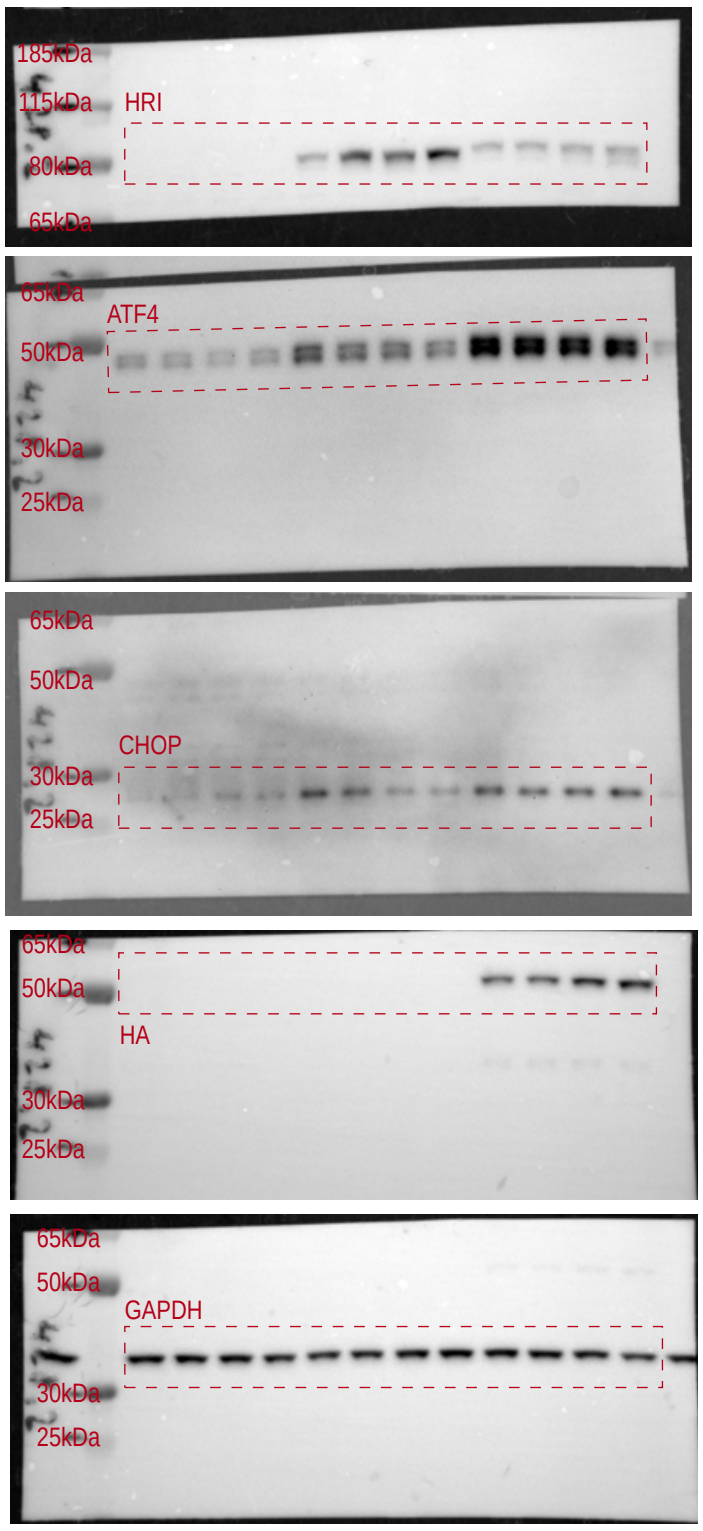

Figure 3e

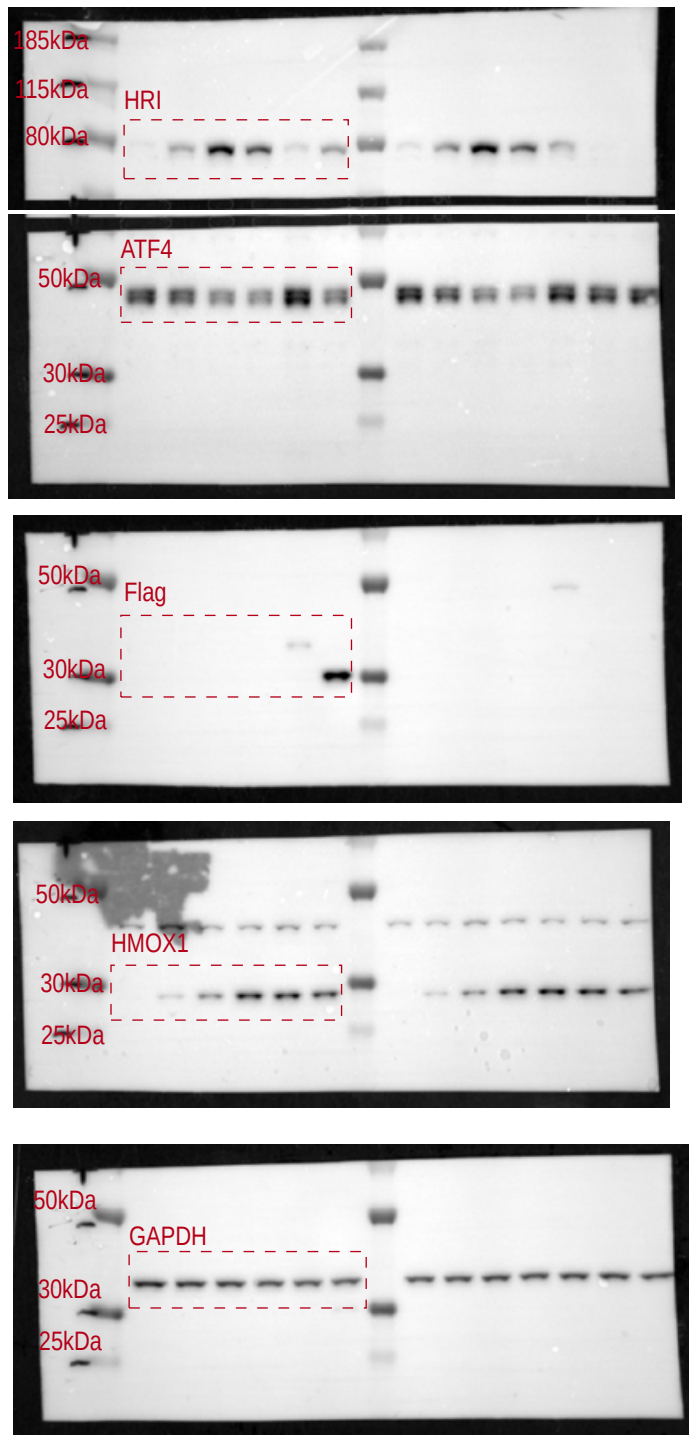

Figure 4a

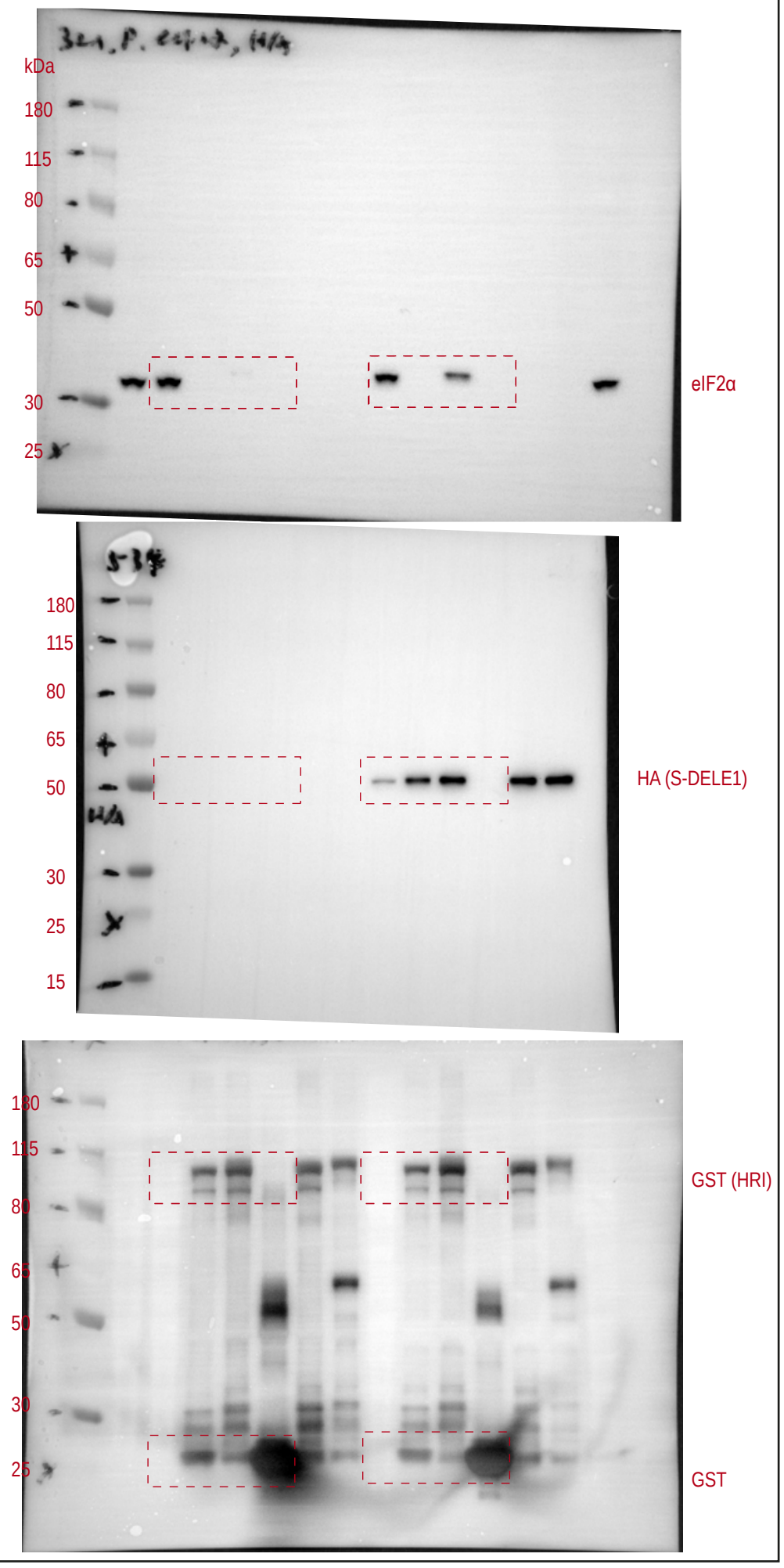

Figure 5a

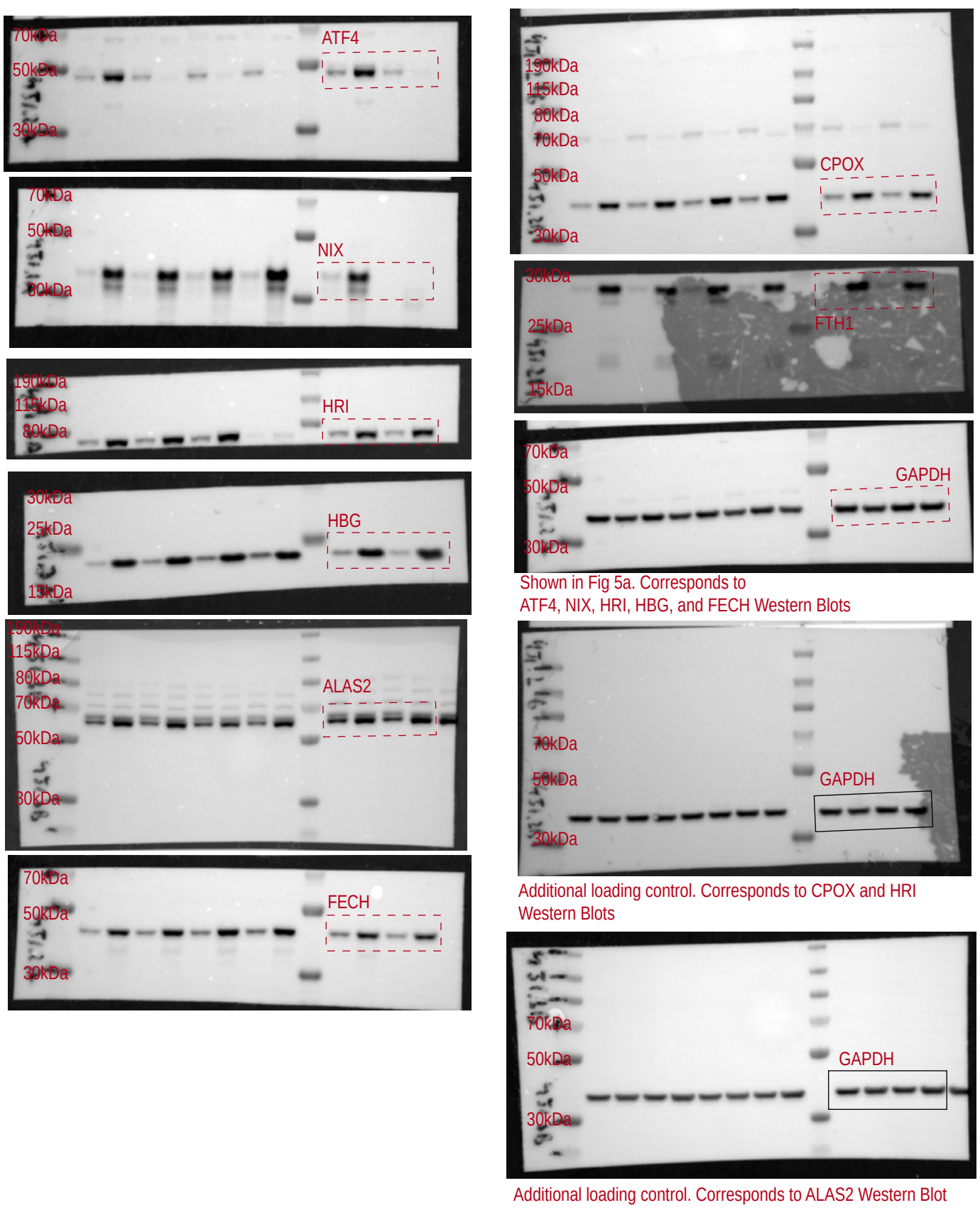

Figure 5b

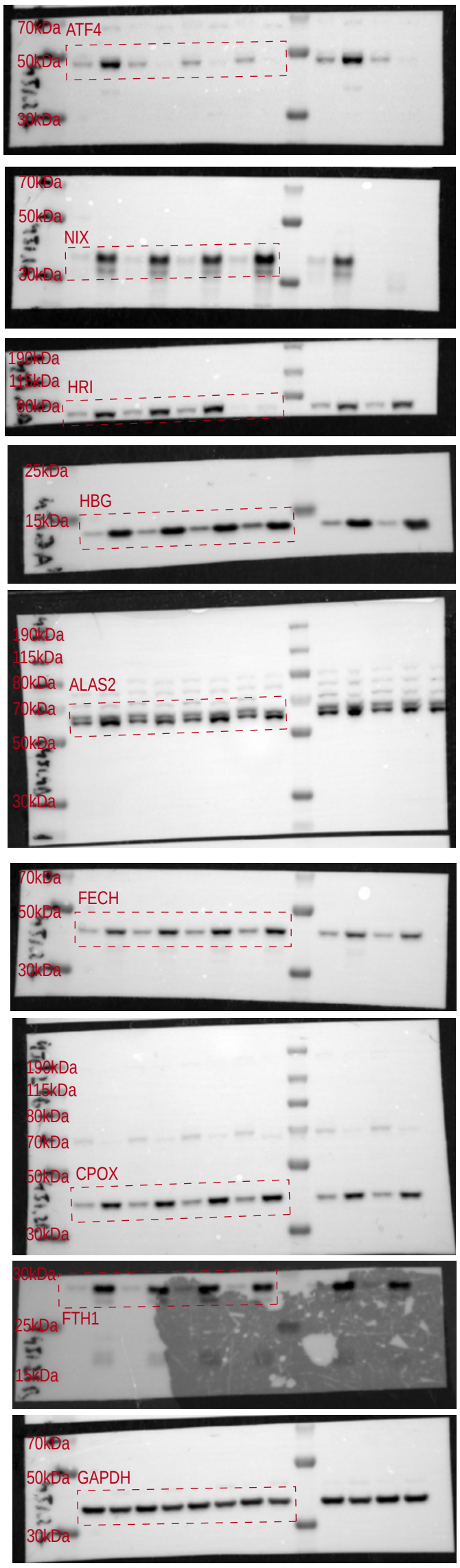

Shown in Fig 5a. Corresponds to ATF4, NIX, HRI, HBG, and FECH Western Blots

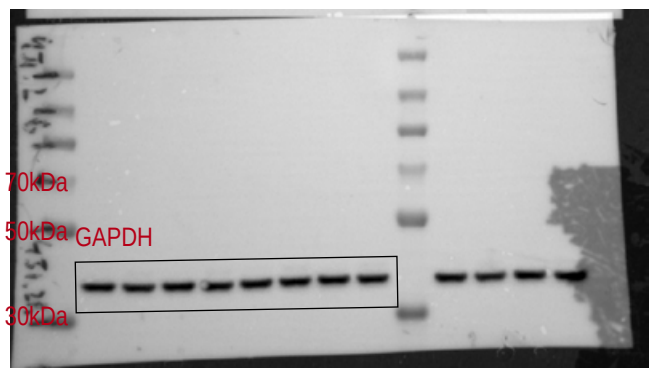

Additional loading control. Corresponds to CPOX and HRI Western Blot

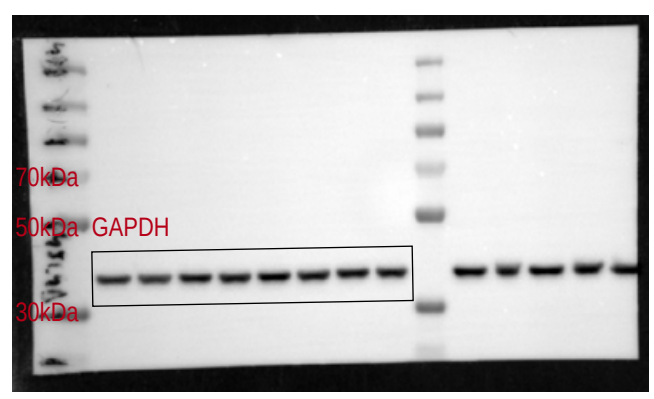

Additional loading control. Corresponds to ALAS2 Western Blot

Figure 5e

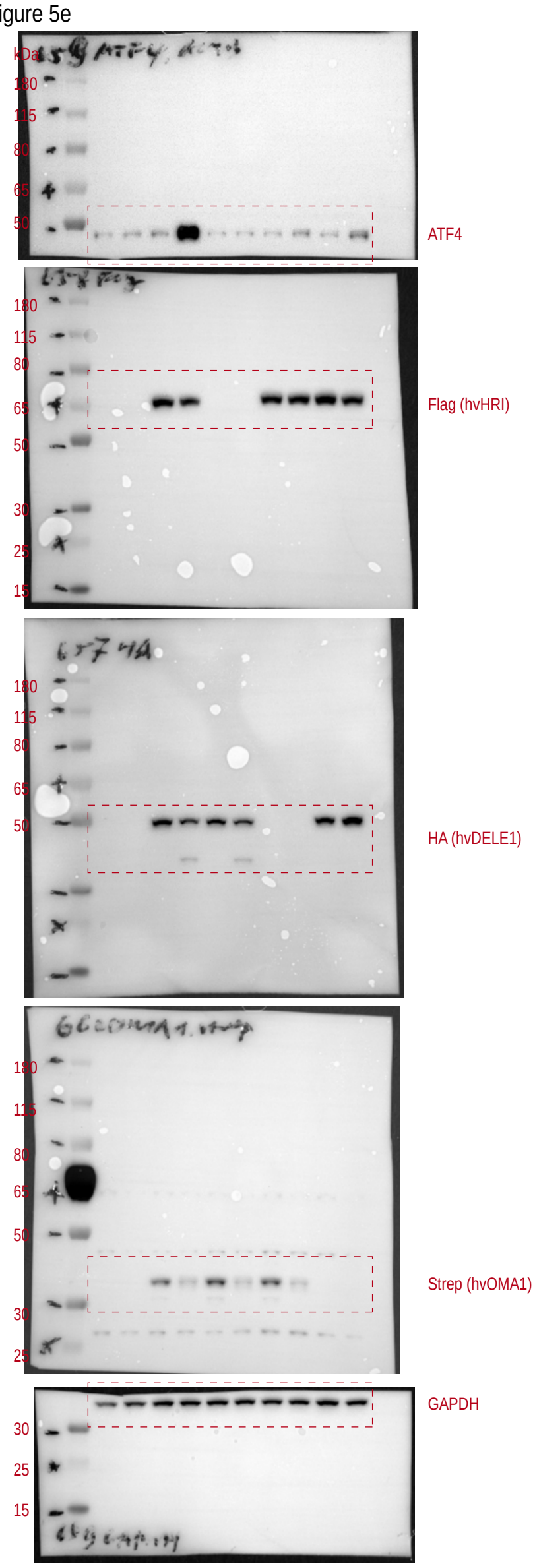

Extended Data Figure 1d

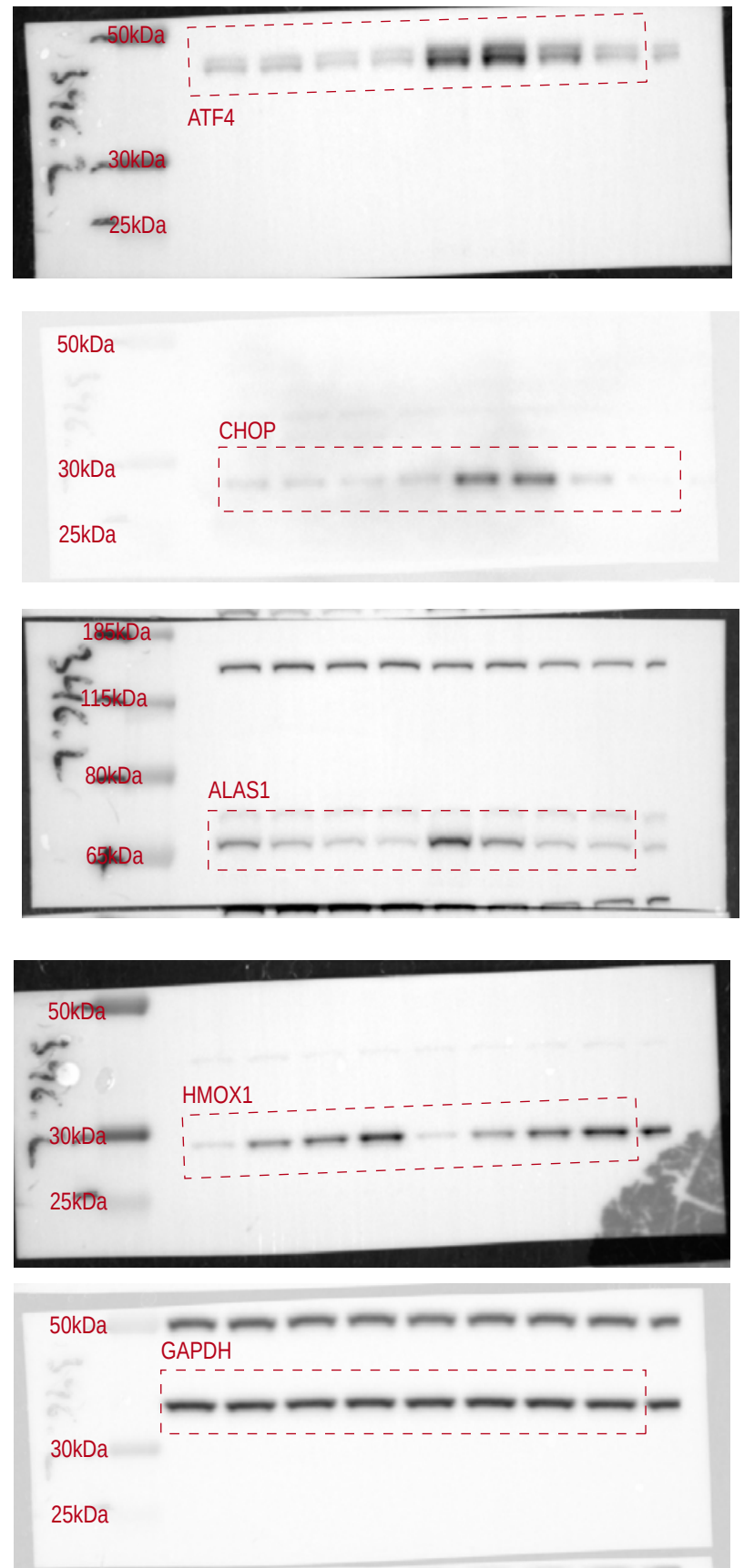

Extended Data Figure 1e

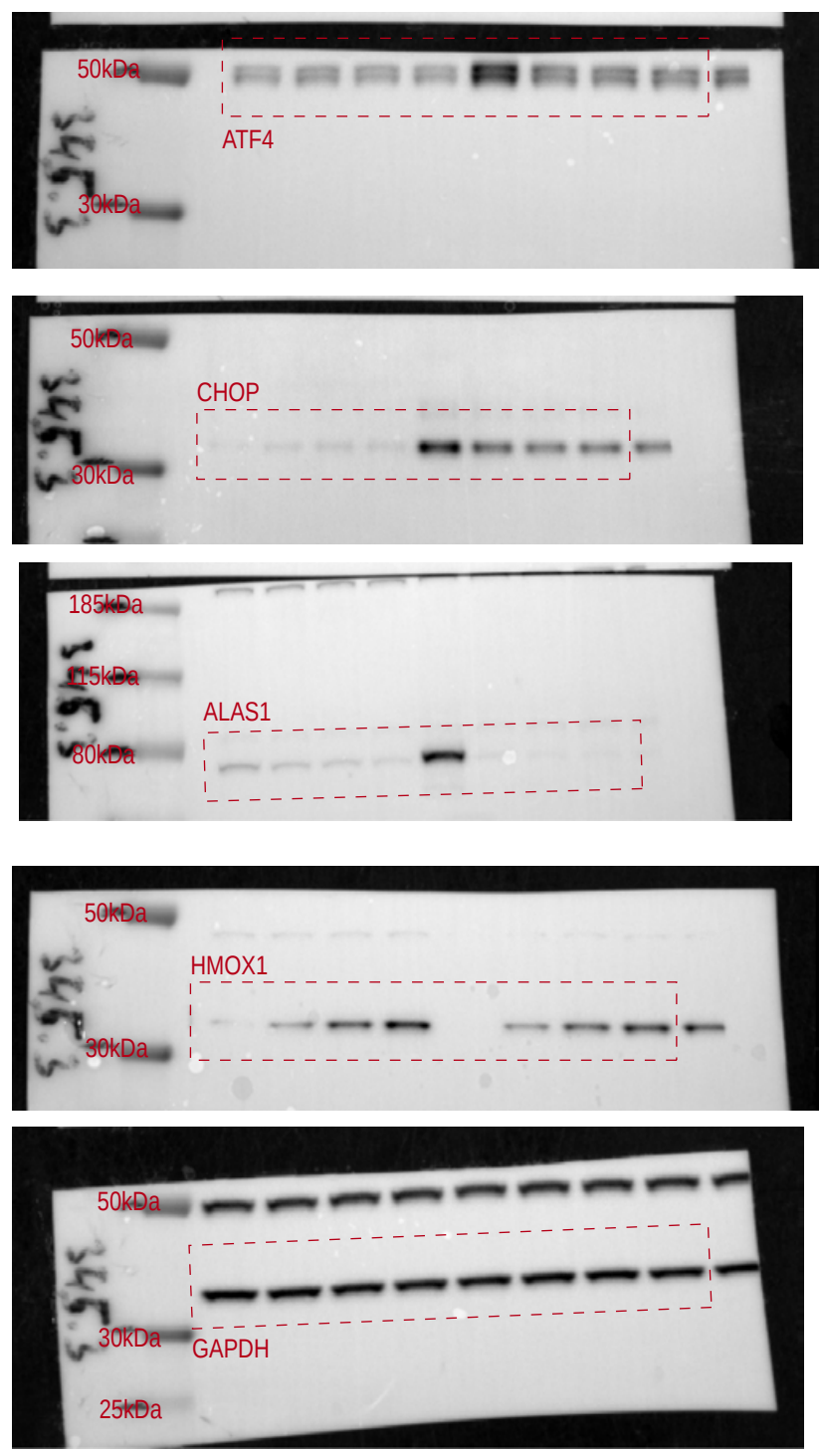

Extended Data Figure 1h

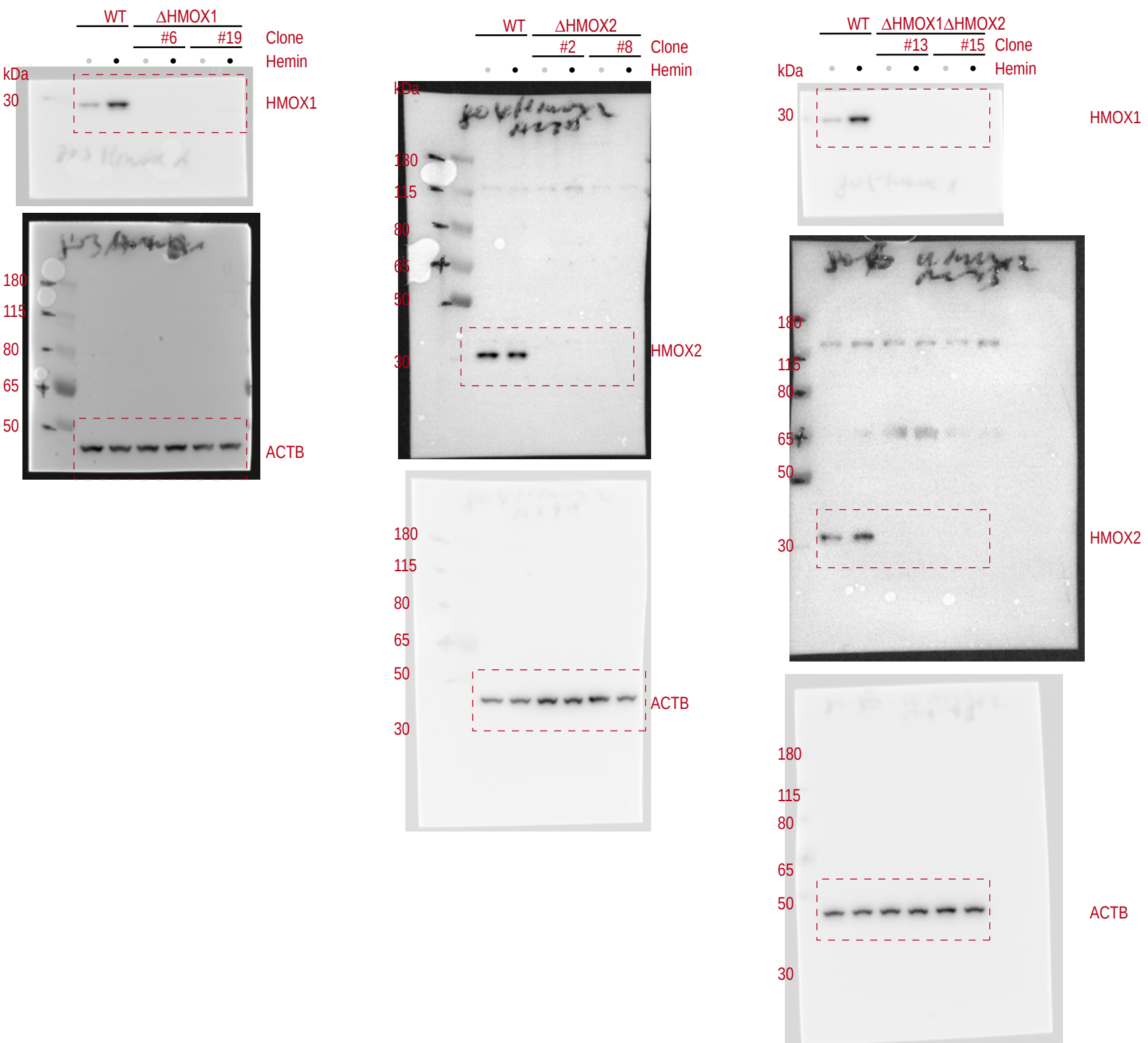

Extended Data Figure 1f

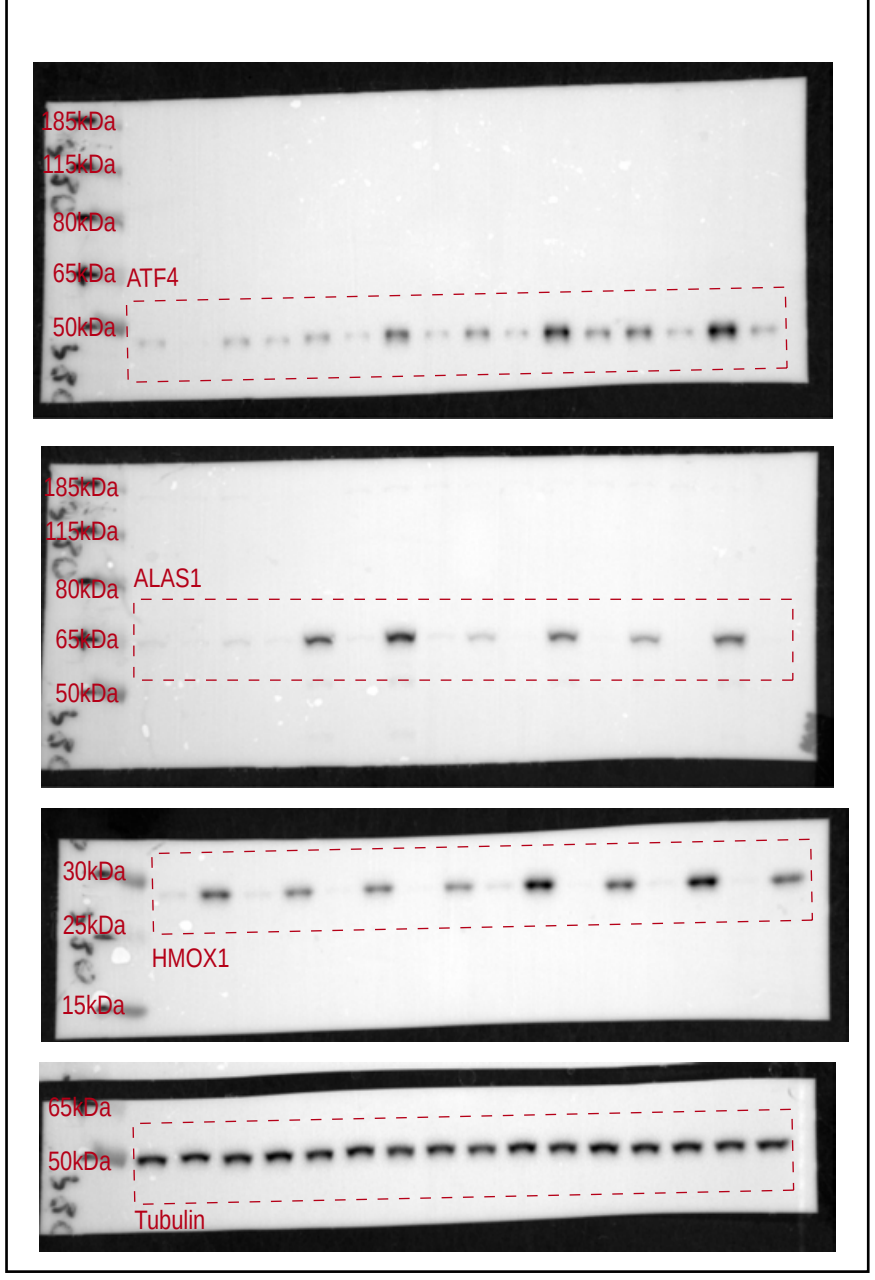

Extended Data Figure 1g

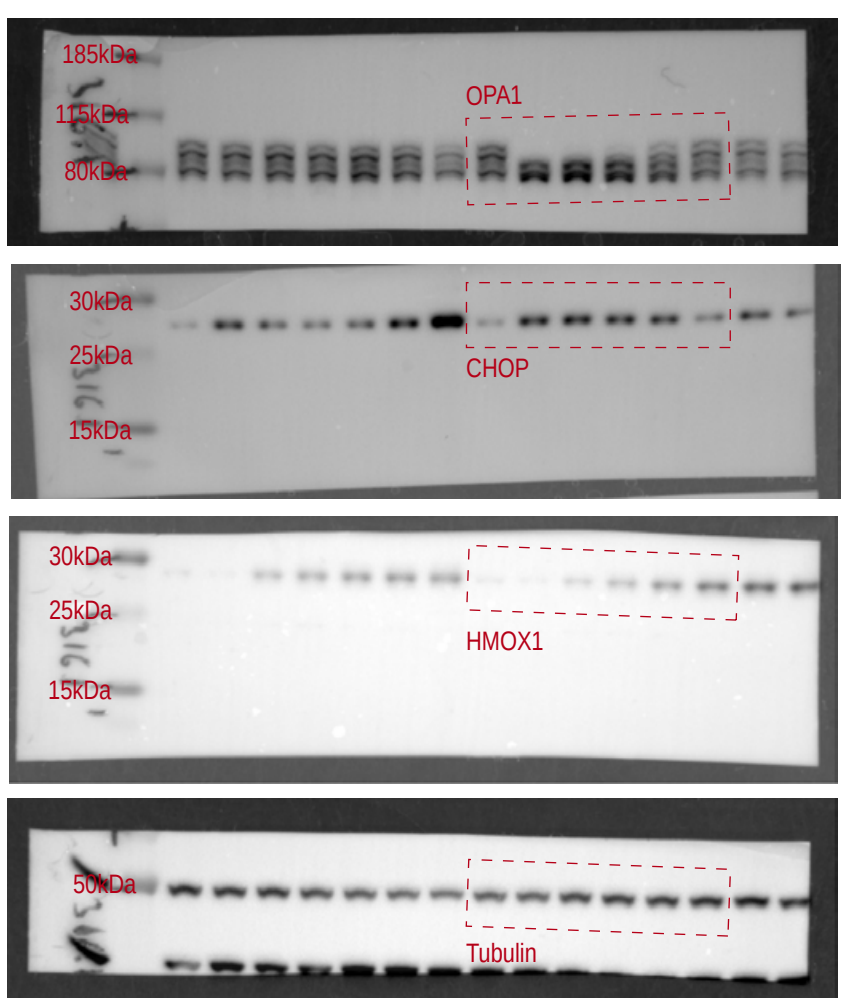

Extended Data Figure 1i

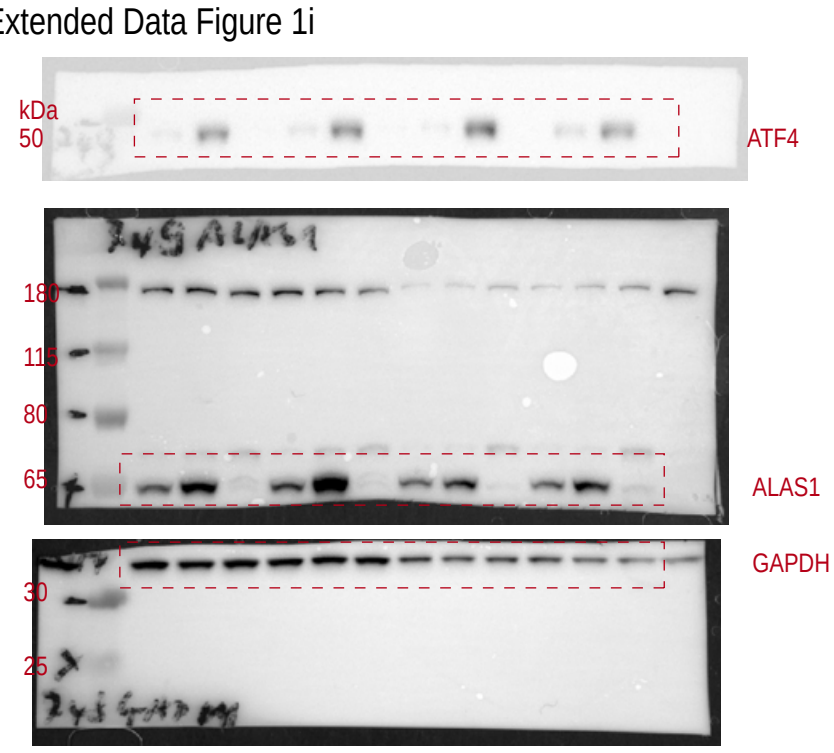

Extended Data Figure 1j

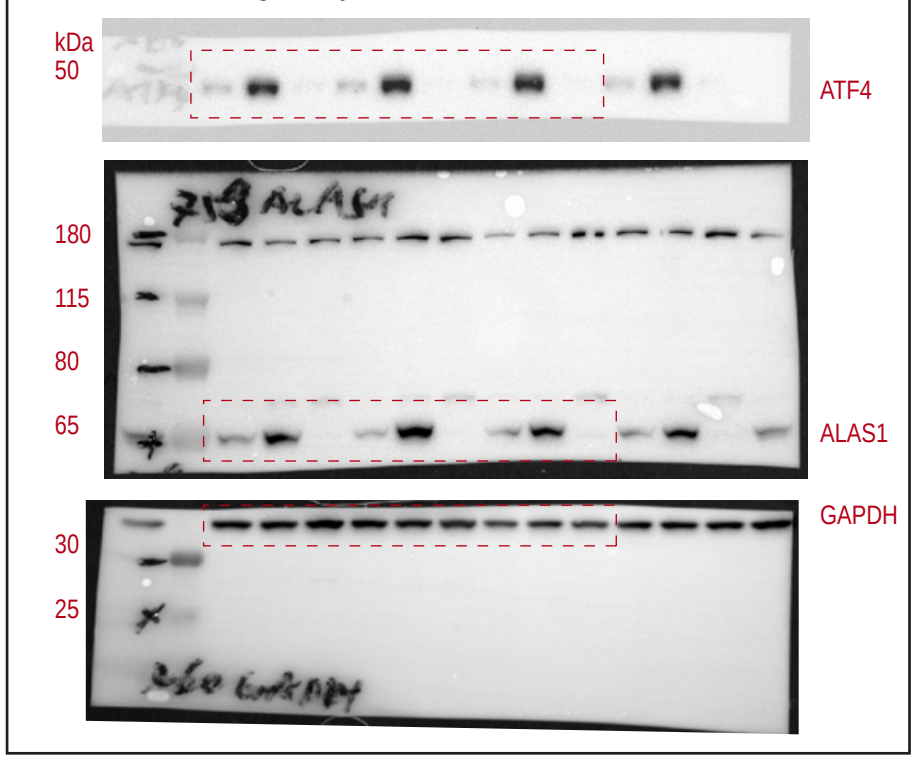

Extended Data Figure 1k

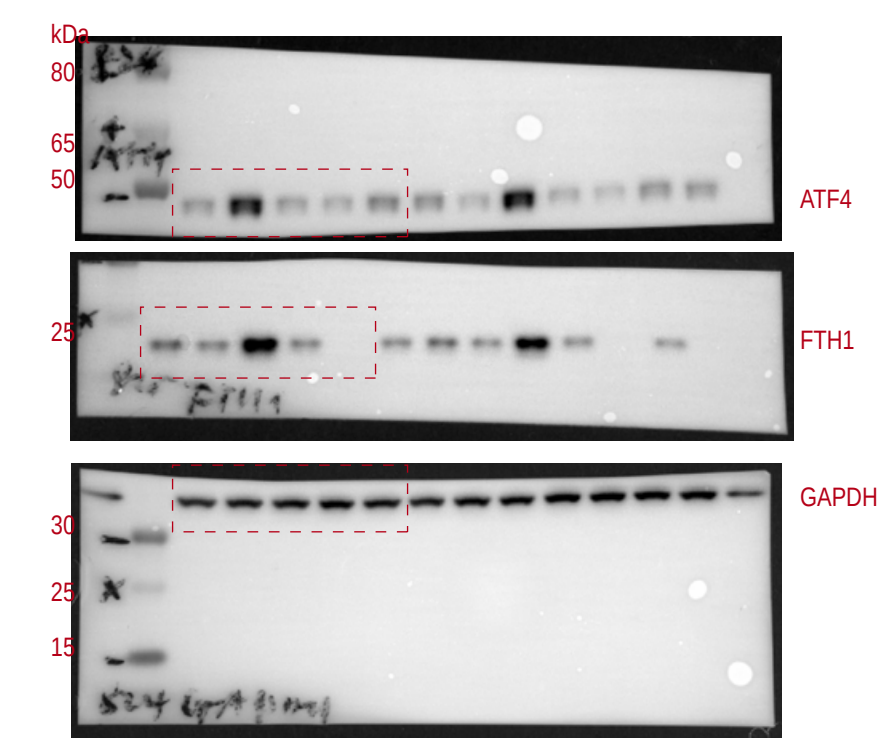

Extended Data Figure 1l

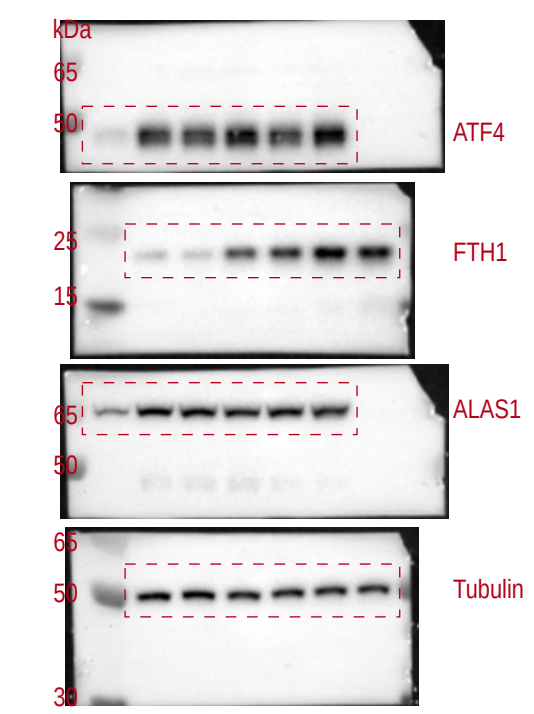

Extended Data Figure 1m

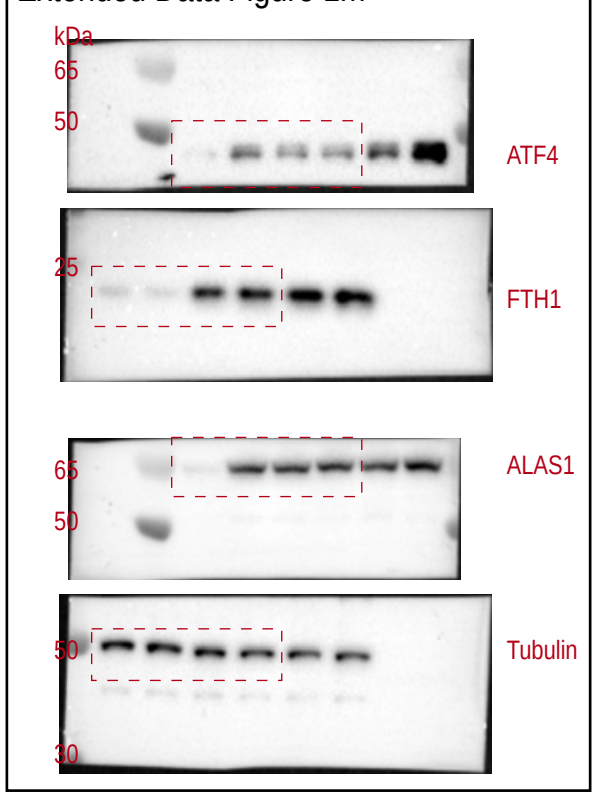

Extended Data Figure 2d

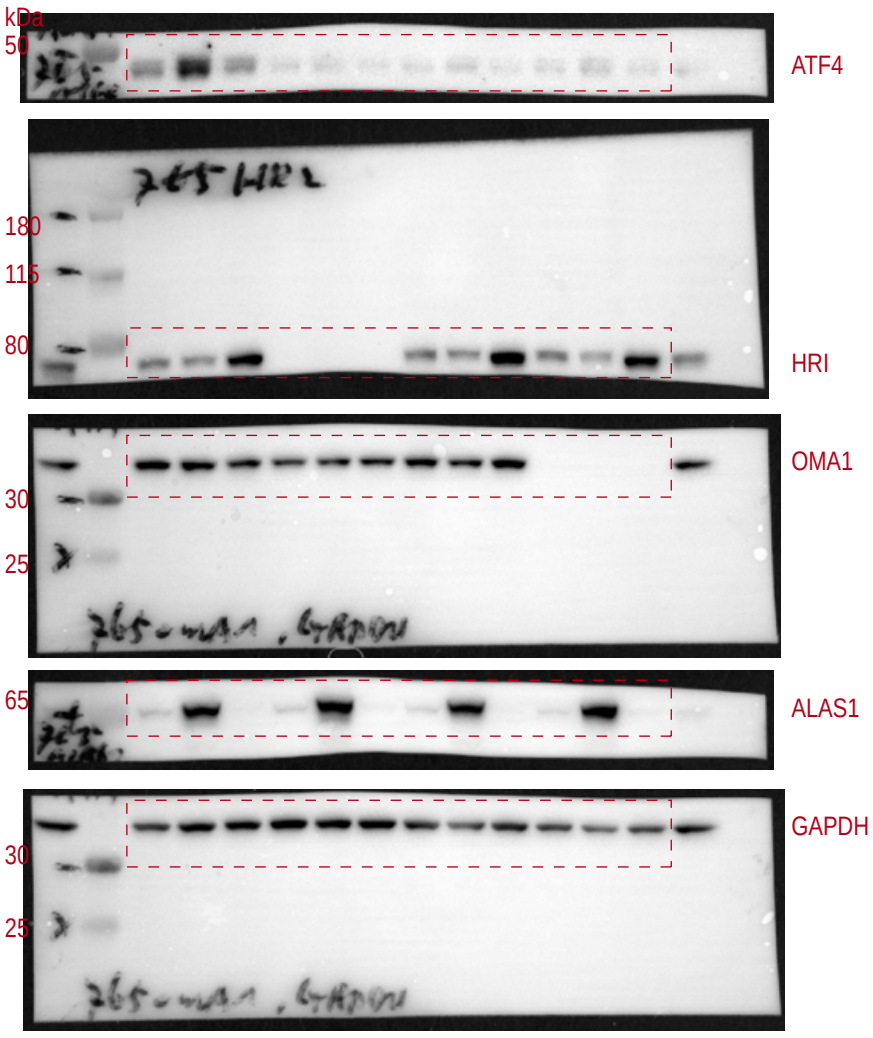

Extended Data Figure 2e

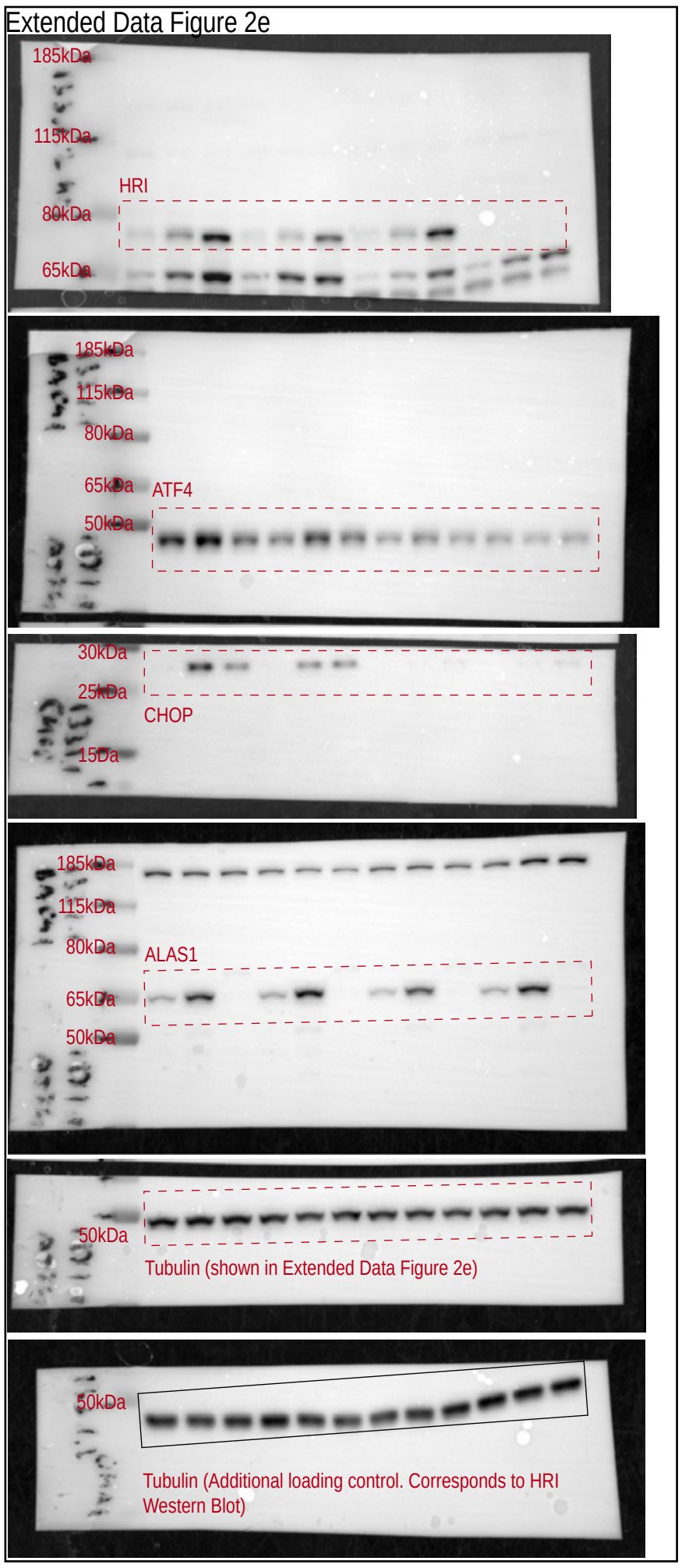

Extended Data Figure 2f

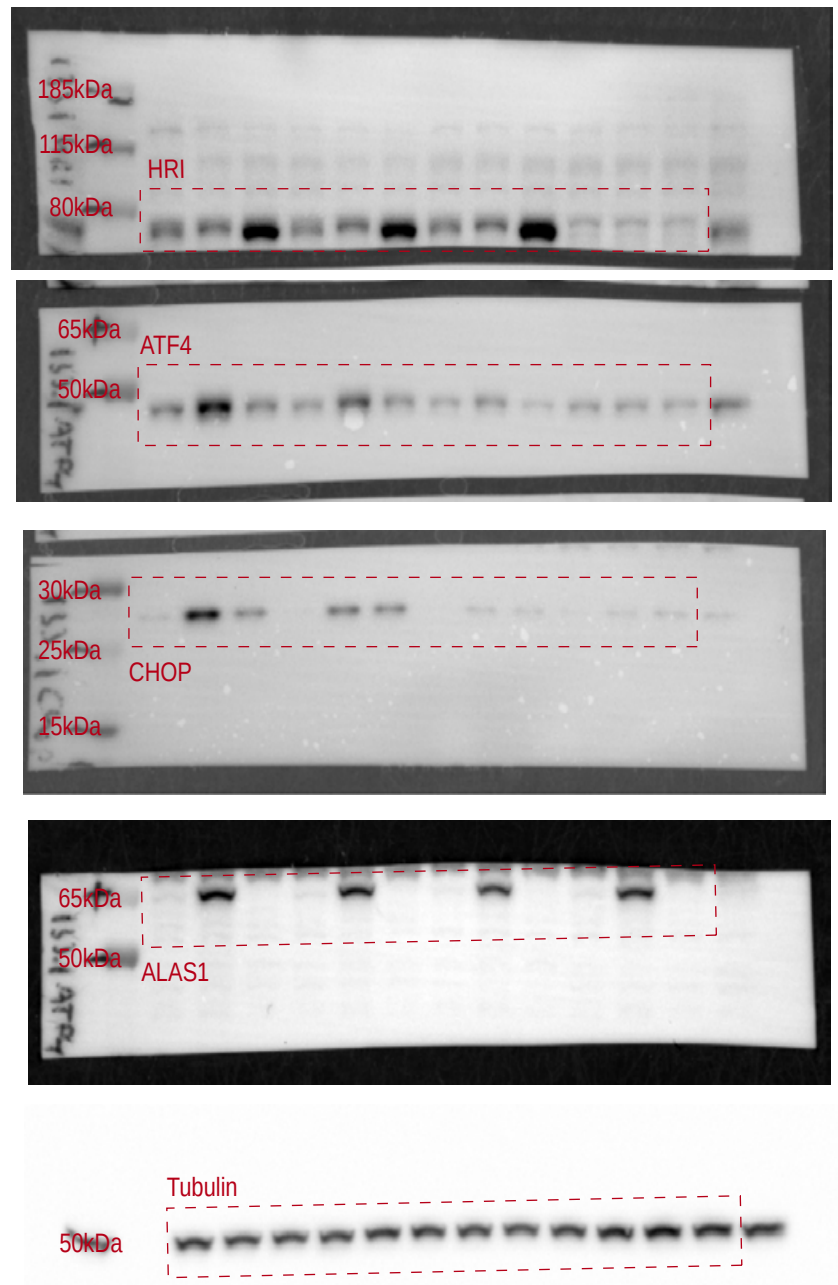

Extended Data Figure 3g

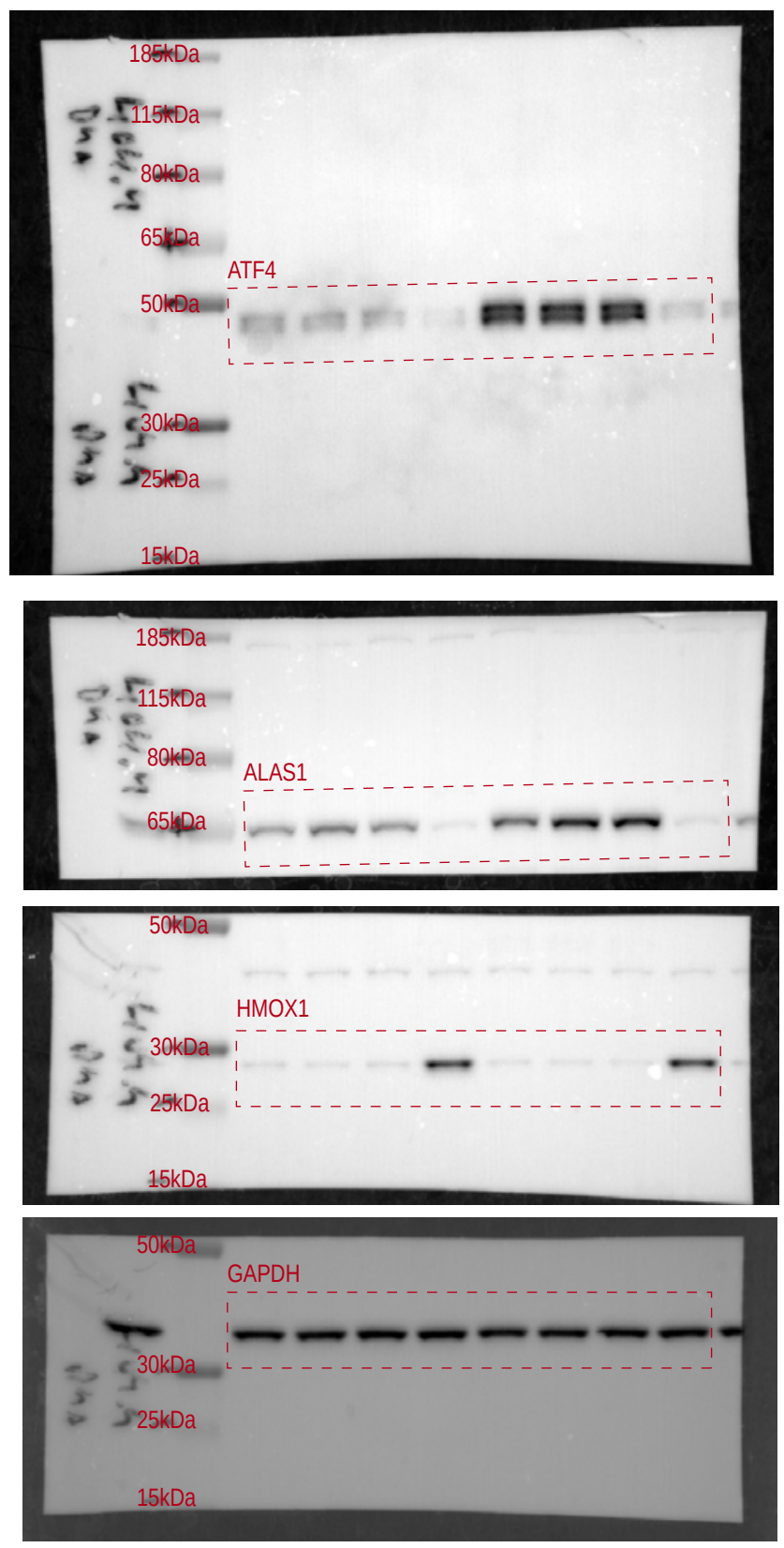

Extended Data Figure 3h

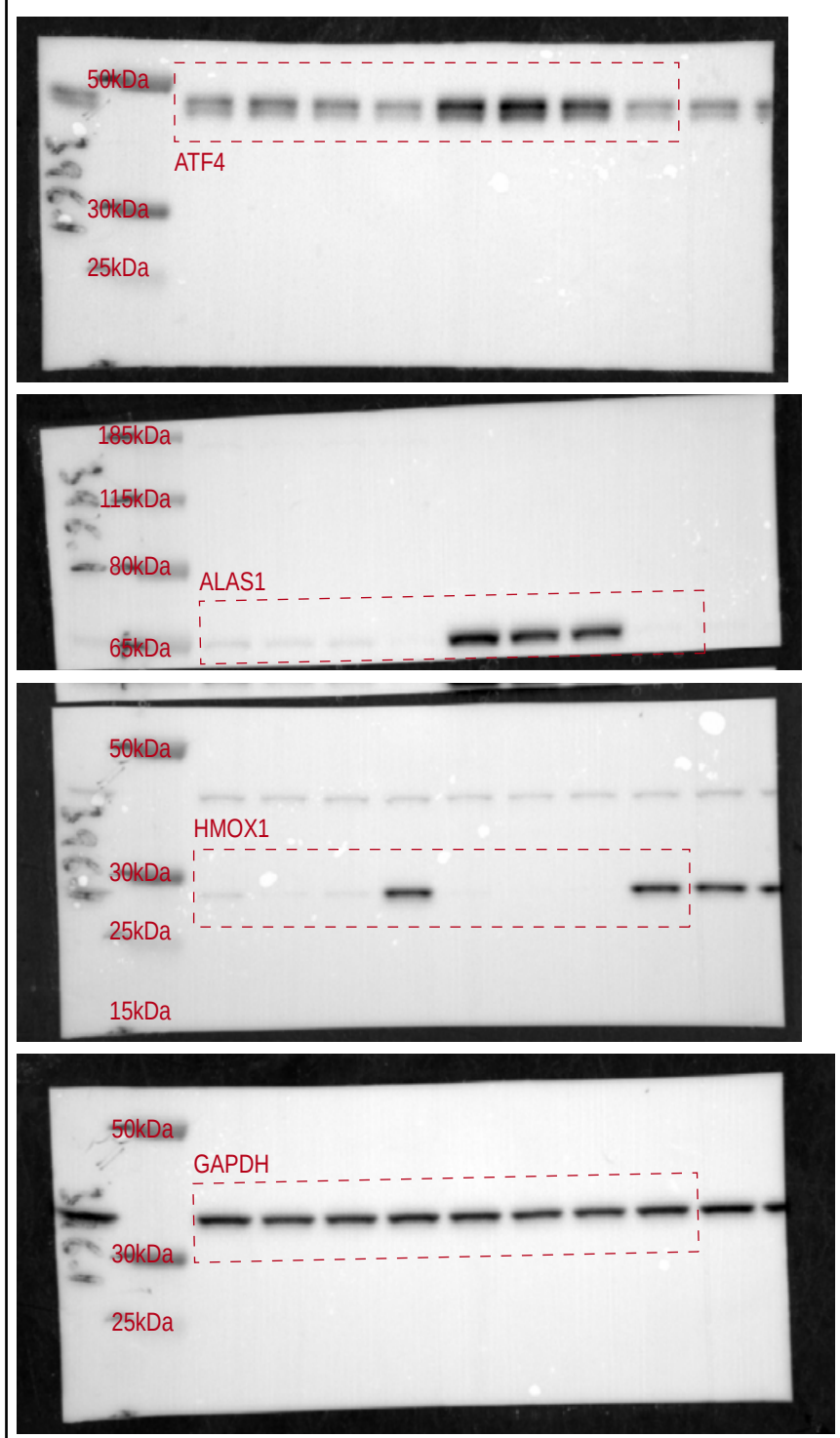

Extended Data Figure 3i

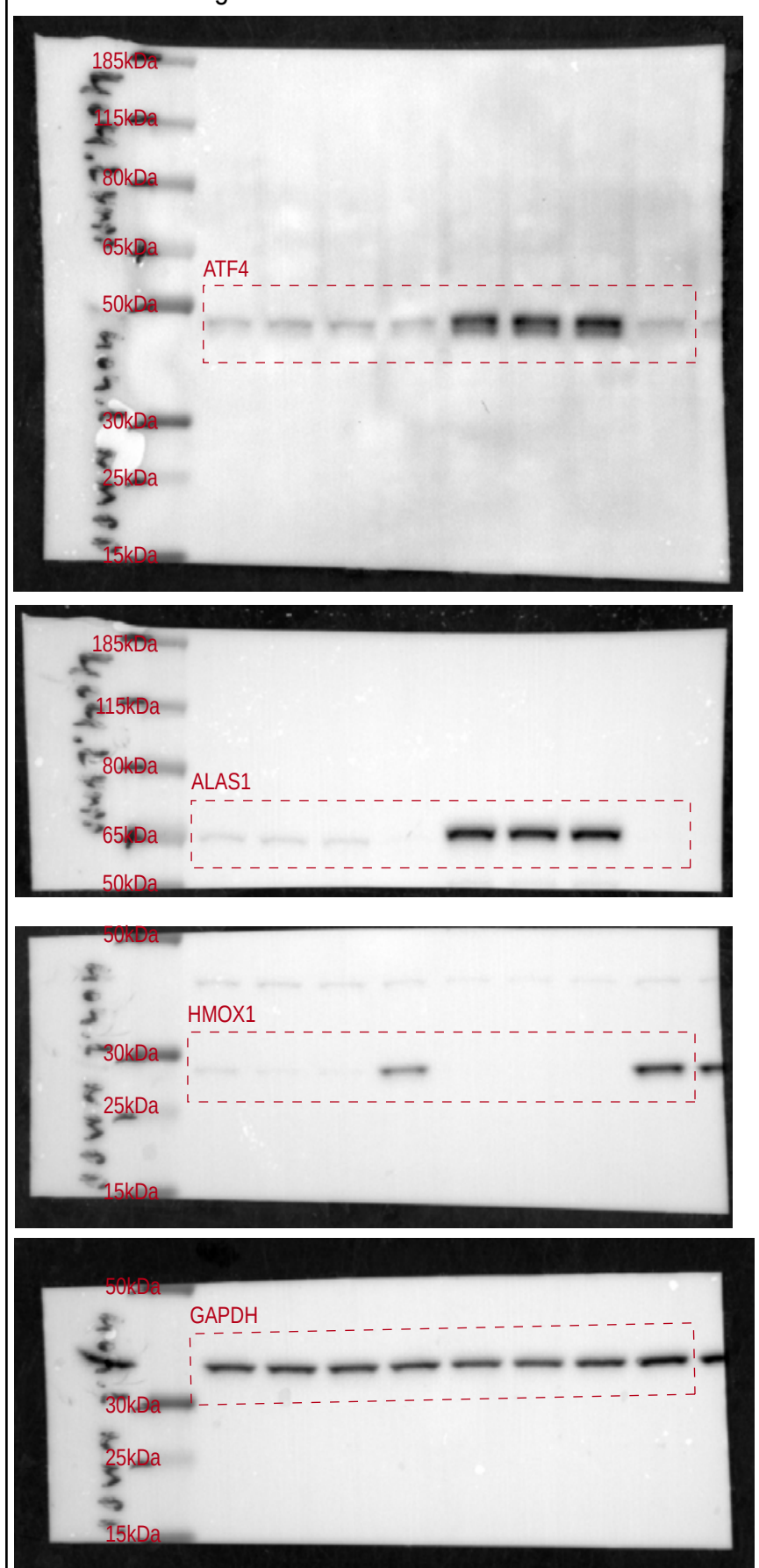

Extended Data Figure 3j

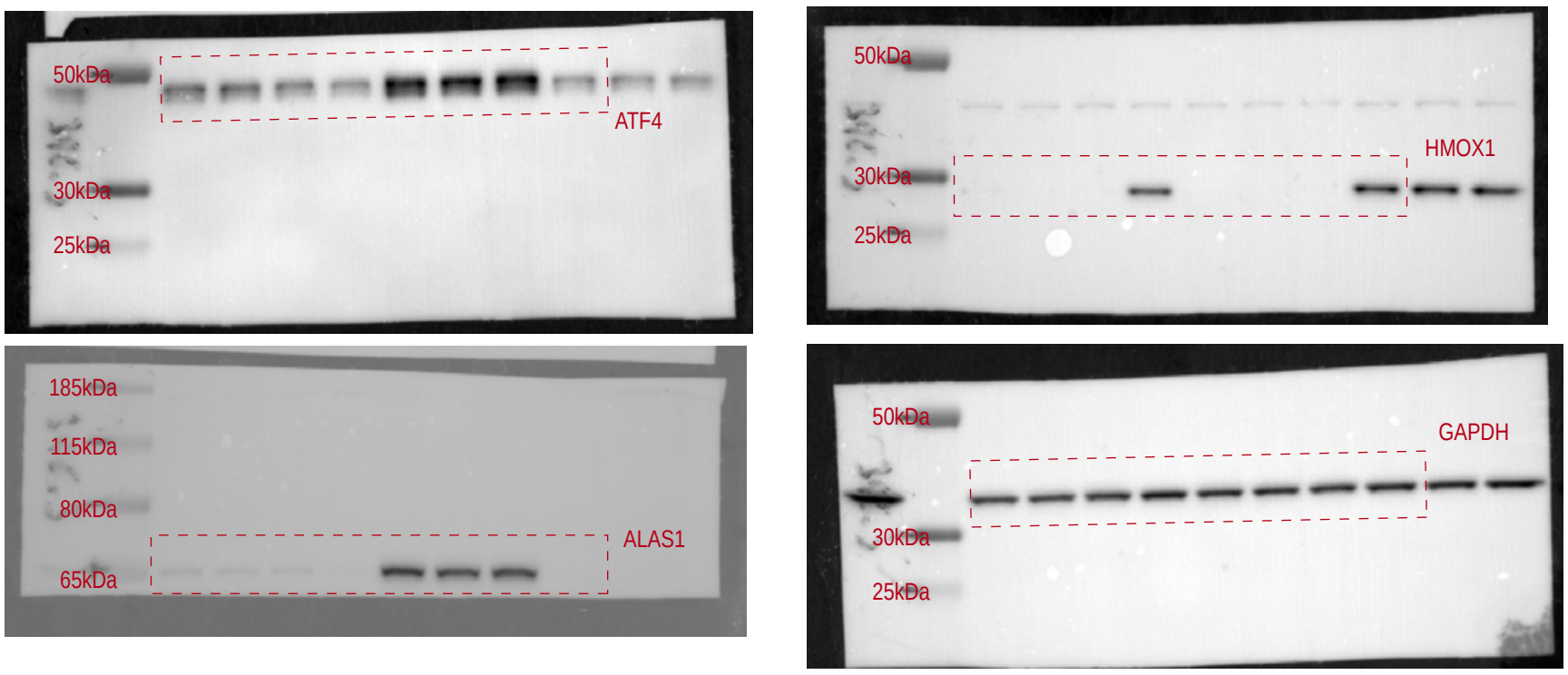

Extended Data Figure 3k

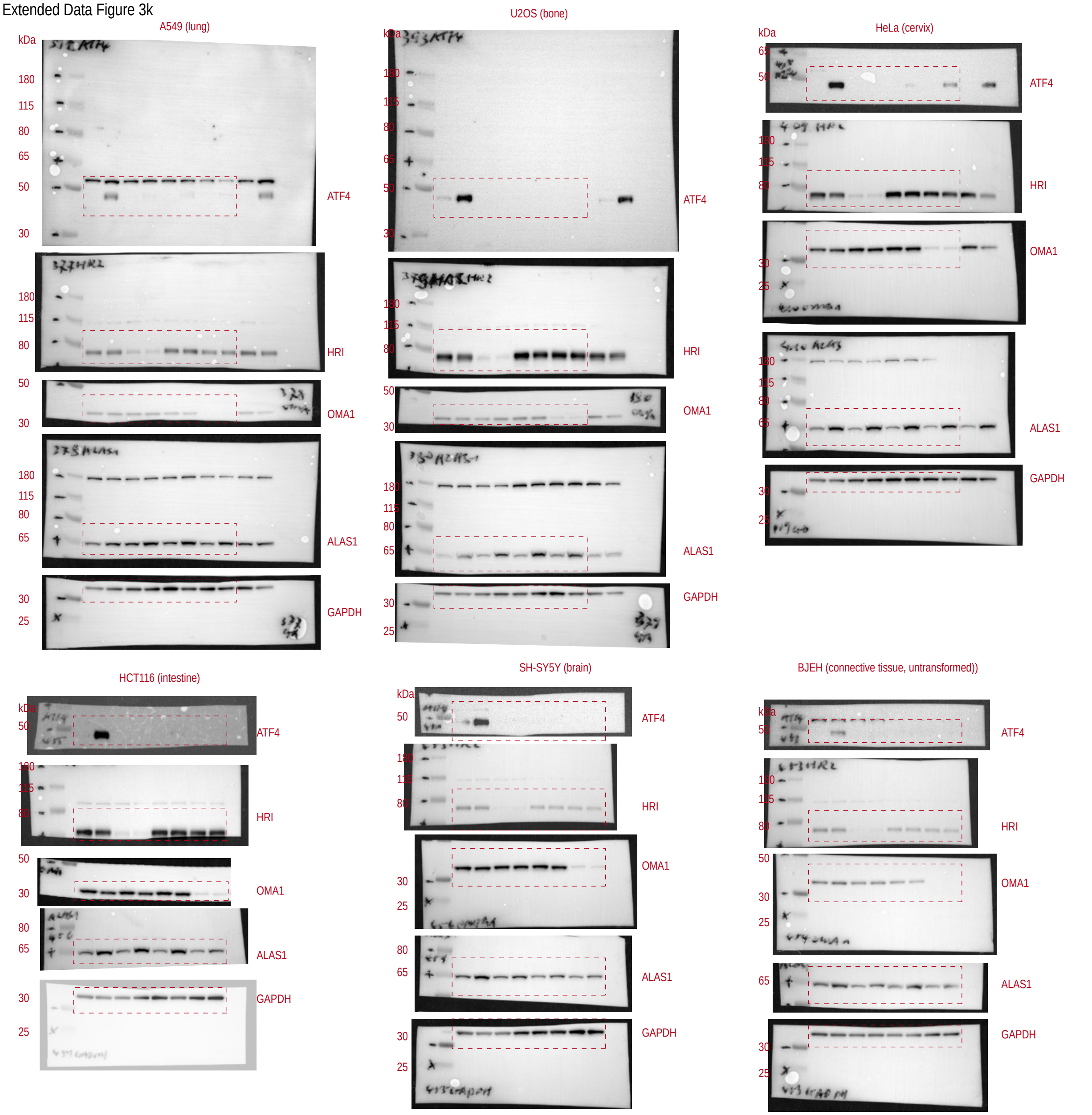

Extended Data Figure 3l

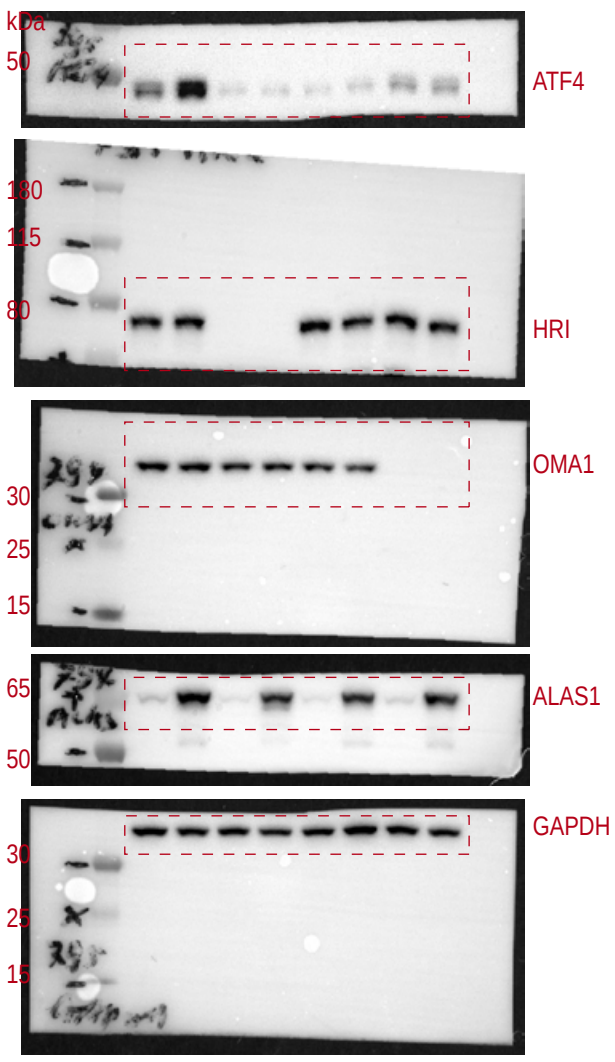

Extended Data Figure 3m

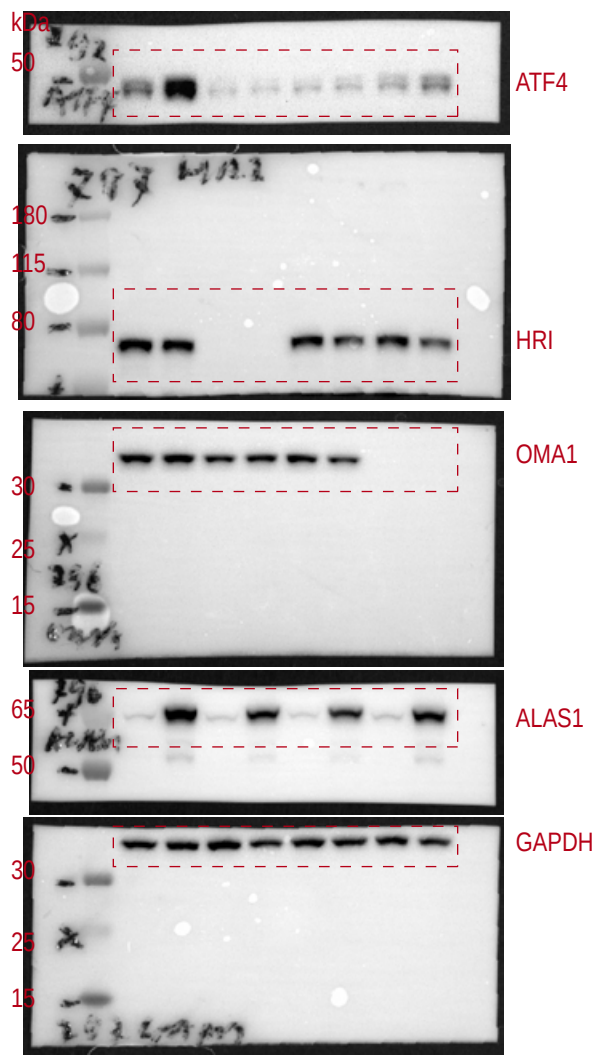

Extended Data Figure 3n

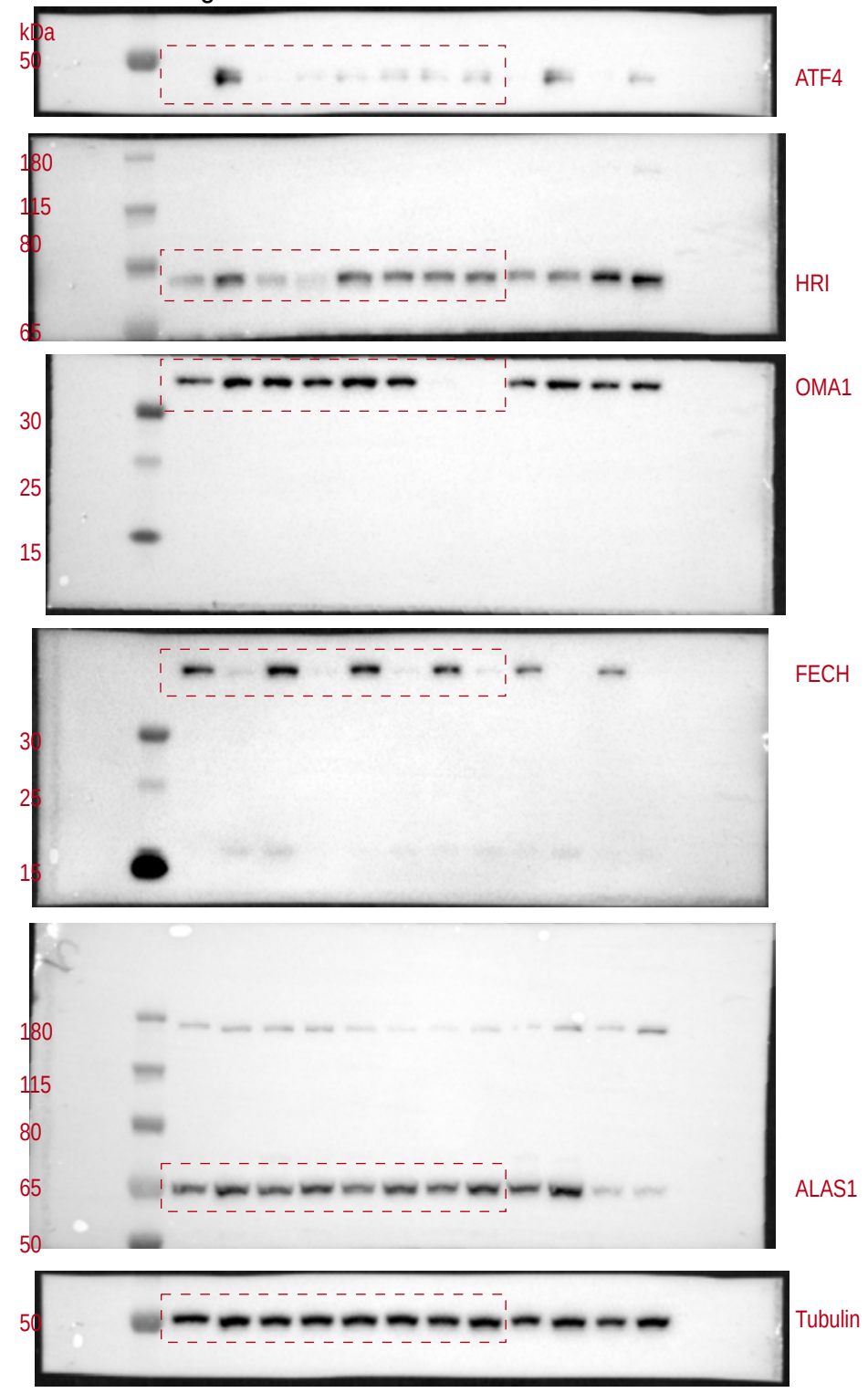

Extended Data Figure 3o

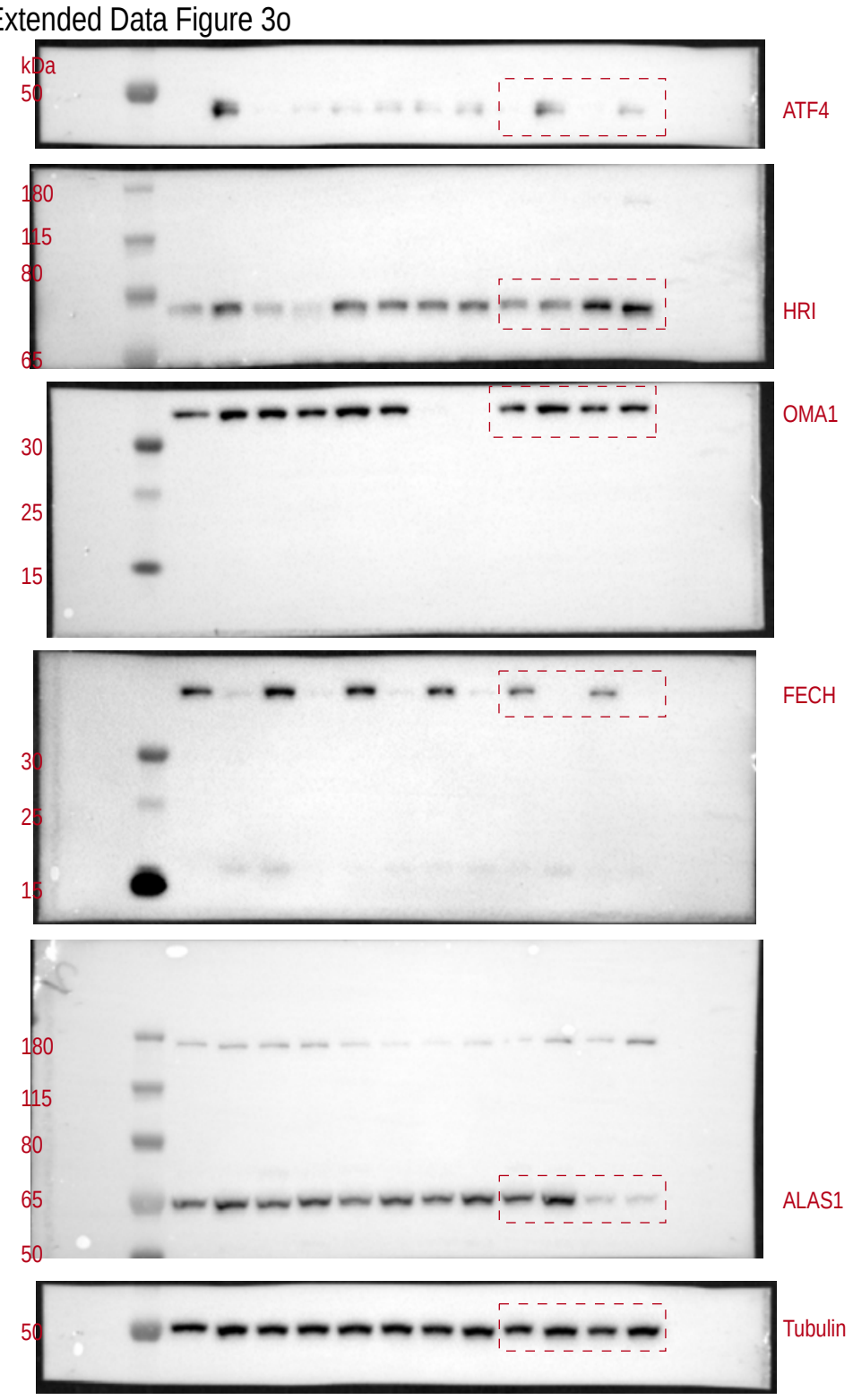

Extended Data Figure 4b

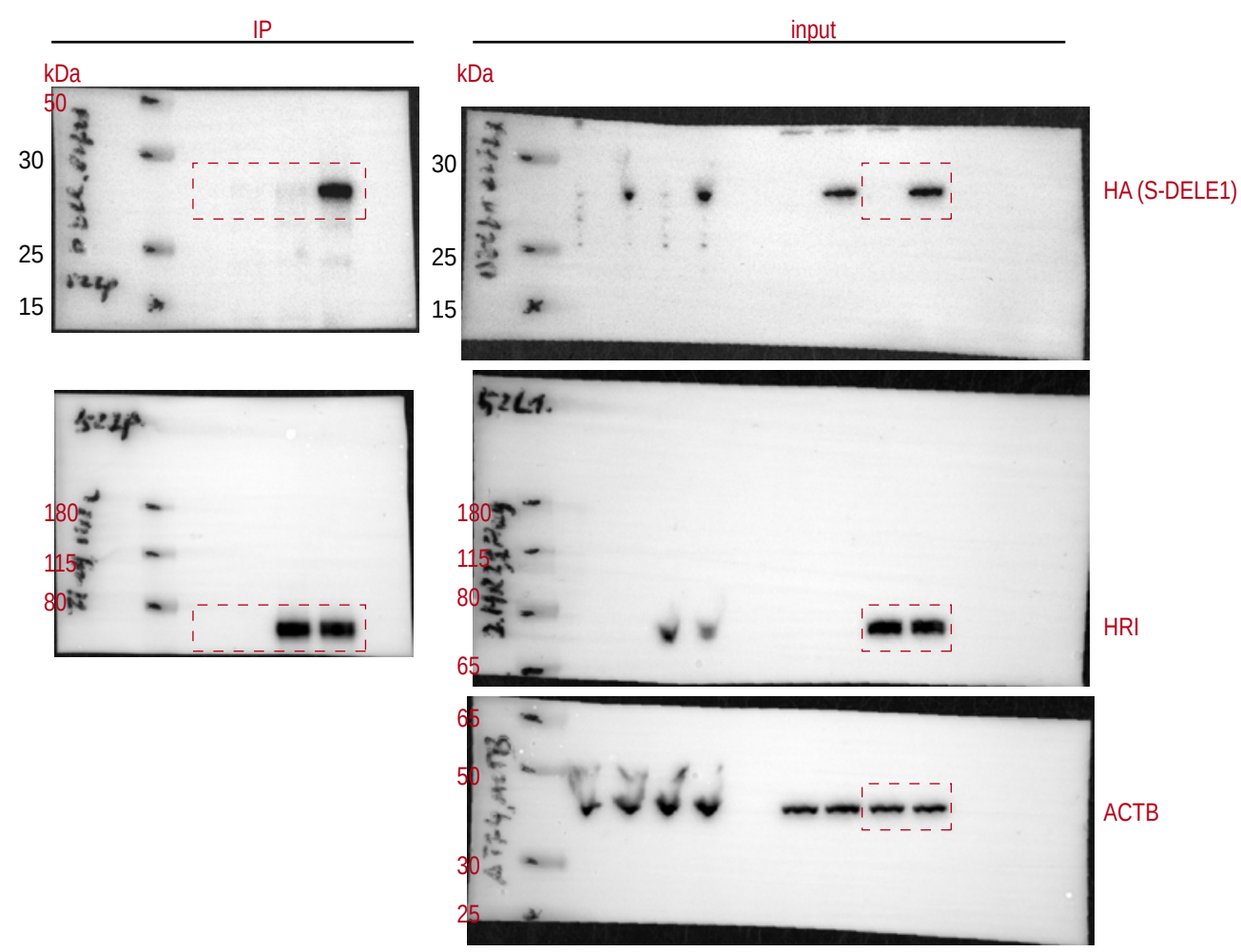

Extended Data Figure 4a

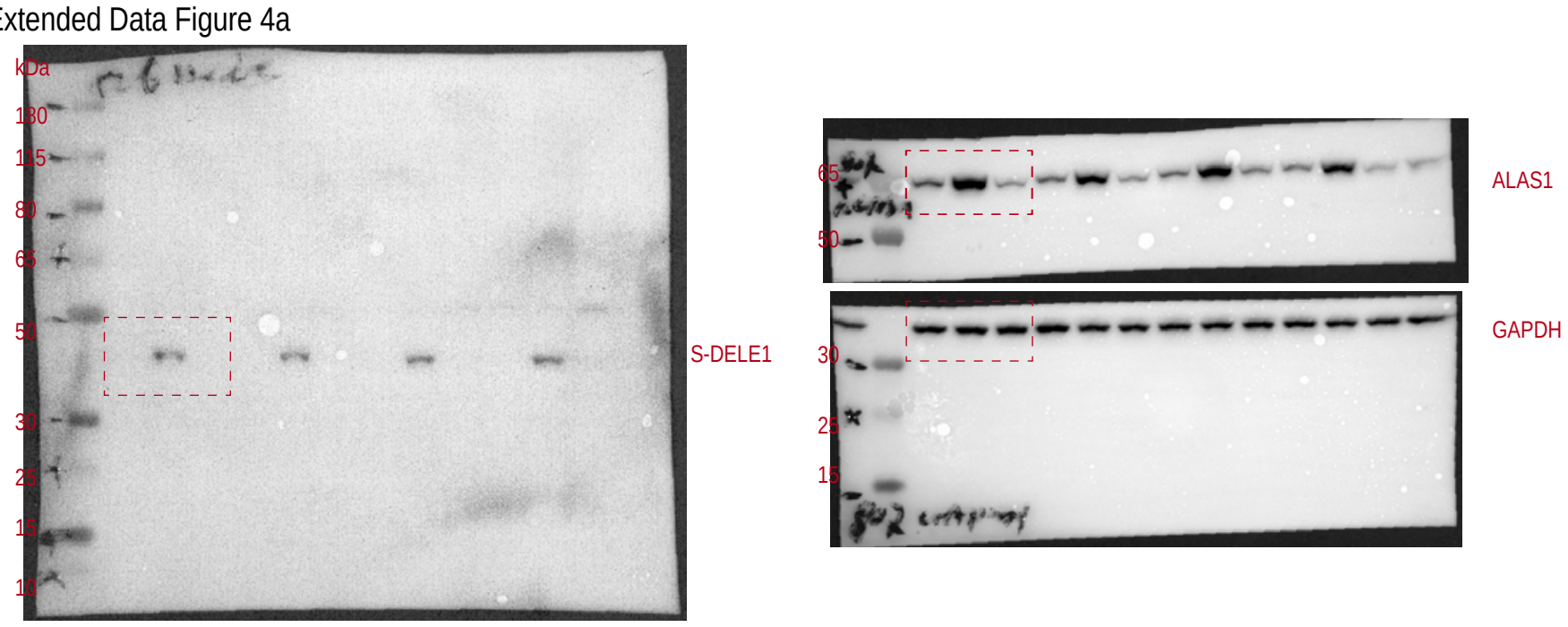

Extended Data Figure 4c

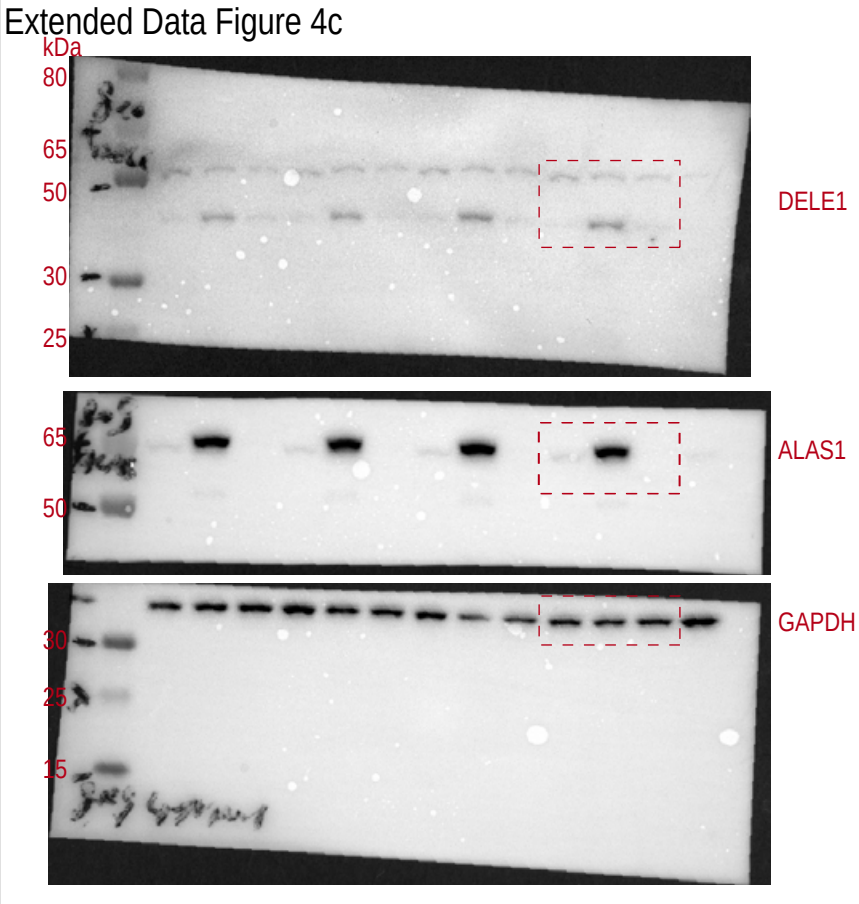

Extended Data Figure 4e

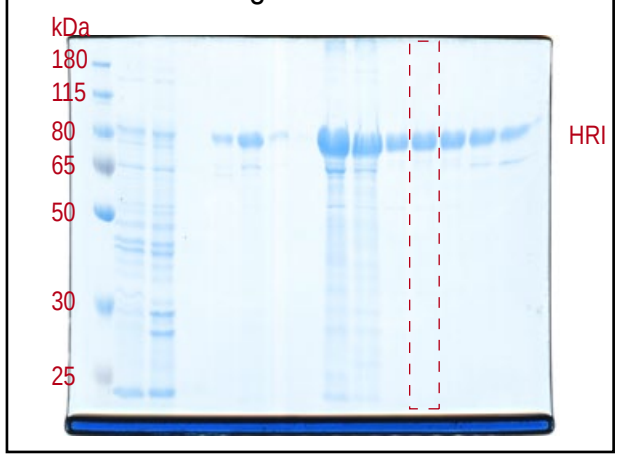

Extended Data Figure 4j

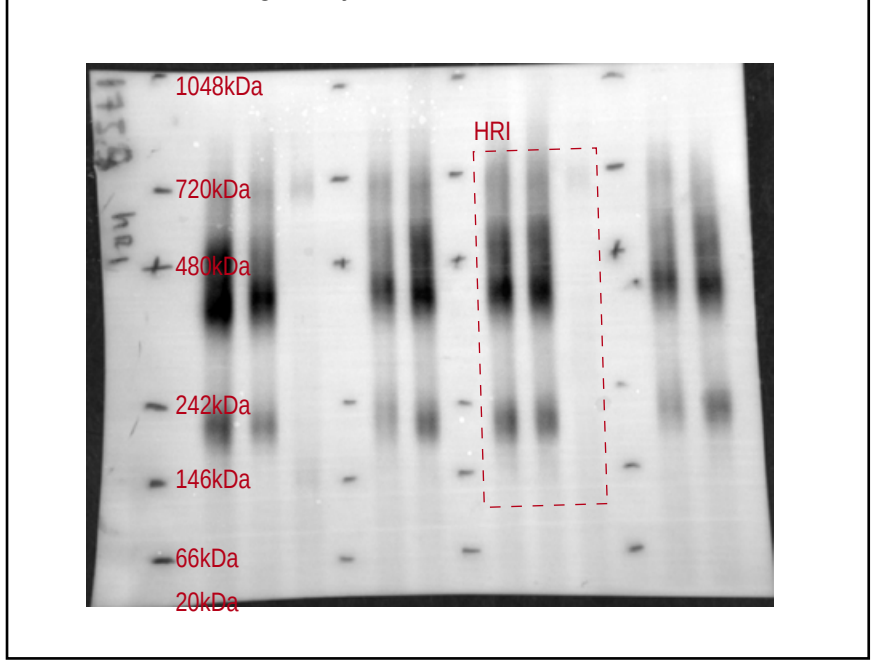

Extended Data Figure 4g

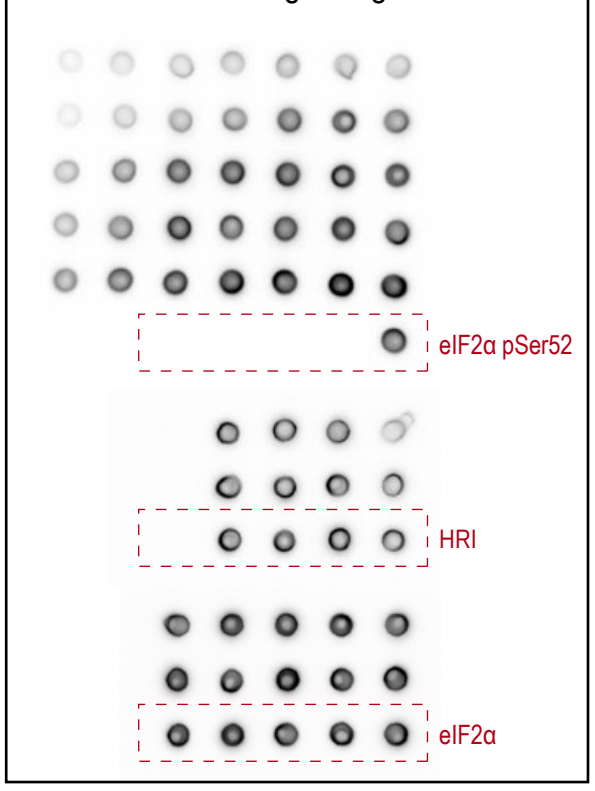

Extended Data Figure 4k

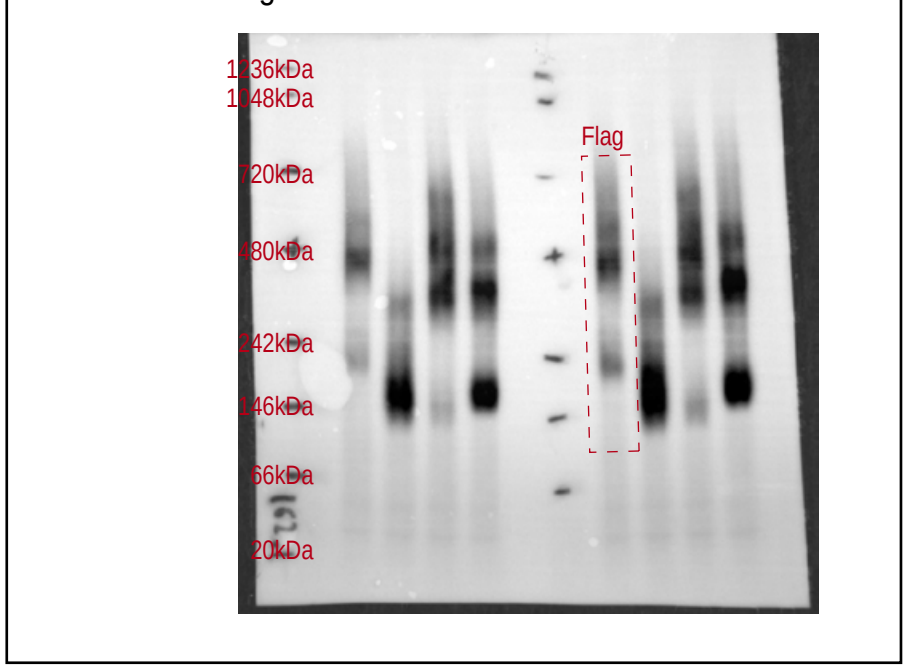

Extended Data Figure 4d

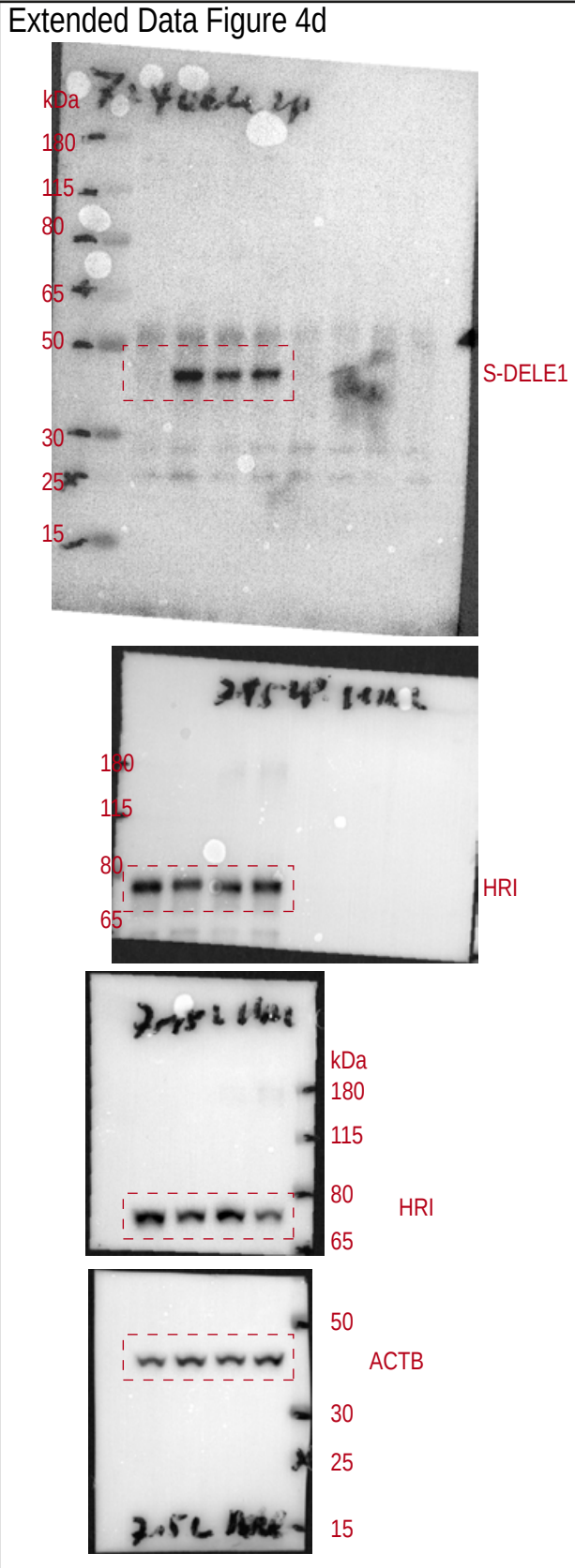

Extended Data Figure 4l

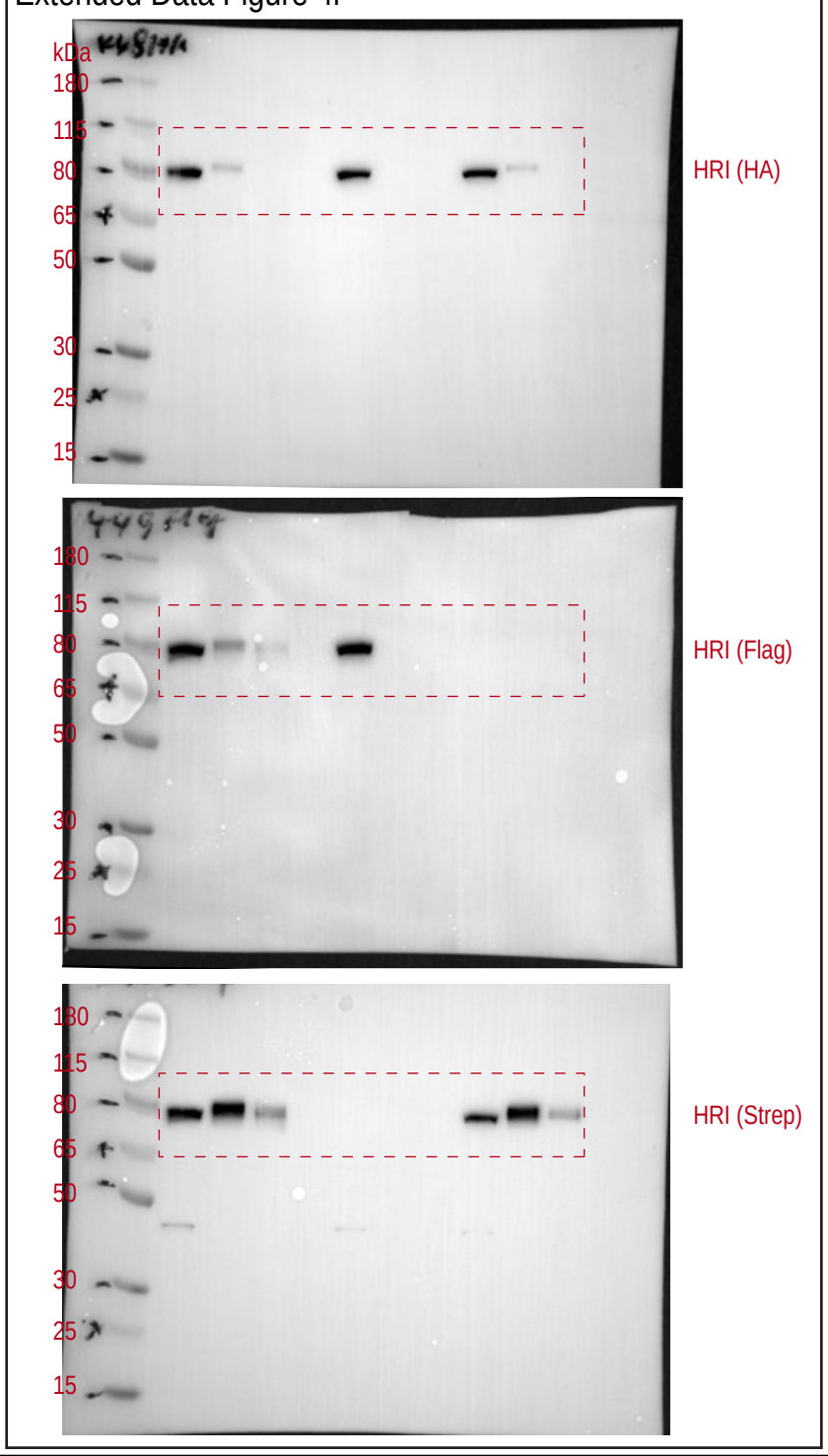

Extended Data Figure 4m

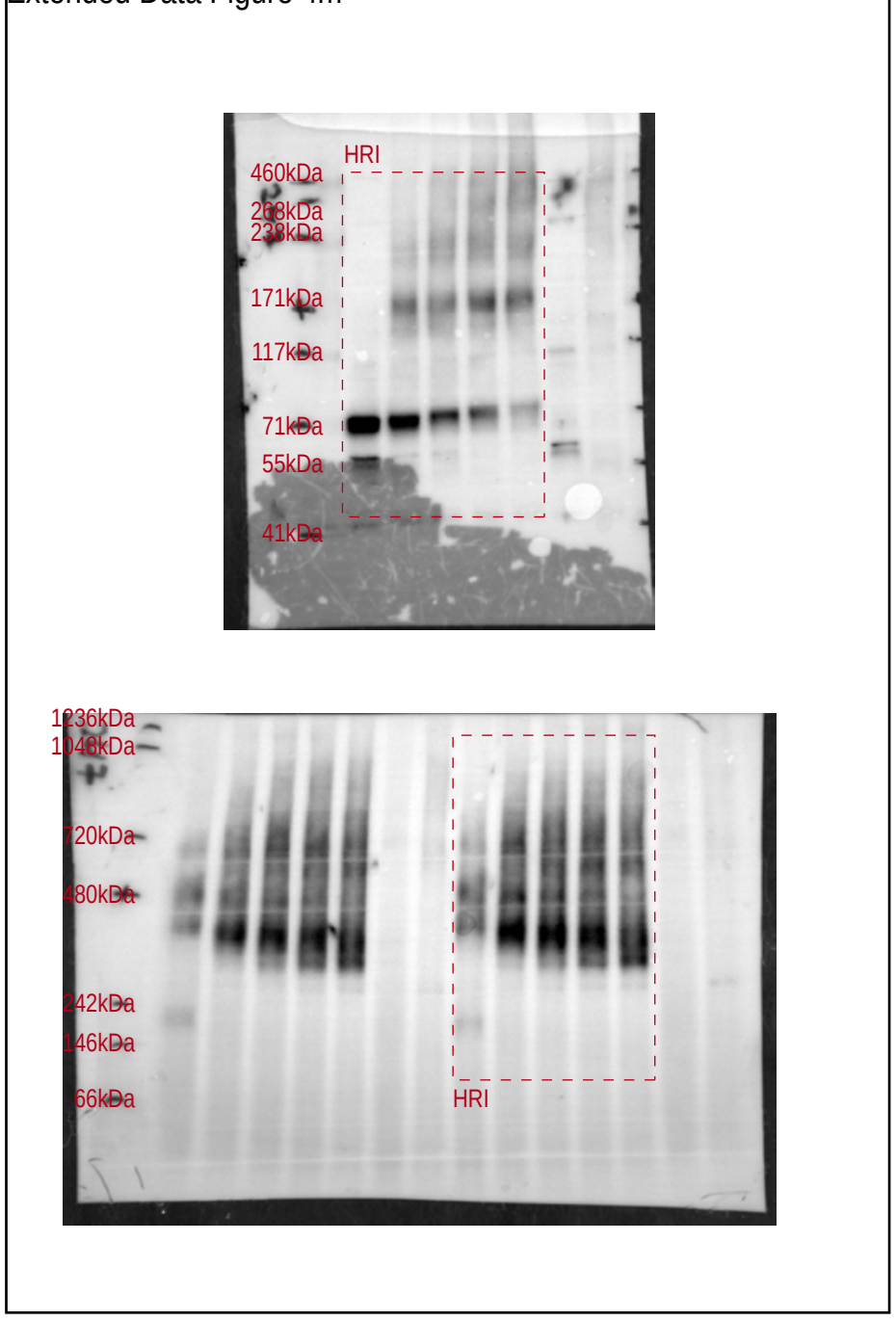

Extended Data Figure 4n

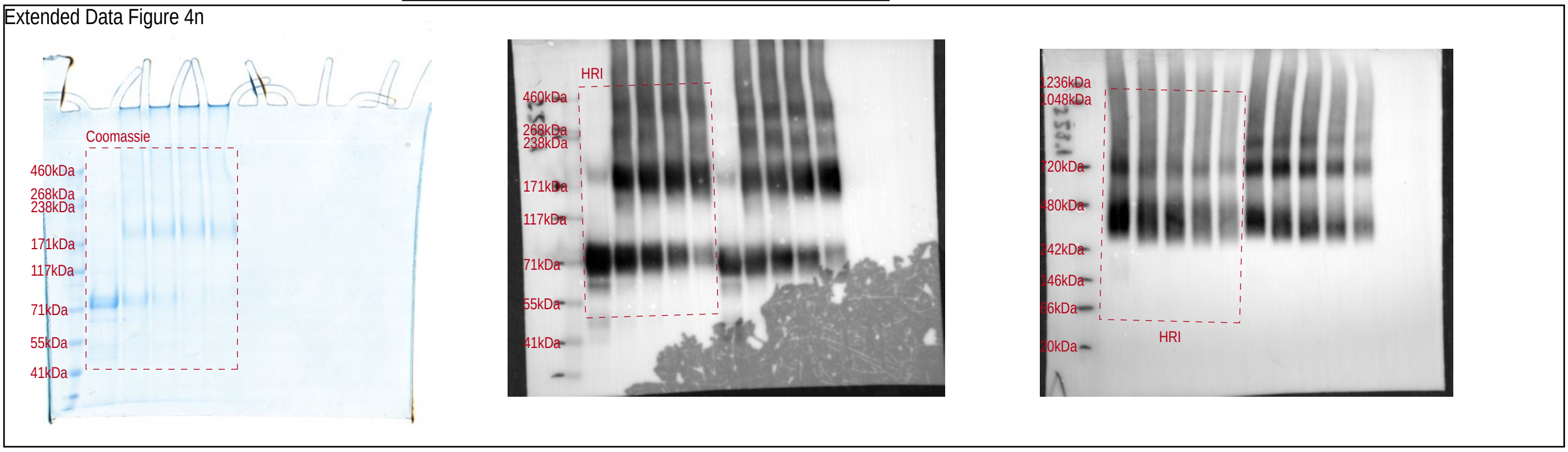

Extended Data Figure 4o

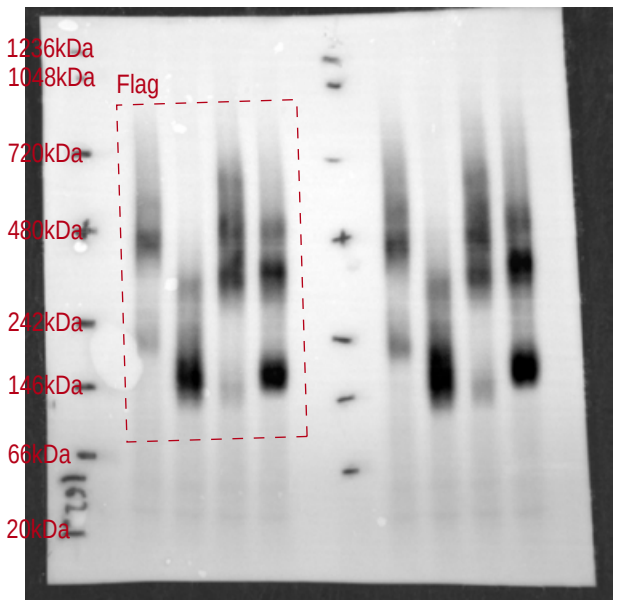

Extended Data Figure 4p

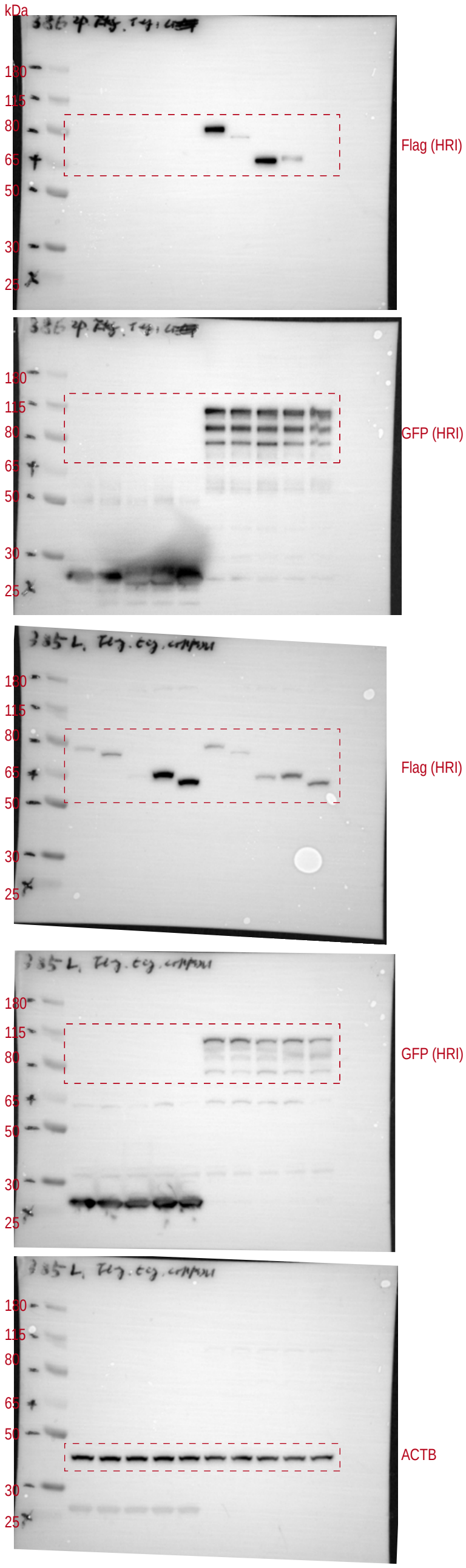

Extended Data Figure 4q

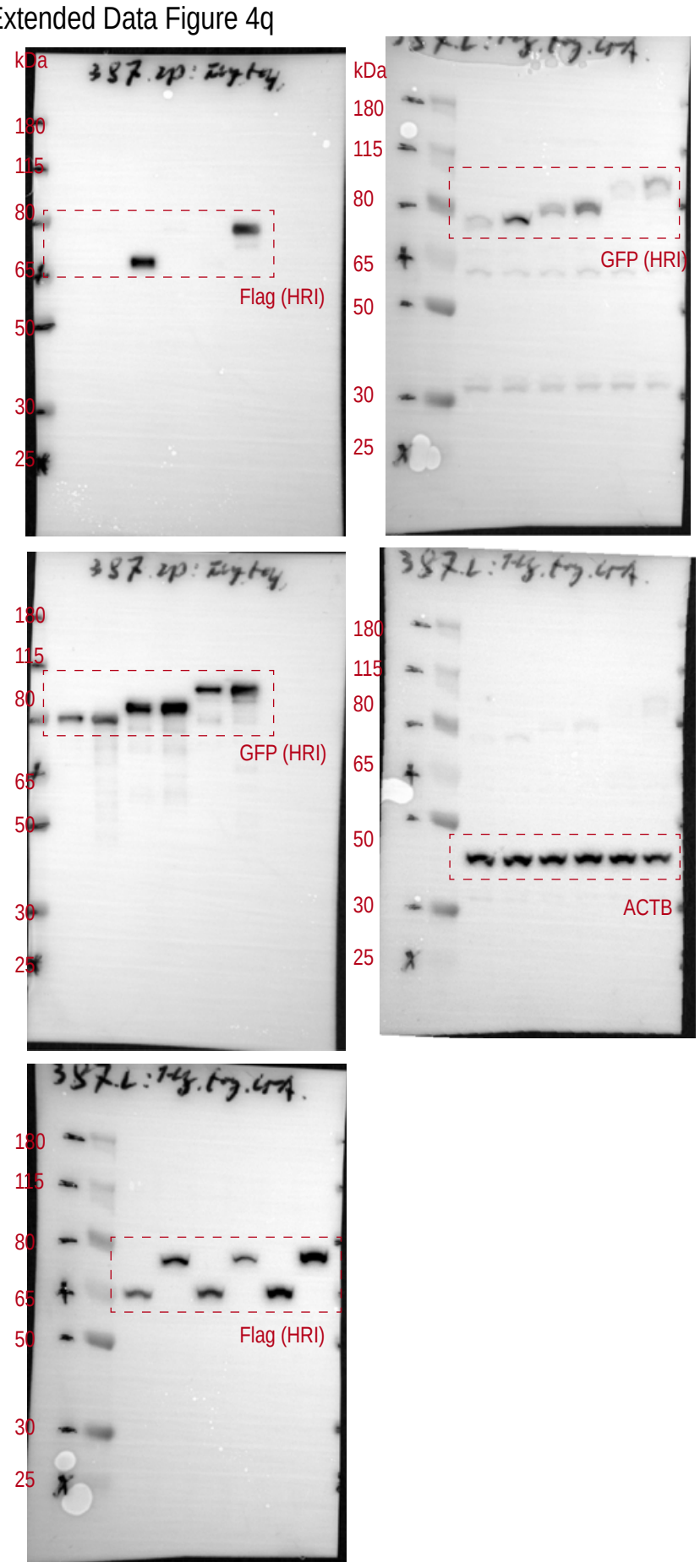

Extended Data Figure 4r

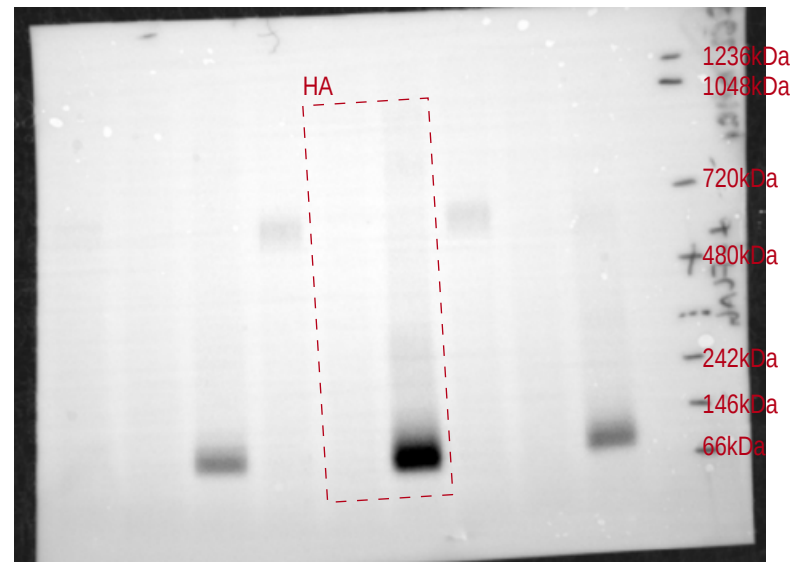

Extended Data Figure 4s

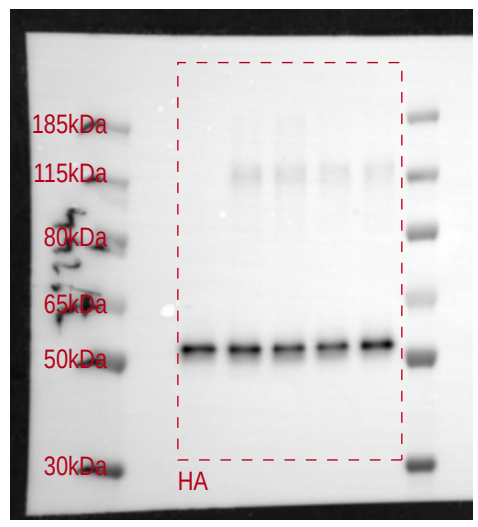

Extended Data Figure 4u

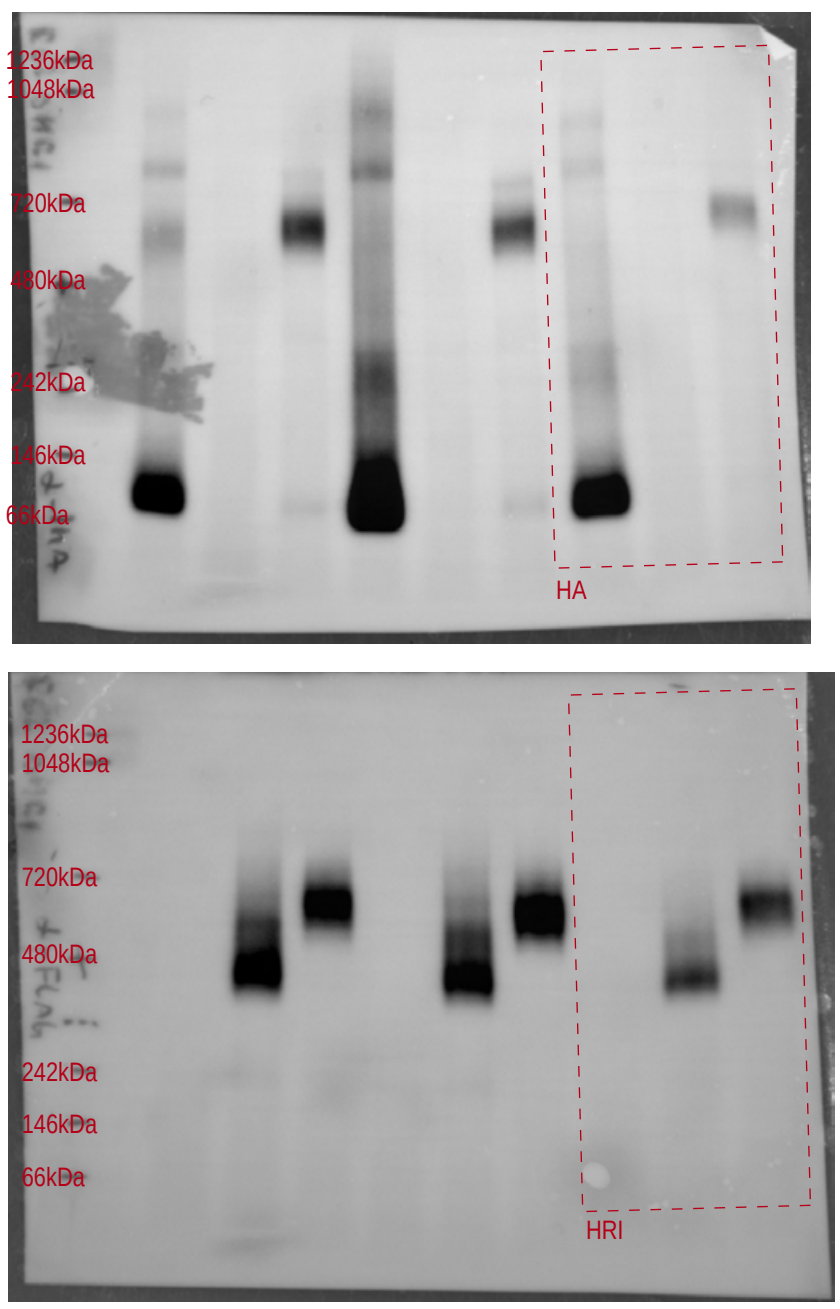

Extended Data Figure 4v

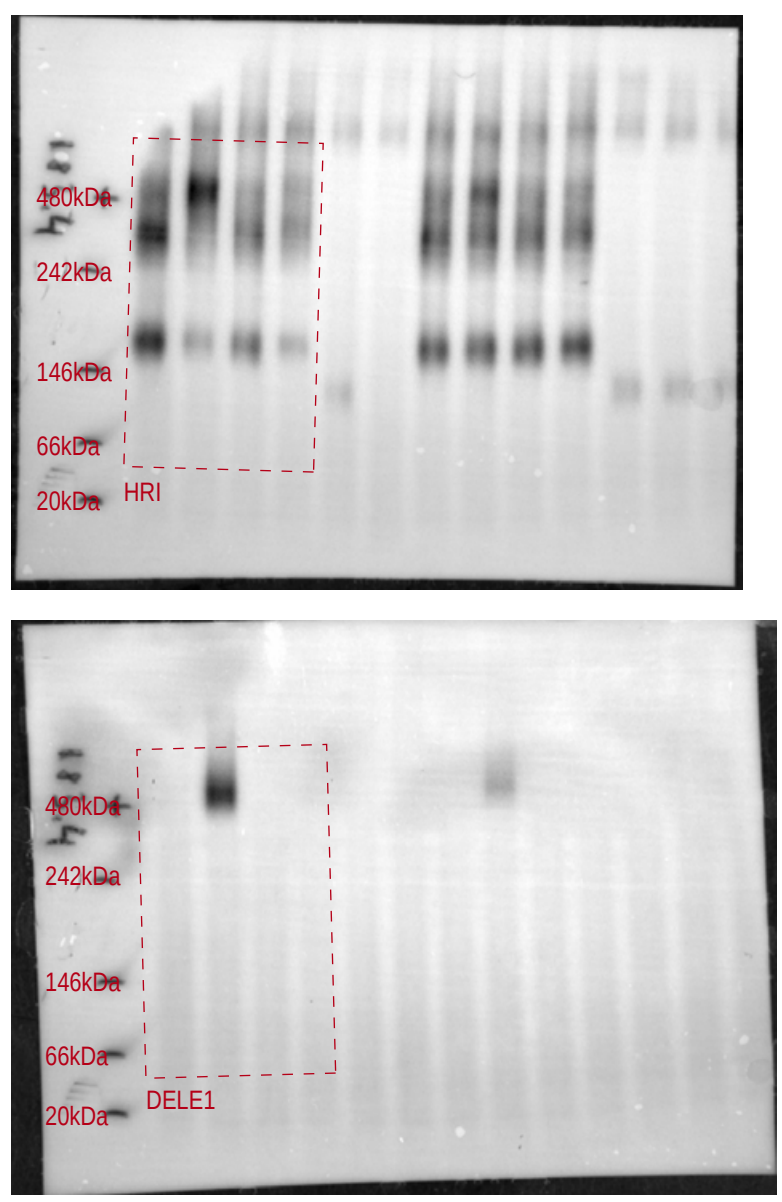

Extended Data Figure 4w

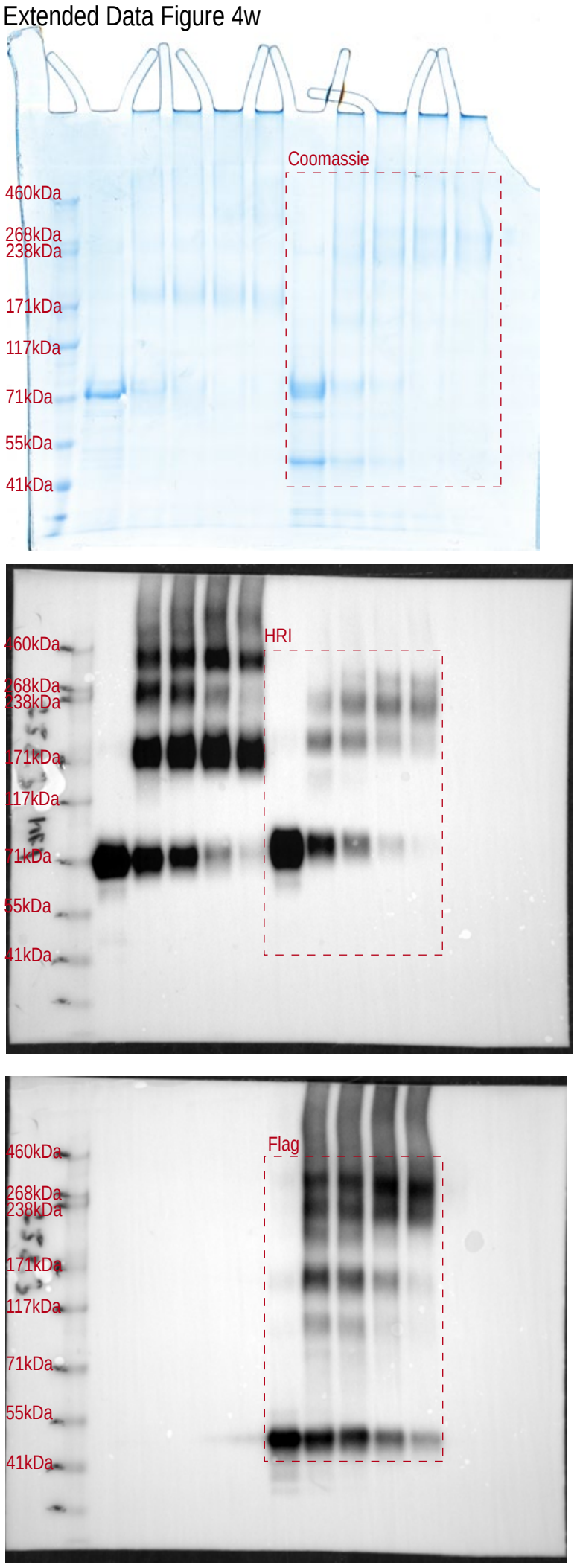

Extended Data Figure 5a

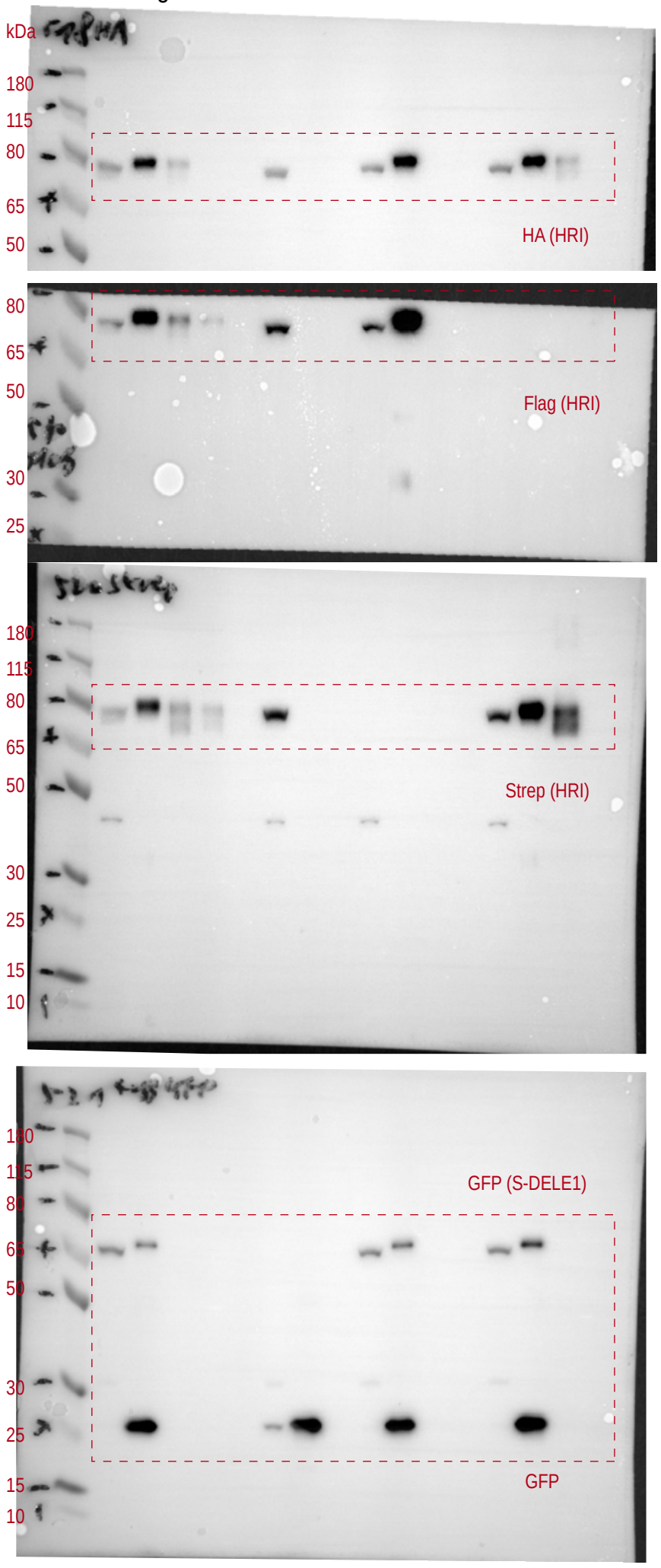

Extended Data Figure 5b

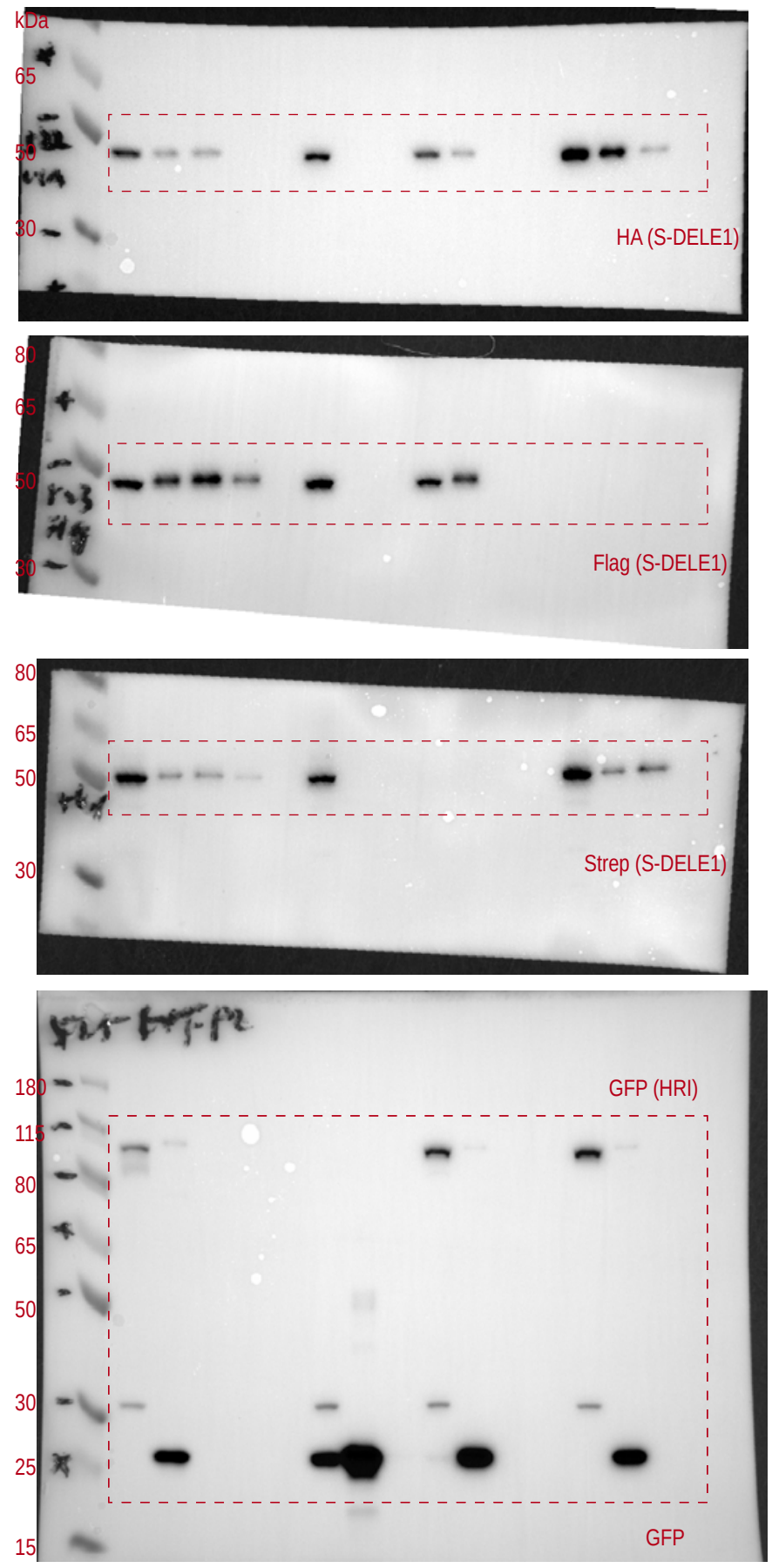

Extended Data Figure 5c

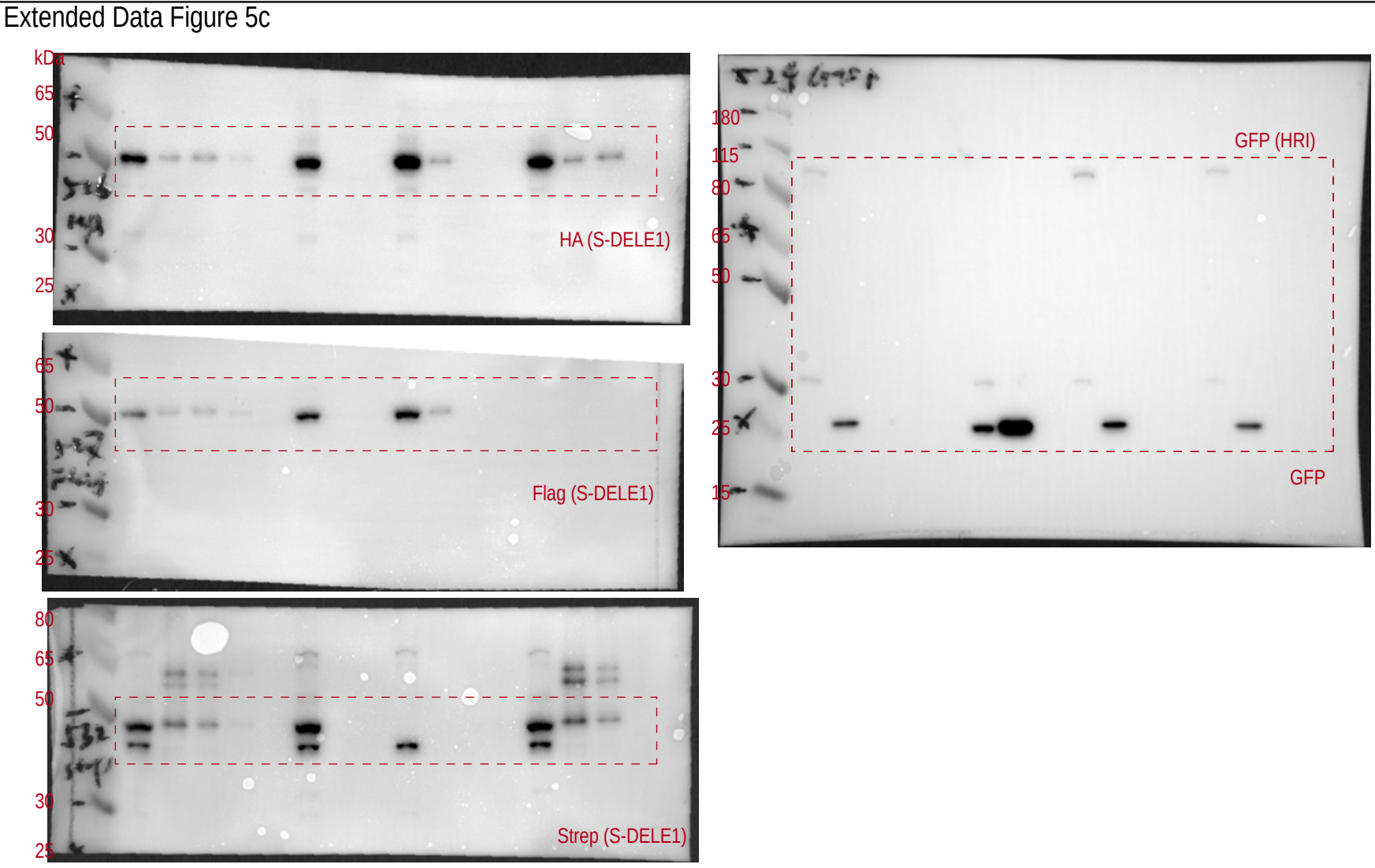

Extended Data Figure 5d

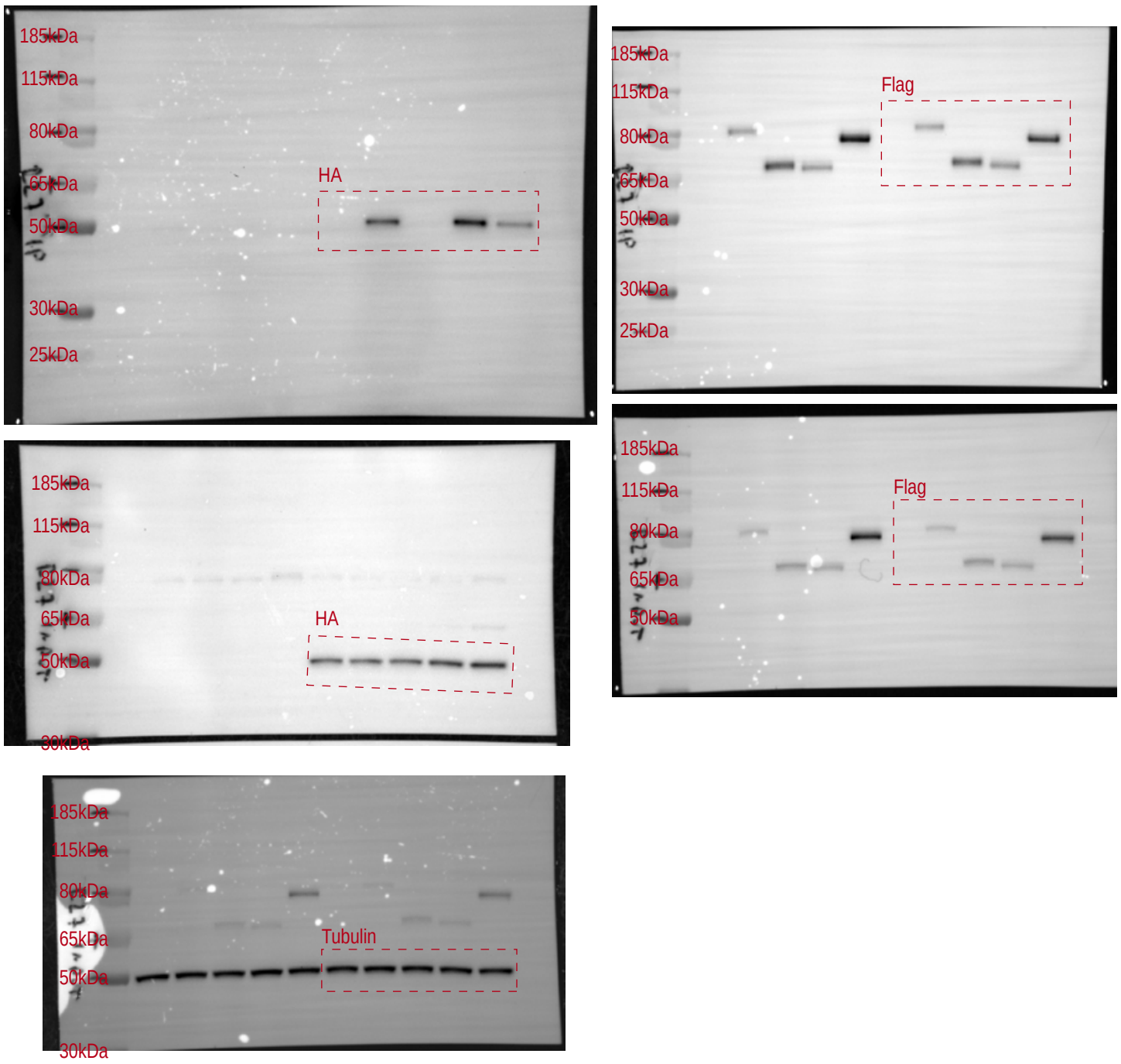

Extended Data Figure 5e

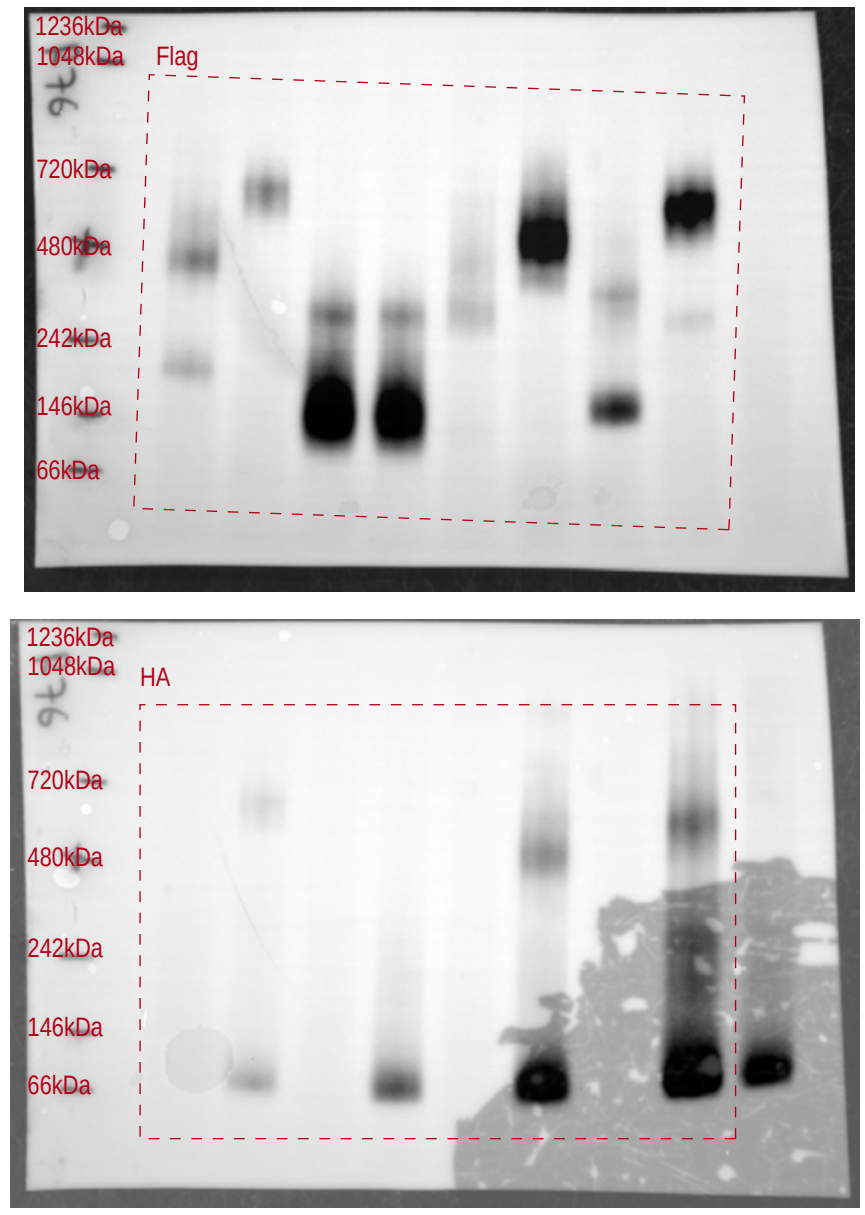

Extended Data Figure 5h

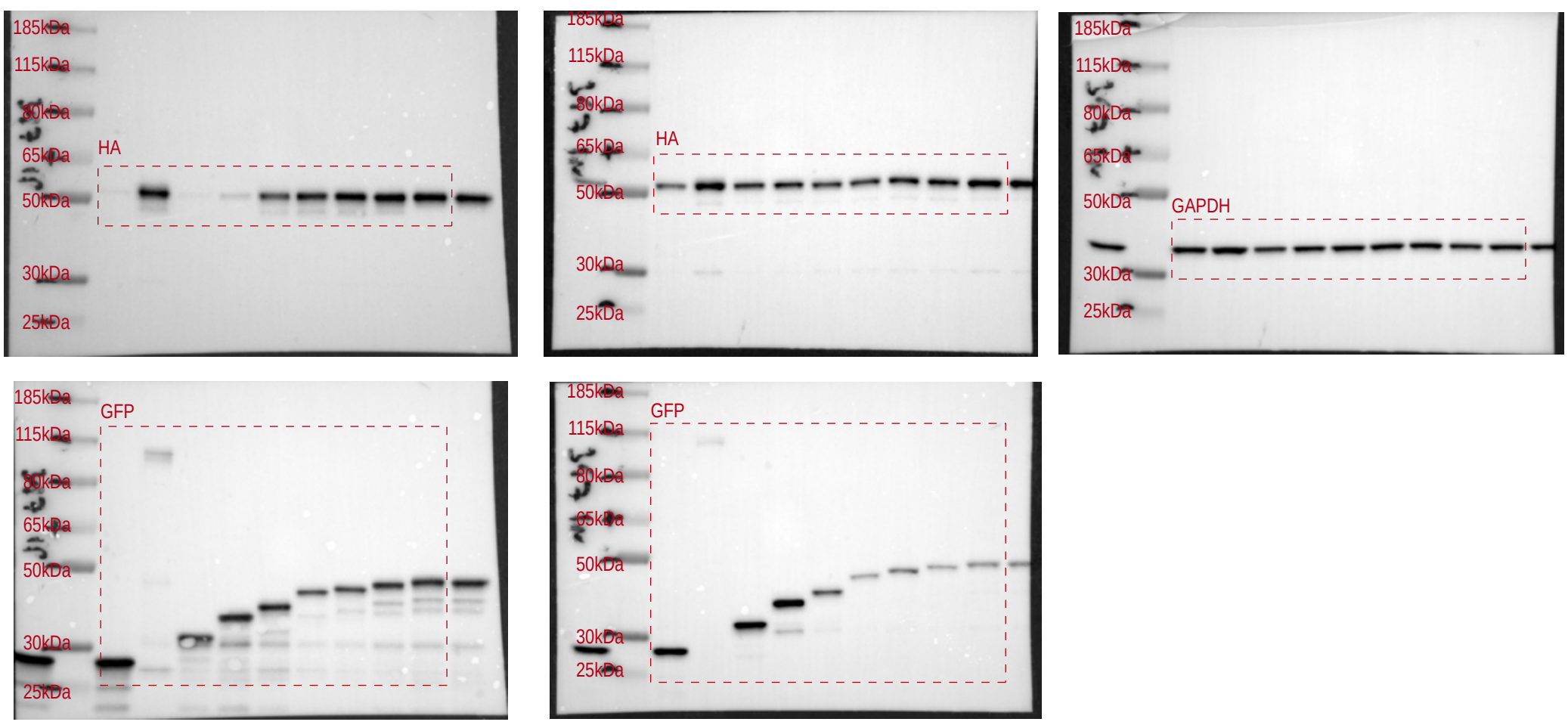

Extended Data Figure 6a

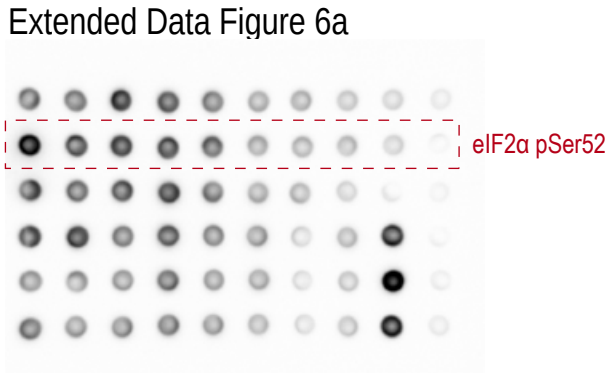

Extended Data Figure 6b

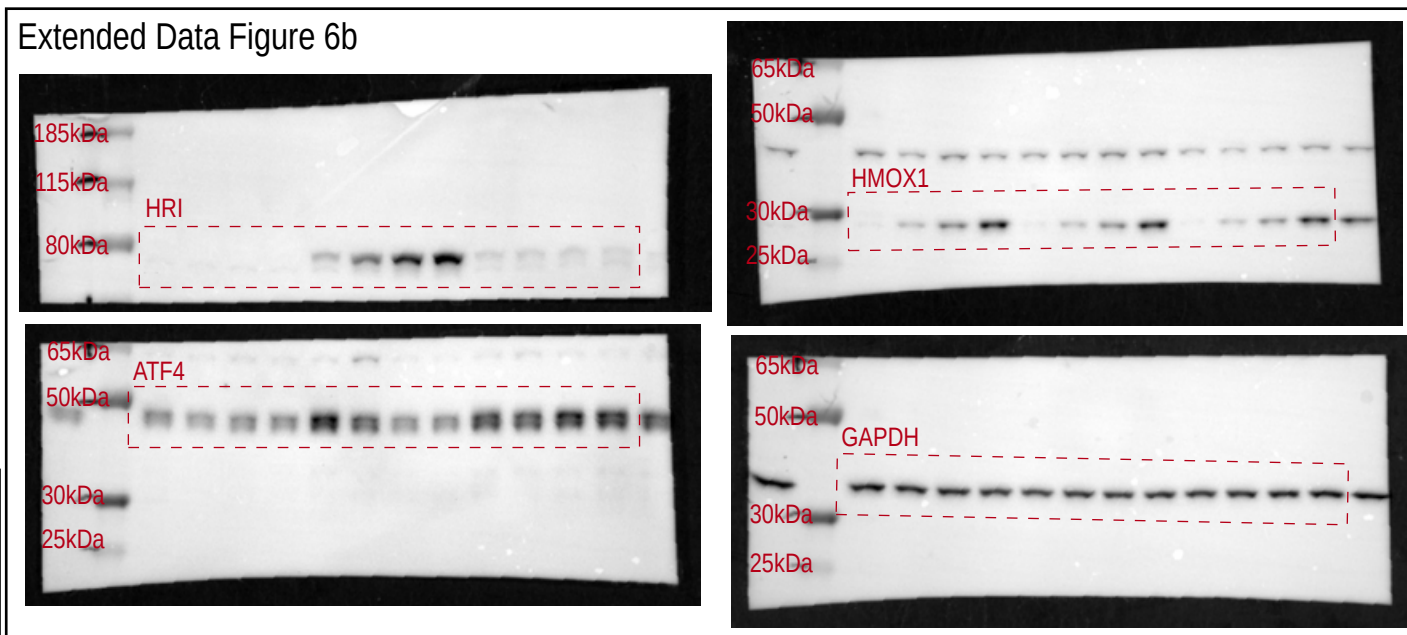

Extended Data Figure 6c

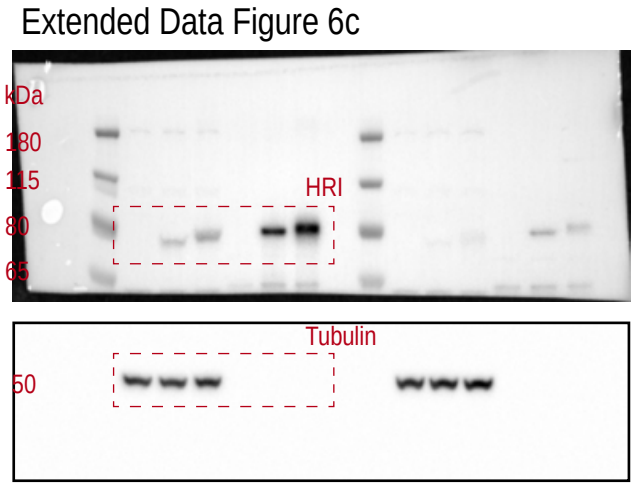

Extended Data Figure 6f

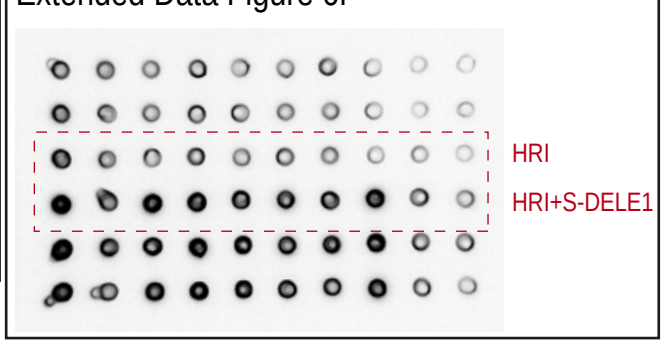

Extended Data Figure 6g

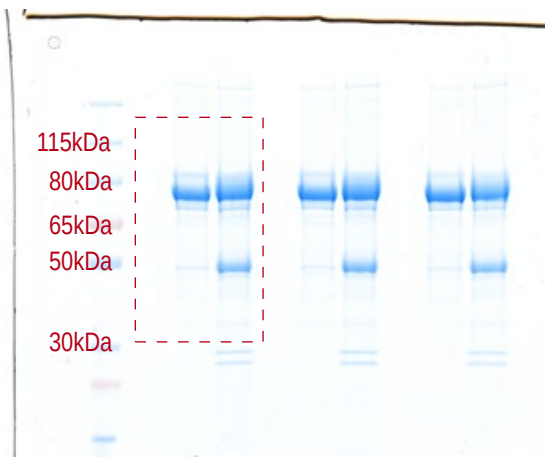

Extended Data Figure 6h

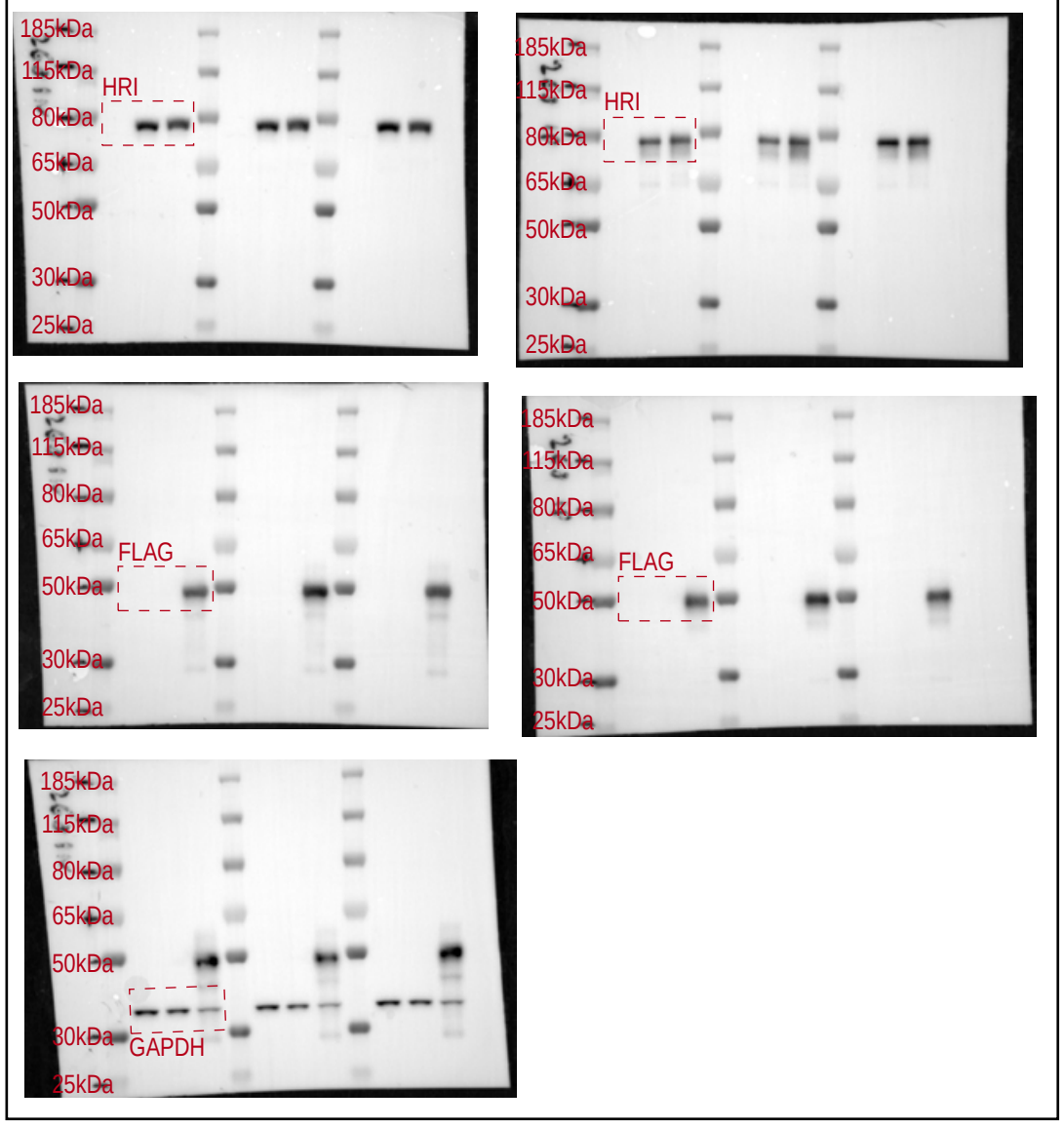

Extended Data Figure 6j

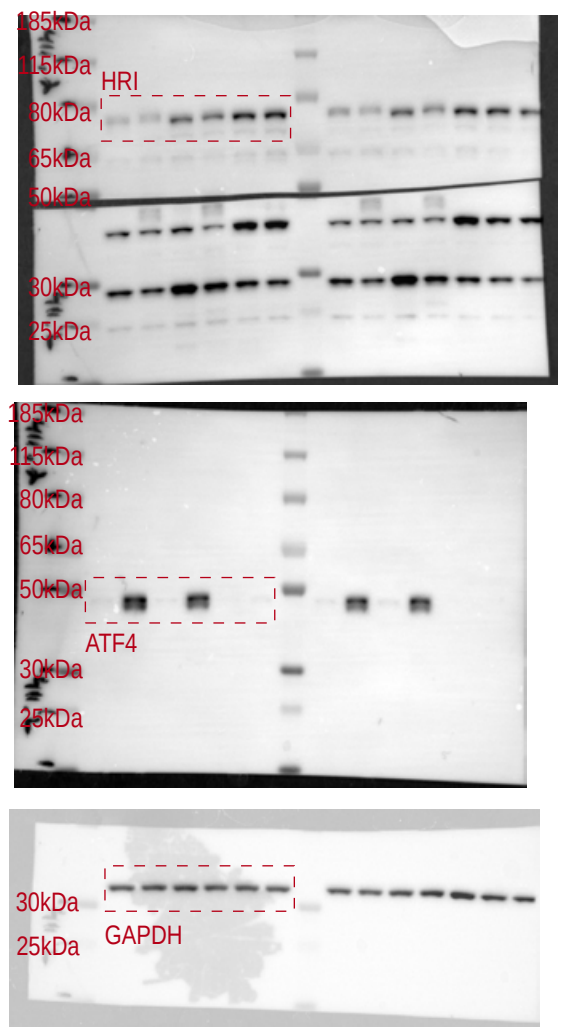

Extended Data Figure 6k

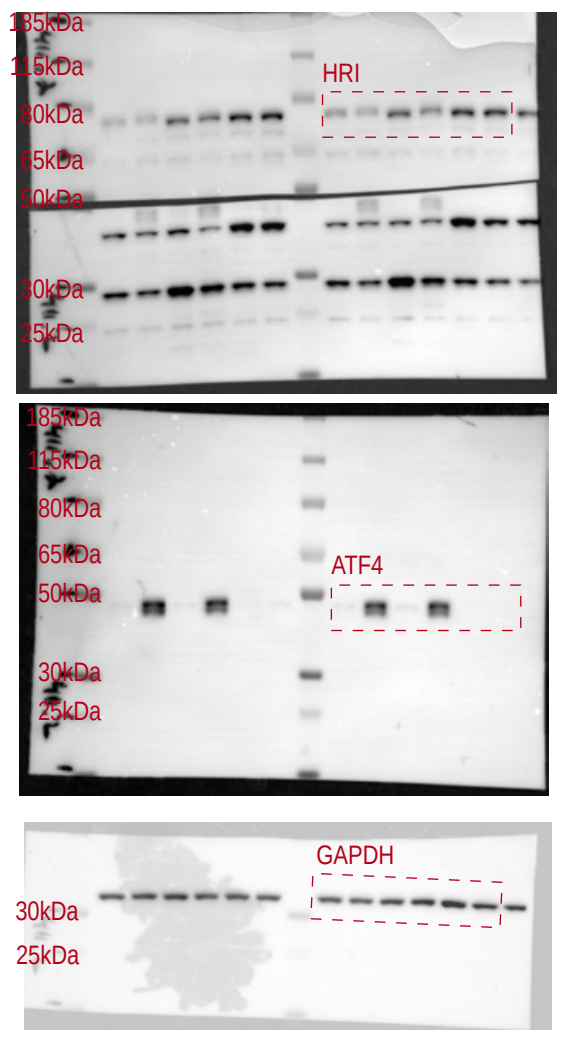

Extended Data Figure 6l

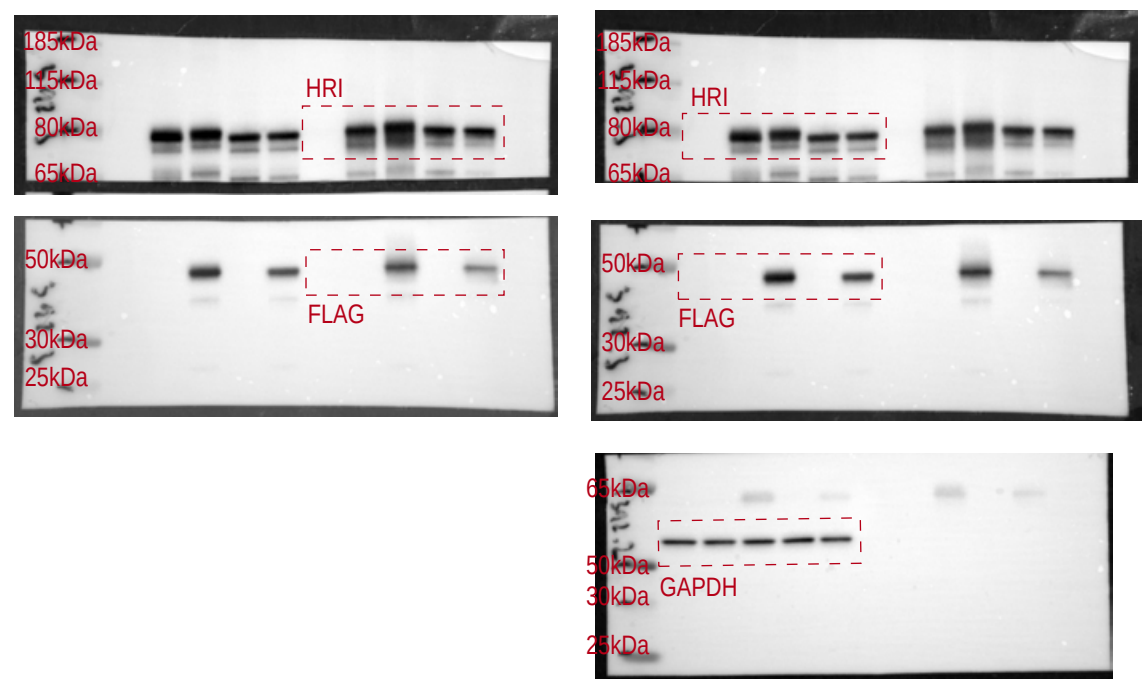

Extended Data Figure 7b

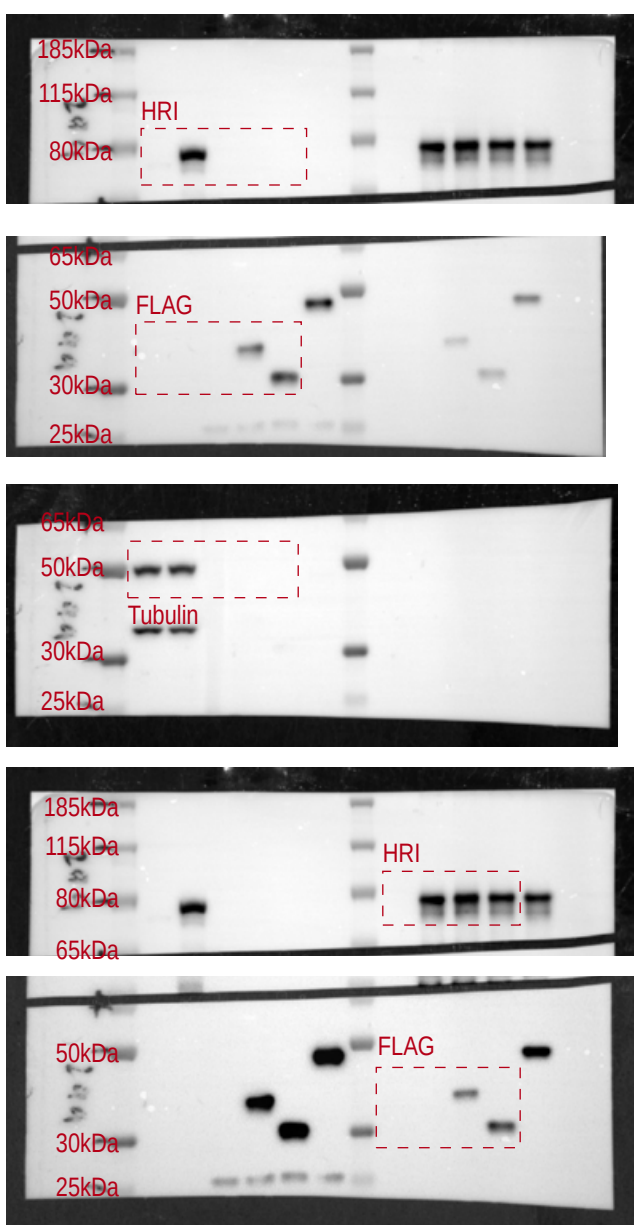

Extended Data Figure 7c

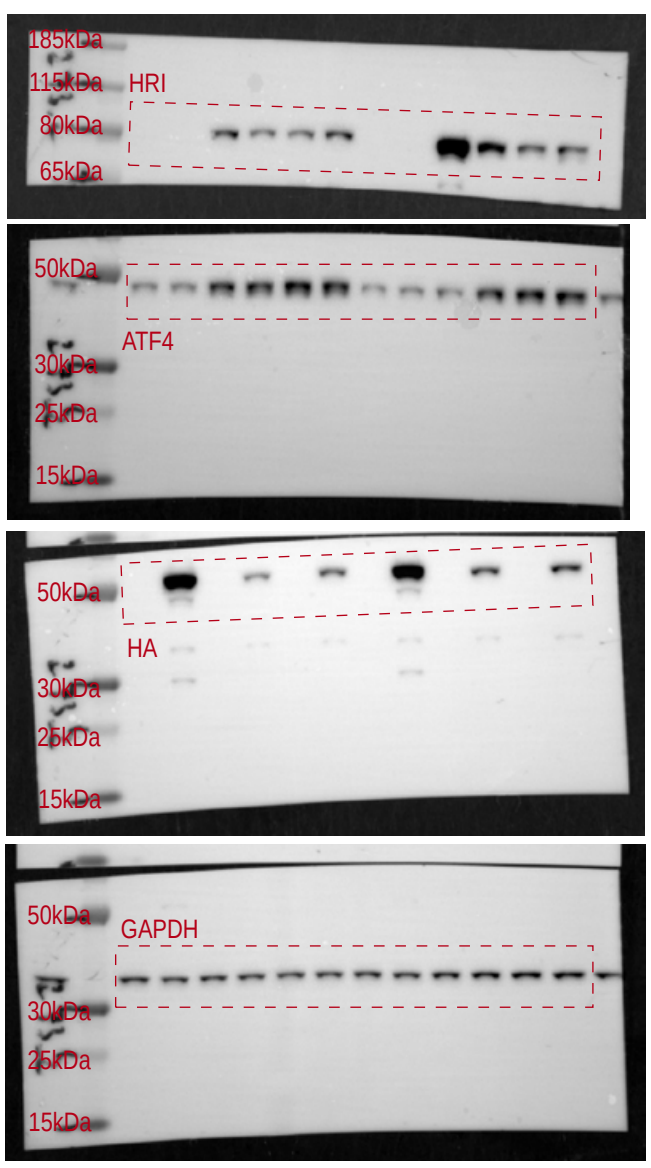

Extended Data Figure 6n

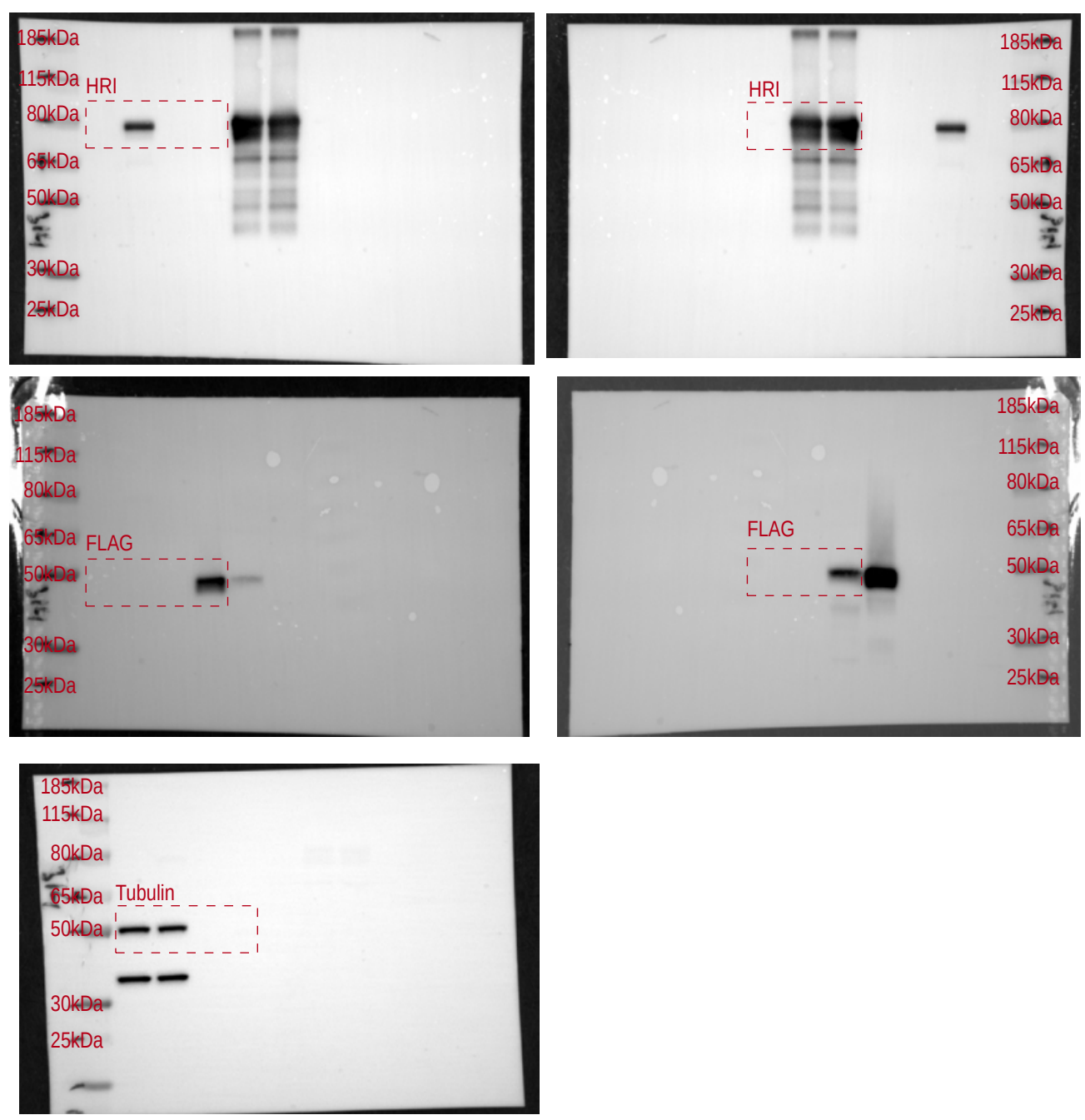

Extended Data Figure 7d

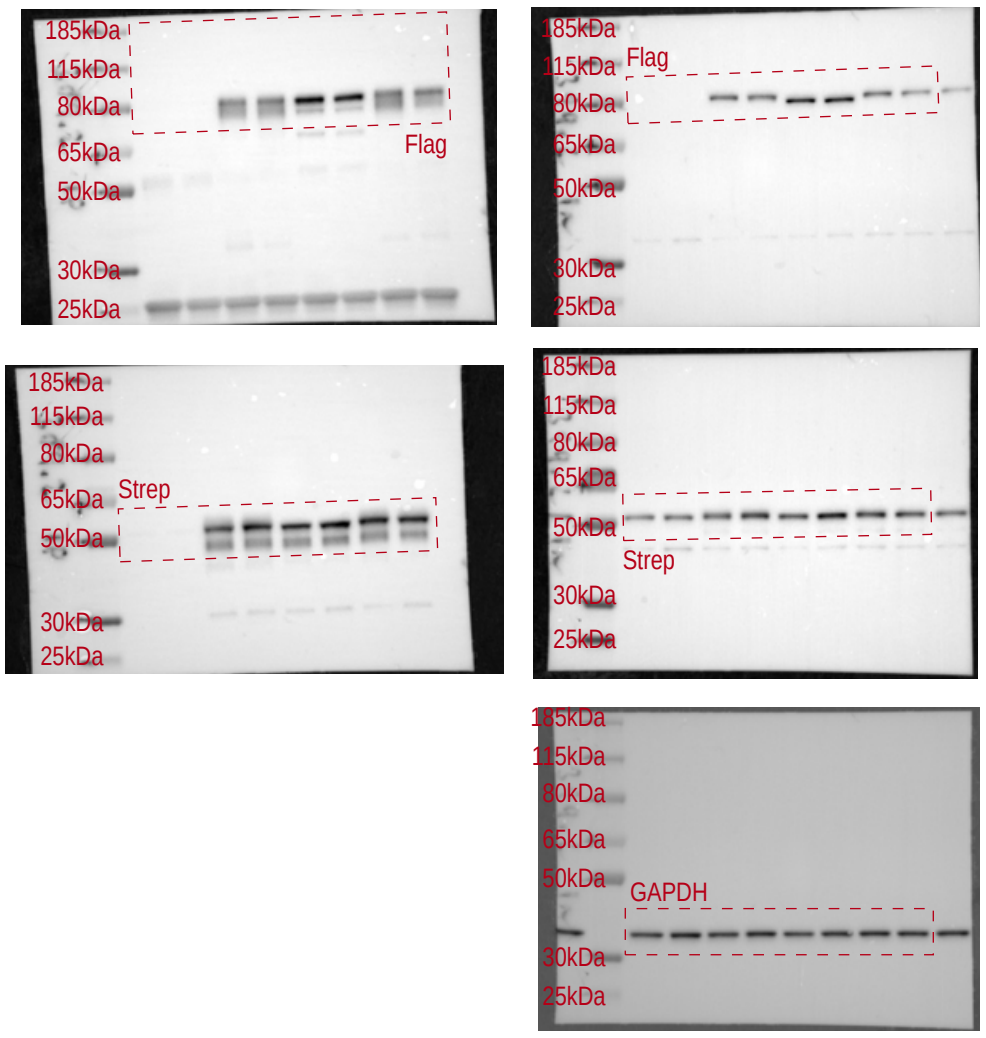

Extended Data Figure 7e

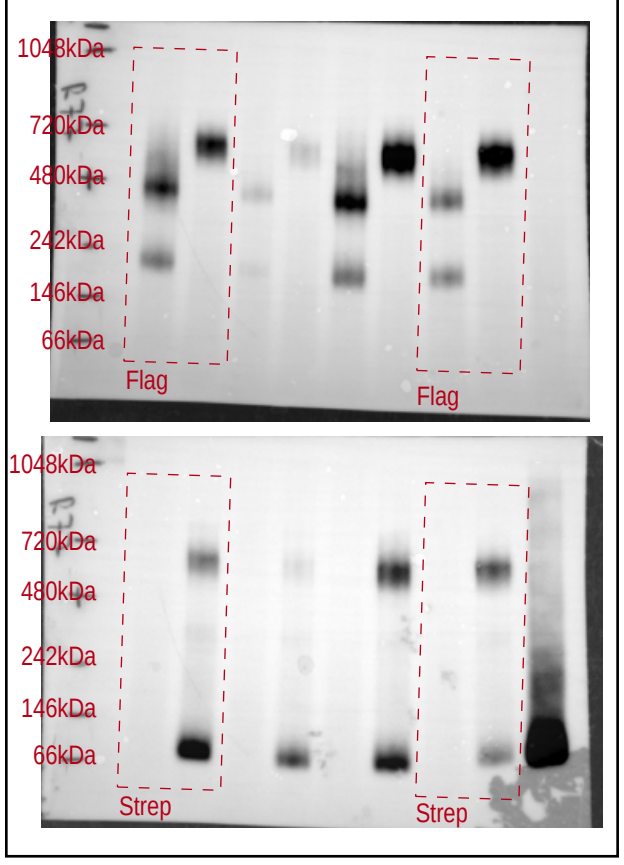

Extnded Data Figure 8a

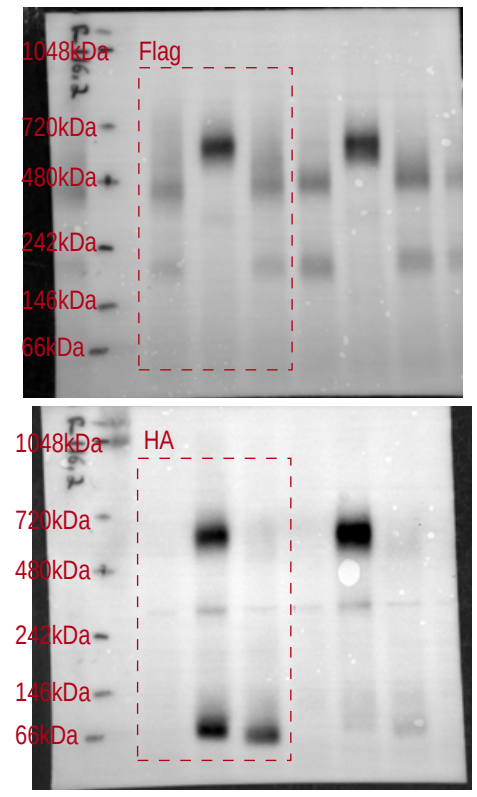

Extended Data Figure 8b

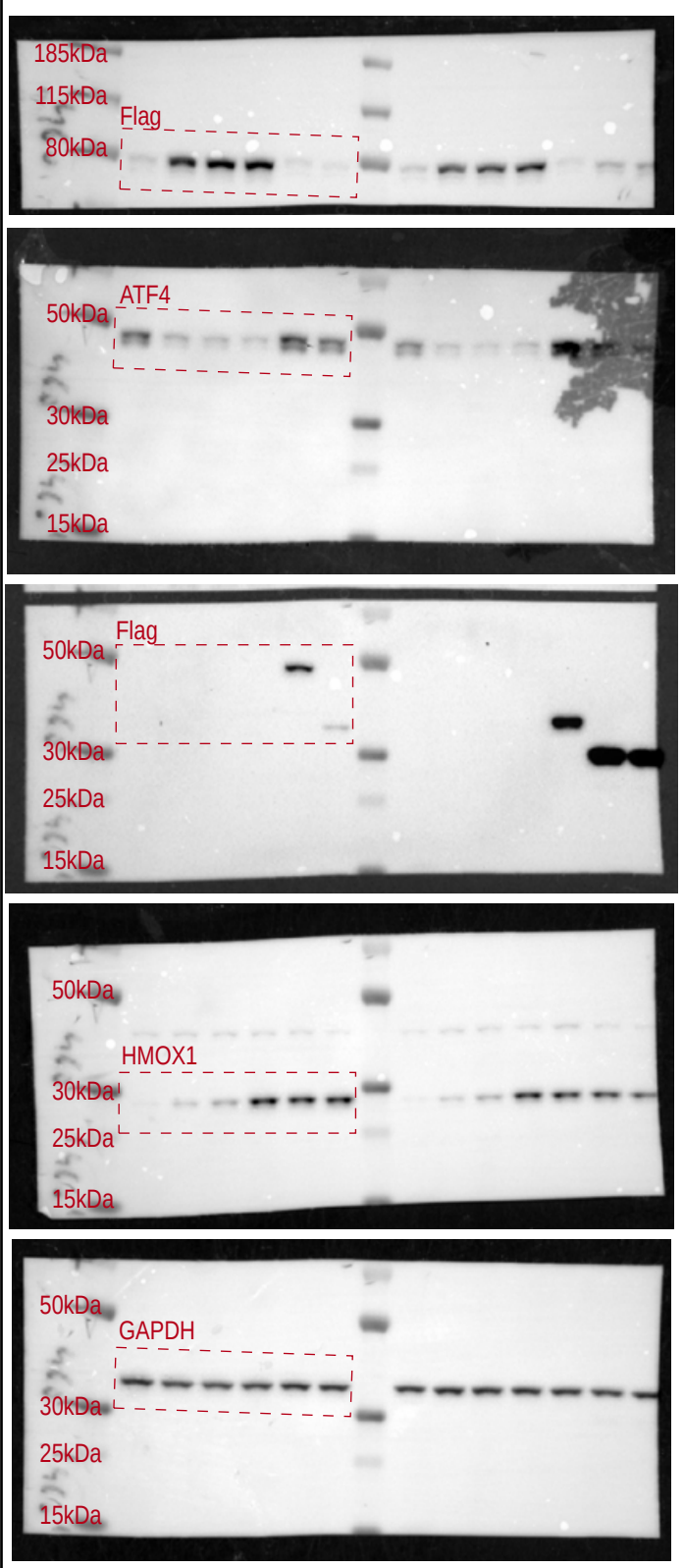

Extended Data Figure 8c

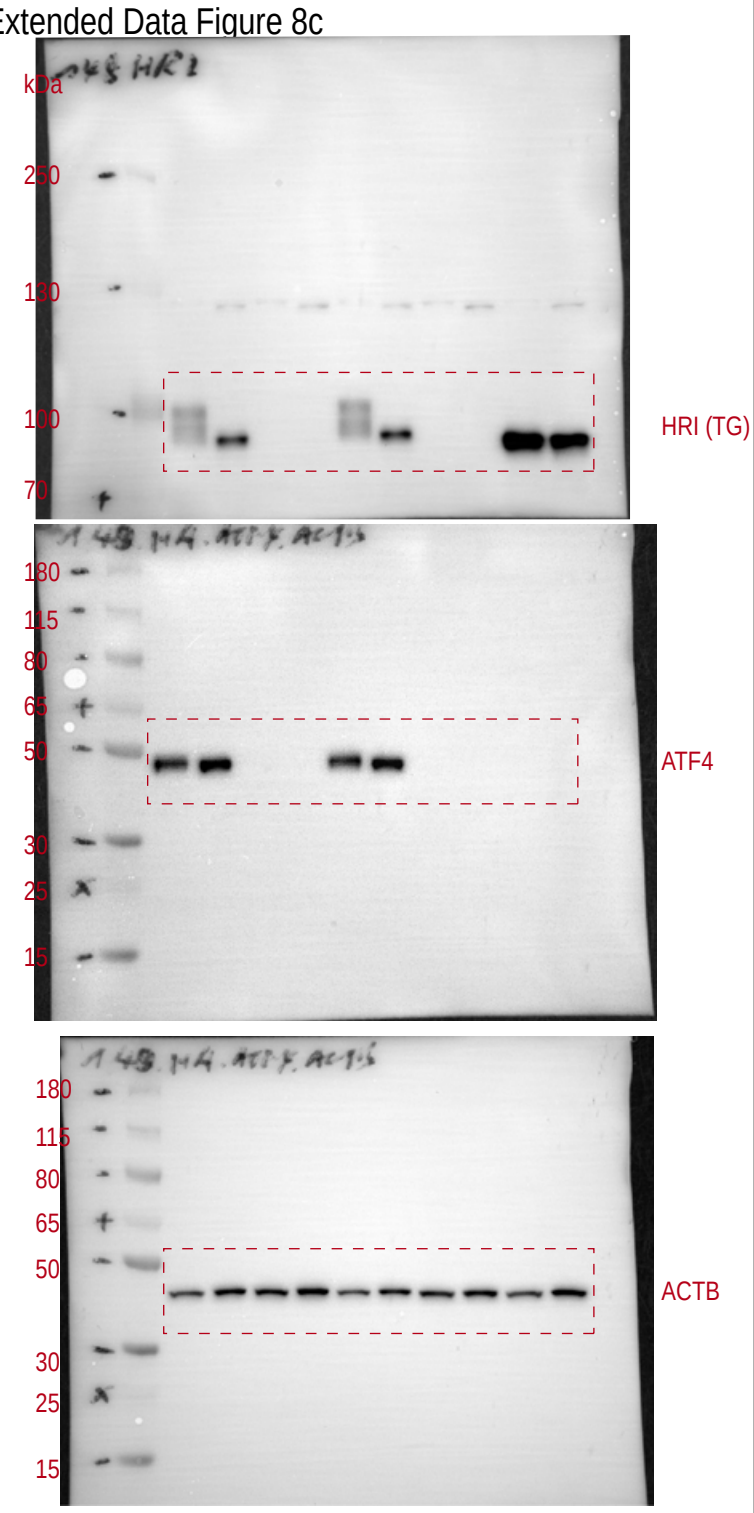

Extended Data Figure 8d

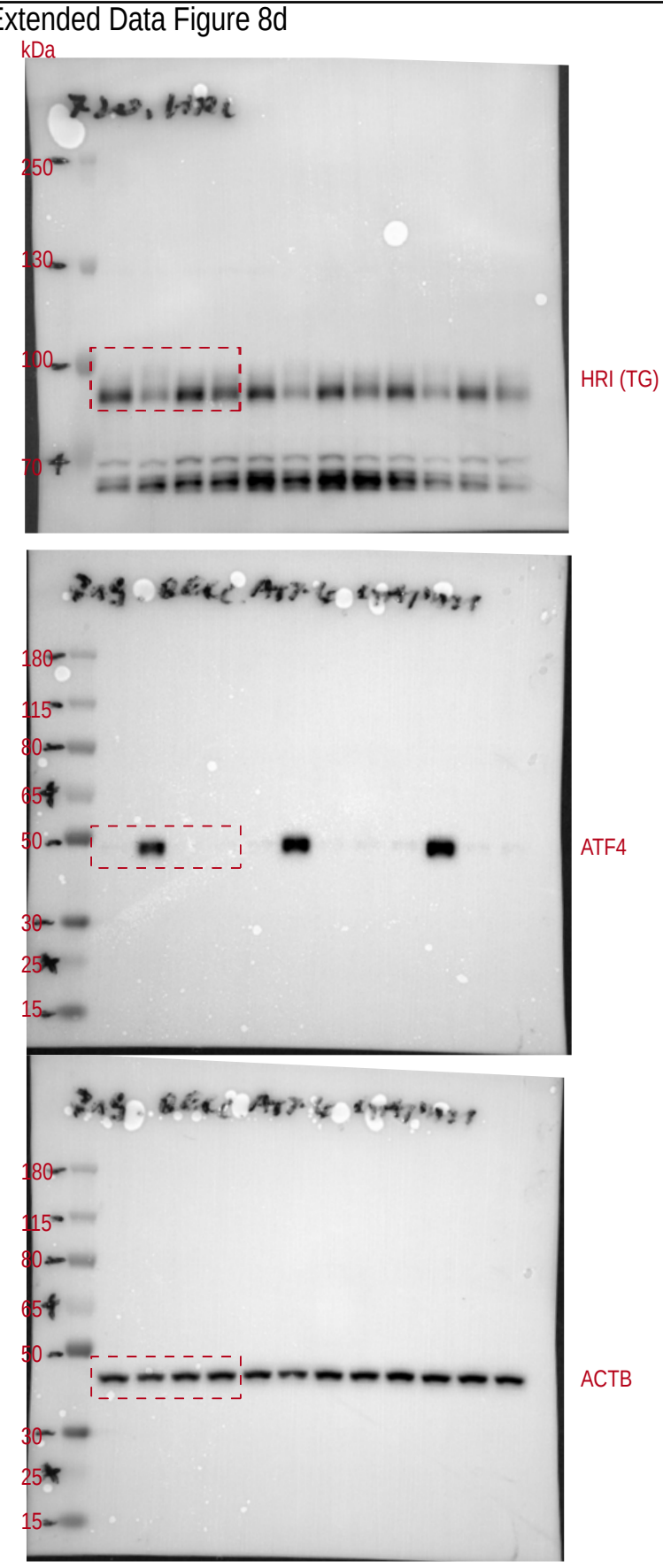

Extnded Data Figure 8e

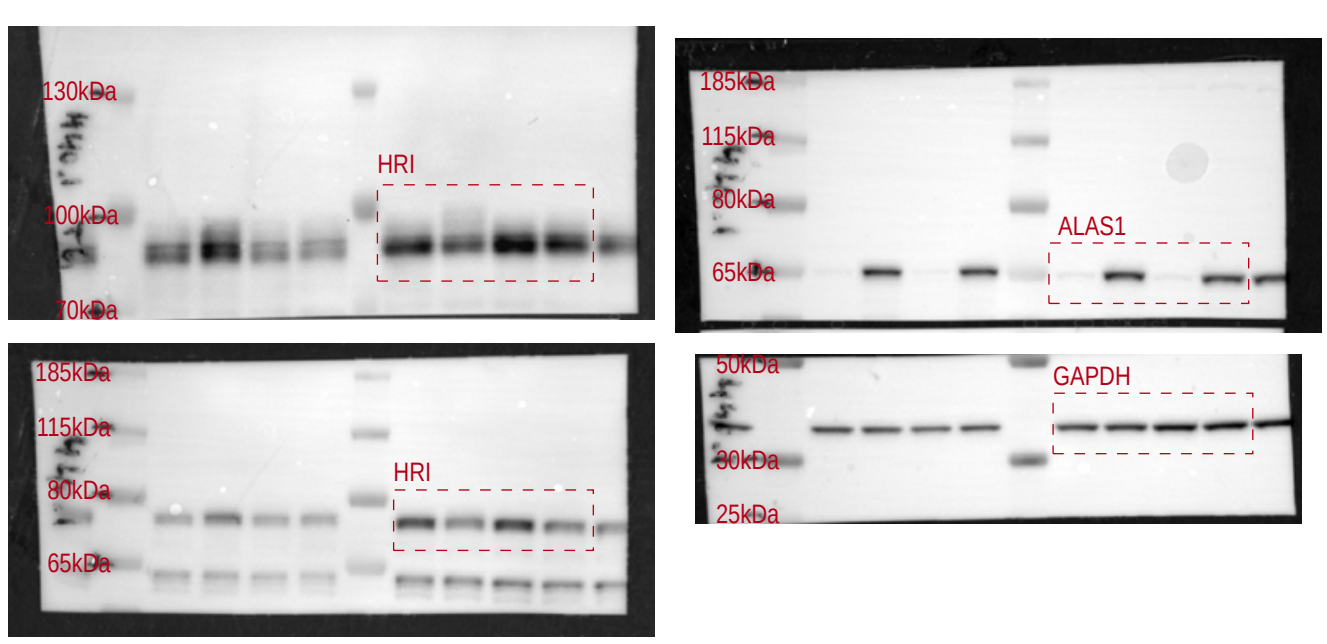

Extnded Data Figure 8f

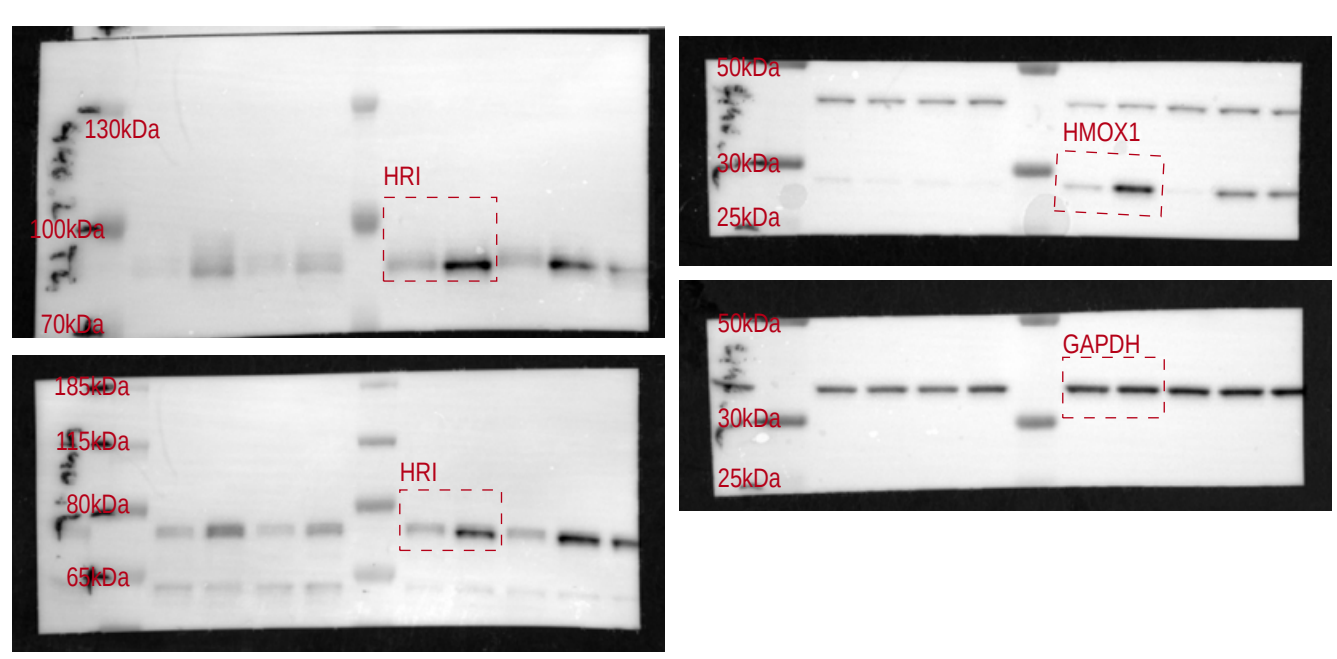

Extnded Data Figure 8g

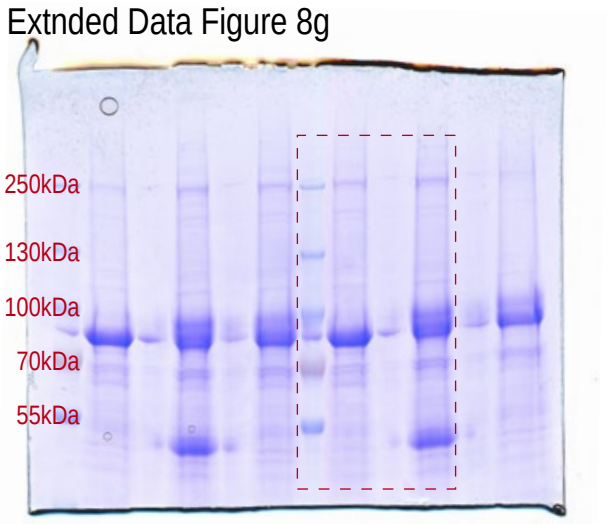

Extended Data Figure 8m

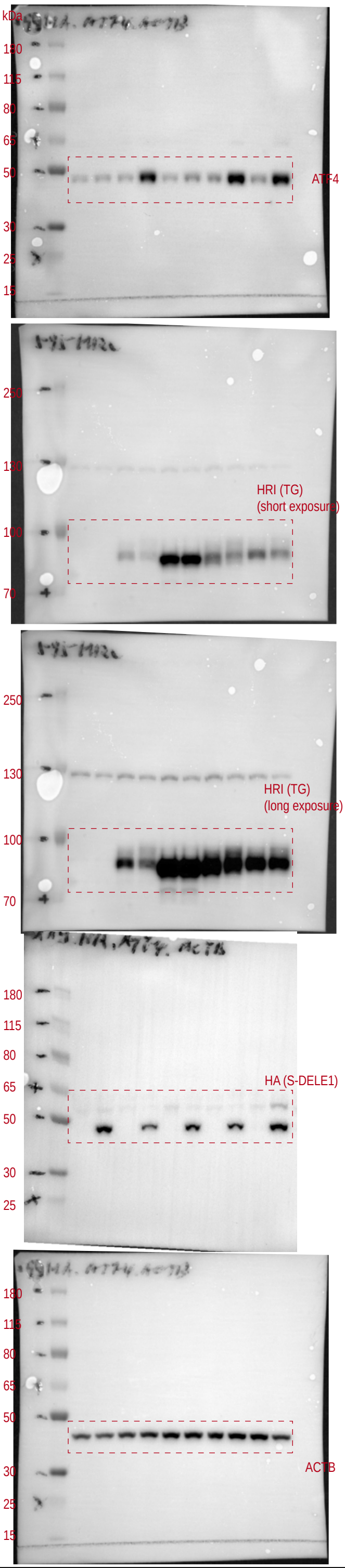

Extnded Data Figure 8n

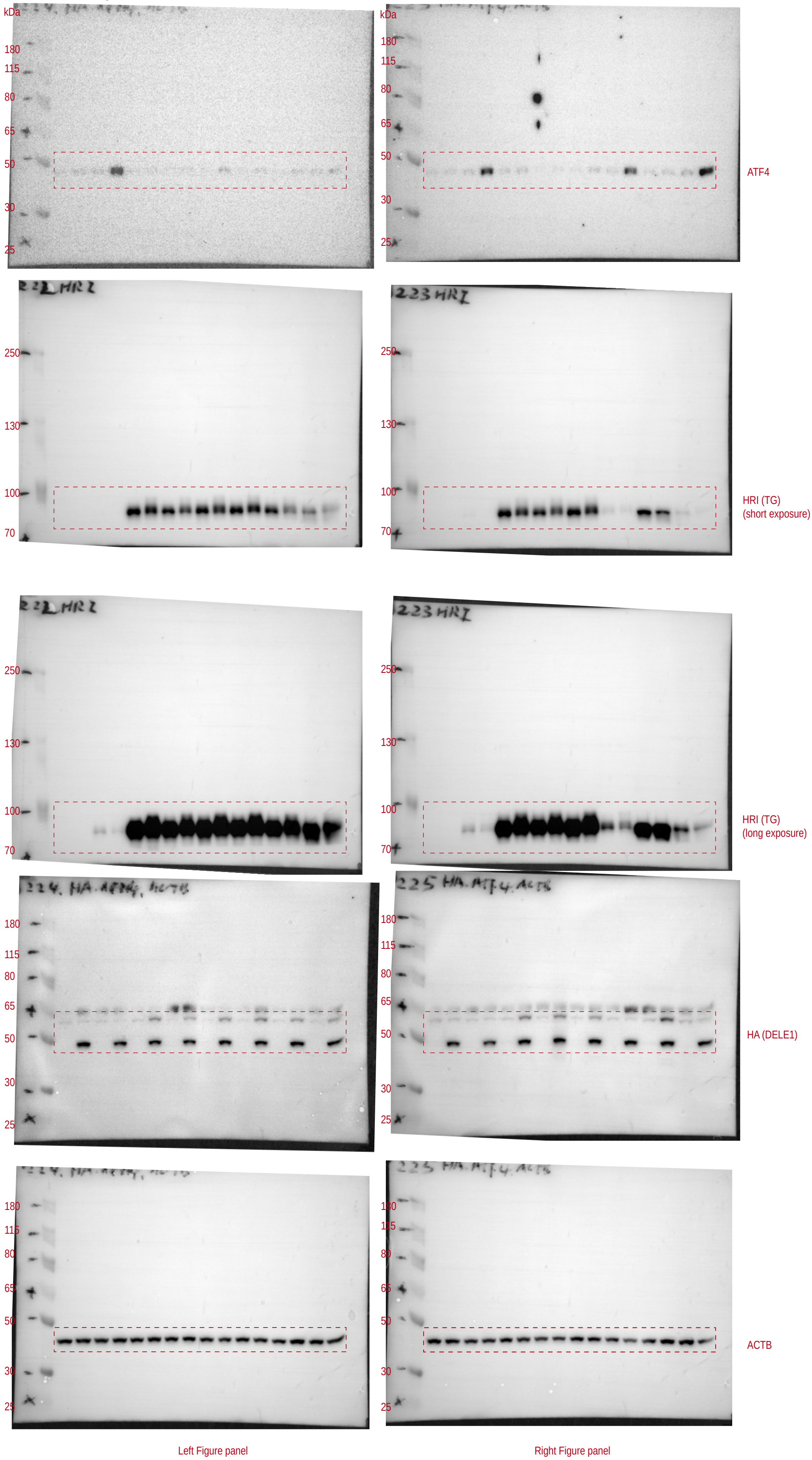

Extnded Data Figure 8o

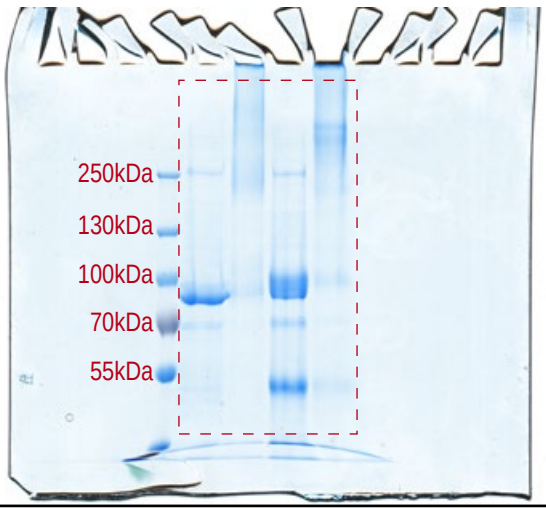

Extended Data Figure 8q

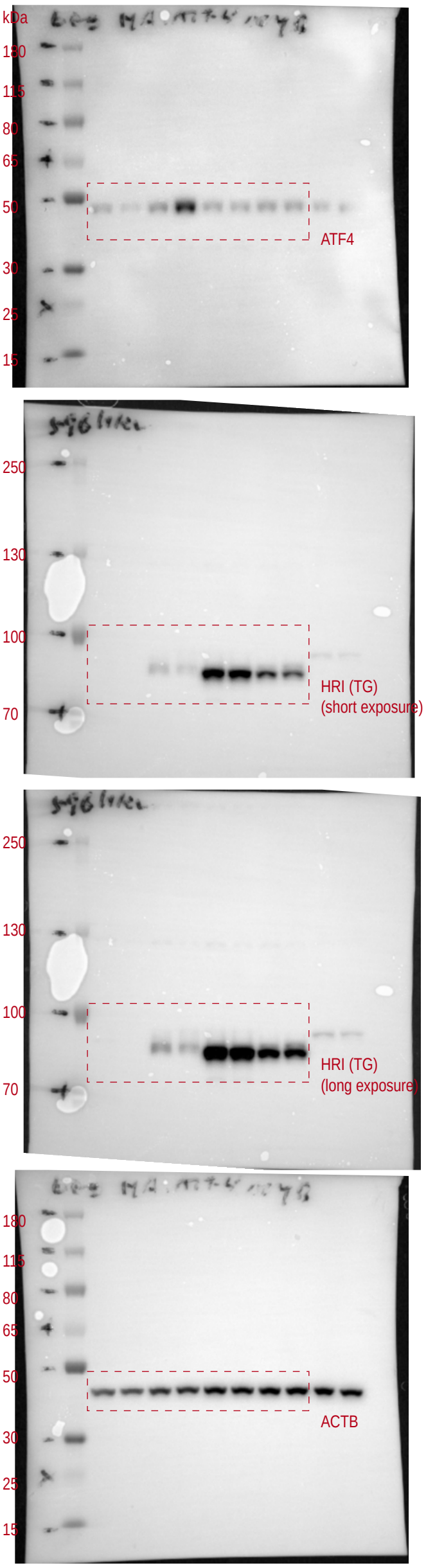

Extended Data Figure 8s

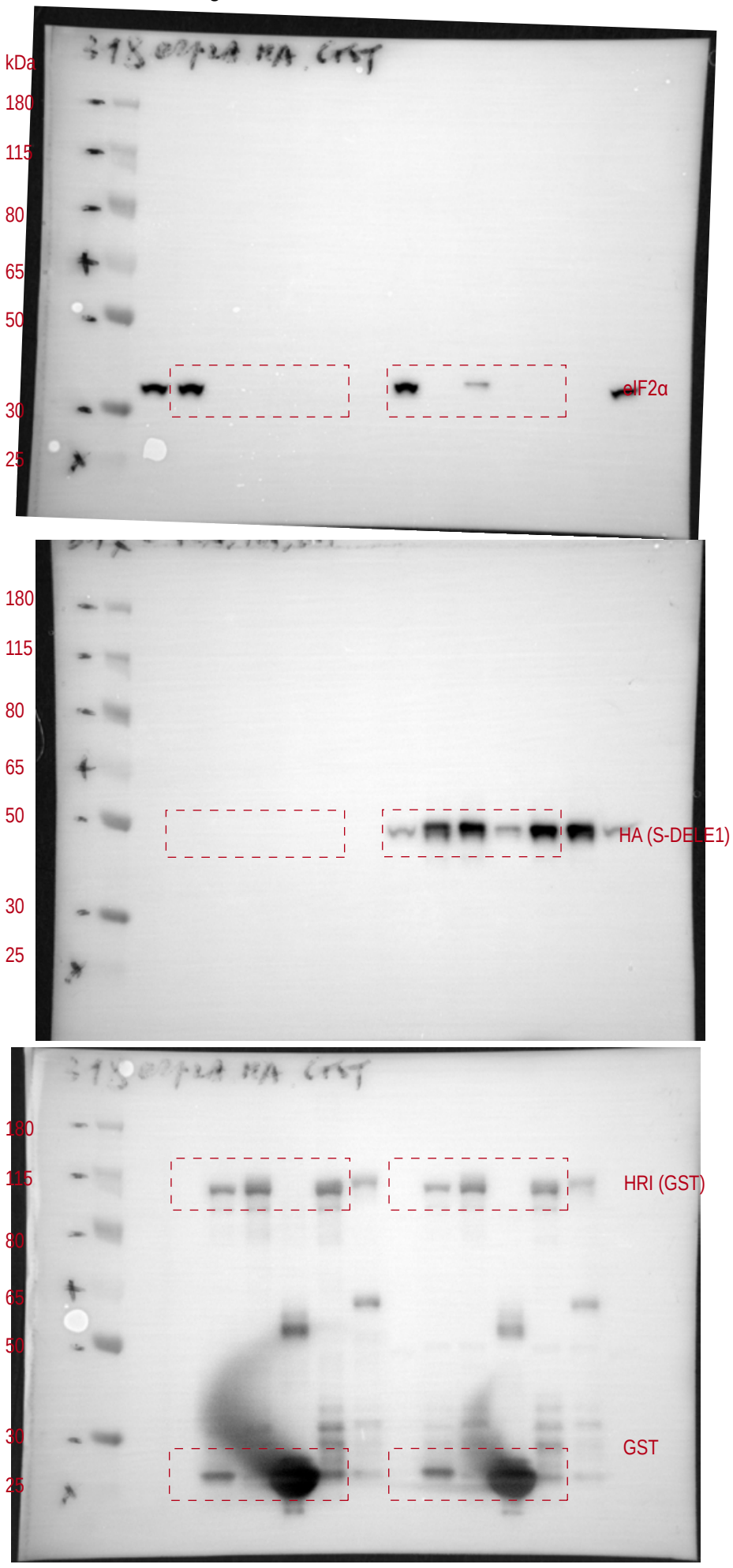

Extended Data Figure 8t

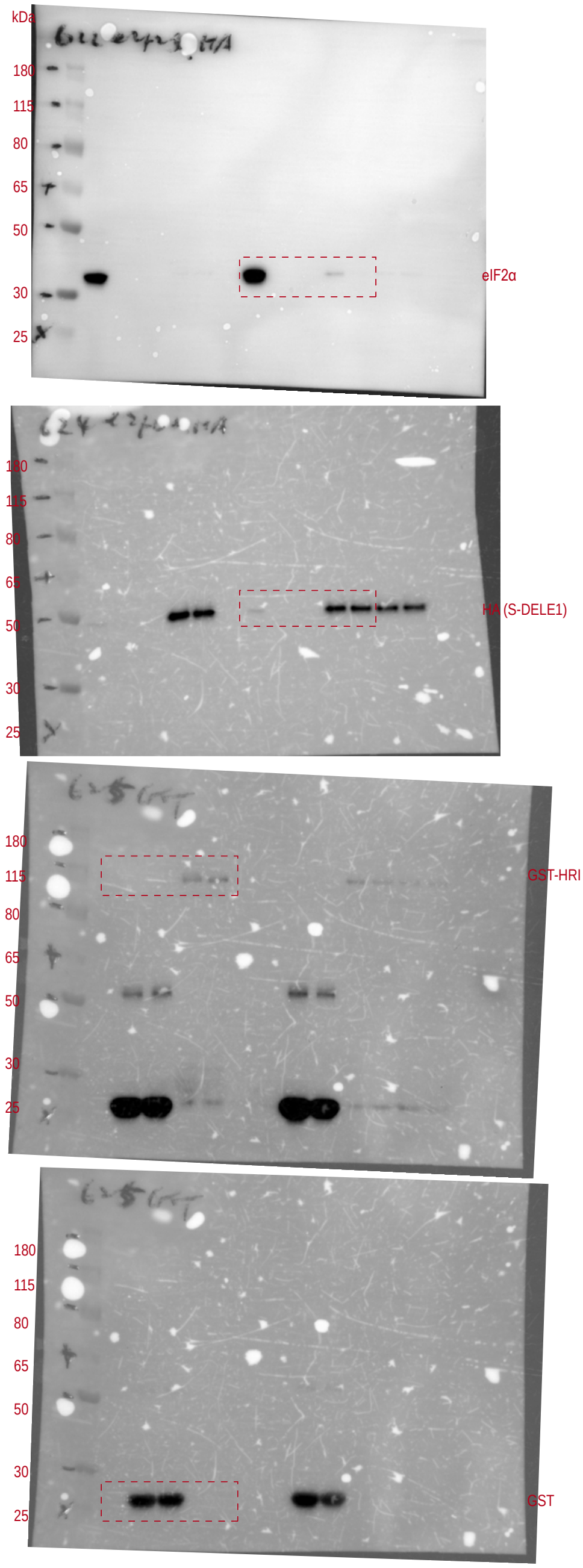

Extended Data Figure 9a

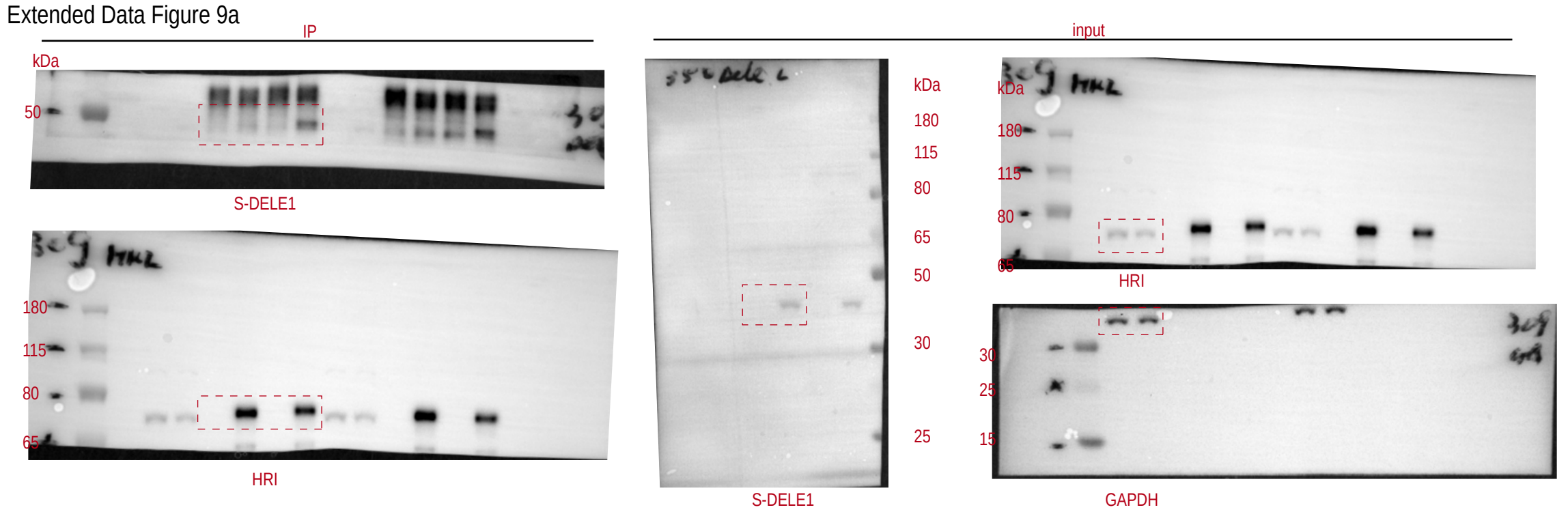

Extended Data Figure 9b

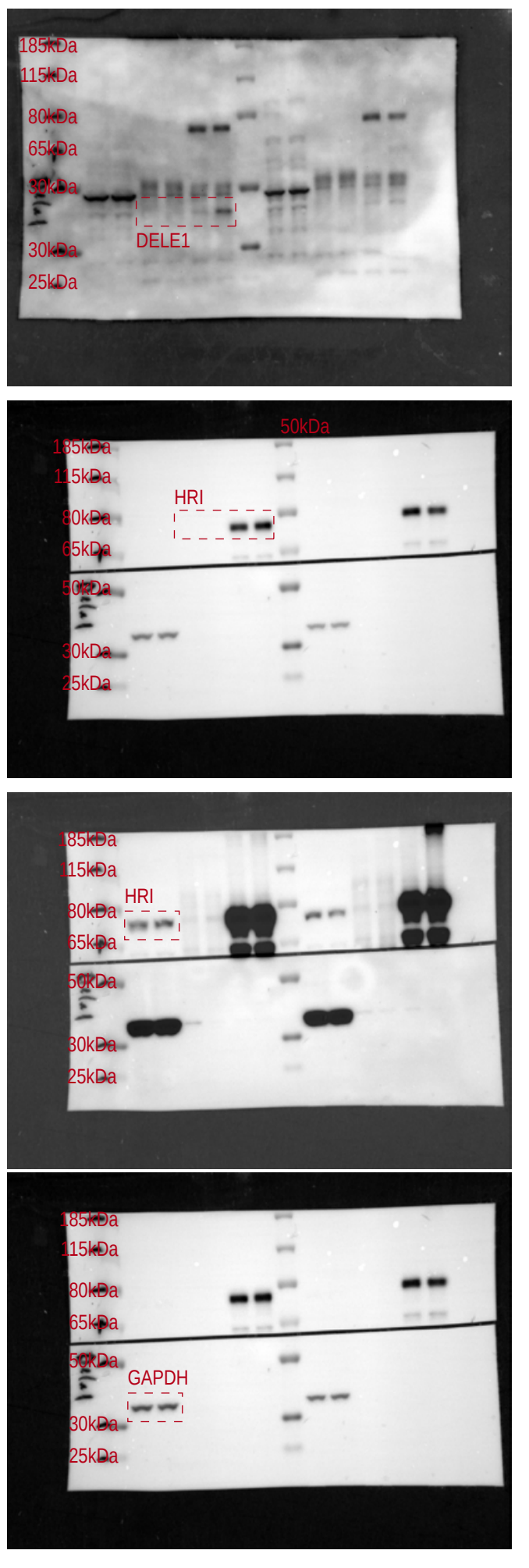

Extended Data Figure 9c

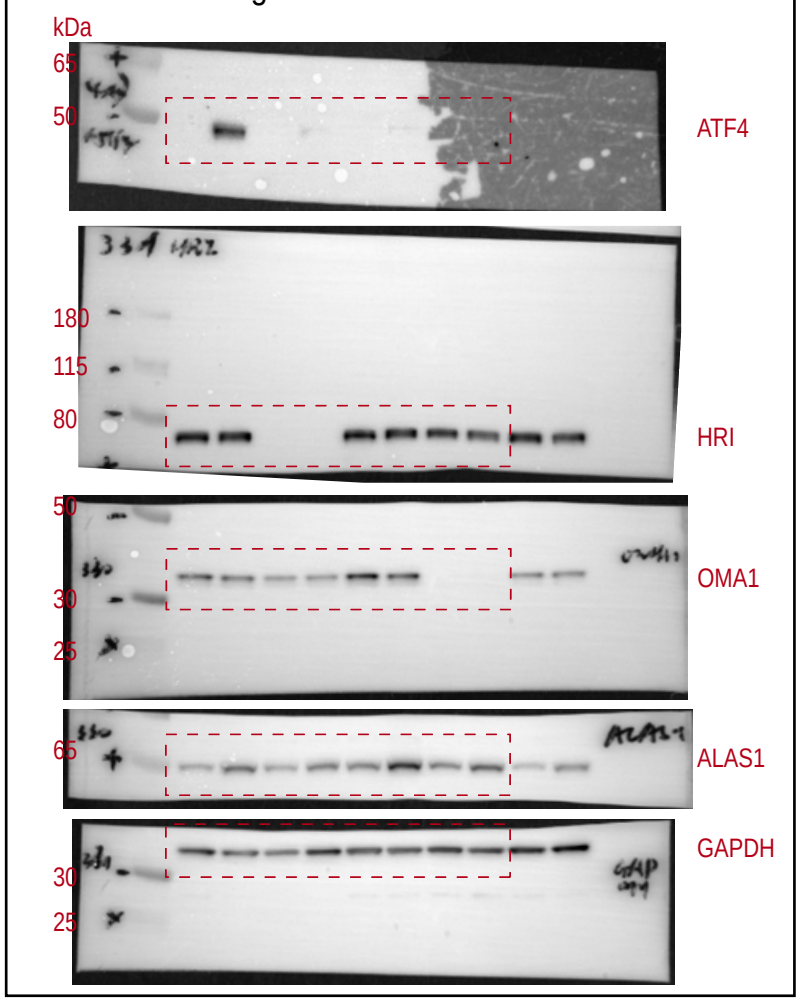

Extended Figure 9d

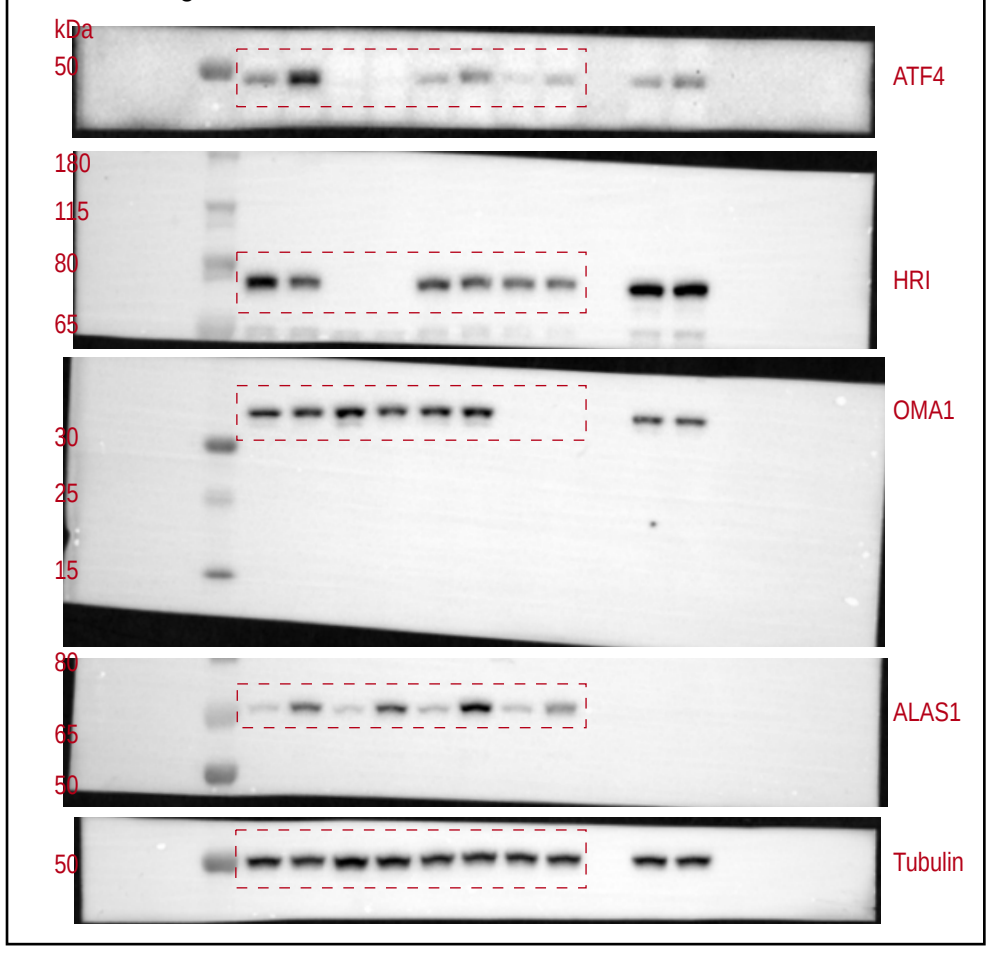

Extended Data Figure 9e

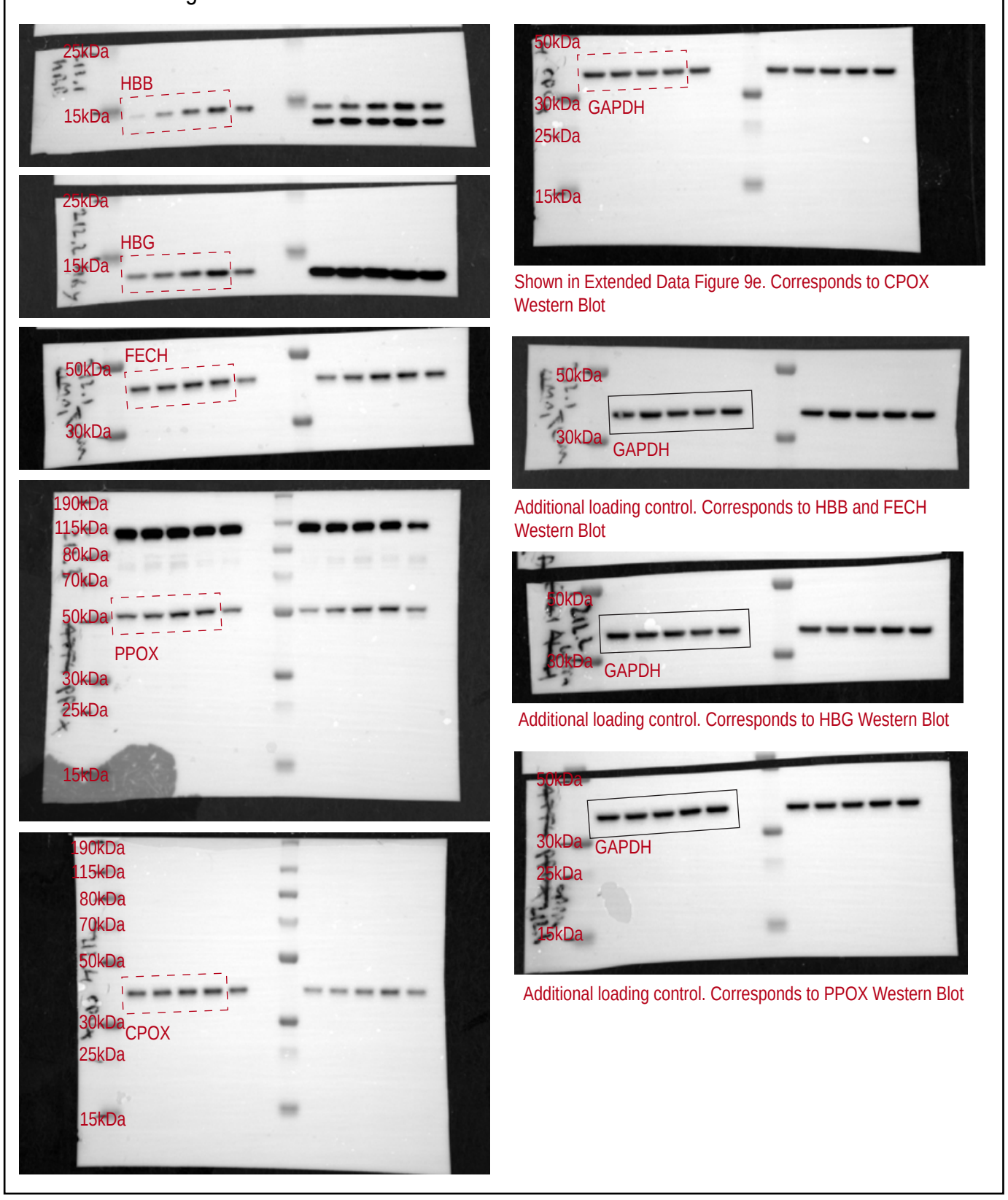

Extended Data Figure 9h

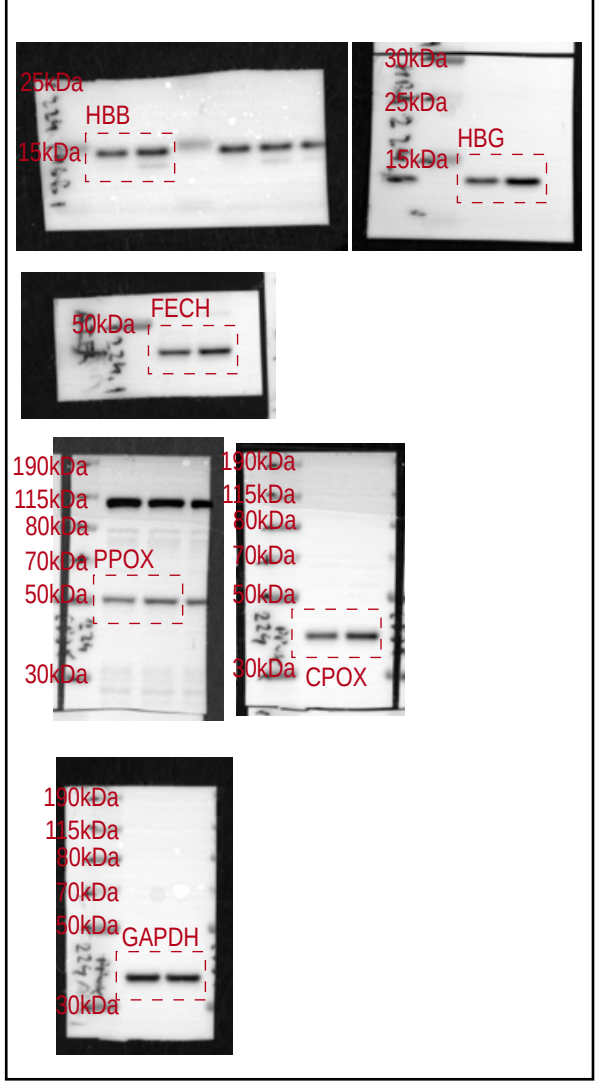

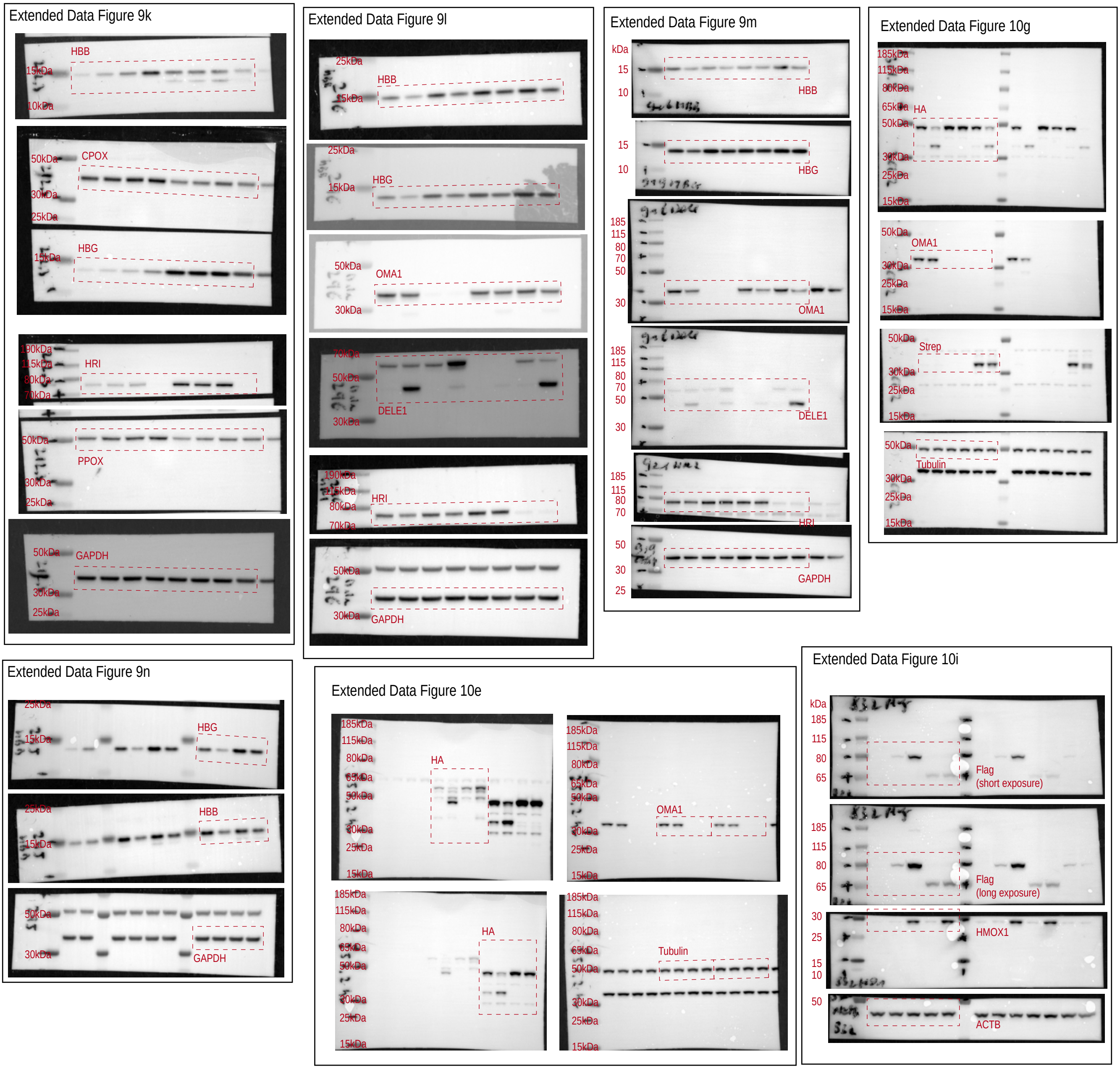

Supplementary Figure 1. Uncropped immunoblots and protein gels. Each box is labelled with its corresponding figure or Extended Data figure. All antibodies, markers, and cropping boxes are indicated in red.
