# Supplementary material for: An ancient mitochondrial program tunes translation to haem availability
Source: Nature. 2026 Aug 5;657(8133):1027–34. doi: 10.1038/s41586-026-10885-x (PMC13600944; doi:10.1038/s41586-026-10885-x)
Supplement: Supplementary file 3 [file 41586_2026_10885_MOESM2_ESM.pdf]

nature portfolio

Corresponding author(s): Lucas T. Jae

Last updated by author(s): Jun 12, 2026

Reporting Summary

Nature Portfolio wishes to improve the reproducibility of the work that we publish. This form provides structure for consistency and transparency in reporting. For further information on Nature Portfolio policies, see our Editorial Policies and the Editorial Policy Checklist.

Statistics

For all statistical analyses, confirm that the following items are present in the figure legend, table legend, main text, or Methods section.

n/a	Confirmed	
☐	☒	The exact sample size (*n*) for each experimental group/condition, given as a discrete number and unit of measurement
☐	☒	A statement on whether measurements were taken from distinct samples or whether the same sample was measured repeatedly
☐	☒	The statistical test(s) used AND whether they are one- or two-sided<br>*Only common tests should be described solely by name; describe more complex techniques in the Methods section.*
☒	☐	A description of all covariates tested
☐	☒	A description of any assumptions or corrections, such as tests of normality and adjustment for multiple comparisons
☐	☒	A full description of the statistical parameters including central tendency (e.g. means) or other basic estimates (e.g. regression coefficient) AND variation (e.g. standard deviation) or associated estimates of uncertainty (e.g. confidence intervals)
☐	☒	For null hypothesis testing, the test statistic (e.g. *F*, *t*, *r*) with confidence intervals, effect sizes, degrees of freedom and *P* value noted<br>*Give P values as exact values whenever suitable.*
☒	☐	For Bayesian analysis, information on the choice of priors and Markov chain Monte Carlo settings
☒	☐	For hierarchical and complex designs, identification of the appropriate level for tests and full reporting of outcomes
☒	☐	Estimates of effect sizes (e.g. Cohen's *d*, Pearson's *r*), indicating how they were calculated

Our web collection on statistics for biologists contains articles on many of the points above.

Software and code

Policy information about availability of computer code

Data collection	For data collection the following software was used: BD FACSDiva 8.0.1 and 8.0.2, FusionCapt Advance FX7 17.04a, Refeyn AcquireMP 2.3, Tecan SparkContol version 3.1, Image Lab version 5.2, Jasco Spectra Manager II, Wave 2.6.1, AlphaFold 3, and Leica Application Suite X version 3.5.7.
Data analysis	Calculations of statistical significance were performed using GraphPad Prism version 10. For data analysis the following software was used: FlowJo 10.4, BD FACSDiva 8.0.1 and 8.0.2, Bowtie 1.0.1, intersectBED 2.26.0, UCSF ChimeraX version 1.8, Refeyn DiscoverMP 2.3, MaxQuant version 2.4.0.0, Perseus version 1.6.15.0, Proteome Discoverer version 2.5.0.400, ImageJ version 1.54f, Seahorse Analytics version 1.0.0.796, Fiji version 2.16.0/1.54p, Skyline-daily version 25.1, MUSCLE version 3.8.31, and ConSurf web server (https://consurf.tau.ac.il/).

For manuscripts utilizing custom algorithms or software that are central to the research but not yet described in published literature, software must be made available to editors and reviewers. We strongly encourage code deposition in a community repository (e.g. GitHub). See the Nature Portfolio guidelines for submitting code & software for further information.

Data

Policy information about availability of data

All manuscripts must include a data availability statement. This statement should provide the following information, where applicable:

- Accession codes, unique identifiers, or web links for publicly available datasets
- A description of any restrictions on data availability
- For clinical datasets or third party data, please ensure that the statement adheres to our policy

The raw data for HRI crosslinks in presence of absence of S-DELE1 is available via proteomeXexchange with identifier PXD063587. The raw data for overexpressed HRI phosphorylation in presence of absence of S-DELE1 is available via proteomeXchange with identifier PXD062916. The raw data for endogenous HR1 phosphorylation can be accessed on the Panorama web repository server and under the PRIDE identifier PXD074030. Deep sequencing datasets have been deposited at the NCBI Sequence Read Archive under the accession code PRJNA1263803. The human reference genome assembly hg19 is available from NCBI (PRJNA31257).

Research involving human participants, their data, or biological material

Policy information about studies with human participants or human data. See also policy information about sex, gender (identity/presentation), and sexual orientation and race, ethnicity and racism.

Reporting on sex and gender	This information has not been collected as no human participants took part and no human data/samples were used in the study.
Reporting on race, ethnicity, or other socially relevant groupings	This information has not been collected as no human participants took part and no human data/samples were used in the study.
Population characteristics	This information has not been collected as no human participants took part and no human data/samples were used in the study.
Recruitment	This information has not been collected as no human participants took part and no human data/samples were used in the study.
Ethics oversight	This information has not been collected as no human participants took part and no human data/samples were used in the study.

Note that full information on the approval of the study protocol must also be provided in the manuscript.

Field-specific reporting

Please select the one below that is the best fit for your research. If you are not sure, read the appropriate sections before making your selection.

☒ Life sciences ☐ Behavioural & social sciences ☐ Ecological, evolutionary & environmental sciences

For a reference copy of the document with all sections, see nature.com/documents/nr-reporting-summary-flat.pdf

Life sciences study design

All studies must disclose on these points even when the disclosure is negative.

Sample size	No statistical method was used to predetermine the sample size. Sample sizes were determined on the basis of previous studies using similar methodologies performed by other groups in the field. For the genome-wide genetic screen, 2.19 × 10^7 single cells were interrogated phenotypically. For heme measurements, at least n = 3 independent biological replicates were performed, with each experiment containing technical duplicates or triplicates. For mass spectrometry experiments, at least n = 3 independent biological replicates were analyzed in the same run. Immunoblots and protein gels are representative of at least n = 3 independent biological replicates. For mitochondrial morphology analysis, n = 3 independent biological replicates were analyzed, with each replicate containing at least 30 cells per condition. Analytical flow cytometry and Seahorse assays were performed with n = 3 independent biological replicates, with each experiment containing at least technical triplicates. Flow cytometry analyses measured at least 10,000 cells, and Seahorse assays measured at least 20,000 cells per technical replicate.
Data exclusions	No data were excluded from the analysis, except for one WT-DMSO replicate (of four) in the endogenous HRI phosphoproteomics run corresponding to Extended Data Fig. 8j and one WT-DMSO replicate (of four) in the endogenous HRI phosphoproteomics run corresponding to Extended Data Fig. 8k. These samples were excluded due to insufficient data quality, reflected by systematically lower signal intensities.
Replication	For all experiments, at least n = 3 independent biological repeats (replicates) were performed. The precise samples size for each experiment is stated in the figure legends or the statistics section. All replication attempts were successful yielding comparable results.
Randomization	Randomization was not performed because the experiments did not involve allocation of subjects to treatment groups. Comparisons were made between predefined experimental conditions (e.g. genotypes or treatments) using identical experimental procedures. Where

applicable, plate positions were assigned without systematic patterning.

Blinding

Blinding was not performed because the experimental conditions (e.g. genotype or treatment) were inherently apparent during sample processing and no subjective analyses were performed.

Reporting for specific materials, systems and methods

We require information from authors about some types of materials, experimental systems and methods used in many studies. Here, indicate whether each material, system or method listed is relevant to your study. If you are not sure if a list item applies to your research, read the appropriate section before selecting a response.

Materials & experimental systems

n/a	Involved in the study
☐	☒ Antibodies
☐	☒ Eukaryotic cell lines
☒	☐ Palaeontology and archaeology
☒	☐ Animals and other organisms
☒	☐ Clinical data
☒	☐ Dual use research of concern
☒	☐ Plants

Methods

n/a	Involved in the study
☒	☐ ChIP-seq
☐	☒ Flow cytometry
☒	☐ MRI-based neuroimaging

Antibodies

Antibodies used

Antibody | Clone Number | Host Species | Company/Vendor | Cat. number | Application | Dilution factor
eIF2AK1 (HRI) | - | Rabbit | Proteintech | 20499-1-AP | IP, WB | 1/500-1,000
ATF4 | D4B8 | Rabbit | Cell Signalling Technologies | 11815S | WB | 1/1,000
CHOP | L63F7 | Mouse | Cell Signalling Technologies | 2895T | WB | 1/1,000
eIF2a | - | Rabbit | Cell Signalling Technologies | 9722S | WB | 1/1,000
Ph-eIF2a | D9G8 | Rabbit | Cell Signalling Technologies | 3398S | WB | 1/1,000
Ph-eIF2a | - | Rabbit | Cell Signalling Technologies | 9721S | WB | 1/1,000
HA | 16B12 | Mouse | BioLegend | 901503 | WB | 1/1,000
FLAG | M2 | Mouse | Sigma Aldrich | F1804 | IP, WB | 1/500-1,000
Strep | - | Rabbit | Abcam | Ab76949 | WB | 1/1,000
GST | - | Goat | Sigma Aldrich | GE27-4577-01 | WB | 1/1,000
OMA1 | D4J7K | Rabbit | Cell Signalling Technologies | 95473S | WB | 1/1,000
OPA1 | D7C1A | Rabbit | Cell Signalling Technologies | 67589S | WB | 1/1,000
OPA1 | Clone 18 | Mouse | BD Biosciences | 612606 | WB | 1/1,000
ALAS1 | EPR10247 | Rabbit | Abcam | Ab154860 | WB | 1/1,000
ALAS2 | - | Rabbit | ProteinTech | 30539-1-AP | WB | 1/1,000
HMOX1 | D60G11 | Rabbit | Cell Signalling Technologies | 5853S | WB | 1/1,000
PERK | - | Rabbit | ProteinTech | 20582-1-AP | WB | 1/1,000
FECH | - | Rabbit | ProteinTech | 14466-1-AP | WB | 1/1,000
GFP | - | Rabbit | Chromotetk | PABG1-100 | WB | 1/1,000
DELE1 | E9K6O | Rabbit | Cell Signalling Technologies | 98271S | WB | 1/1,000
HBB | 37-8 | Mouse | SantaCruz | Sc-21757 | WB | 1/1,000
HBG | EPR9708(B) | Rabbit | Abcam | Ab137096 | WB | 1/1,000
PPOX | - | Rabbit | ProteinTech | 14870-1-AP | WB | 1/1,000
CPOX | - | Rabbit | ProteinTech | 12211-1-AP | WB | 1/1,000
HMOX2 | - | Rabbit | ProteinTech | 14817-1-AP | WB | 1/1,000
BNIP3L (NIX) | D4R4B | Rabbit | Cell Signalling Technologies | 12396S | WB | 1/1,000
FTH1 | - | Rabbit | Cell Signalling Technologies | 3998S | WB | 1/1,000
Tubulin | 1E4C11 | Mouse | ProteinTech | 66031-1-AP | WB | 1/1,0000
GAPDH | 1E6D9 | Mouse | ProteinTech | 60004-1-AP | WB | 1/5,000
IgG control | - | Rabbit | ProteinTech | 30000-1-AP | IP | 1/500
Actin IgG-HRP | C4 | Mouse | SantaCruz | Sc-47778 | WB | 1/3,000
mNeonGreen | 32F6 | Mouse | Chromotek | 32F6 | FACS | 1/2,500
AF 488-labeled Goat anti-mouse | - | Goat | Thermo Fisher Scientific | A11001 | FACS | 1/500
Goat-anti-Mouse IgG-HRP | - | Goat | BioRad | 170-6516 | WB | 1/3,000
Goat-anti-Rabbit IgG-HRP | - | Goat | BioRad | 170-6515 | WB | 1/3,000
Donkey-anti-Goat IgG-HRP | - | Donkey | SantaCruz | sc-2020 | WB | 1/3,000
TrueBlot® ULTRA anti-Mouse IgG-HRP | eB144 | Rat | Rockland | 18-8817-30 | WB | 1/3,000
TrueBlot® ULTRA anti-Rabbit IgG-HRP | eB182 | Mouse | Rockland | 18-8816-31 | WB | 1/3,000

Validation

eIF2AK1(HRI): https://www.ptglab.com/products/EIF2AK1-Antibody-20499-1-AP.htm?srsltid=AfmBOorUU5pWdbYUbAyrPCSZbopJnbA64FirxAfb8CGtso72WUVUSPDg
ATF4: https://www.cellsignal.com/products/primary-antibodies/atf-4-d4b8-rabbit-mab/11815?srsltid=AfmBOorlvP5oDCuPsFE4SFw-PZxebvvxhkp_UZY8symqGP4mW6wwZAaN
CHOP: https://www.cellsignal.com/products/primary-antibodies/chop-l63f7-mouse-mab/2895?srsltid=AfmBOoqcQX6_oxftTWnhgheFhhe53WrNF8kCyZCd1krrr1BqgM7rQKr

eIF2a: https://www.cellsignal.com/products/primary-antibodies/eif2a-antibody/9722?srsltid=AfmBOofmJnQF_yA0rk8qfCTZnUKl7KSPGBt9NzMn-eHIA1ay_3DUhrx
Ph-eIF2a: https://www.cellsignal.com/products/primary-antibodies/phospho-eif2a-ser51-d9g8-xp-rabbit-mab/3398?srsltid=AfmBOooxxWSXobkmXUwr-m5bBJ5ZuIMqJ4m2tBC-__CPBCRMB1Z6S8UL
Ph-eIF2a: https://www.cellsignal.com/products/primary-antibodies/phospho-eif2-alpha-ser51-antibody/9721?srsltid=AfmBOooBs-Zk_bPadmiUQSutUzSEp--biE0asgafdh4_zUoV-GeuoieV
HA: https://www.biolegend.com/en-ie/products/purified-anti-ha-11-epitope-tag-antibody-11374
FLAG: https://www.sigmaaldrich.com/DE/en/product/sigma/f1804
Strep: https://www.abcam.com/en-us/products/primary-antibodies/strep-tag-ii-antibody-ab76949?srsltid=AfmBOorKYxfZh04xGgOqwsIMhES0NbkMaQ8ainmKgtC1bZtHeFWjN9_n
GST: https://www.sigmaaldrich.com/DE/en/product/sigma/ge27457701
OMA1: https://www.cellsignal.com/products/primary-antibodies/oma1-d4j7k-rabbit-mab/95473?srsltid=AfmBOoqKE-ykQ7rg67x1svV7SmLiCl7vUx5yoAGSRPnNFovB5z3sdb1P
OPA1: https://www.cellsignal.com/products/primary-antibodies/opa1-d7c1a-rabbit-mab/67589?srsltid=AfmBOopmm0XSRchTg9dvRd-d1csAkeiFoTAGujZUNI_XQ8zH92Ijh__7
OPA1: https://www.bdbiosciences.com/en-eu/products/reagents/microscopy-imaging-reagents/immunofluorescence-reagents/purified-mouse-anti-opa1.612606?tab=product_details
ALAS1: https://www.abcam.com/en-us/products/primary-antibodies/alas1-antibody-epr10247-mitochondrial-marker-ab154860?srsltid=AfmBOoo7i6Dwg4Q-nYExqCNGC8Y5k8Y442sdeJtgSzBHcp4eiLxiB4Wo1
ALAS2: https://www.ptglab.com/products/ALAS2-Antibody-30539-1-AP.htm?srsltid=AfmBOopOUBtkrr0fseOS-36Hd55tr-tEEfPPUdzy-2DN3llXuPaXQFNM
HMOX1: https://www.cellsignal.com/products/primary-antibodies/ho-1-d60g11-rabbit-mab/5853?srsltid=AfmBOorLuLapbX1IWnAuDDitLAzL7th-6djKgEvW0-jwKF25MNS3GVwA
PERK: https://www.ptglab.com/products/PERK-Antibody-20582-1-AP.htm?srsltid=AfmBOooG0sjOdK5A0ZQe7kYzuCcdnx0qmsxAl4HOVV_3ZUoMs-QQW3Ll
FECH: https://www.ptglab.com/products/FECH-Antibody-14466-1-AP.htm?srsltid=AfmBOopUVl-X95wWrJau2W11xmZsCFr0JEF-TXEQ6LcPCJsXfZ4cjiRQ
GFP: https://www.ptglab.com/products/GFP-antibody-rabbit-polyclonal-PABG1.htm?srsltid=AfmBOorQ2a5xo8s_nnCE9-fguBtaTsrMG3GZDVl6TIHf46feo1xQ1ATN
DELE1: https://www.cellsignal.com/products/primary-antibodies/dele1-e9k6o-rabbit-mab/98271?srsltid=AfmBOoq__4kOlGMlpEF6qWkllDDY-S3IH3RCw1r_gYQfBRUGWorvMg2-
HBB: https://www.scbt.com/p/hemoglobin-beta-antibody-37-8?srsltid=AfmBOopAg3jccNIU33uSUWt4rLB7VQAYx5G27fJ2dnOk1-BbTIGMhsDc
HBG: https://www.abcam.com/en-us/products/primary-antibodies/fetal-hemoglobin-antibody-epr9708b-ab137096?srsltid=AfmBOopAotk9RvnuLrt6qUHCIuYbgKaRE2v4aWs0d-dVh72j4py6G2Dq
PPOX: https://www.ptglab.com/products/PPOX-Antibody-14870-1-AP.htm?srsltid=AfmBOopdAkLix3Yk22hkQkNS8H72jjkbEpK8K4coRMU8QUJAyN2Y_FpC
CPOX: https://www.ptglab.com/products/CPOX-Antibody-12211-1-AP.htm?srsltid=AfmBOoqZnyGjkbEVFxAqJFTMofD3NcK3qOy_oP3-ehbG7AGfN3xy_R9R
HMOX2: https://www.ptglab.com/products/HMOX2-Antibody-14817-1-AP.htm?srsltid=AfmBOopUhFLr3cSGZWboAw7sSASPUAmJUN8oid0gfX76RlT8xB6DAH3S
BNIP3L (NIX): https://www.cellsignal.com/products/primary-antibodies/bnip3l-nix-d4r4b-rabbit-monoclonal-antibody/12396?srsltid=AfmBOopssa-3ubqf1O9kfWlgN78xG27Ss6pBhU1aIbWLc2vHwOPaWqUE
FTH1: https://www.cellsignal.com/products/primary-antibodies/fth1-antibody/3998
Tubulin: https://www.ptglab.com/products/tubulin-Alpha-Antibody-66031-1-Ig.htm?srsltid=AfmBOorCb3B0O5NGZHx8o0MirflFoatWDgYJqdjsvwJex1n-Xrmop4M9
GAPDH: https://www.ptglab.com/products/GAPDH-Antibody-60004-1-Ig.htm?srsltid=AfmBOopWHG-38ZVPy4VY5HLtZANJSbB_qKJpS6loW2Ze1GwXjX4aFpRj
IgG control: https://www.ptglab.com/products/IgG-control-Antibody-30000-0-AP.htm?srsltid=AfmBOootCwl04OVpCZLf2aq85CkiKkzUUhCtS9T8iJLyVbIAPHWZ_LJ4
Actin IgG-HRP: https://www.scbt.com/p/beta-actin-antibody-c4?srsltid=AfmBOoo9AQhgzq1-lJOMZsTSq9hkhyGxaBQ8HyBirA4yS8sPjRcMFmLc
mNeonGreen: https://www.ptglab.com/products/mNeonGreen-antibody-32F6.htm?srsltid=AfmBOoqIphMffTIvaQppsHS1dM2In2X_Cq77c_k6jwtrnifJw2YuYS5h
AF 488-labeled Goat anti-mouse: https://www.thermofisher.com/antibody/product/Goat-anti-Mouse-IgG-H-L-Cross-Adsorbed-Secondary-Antibody-Polyclonal/A-11001
Goat-anti-Mouse IgG-HRP: https://www.bio-rad.com/de-de/sku/1706516-goat-anti-mouse-igg-h-l-hrp-conjugate?ID=1706516
Goat-anti-Rabbit IgG-HRP: https://www.bio-rad.com/de-de/sku/1706515-goat-anti-rabbit-igg-h-l-hrp-conjugate?ID=1706515
Donkey-anti-Goat IgG-HRP: https://www.scbt.com/de/p/donkey-anti-goat-igg-hrp?srsltid=AfmBOoo6Fdeh-z-ONeuxp5CoFsgtIiGEHmFVd8odBNKKhvlk7d-jYWBj
TrueBlot® ULTRA anti-Mouse IgG-HRP: https://www.rockland.com/categories/trueblot/mouse-trueblot-ultra-anti-mouse-ig-hrp-18-8817-30/?srsltid=AfmBOop7EV4vzMrigUNJk7DyjkcspQUfwZQ8J1qfpSXgGIeWp5843BLl
TrueBlot® ULTRA anti-Rabbit IgG-HRP: https://www.rockland.com/categories/trueblot/rabbit-trueblot-ultra-anti-rabbit-igg-hrp-18-8816-31/?srsltid=AfmBOooIJ9QcIJ_9_FIUF3ZpxuTOM4sEnh5Q7vRG_-PGADpZojWnk2bP

Eukaryotic cell lines

Policy information about cell lines and Sex and Gender in Research

Cell line source(s)	Cells lines obtained from authenticated stocks were a kind gift from the Brummelkamp laboratory (HEK293T, HeLa, HCT116, A549, U2OS, BJEH (immortalized BJ) obtained from ATCC; HAP1 isolated in house and available from Horizon Discovery), from the Hornung laboratory (SH-SY5Y; DSMZ, no. ACC 209), and from the Stingele laboratory (K562; ATCC, no. CCL-243).
Authentication	Purchased cell lines were authenticated by source. During cultivation, cell lines were authenticated by monitoring phenotypic features, such as morphology and growth characteristics.

Mycoplasma contamination	All cell lines tested negative for mycoplasma.
Commonly misidentified lines (See ICLAC register)	No commonly misidentified cell lines were used in this study.

Plants

Seed stocks	This information has not been collected as no plants were in the study.
Novel plant genotypes	This information has not been collected as no plants were in the study.
Authentication	This information has not been collected as no plants were in the study.

Flow Cytometry

Plots

Confirm that:

☒ The axis labels state the marker and fluorochrome used (e.g. CD4-FITC).

☒ The axis scales are clearly visible. Include numbers along axes only for bottom left plot of group (a 'group' is an analysis of identical markers).

☒ All plots are contour plots with outliers or pseudocolor plots.

☒ A numerical value for number of cells or percentage (with statistics) is provided.

Methodology

Sample preparation	Cells were acquired and cultured according to Methods section. Before analysis, cells were trypsinized, homogenized and vortexed.
Instrument	BD LSRFortessa (BD, Franklin Lakes, New Jersey, USA), BD Fusion
Software	Data were analyzed using BD FACSDiva (BD, Franklin Lakes, New Jersey, USA) or FlowJo software (TreeStar Inc, Ashland, Oregon, USA).
Cell population abundance	For the genetic screen, 2.19×10^7 single cells were collected. Prior to genomic DNA isolation, several thousand sorted cells from each population were re-analysed to ensure the purity of the sorted populations. For flow cytometry analysis, at least 10,000 cells per technical replicate were analysed.
Gating strategy	For analytical flow cytometry or sorting of living or fixed cells, cells were first gated on FSC-A vs SSC-A to exclude debris. Subsequently, single cells were gated using the SSC-H vs SSC-A plot. Single cells were analyzed for fluorescence. A gating strategy is shown in the Extended Data Figure 5f.<br>For sorting of fixed cells for mutation mapping, cells were identified using the FCS-A vs SSC-A plot to exclude debris. Subsequently, single cells were identified using a DAPI-A vs DAPI-W plot. Single cells were displayed in a histogram, on which gating on 1n DNA content was performed to exclude diploid cells. Sorting was performed on the haploid cell population, the gates used for sorting were set to top 4% CHOP-Neon high and bottom 4% CHOP-Neon low. The gating strategy is shown in Extended Data Figure 2a.

☒ Tick this box to confirm that a figure exemplifying the gating strategy is provided in the Supplementary Information.
